# Synthesis of *trans*-2,5-Disubstituted Tetrahydro-1-benzazepines via Nucleophilic Ring Opening of Selectively Quaternized *N*,*N*‑Acetals

**DOI:** 10.1021/acs.joc.5c01958

**Published:** 2026-01-23

**Authors:** Chriss E. McDonald, Holly D. Bendorf, William G. Dougherty, Juan M. Martínez, Zoie V. Dodson, Cameron L. Upcraft, Dylan J. McGowan, Lilith M. Taylor, Josue Urbina

**Affiliations:** † Department of Chemistry and Biochemistry, 8606 Lycoming College, Williamsport, Pennsylvania 17701, United States; ‡ Department of Chemistry, 246462 Susquehanna University, Selinsgrove, Pennsylvania 17870, United States

## Abstract

A one-pot, two-step sequence involving a regiospecific quaternization of readily available aryl-fused *N*,*N*-acetals followed by a stereospecific nucleophilic ring opening has been developed. A wide range of nucleophiles have been shown to be compatible with this reaction sequence. This operationally simple methodology provides an effective route for the synthesis of a variety of *trans*-2,5-disubstituted tetrahydroarylazepines. In a demonstration of the utility of this reaction sequence, mozavaptan, a compound that is beneficial in the treatment of hyponatremia, has been efficiently prepared.

## Graphical abstract

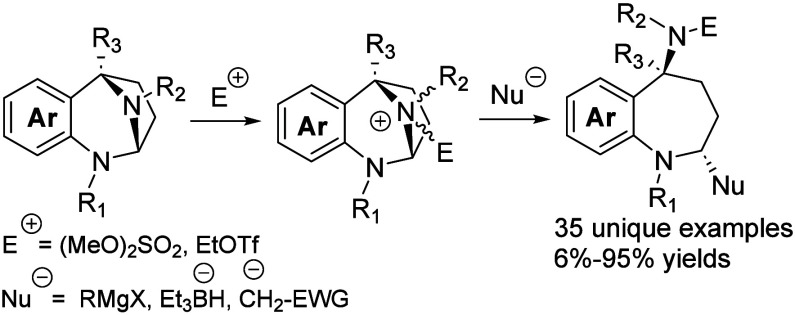

## Introduction

Tetrahydro-1-benzazepines and their more highly oxidized congeners have proven to be useful compounds with a host of pharmacologically relevant activities. Mozavaptan^1^ (**1**) and tolvaptan^2^ (**2**), are known to function as vasopressin V2 receptor antagonists and are used clinically to mitigate hyponatremia by inducing hypotonic diuresis (Scheme 1).^3^ The related compound zilpaterol (**3**) functions as a β2 adrenergic receptor agonist used as a feed supplement for beef cattle.^4^ Benazepril (**4**), an ACE inhibitor, is used in the treatment of hypertension and heart failure.^5^

**Scheme 1:**
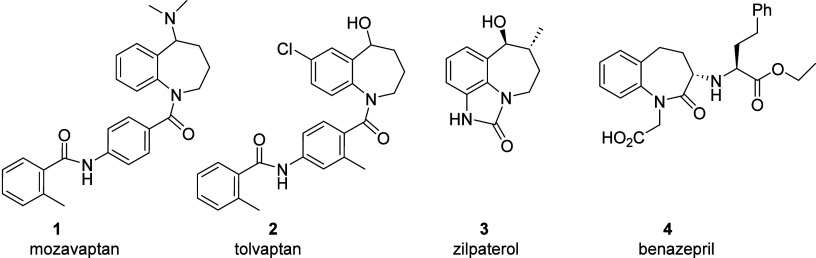
1-Benzazepine-Based Pharmaceuticals

Traditional routes to 1-benzazepinones have begun with 1-tetralone and employed either the Schmidt rearrangement^6^ or the Beckman rearrangement.^7^ Boekelheide constructed benzazepinones efficiently via a Dieckmann condensation route.^8^ More modern methods have been introduced. Appropriately substituted 2-vinyloanilino amides can be converted to dihydro-1-benzazepinones upon treatment with Tf_2_O and base.^9^ Zard has developed a radical pathway for the construction of 1-benzazapinones from xanthate precursors.^10^ An intramolecular Heck arylation was employed by Boger for the synthesis of the benzazepine nucleus.^11^ Hii has reported a ring closure metathesis route from diallylated acetanilides.^12^ Bendorf has designed a rhodium-catalyzed procedure for the construction of 1-benzazepinones from 2-(*N*-butenyl)­benzaldeydes.^13^ Sutherland has disclosed a one-pot process involving a 3,3-sigmatropic rearrangement of trichloroacetimidates followed by ring closure metathesis to afford 2,3-dehydro-1-benzapepines.^14^ Dihydro-1-benzazepines can also be synthesized by treatment of *N*-triflyl-2-vinylanilides via a cooperative catalytic system with Pd­(OAc)_2_ and Cu­(OAc)_2_.^15^ Doye has developed a hydroaminoalkylation/Buchwald-Hartwig sequence which converts 4-(2-bromophenyl)­1-butenes to tetrahydro-1-benzazepines.^16^ Recently Mo reported an elegant Pd^2+^ catalyzed [3 + 2] cycloaddition of *N*-aryl nitrones with allenoates to produce 1-benzazepines with three contiguous chiral centers.^17^ Very recently, Wu and co-workers revealed an enantioselective Rh­(II)-catalyzed cycloisomerization/nucleophilic addition strategy which affords 1-dihydrobenzazapines.^18^ Interestingly, this reaction is compatible with both oxygen and carbon-based nucleophiles.

We were intrigued by the possibility of using differentially substituted *N*,*N*-acetals as precursors for tetrahydro-1-benzazepines. The *N*,*N*-acetals are conveniently prepared in a single step from 2-(allylamino)­aryl aldehydes and primary amines. The transformation begins with imine formation and is followed by an ene reaction. Protonation of the resultant enamine and subsequent intramolecular attack of the aliphatic amine on the iminium ion affords the benzo-fused bicyclic *N*,*N*-acetal.^19–21^

It has been shown that pyridyl-fused *N*,*N*-acetals can be regioselectively reduced with NaBH_3_CN/AcOH to afford nicotine derivatives. This reaction relies on the greater nucleofugacity of the pyridylic portion of the *N*,*N*-acetal (Scheme 2a).^22^ Yu has recently demonstrated that the related *N*,*O*-acetals can be regioselectively opened with Grignard reagents to produce benzazepines.^23^ Interestingly, this reaction usually results in the cis diastereomer as the major product (Scheme 2b). The key step in our synthetic sequence (Scheme 2c) involves the selective activation of the more nucleophilic aliphatic nitrogen of the bridged *N*,*N*-acetal with dimethyl sulfate such that it, and not the anilinic nitrogen, serves as the leaving group in subsequent substitution reactions. The activated species, a quaternized *N*,*N*-acetal, can then be opened with a variety of nucleophiles in a two-reaction one-pot *anti*-specific process. This reaction sequence opens the five-membered ring of the bicyclic acetal while preserving the more synthetically interesting seven-membered ring.

**Scheme 2:**
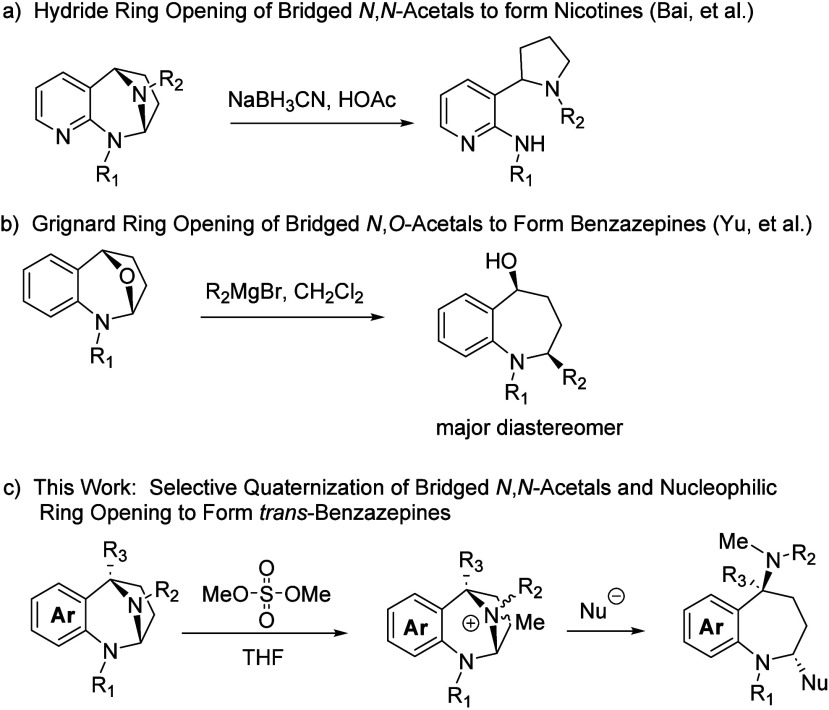
Nucleophilic Ring Opening of Acetals

## Results and Discussion

The first task was construction of **6a** and **6b**, the initial aza-bridged *N*,*N*-acetal substrates. Treatment of the known 2-*N*-allylbenzaldehyde **5** ^24^ with a primary amine and catalytic *p*-TsOH and azeotropic removal of water, afforded the requisite acetals **6** via the imino-ene reaction (Scheme 3).

**Scheme 3:**
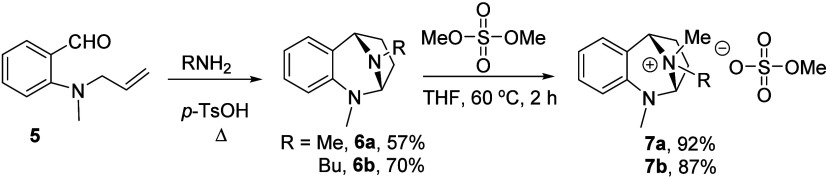
Selective Quaternization of Bridged *N*,*N*-Acetals

It should be noted that the reaction proceeds more efficiently with *n*-butylamine in toluene than methylamine (used as a solution in THF with added toluene) simply based on the different volatilities of the amines and the achievable temperature of the reaction mixture. In later experiments, when the reactions with methylamine were run in pressure tubes in the presence of molecular sieves, yields similar to those observed in the butylamine reactions were achieved.

Each of the pro-electrophilic *N*,*N*-acetals were treated with dimethyl sulfate in THF. While no reaction was observed at room temperature, the reactions proceeded to completion within 2 h at 60 °C. Removal of volatiles and ^1^H NMR analyses of the crude reaction mixtures indicated that the desired selectivity was realized. The solid methyl quaternary salt was found to be a single ionic compound. The butyl analogue, a viscous liquid, was found to consist of a 62:38 ratio of diastereomers as determined by integration of the ^1^H NMR spectra. These results are consistent with the known selective quaternization of the *N*,*N*-acetal eseroline by dimethyl sulfate.^25^ No evidence of THF solvent ring opening was observed. This can be a significant problem when THF is employed under strongly electrophilic conditions.^26^ Quaternary salts **7a** and **7b** are surprisingly stable. They can be kept for up to 18 months at −10 °C, with no discoloration or changes in their ^1^H NMR spectra.

Confident in our ability to activate the desired aliphatic nitrogen, we turned our attention to opening the bridging ring of **7b** with common phenyl nucleophiles (Table 1). The two-step, one-pot sequence began with the formation of the quaternized species in THF. The THF solution of the quaternized intermediate was subsequently cooled to the indicated temperature and the nucleophile was added. Phenylzinc bromide, a mild nucleophilic phenyl source which has been used for the arylation of isochromans under oxidative conditions,^27^ yielded no observable product when added at −84 °C and allowed to warm to room temperature or when added at room temperature and heated to 40 °C (entries 1 and 2). Diphenylzinc, as reported by Hevia and co-workers, efficiently adds to *N*,*O*-acetals.^28^ Addition of Ph_2_Zn to **7b** at −84 °C, and allowing the reaction to warm to room temperature overnight, returned only starting material.

**Table 1:**
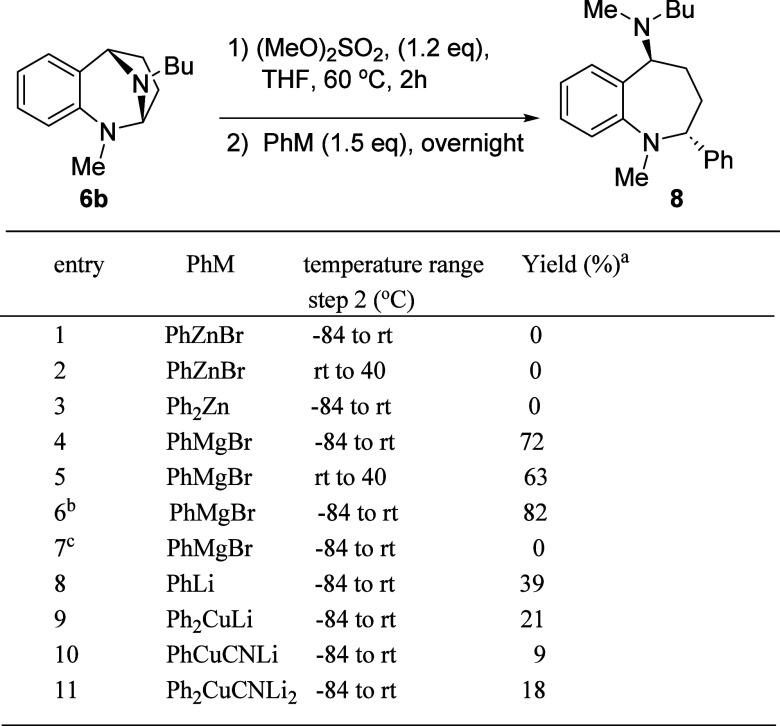
Quaternization and Ring Opening of *N*,*N*-Acetal **5b**

a Yields refer to the isolated, purified product.

b PhMgBr (2.0 equiv).

c (MeO)_2_SO_2_ not added.

Grignard reagents have been shown to be useful for opening cyclic *N*,*O*-acetals.^23,29,30^ Phenylmagnesium bromide (entries 4–6), proved to be an effective nucleophile for the quaternized *N*,*N*-acetals, as well, providing an excellent yield of the 2,5-disubstituted benzazepine **8**. Raising the temperature of the Grignard reaction did not improve the outcome of the reaction (entry 5). However, an increase in the equivalents of phenylmagnesium bromide did provide an improved yield of **8** (entry 6). The atom economy of the activation-Grignard addition sequence is noteworthy; the only byproduct being the ionic compound MgBrOSO_3_CH_3_. As a control experiment, we added PhMgBr to the pro-electrophile **6b** directly (entry 7). Under these circumstances, no reaction was observed and 92% of the starting compound, **6b**, was recovered.

It should be noted that the crude ^1^H NMR spectrum of the addition of PhMgBr to quaternized **6b** shows no indication of a second diastereomer being present. Examination of minor byproducts from the reaction reveals the presence of small amounts of the starting *N*,*N*-acetal **6b** and traces (<1%) of alkene-containing species but there is no evidence of the *cis*-diastereomer, which suggests essentially a pure S_N_2 mechanism with complete inversion of configuration at the electrophilic carbon yielding a *trans*-2,5-disubstituted benzazepine (*vide infra*). Also of note, we do not see evidence of the alternative ring opening pathway which would lead to nicotine derivatives.

We were quite surprised when phenyllithium did not yield a similar result (entry 8). A complex product mixture was obtained from which a modest yield of the desired product could be isolated. This could be a result of the tendency of PhLi (supplied as a solution in Bu_2_O) to form aggregates in solution, as do most alkyl lithium species.^31,32^ Higher degrees of aggregation are known to diminish the reactivity of organolithiums.^33^ It is worth pointing out there is substantial evidence for monomeric PhLi to exist in an equilibrium with the dimeric form in THF, which constitutes a substantial portion of the reaction solvent mixture.^34^ The corresponding Grignard reagent, PhMgBr, exists in monomeric form, which undoubtedly contributes to the success of the ring opening.^35^ A series of three cuprate reagents were then evaluated (entries 9–11).^36,37^ These also yielded inferior results in comparison to the Grignard nucleophile. Except for the curious phenyllithium experiment these results align with the understood nucleophilicity of these metal-containing species.^38^

A variety of nucleophiles were added to **6b** via quaternized *N*,*N*-acetal **7b** to determine the generality of the reaction sequence (Table 2). Grignard reagents were successfully added to **6b** via its methyl quaternized intermediate (entries 1–5). Methyl, allyl, butyl, and 4-methoxyphenyl Grignard reagents provided similar product yields as the previous phenyl addition, which indicates that hybridization of the nucleophilic carbon atom is not a significant factor in the success of the reaction. Likewise, increased substitution at the nucleophilic atom in isopropylmagnesium bromide was not detrimental to the ring opening. It is reasonable to determine if solvent polarity played a role in our poor results for the ring opening of **6b** by PhLi, which is supplied in Bu_2_O (Table 1, entry 8). Therefore, an attempt was made to effect ring opening with MeLi, supplied in the more polar Et_2_O (entry 8). MeLi is known to be tetrameric in ethereal solvents.^31^ The MeLi attempt yielded no isolable product.

**Table 2:**
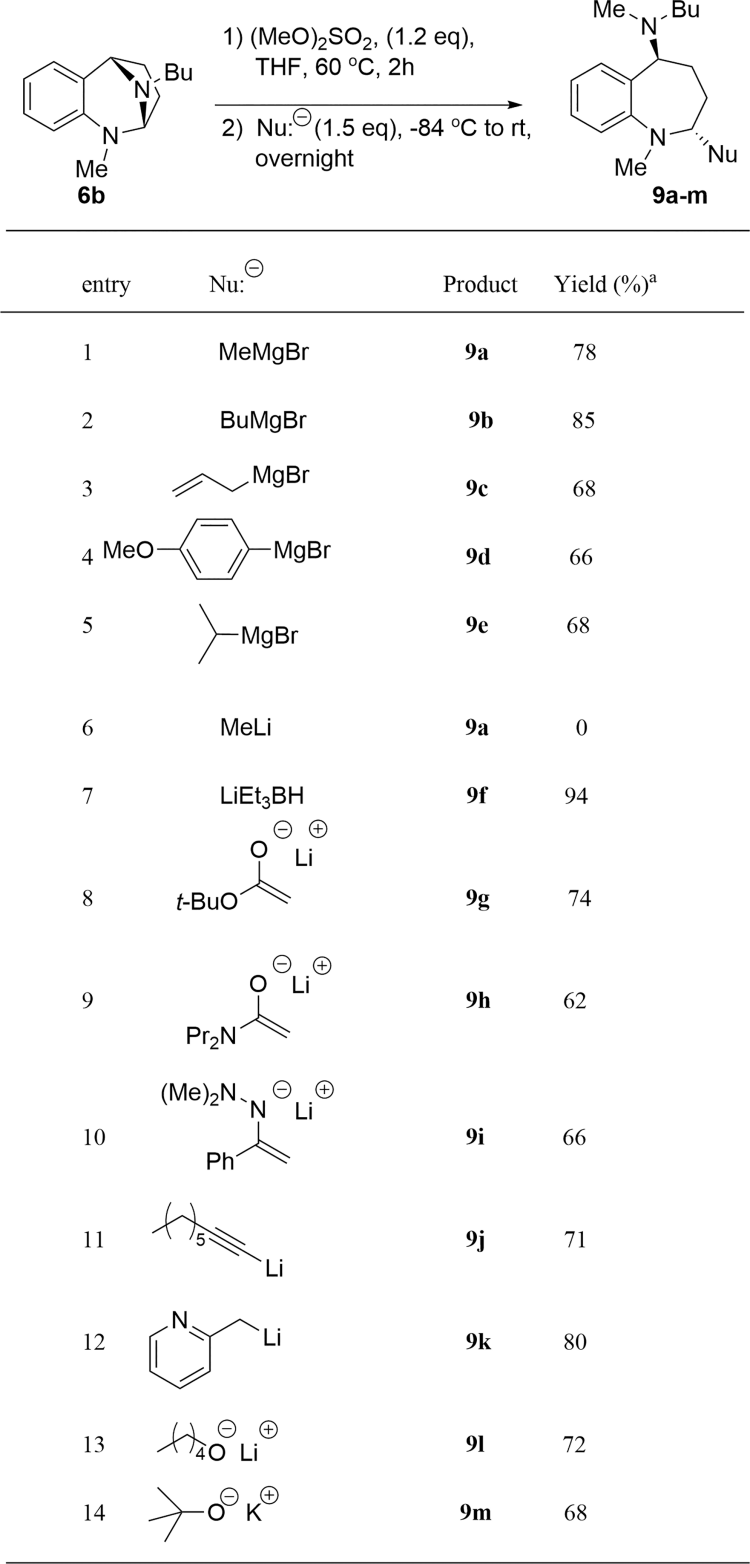
Addition of Various Nucleophiles to **6b**

a Yields refer to the isolated, purified product.

The superior nucleophile LiHBEt_3_ was examined next (entry 7).^39^ This reaction proceeded smoothly to provide an excellent yield of the reduced 1-benzazepine. It should be noted that this reaction is the regiospecific analogue of the Bai reduction of unactivated (neutral) *N*,*N*-acetals with NaBH_3_CN.^22^ Also of note, Husson has shown that unactivated *N*,*N*-acetals can be reduced with LiAlH_4_.^40^

Stabilized nucleophiles were then examined. It was anticipated that these would exhibit a lower level of reactivity with the quaternized *N*,*N*-acetal **7b**. The lithium enolate of *tert*-butyl acetate adds nicely, affording a 74% yield of ester **9g**. The enolate derived from *N*,*N*-dipropyl acetamide also adds, providing a 62% yield of the very polar amide, **9h**. Attempted addition of the enolate of acetophenone led to a complex mixture. The corresponding anion of the dimethylhydrazone derivative of acetophenone was successful, however.^41^ Lithiated 1-octyne added to **7b** to produce alkyne **9j**. We were quite gratified to observe that the anion derived from 2-methylpyridine also added leading to **9k**.^42^

It was deemed appropriate to attempt to add oxygen-based nucleophiles to quaternized *N*,*N*-acetal **7b** as well. The lithium salt of 1-pentanol added in very clean manner to provide a good yield of ring opened *N*,*O*-acetal **9l**. Surprisingly, even the bulky base potassium *t*-butoxide, which is often used to effect E2 reactions, added smoothly to provide **9m** in 68% yield.^43,44^ Examination of the crude ^1^H NMR of **9m** revealed no trace of elimination products. The two *N*,*O*-acetals **9l** and **9m** were of insufficient stability to allow for chromatographic purification using either silical gel or basic alumina.

Thus, we have demonstrated that a diverse set of nucleophiles can be used to open *N*,*N*-acetal **6b** via its quaternary ammonium cation in an operationally simple manner. Products **9a**–**9m** were typically accompanied by a small amount (1–4%) of returned **6b**, which may arise via misdirected nucleophilic attack at the quaternized *N*-methyl group. There was no evidence for elimination products. It should be noted that all compounds **9a**–**9m** were obtained as single diastereomers and were viscous oils.

Given that benzazepines **8** and **9a**–**9m** are oils, we devised a route to a crystalline amide derivative of our ring opened products (Scheme 4). Allylated *N*,*N*-acetal **10** was subjected to our activation/nucleophilic ring opening sequence with MeMgBr, providing benzazepine **11**. Examination of the crude ^1^H NMR of **11** provided no evidence for the presence of a second diastereomer (page S58). Deallylation using the method of Genêt^45^ followed by benzoylation provided **12** which proved to be a solid compound. Benzamide **12** was successfully crystallized by slow evaporation of a hexanes solution after column chromatography. Single-crystal diffraction analysis confirmed the *trans* relative stereochemistry between the resulting *N*-butyl-*N*-methylamine moiety at position 5 and the added methyl group at position 2 in the ring-opened product **11**. The fact that only a single relative geometry is being observed and that it is *trans* as indicated by the crystal structure determination of **12** substantiates the reaction mechanism as S_N_2.

**Scheme 4:**
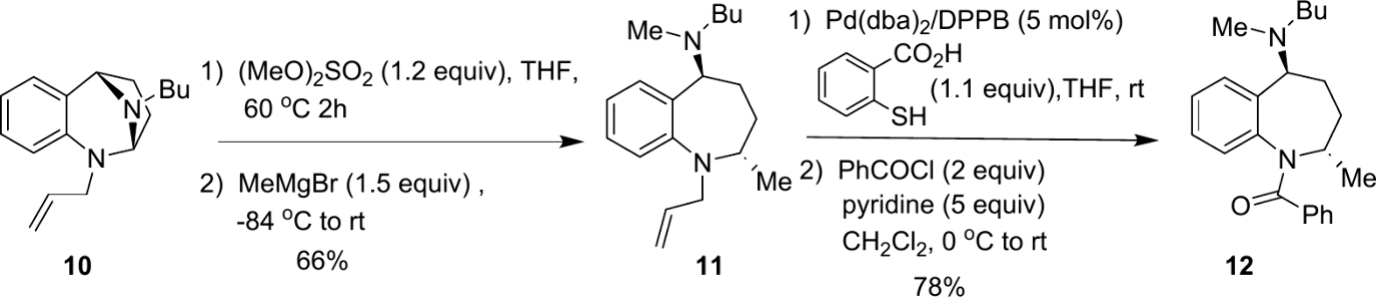
Determination of the Stereochemistry of the Ring Opening

The stereochemistry of products **8** (Table 1) and **9a**–**9e** and **9g**–**9k** (Table 2) can be assigned by correlation of ^1^H NMR spectra with the methyl-substituted benzazepine **11** which has been unambiguously determined via the crystal structure of **12** (see Figures 1 and 2 on pages S11–S13 for details).

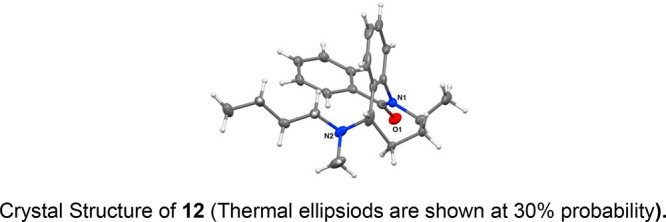



Having established the relative stereochemistry of the ring opening and the versatility of the nucleophilic component of the reaction sequence, we turned our attention to the *N*,*N*-acetal species. A series of compounds were constructed (Scheme 5) and subjected to the aforementioned quaternization-ring opening conditions with representative nucleophiles. These *N*-allylated *N*,*N*-acetals, along with **10** were selected to vary the electronic and steric characteristics of the electrophile formed upon quaternization.

**Scheme 5:**
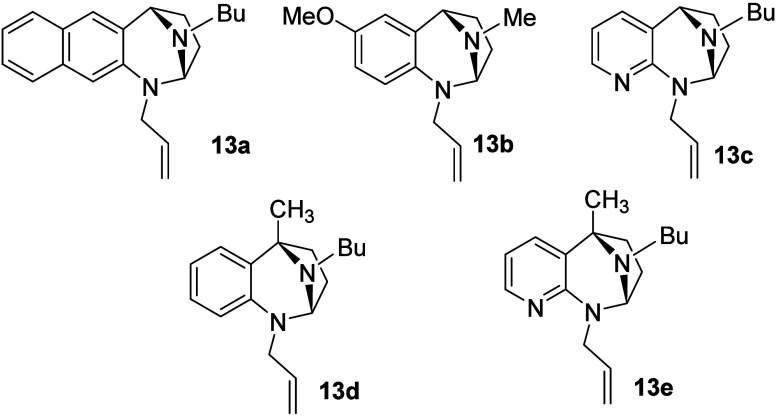
Additional *N*,*N*-Acetals for Study

Pro-electrophiles **10** and **13a**–**13e** were screened for their reactivity with various Grignard reagents (Table 3). The presence of the *N*-allyl protecting group does not compromise either the activation or nucleophilic ring opening components of the sequence (entry 1).

**Table 3:**
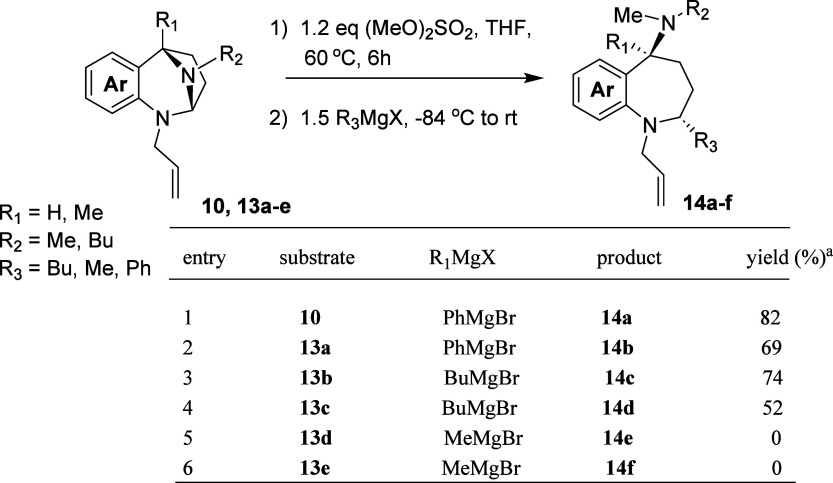
Addition of RMgX to Various *N*,*N*-Acetals

a Yields refer to the isolated, purified product.

Naphthyl-based *N*,*N*-acetals are likewise compatible with this chemistry (entry 2). The methoxy derivative **13b** proved interesting; the crude product was quite pure as shown by ^1^H NMR and afforded a high yield of ring opened product (entry 3). This suggests that the ring opening is facilitated by electron donation from the methoxy to the anilinic portion of the quaternized *N*,*N*-acetal. The pyridyl-based *N*,*N*-acetal **13c**, chosen as an electron withdrawing group compatible with the strongly nucleophilic conditions of the ring opening, provided the expected counterpoint (entry 4). The crude product was substantially impure with a great deal of the starting compound present (up to 46% of the mixture; see page S89) and a middling yield of 52% was realized upon purification.

The results obtained for **13b** and **13c** require further discussion. The ability of an appropriately located methoxy to facilitate S_N_2 reactions has considerable precedent. Fujio and co-workers have reported that displacement of 1-arylethyl bromides by pyridine in acetonitrile is dramatically accelerated by a *p*-methoxy moiety.^46^ Armstrong has recently disclosed a kinetic study which indicates that a *p*-methoxy substituent accelerates displacement of chloride by iodide in benzyl chlorides.^47^ Robiette, in an examination of the effect of adjacent π systems on S_N_2 reactions, observes that the ring closure of epoxides and aziridines by nucleophilic displacement is accelerated by the presence of electron donating substituents, such as *p*-methoxyphenyl, on the electrophilic carbon and decelerated by the presence of electron withdrawing groups.^48^ Computational studies performed by Robiette support the assertion that the electron-donating group lowers the energy of the transition state for S_N_2 mechanisms where there is significant bond breaking in the transition state. Our results suggest that the ring opening of the *N*,*N*-acetals occurs via a similar dissociative S_N_2 mechanism.

Surprisingly, placement of a methyl group at position 5 of the *N*,*N*-acetal, **13d**, completely disrupted the reaction (entry 5). The reaction of the methyl-substituted pyridyl analogue, **13e**, was also unsuccessful (entry 6). Although the quaternization steps for **13c** and **13e** were sluggish, presumably due to the greater steric congestion in the vicinity of the aliphatic amine of the *N*,*N*-acetal, the problem lies with the ring-opening step of the two-step reaction sequence. Material balance was poor in each of these reactions (entries 5 and 6), which is evidence for the formation of the water-soluble quaternized salts that would be washed-out in the aqueous workup.

The addition of LiBHEt_3_ to the diverse set of *N*,*N*-acetals **10** and **13a**–**13e** was then examined (Table 4). Yields for benzazepines **10**, **13a**, and **13b** (entries 1–3) are excellent as expected with the use of this strongly nucleophilic source of hydride. Substrate **13c** (entry 4) reacted cleanly but provided only a moderate yield of **15d**. Product **15e** isolated in 46% (entry 5), was accompanied by the isolation of substantial quantities of recovered starting material (25%), likely the result of nucleophilic attack at the methyl substituent of the quaternary amine. The methyl-substituted pyridyl-fused *N*,*N*-acetal, **13e**, continues to be the most problematic for ring opening (entry 6). Comparing the results in Tables 3 and 4, it is apparent that the LiBHEt_3_ ring openings are more efficient than the corresponding Grignard ring openings.

**Table 4:**
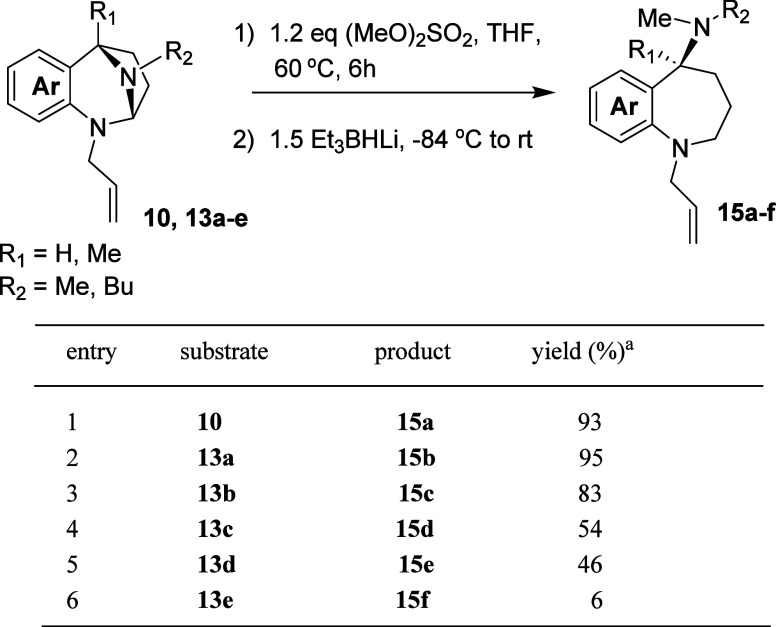
Addition of LIBHEt_3_ to Various *N*,*N*-Acetals

a Yields refer to the isolated, purified product.

To complete the study of nucleophilic ring opening of methyl quaternized *N*,*N* acetals, we subjected **10** and **13a**–**13e** to attack by lithiated 1-octyne (Table 5). These additions with a less reactive nucleophile were expected to be more problematic and that turned out to be true.

**Table 5:**
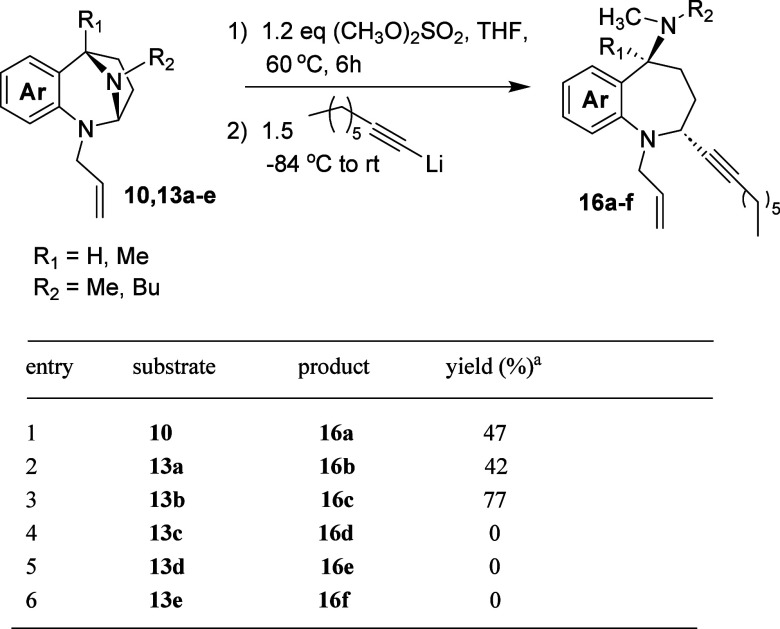
Addition of 1-Lithio-1-octyne to Various *N*,*N*-Acetals

a Yields refer to the isolated, purified product.

The reactions of substrates **10** and **13a** provided acceptable yields of the alkyne addition products **15a** and **15b**, which were isolated as single diastereomers (entries 1 and 2). Methoxy-substituted compound **13b** performed better (entry 3). This is consistent with the earlier observation that electron donation to the anilinic nitrogen of the *N*,*N*-acetal facilitates ring opening. The more challenging substrates **13c**–**13e** afforded no ring opened products, and material balance was poor, likely due to the loss of the unreacted quaternized *N*,*N*-acetal species to the aqueous layer during the reaction workup. Increasing the temperature of the ring-opening portion of the reaction sequence did not improve the outcome.

In an effort to determine whether the reluctance of *N*,*N*-acetal **13e** to yield ring opened products lies with the quaternization step or the ring opening step, we treated **13e** with dimethyl sulfate under the standard conditions and isolated the intermediate quaternized salt in the same manner as was done with **6a** and **6b** (Scheme 6). We were intrigued to find a single diastereomer resulted from this reaction, assigned as shown by NOE studies (page S112). The success of the quaternization indicates that the relative lack of success with this substrate is based on the ring opening portion of the reaction sequence.

**Scheme 6:**
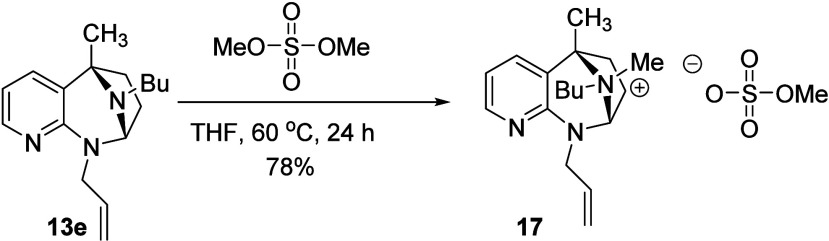
Quaternization of **13e** with (MeO)_2_SO_2_

To this point a method has been developed to afford an array of arylazepines via the activation a variety of aryl-fused *N*,*N*-acetals with dimethyl sulfate, followed by ring opening with a diverse set of nucleophiles. It is also apparent that challenges remain. Substrates **13c**–**13e** have proven to be less efficient in their ring opening, especially with stabilized nucleophiles. Likely problems with the ring opening include misdirected nucleophilic attack on the *N*-methyl moiety of the quaternized *N*,*N*-acetal intermediate, and perhaps more significantly, the lower reactivity of electron-poor and 5-methyl substituted cationic intermediates.

We were struck by the idea that these problems might be simultaneously mitigated by the introduction of a larger substituent on the aliphatic nitrogen atom during the quaternization step. The misdirected nucleophilic attack observed at the *N*-methyl substituent would be discouraged by a larger, more sterically congested group. Also, the intentional enhancement of the steric congestion at the aliphatic portion of the *N*,*N*-acetal might increase the rate of the nucleophilic displacement step given the concomitant relief of steric strain upon ring opening. Commercially available ethyl triflate has long been known as an excellent electrophile and quite useful for many synthetic applications.^49–51^ Thus, we opted to explore the two-step sequence with EtOTf as the electrophilic activator.

We initially treated substrate **13d** with EtOTf in THF. Not surprisingly, this reaction suffered from solvent incompatibility issues, likely due to the documented ring-opening polymerization of THF in the presence of EtOTf.^52^ The quaternization was then attempted in dichloromethane (Scheme 7). After 24 h at room temperature, ^1^H NMR analysis of the reaction mixture revealed an approximately 60:40 mixture of the diastereomeric *N*-ethyl-quaternized *N*,*N*-acetals, **18**. The analogous reaction was attempted with the commercially available TMSCH_2_OTf, but returned only starting material.

**Scheme 7:**
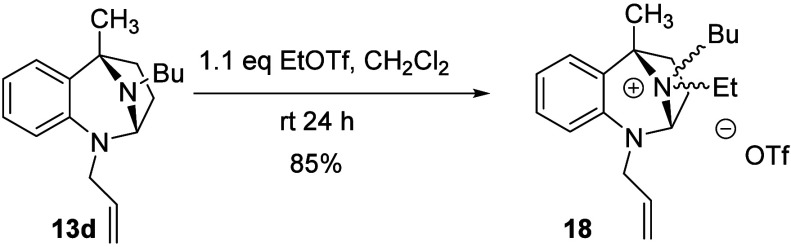
Quaternization of **13d** with EtOTf

Having secured a methodology for quaternization with EtOTf, we proceeded to react the more problematic substrates, **13c**–**13e**, with a variety of nucleophiles (Table 6). Pyridyl-based *N*,*N*-acetal **13c** was first quaternized with EtOTf as described above. Volatiles were removed *in vacuo* to provide a viscous oil. THF was added, which solubilized the salt, then the mixture was cooled to −84 °C and 1.5 equiv of BuMgCl were added. The mixture was warmed to room temperature overnight, then heated to 30 °C for 1 h (temperatures above 30 °C proved detrimental). The crude product was obtained cleanly, as evidenced by TLC and the ^1^H NMR spectrum. Upon purification the ring-opened product **19a** was isolated as a single diastereomer in 76% yield. This outcome is obviously superior to the similar dimethyl sulfate-based procedure (Table 3, entry 4). The EtOTf-based ring opening of **13c** with LiBHEt_3_ provided similarly improved results relative to the analogous dimethyl sulfate reaction (Table 4, entry 4). While the Grignard and hydride additions proceeded smoothly, the analogous reaction with the less reactive lithiated 1-octyne was unsuccessful, presumably due to the deactivating effect of the fused pyridine ring.

**Table 6:**
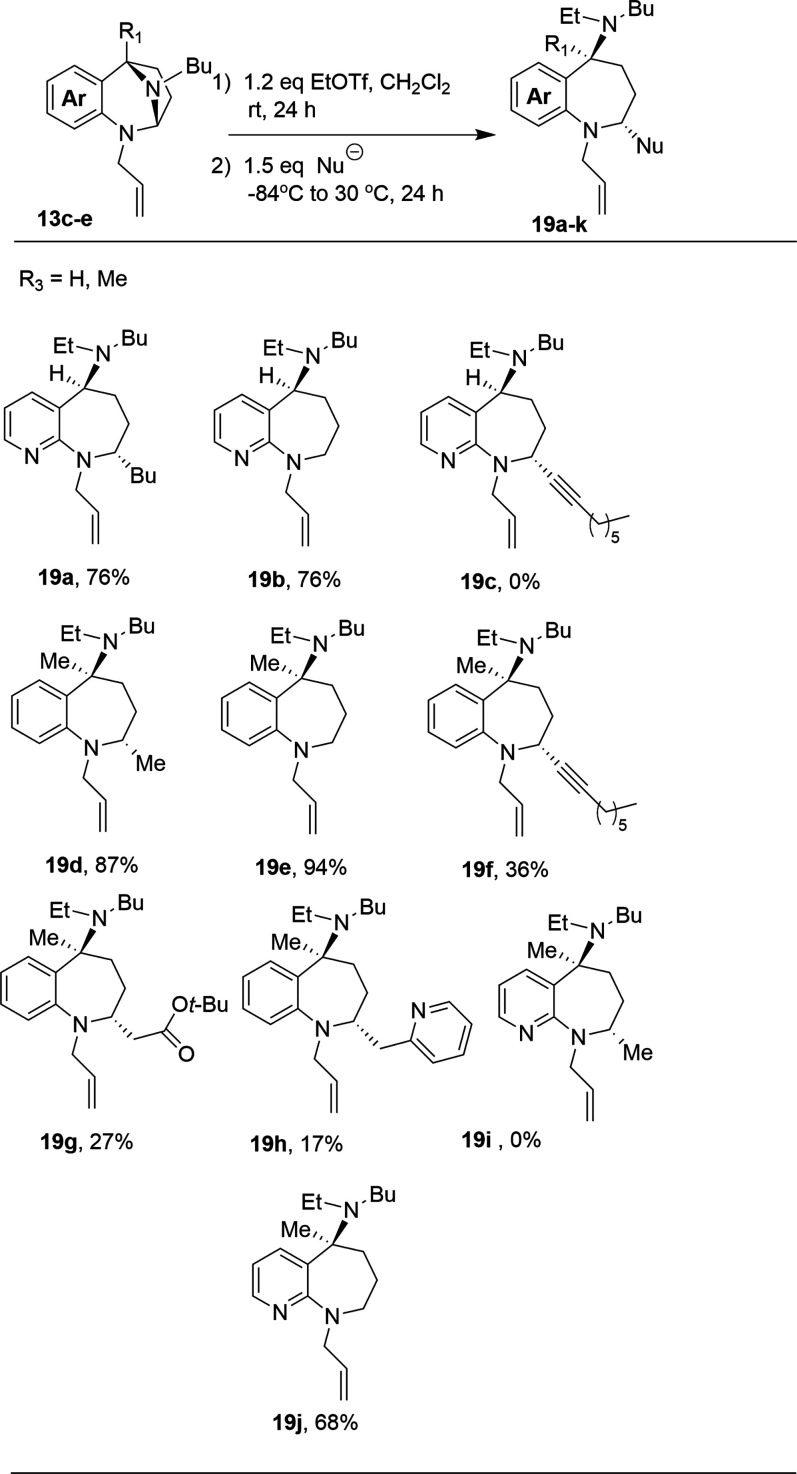
EtOTf-Based Ring Openings^a^

a Yields refer to the isolated, purified products.

The benzo-fused, 5-methyl substituted substrate, **13d**, was examined next. The intermediate **18** is soluble in THF and reaction with CH_3_MgBr provided the ring opened product, **19d**, in excellent yield as a single diastereomer. This is a dramatic improvement over the dimethyl sulfate-based procedure (Table 3, entry 5) where the intended product was not observed. These results suggest that the ring-opening reaction is facilitated by the relief of steric congestion resulting from the presence of the ethyl group in **18**. Also of note, the ethyl quaternized salt is THF soluble whereas its methyl analogue is insoluble. This factor no doubt aided this transformation. Ring opening of **13d** with LiBHEt_3_ provided a 94% yield of **19e**. We were pleased to observe that ring opening with stabilized nucleophiles was also possible for this substrate. Addition of lithiated 1-octyne produced a 36% yield of the desired product. Given this limited success, we also subjected ethyl quaternized salt **18** to ring opening with the lithium enolate of *tert*-butyl acetate and lithiated 2-methylpyridine which provided the ring opened products **19g** and **19h** in moderate yields. Products **19f**–**19h** were accompanied by small amounts of returned **13d** (3–6%).

The least reactive substrate, **13e**, was investigated next. We believe this substrate is challenging for two reasons. The electron deficient nature of the pyridyl ring clearly inhibits ring opening, as is evident in the reactions described in Tables 2–5. The methyl located at the 9 position of the pyridylazepine also clearly retards ring opening. Based on the results observed for **13d**, we anticipated that ethyl quaternization would mitigate the latter problem and might allow access to ring-opened products. Addition of MeMgBr to ethyl quaternized **13e** failed to yield any detectable product. On the other hand, the ring opening of **13e** by LiBHEt_3_ was greatly benefited by the change from methyl to ethyl quaternization. The dimethyl sulfate mediated version (Table 4, entry 6) proceeded in a mere 6% yield whereas the ethyl triflate version provided a 68% yield of the indicated product **19j**. This result further substantiates the theory that the reactivity problem that exists with substrate **13e** lies with the ring opening not the quaternization. Overall, the change from methyl quaternization to ethyl quaternization provided substantial benefit. Yields of ring-opened products, with either RMgX or LiBHEt_3_ increased, and quantities of returned starting material decreased. Ring opening of the more resistant pyridyl-fused substrates **13c** and **13e** with stabilized nucleophiles, however, remains out of reach.

The final task we assigned ourselves was to leverage this reaction sequence for an efficient synthesis of mozavaptan (Scheme 8). Known diallyl aldehyde **20**,^24^ which is obtained in two steps from the commercially available 2-aminobenzyl alcohol, was subjected to the imino-ene reaction with methylamine in a pressure vessel to provide **21** in excellent yield. Activation with dimethyl sulfate followed by ring opening with LiBHEt_3_ under our standard conditions affords benzazepine **22** in 88% yield as a colorless oil. Removal of the allyl group using a modified version of Guibé’s procedure^53^ and subsequent amidation with known acid chloride **23** ^54^ provides mozavaptan **1** as a microcrystalline solid. This synthesis requires fewer linear steps and proceeds in higher overall yield than previously reported routes to Mozavaptan.^14,54–56^

**Scheme 8:**
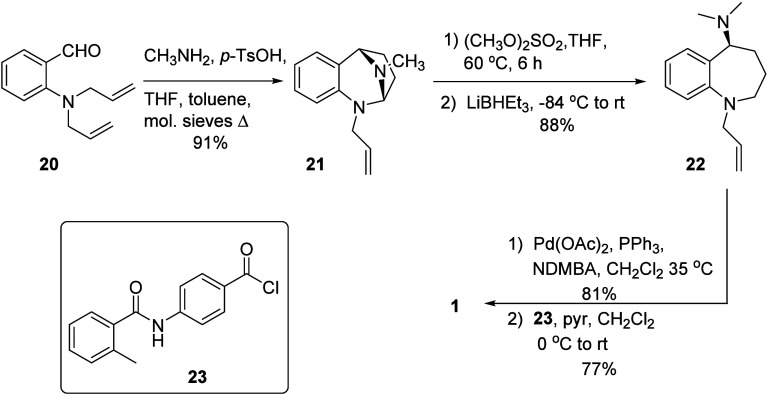
Synthesis of Mozavaptan, **1**

## Conclusion

In summary we have designed and executed a thorough study of the selective ring opening of a variety of aryl-fused *N*,*N*-acetals. The activation of the *N*,*N*-acetal has been shown to be selective, with both dimethyl sulfate and ethyl triflate, favoring reaction at the aliphatic nitrogen atom of the acetal. S_N_2 ring opening, with the activated quaternary aliphatic nitrogen as the leaving group, can be consistently accomplished with Grignard-based nucleophiles and LiBHEt_3_. In cases where the *N*,*N*-acetal substrate does not feature an electron withdrawing pyridyl moiety, ring opening can also be effected with more stabilized nucleophiles such as acetylides, enolates, and 2-methylpyridyl anions. These reactions are stereospecific with respect to the ring opening, affording *trans*-2,5-disubstituted arylazepine products.

## Experimental Section

### General Remarks

*tert*-Butyl acetate, CH_2_Cl_2_, DMF, 2-methylpyridine, 1-octyne, and toluene were distilled from CaH_2_ prior to use. 1-Pentanol was dried over 3Å molecular sieves. Tetrahydrofuran was distilled from Na/benzophenone. *n*-BuLi, *N*-butylamine, diallylamine, NDMBA, dimethyl sulfate (**Caution!** Dimethyl sulfate is a GHS Catergory 1B compound with respect to carcinogenicity and constitutes significant safety hazards and must be handled with extreme care), DPPB, ethyl triflate (**Caution!** Ethyl triflate is a GHS Catergory 1B compound with respect to eye damage and constitutes significant safety hazards and must be handled with extreme care), Grignard reagents, LiBHEt_3_, methylamine as a solution in THF, MeLi, Pd­(OAc)_2_, Pd­(dba)_2_, potassium *t*-butoxide as a solution in THF, and *p*-TsOH were purchased and used as received. Compound **5**,^24^ *N*,*N*-dipropylacetamide,^57^ and acetophenone dimethylhydrazone^41^ were prepared via published procedures.

All reactions were performed under nitrogen or argon atmosphere in oven-dried glassware. Reagent transfer was accomplished using gastight syringes. The heating of reaction mixtures was done with a digital hot plate stirrer. Column chromatography was accomplished using either silica gel (70–230 mesh) or basic alumina (40–300 μm, activity level III) as the stationary phase and mixtures of hexanes and ethyl acetate as the mobile phase. Thin-layer chromatography was performed using silica gel plates with fluorescent indicator. Visualization was accomplished by UV light (254 nm) or iodine.

NMR spectra were recorded using a JEOL spectrometer (400 MHz for ^1^H and 100 MHz for ^13^C), at room temperature, in CDCl_3_. Chemical shifts are reported in δ parts per million referenced to the residual solvent proton resonance of CDCl_3_ (7.28 ppm) or the solvent carbon resonance of CDCl_3_ (77.0 ppm). High-resolution mass spectra (ESI) were acquired on an Agilent QToF mass spectrometer. Positive ion mode was employed in all cases.

### X-ray Analysis

A single colorless, block-shaped crystal (0.30 × 0.35 × 0.40 mm) of complex **11** compound **12** grown from slow evaporation of an ethyl acetate/hexanes solution was mounted on a goniometer using a low-background loop and paratone oil. Data were collected at 114(2) K using an Oxford Cryostream 1000 low temperature device on a Bruker D8 QUEST ECO Fixed CHI diffractometer using Cu Kα (λ = 1.5418 Å; sealed tube) radiation, a flat graphite monochromator and a Bruker PHOTON II 7 CPAD detector. Data were collected over θ = 3.99–68.46° and integrated with SAINT V8.40B yielding 34316 reflections of which 3623 where independent (*R*_int_ = 0.0475) and 90.1% were greater than 2σ­(*F* ^2^).^58^ A Multi-Scan absorption correction using SADABS 2016/2 was applied.^59^ The systematic absences in the diffraction data were consistent with the centrosymmetric, monoclinic space group, *P*2­(1)/c. The structure was solved using intrinsic phasing methods with SHELXT 2018/2 and refined by full-matrix least-squares methods against *F* ^2^ using SHELXL-2019/2.^60,61^ The molecule is located on a general position yielding *Z* = 4, and *Z*′ = 1. All non-hydrogen atoms were refined with anisotropic displacement parameters. All hydrogen atoms were refined isotropic on calculated positions using a riding model with their *U*_iso_ values constrained to 1.5 times the *U*_eq_ of their pivot atoms for terminal sp^3^ carbon atoms and 1.2 times for all other carbon atoms. The goodness of fit on *F* ^2^ was 1.041 with *R*1­(*wR*2) 0.0478(0.1112) for [*I*θ > 2­(*I*)] and with largest difference peak and hole of 0.20 and −0.30 e/Å^3^. The final Crystallographic data for the structure reported in this paper have been deposited with the Cambridge Crystallographic Data Centre.^62^ CCDC 2452689 contain the supplementary crystallographic data for this paper. These data can be obtained free of charge from The Cambridge Crystallographic Data Centre via www.ccdc.cam.ac.uk/structures. The CIF file was generated using FinalCif.^63^

#### 1,10-Dimethyl-2,3,4,5-tetrahydro-1*H*-2,5-epiaminobenzo­[*b*]­azepine (**6a**)

Aldehyde **5** (300 mg, 1.71 mmol), toluene (2.6 mL), *p*-TsOH (16 mg, 0.086 mmol), and MeNH_2_ (2.6 mL of a 2.0 M solution in THF, 5.2 mmol) were refluxed for 2 h. A still-head was fitted onto the reaction vessel and 2.6 mL was distilled to azeotrope off water. The volume was replaced by toluene (1.3 mL) and MeNH_2_ (1.3 mL, 2.0 M solution in THF). This was refluxed for an additional 2 h then 2.6 mL was again removed by distillation. After cooling, NaHCO_3_ (1.0 g) was added and the mixture was stirred for 5 min. The mixture was vacuum filtered. This mixture was partitioned between Et_2_O (10 mL) and water (5 mL). The aqueous layer was extracted with Et_2_O (2 × 5 mL). The combined organic layers were concentrated under reduced pressure. The residue was purified by column chromatography (SiO_2_, gradient ranging from hexanes to 10% EtOAc in hexanes) to provide 183 mg (57%) of the title compound **6a** as a light-yellow oil. ^1^H NMR (400 MHz, CDCl_3_): 7.15–7.09 (m, 1H), 6.87 (dd, *J* = 7.1, 1.1 Hz, 1H), 6.73–6.58 (m, 1H), 6.50 (d, *J* = 8.2 Hz, 1H), 4.10 (d, *J* = 4.6 Hz, 1H), 3.75, (d, *J* = 6.4 Hz, 1H), 2.88 (s, 3H), 2.40 (s, 3H), 2.34–2.22, (m, 1H), 2.13–1.93 (m, 3H). ^13^C­{^1^H} NMR (100 MHz, CDCl_3_): 143.3, 127.8, 126.4, 124.4, 116.1, 109.6, 79.6, 67.8, 36.3, 35.7, 34.1, 32.8. HRMS (ESI-TOF) *m*/*z*: [M + H]^+^ calcd for C_12_H_17_N_2_, 189.1386; found: 189.1386.

#### 1-Methyl-10-butyl-2,3,4,5-tetrahydro-1*H*-2,5-epiaminobenzo­[*b*]­azepine (**6b**)

Aldehyde **5** (1.50 g, 8.60 mmol), toluene (65 mL), *p*-TsOH (82 mg, 0.43 mmol) and BuNH_2_ (1.10 mL, 11.2 mmol) were refluxed for 2 h. A still-head was fitted onto the reaction vessel and 25 mL of the volume was distilled to azeotrope off water. The volume was replaced by toluene (24 mL) and BuNH_2_ (1.0 mL). This mixture was refluxed for an additional 2 h then 25 mL of the volume was again removed by distillation. After cooling, NaHCO_3_ (3.0 g) was added and the mixture was stirred for 5 min. The mixture was vacuum filtered. Water was added and the mixture was partitioned. The aqueous layer was further extracted with Et_2_O (2 × 15 mL). The combined organic layers were concentrated under reduced pressure. The residue was purified by column chromatography (SiO_2_, gradient ranging from 1% to 10% EtOAc in hexanes) to provide 1.38 g (70%) of the title compound **6b** as a light-yellow oil. ^1^H NMR (400 MHz, CDCl_3_): 7.11 (m, 1H), 6.86 (dd, *J* = 7.1, 1.1 Hz, 1H), 6.60 (t, *J* = 7.3 Hz, 1H), 6.48 (d, *J* = 8.2 Hz, 1H), 4.20 (d, *J* = 4.6 Hz, 1H), 3.84 (d, *J* = 6.4 Hz, 1H), 2.87 (s, 3H), 2.60, (m, 1H), 2.49 (m, 1H), 2.25 (m, 1H), 2.06 (m, 2H), 1.94 (m, 1H), 1.54 (m, 2H), 1.32 (td, *J* = 4.9, 7.5 Hz, 2H), 0.91 (t, *J* = 7.3 Hz, 3H). ^13^C­{^1^H} NMR (100 MHz, CDCl_3_): 143.9, 127.7, 126.2, 124.9, 115.9, 109.5, 77.7, 62.0, 46.6, 35.8, 35.6, 32.3, 30.9, 20.8, 14.1. HRMS (ESI-TOF) *m*/*z*: [M + H]^+^ calcd for C_15_H_23_N_2_, 231.1856; found: 231.1855.

#### 1,10,10-Trimethyl-2,3,4,5-tetrahydro-1*H*-2,5-epiaminobenzo­[*b*]­azepinium methyl sulfate (**7a**)

To a mixture of acetal **6a** (400 mg, 0.21 mmol) in THF (0.42 mL, 0.50 M), was added dimethyl sulfate (24 μL, 0.26 mmol). The mixture was heated to 60 °C in a closed vessel for 2 h. After cooling to rt, the volatiles removed under reduced pressure. Hexane (0.5 mL) was added, the mixture stirred, and the liquid was removed. This washing was repeated. The remaining solvent was removed under reduced pressure to afford 61 mg (92%) of an off-white solid, mp 124–126 °C. ^13^C­{^1^H } NMR (100 MHz, CDCl_3_): 139.5, 130.3, 126.9, 121.8, 119.9, 112.8, 88.3, 73.5, 54,4, 46.9, 44.0, 37.8, 35.4, 30.1. ^1^ HRMS (ESI-TOF) *m*/*z*: [M]^+^ calcd for C_13_H_19_N_2_, 203.1548; found: 203.1541.^1^H NMR (400 MHz, CDCl_3_): 7.33–7.28 (m, 1H), 7.09 (d, *J* = 7.3 Hz, 1H), 6.88–6.82 (m, 1H), 6.72 (d, *J* = 8.2 Hz, 1H), 5.63 (d, *J* = 5.5 Hz, 1H), 4.87 (d, *J* = 5.0 Hz, 1H), 3.77 (s, 3H), 3.48 (s, 3H), 3.23 (s, 3H), 3.21 (s, 3H), 2.82–2.62 (m, 2H), 2.42–2.28 (m, 2H).

#### 1,10-Dimethyl-10-butyl-2,3,4,5-tetrahydro-1*H*-2,5-aminobenzo­[*b*]­azepinium methyl sulfate (**7b**)

To a mixture of acetal **6b** (67 mg, 0.29 mmol) in THF (0.58 mL, 0.50 M), was added dimethyl sulfate (33 μL, 0.26 mmol). The mixture was heated to 60 °C in a closed vessel for 2 h. After cooling to rt, the volatiles removed under reduced pressure. Hexane (0.5 mL) was added, the mixture stirred, and the liquid was removed. This washing was repeated. The remaining solvent was removed under reduced pressure to afford 90 mg (87%) of an off-white solid ^1^H NMR (400 MHz, CDCl_3_): Major diastereomer: 7.34–7.28 (m, 1H), 7.05–7.03 (m, 1H), 6.88–6.82 (m, 1H), 6.71–6.68 (m, 1H), 5.71 (d, *J* = 4.6 Hz, 1H), 4.69 (d, *J* = 4.6 Hz, 1H), 3.97–3.88 (m, 1H), 3.76 (s, 3H), 3.53–3.45 (m, 1H), 3.37 (s, 3H), 3.15 (s, 3H), 2.76–2.65 (m, 2H), 2.45–2.27 (m, 2H), 1.95–1.77 (m, 1H), 1.72–1.62 (m, 1H), 1.33–1.15 (m, 1H), 1.28–1.20 (m, 1H), 1.05 (t, *J* = 7.3 Hz, 3H). Minor diastereomer: 7.34–7.28 (m, 1H), 7.05–7.03 (m, 1H), 6.88–6.82 (m, 1H), 6.75–6.72 (m, 1H), 5.76 (d, *J* = 6.4 Hz, 1H), 4.54 (d, *J* = 5.5 Hz, 1H), 3.76 (s, 3H), 3.37–3.28 (m, 1H), 3.24 (s, 3H), 3.23–3.16 (m, 1H), 3.12 (s, 3H), 2.76–2.65 (m, 2H), 2.58–2.48 (m, 1H), 2.39–2.27 (m, 1H), 1.95–1.77 (m, 1H), 1.72–1.62 (m, 1H), 1.60–1.52 (m, 1H), 1.33–1.15 (m, 1H), 1.05 (t, *J* = 7.3 Hz, 3H). ^13^C­{^1^H} NMR (100 MHz, CDCl_3_): Major diastereomer: 139.8, 130.5, 126.6, 121.2, 120.0, 112.6, 87.9, 71.8, 55.7, 43.9, 37.4, 35.0, 29.8, 25.8, 19.9, 13.4; Minor diastereomer: 140.2, 130.7, 126.9, 120.9, 120.0, 113.2, 89.0, 70.2, 57.4, 40.4, 37.8, 35.4, 30.3, 24.9, 19.8, 13.7. Methyl sulfate anion: 54.5. HRMS (ESI-TOF) *m*/*z*: [M]^+^ calcd for C_16_H_25_N_2_, 245.2012; found: 245.2012.

### General Procedure for the Activation/Ring Opening of **6b** with Ph-M

*N*,*N*-Acetal **6b** (72 mg, 0.31 mmol) is placed in a screw-capped vial, evacuated for 10 min, then backfilled with Ar. THF (0.62 mL, 0.50 M) and dimethyl sulfate (36 μL, 0.37 mmol, 1.2 equiv) were added, and the mixture was heated to 60 °C for 2 h. The mixture was cooled to −84 °C and the selected phenyl nucleophile (0.47 mmol, 1.5 equiv) was added as a solution in either THF, Et_2_O, or Bu_2_O The mixture was allowed to warm to rt, and stirred overnight. A 10% aqueous solution of K_2_CO_3_ (0.5 mL) was added along with Et_2_O (0.5 mL). The mixture was partitioned, and the aqueous phase is further extracted with Et_2_O (2 × 0.5 mL). The combined organic layers were concentrated under reduced pressure and purified by column chromatography (basic Al_2_O_3_, gradient ranging from hexanes to 2% EtOAc in hexanes).

#### *trans*-*N*-Butyl-*N*,1-dimethyl-2-phenyl-2,3,4,5-tetrahydro-1*H*-benzo­[*b*]­azepin-5-amine (**8**)

The product **8** was isolated (70 mg, 72%) as a pale-yellow colored oil. ^1^H NMR (400 MHz, CDCl_3_): 7.44 (d, *J* = 7.3 Hz, 2H), 7.41–7.35 (m, 3H), 7.32–7.22 (m, 2H), 7.10–7.06 (m, 1H), 6.98 (d, *J* = 7.8 Hz, 1H), 4.03 (dd, *J* = 10.4, 7.6 Hz, 1H), 3.91–3.86 (m, 1H), 2.73 (s, 3H), 2.66–2–59 (m, 1H), 2.25 (s, 3H), 2.25–2.18 (m, 1H), 1.81–1.60 (m, 3H), 1.58–1–38 (m, 3H), 1.33–1.23 (m, 2H), 0.91 (t, *J* = 7.3 Hz, 3H). ^13^C­{^1^H} NMR (100 MHz, CDCl_3_): 148.1, 142,4, 134.4, 128.1, 127.5, 127.2, 126.8, 126.6, 121.8, 118.3, 67.5, 63.1, 55.0, 39.6, 39.4, 31.3, 29.3, 24.4, 20.7, 14.1. HRMS (ESI-TOF) *m*/*z*: [M + H]^+^ calcd for C_22_H_31_N_2_, 323.2482; found: 323.2480.

### General Procedure for the Activation/Ring Opening of **6b** with Various Nucleophiles

*N*,*N*-Acetal **6b** (72 mg, 0.31 mmol) was placed in a screw-capped vial, evacuated for 10 min, then backfilled with Ar. THF (0.62 mL, 0.50 M) and dimethyl sulfate (36 μL, 0.37 mmol, 1.2 equiv) were added, and the mixture was heated to 60 °C for 2 h. The mixture was cooled to −84 °C and the selected nucleophile was added as a solution in either THF or Et_2_O (0.47 mmol, 1.5 equiv). The mixture was allowed to warm to rt, and stirred overnight. A 10% aqueous solution of K_2_CO_3_ (0.5 mL) was added along with Et_2_O (0.5 mL). The mixture was partitioned, and the aqueous phase was further extracted with Et_2_O (2 × 0.5 mL). The combined organic layers were concentrated under reduced pressure.

#### *trans*-*N*-Butyl-N,1,2-trimethyl-2,3,4,5-tetrahydro-1*H*-benzo­[*b*]­azepine-5-amine (**9a**)

The residue was purified by column chromatography (basic Al_2_O_3_, gradient ranging from hexanes to 2% EtOAc in hexanes) to provide 63 mg (78%) of **9a** as a colorless oil. ^1^H NMR (400 MHz, CDCl_3_): 7.30 (d, *J* = 7.8 Hz, 1H), 7.23–7.17 (m, 1H), 7.02–6.95 (m, 2H), 3.74–3.68 (m, 1H), 2.94–2.84 (m, 1H), 2.82 (s, 3H), 2.66–2.58 (m, 1H), 2.27 (s, 3H), 2.24–2.15 (m, 1H), 2.12–2.03 (m, 1H), 1.93–1.86 (m, 1H), 1.58–1.41 (m, 4H), 1.33–1.20 (m, 2H), 1.16 (d, *J* = 6.4 Hz, 3H), 0.91 (t, *J* = 7.3 Hz, 3H). ^13^C­{^1^H} NMR (100 MHz, CDCl_3_): 148.4, 134.5, 127.9, 126.6, 121.3, 118.3, 63.9, 58.0, 54.8, 39.2, 39.0, 29.8, 29.7, 24.4, 20.7, 16.5, 14.1. HRMS (ESI-TOF) *m*/*z*: [M + H]^+^ calcd for C_17_H_29_N_2_, 261.2325; found: 261.2321.

#### *trans*-*N*,1-Dibutyl-*N*-methyl-2,3,4,5-tetrahydro-1*H*-benzo­[*b*]­azepine-5-amine (**9b**)

The residue was purified by column chromatography (basic Al_2_O_3_, gradient ranging from hexanes to 2% EtOAc in hexanes) to provide 80 mg (85%) **9b** as a colorless oil. ^1^H NMR (400 MHz, CDCl_3_): 7.30 (d, *J* = 7.3 Hz, 1H), 7.22–7.16 (m,1H), 7.01–6.93 (m, 2H), 3.67 (m, 1H), 2.85 (s, 3H), 2.74–2.65 (m, 1H), 2.64–2.55 (m, 1H), 2.27 (s, 3H), 2.24–2.15 (m, 1H), 2.07–1.95 (m, 1H), 1.83–1.74 (m, 1H), 1.55–1.25 (m, 11H), 1.23–1.15 (m, 1H), 0.96–0.86 (m, 6H). ^13^C­{^1^H} NMR (100 MHz, CDCl_3_): 148.6, 134.3, 127.7, 126.6, 121.1, 118.1, 64.1, 63.0, 54.9, 39.3, 39.2, 30.1, 29.6, 28.9, 26.0, 24.4, 23.0, 20.7, 14.1, 14.1. HRMS (ESI-TOF) *m*/*z*: [M + H]^+^ calcd for C_20_H_35_N_2_, 303.2795; found: 303.2793.

#### *trans*-*N*-Butyl-N,1-dimethyl-2-allyl-2,3,4,5-tetrahydro-1*H*-benzo­[*b*]­azepine-5-amine (**9c**)

The residue was purified by column chromatography (basic Al_2_O_3_, gradient ranging from hexanes to 2% EtOAc in hexanes) to provide 60 mg (68%) **9c** as a light-yellow oil. ^1^H NMR (400 MHz, CDCl_3_): 7.30 (d, *J* = 7.2 Hz, 1H), 7.23–7.17 (m,1H), 7.18–6.94 (m, 2H), 5.83–5.71 (m, 1H), 5.15–5.02 (m, 2H), 3.74–3.65 (m, 1H), 2.87 (s, 3H), 2.86–2.77 (m, 1H), 2.65–2.55 (m, 2H), 2.27 (s, 3H), 2.26–2.10 (m, 1H), 2.10–1.97 (m, 1H), 1.59–1.42 (m, 4H), 1.42–1.20 (m, 4H), 0.91 (t, *J* = 7.3 Hz, 3H). ^13^C­{^1^H} NMR (100 MHz, CDCl_3_): 148.2, 136.2, 134.3, 128.1, 126.8, 121.4, 118.5, 116.5, 64.3, 62.8, 54.8, 39.3, 39.1, 35.3, 29.6, 26.3, 24.3, 20.7, 14.1. HRMS (ESI-TOF) *m*/*z*: [M + H]^+^ calcd for C_19_H_31_N_2_, 287.2482; found: 287.2479.

#### *trans*-*N*-Butyl-*N*,1-dimethyl-2-(4-methoxyphenyl)-2,3,4,5-tetrahydro-1*H*-benzo­[*b*]­azepine-5-amine (**9d**)

The residue was purified by column chromatography (basic Al_2_O_3_, gradient ranging from hexanes to 2% EtOAc in hexanes) to provide 73 mg (66%) **9d** as a colorless oil. ^1^H NMR (400 MHz, CDCl_3_): 7.36 (d, *J* = 7.2 Hz, 1H), 7.32 (d, *J* = 8.4 Hz, 2H), 7.06 (m, 1H), 6.94 (d, *J* = 8.2 Hz, 1H), 6.91 (*J* = 8.2 Hz, 2H), 4.02–3.95 (m, 1H), 3.84 (s, 3H), 3.84–3.80 (m, 1H), 2.70 (s, 3H), 2.66–2.57 (m, 1H), 2.24 (s, 3H), 2.25–2.15 (m, 1H), 1.83–1.69 (m, 2H), 1.68–1.58 (m, 2H), 1–55–1.37 (m, 3H), 1.33–1.22 (m, 2H), 0.90 (t, *J* = 7.3 Hz, 3H). ^13^C­{^1^H} NMR (100 MHz, CDCl_3_): 158.3, 148.1, 134.4, 134.3, 128.5, 127.4, 126.8, 121.8, 118.4, 113.4, 67.0, 63.3, 55.2, 55.0, 39.6, 39.3, 31.4, 29.4, 24.5, 20.7, 14.2. HRMS (ESI-TOF) *m*/*z*: [M + H]^+^ calcd for C_23_H_33_N_2_O, 353.2587; found: 353.2585.

#### *trans*-*N*-Butyl-*N*,1-dimethyl-2-isopropyl-2,3,4,5-tetrahydro-1*H*-benzo­[*b*]­azepine-5-amine (**9e**)

The residue was purified by column chromatography (basic Al_2_O_3_, gradient ranging from hexanes to 2% EtOAc in hexanes) to provide 61 mg (68%) **9e** as a colorless oil. ^1^H NMR (400 MHz, CDCl_3_): 7.19–7.10 (m, 2H), 6.98 (d, *J* = 7.3 Hz, 1H), 6.91–6.84 (m, 1H), 3.72–3.66 (m, 1H), 2.94 (s, 3H), 2.88–2.82 (m, 1H), 2.53–2.45 (m, 1H), 2.22–2.14 (m, 1H), 2.15 (s, 3H), 2.08–1.93 (m, 2H), 1.85–1.70 (m, 3H), 1.48–1.36 (m, 2H), 1.32–1.23 (m, 2H), 0.92 (d, *J* = 6.8 Hz, 3H), 0.88 (t, *J* = 7.2 Hz, 3H), 0.72 (d, *J* = 6.9 Hz, 3H). ^13^C­{^1^H} NMR (100 MHz, CDCl_3_): 149.3, 132.9, 131.3, 127.1, 120.8, 120.5, 68.9, 67.0, 53.9, 40.4, 38.6, 29.9, 29.5, 24.9, 23.4, 21.1, 20.7, 18.5, 14.1. HRMS (ESI-TOF) *m*/*z*: [M-N­(Me)­Bu]^+^ calcd for C_19_H_33_N_2_, 289.2638; found: 289.2633.

#### *trans*-*N*-Butyl-*N*,1-dimethyl-2,3,4,5-tetrahydro-1*H*-benzo­[*b*]­azepine-5-amine (**9f**)

The residue was purified by column chromatography (basic Al_2_O_3_, gradient ranging from hexanes to 2% EtOAc in hexanes) to provide 72 mg (94%) **9f** as a colorless oil. ^1^H NMR (400 MHz, CDCl_3_): 7.38 (d, *J* = 7.3 Hz, 1H), 7.23–7.17 (m, 1H), 6.99–6.94 (m, 1H), 6.91 (d, *J* = 7.3 Hz, 1H), 3.79–3.73 (m, 1H), 3.16–3.07 (m, 1H), 2.85 (s, 3H), 2.81–2.73 (m, 1H), 2.58–2.50 (m, 1H), 2.33–2.25 (m, 1H), 2.31 (s, 3H), 2.04–1.94 (m, 1H), 1.67–1.47 (m, 5H), 1.37–1.24 (m, 2H), 0.91 (t, *J* = 7.3 Hz, 3H). ^13^C­{^1^H} NMR (100 MHz, CDCl_3_): 149.5, 134.8, 127.7, 126.8, 120.8, 116.7, 64.6, 55.3, 54.9, 41.7, 39.3, 29.6, 27.4, 24.9, 20.6, 14.1. HRMS (ESI-TOF) *m*/*z*: [M + H]^+^ calcd for C_16_H_27_N_2_, 247.2169; found: 247.2167.

#### *tert*-Butyl *trans*-*N*-Butyl-*N*,1-dimethyl-2,3,4,5-tetrahydro-1*H*-benzo­[*b*]­azepine-5-amino-2-acetate (**9g**)

The residue was purified by column chromatography (basic Al_2_O_3_, gradient ranging from hexanes to 3% EtOAc in hexanes) to provide 83 mg (74%) **9g** as a colorless oil. ^1^H NMR (400 MHz, CDCl_3_): 7.25 (d, *J* = 7.2 Hz, 1H), 7.23–7.18 (m, 1H), 7.03–6.98 (m, 1H), 6.95 (d, *J* = 7.8 Hz, 1H), 3.73–3.64 (m, 1H), 3.33–3.24 (m, 1H), 2.84 (s, 3H), 2.71–2.64 (m, 1H), 2.63–2.54 (m, 1H), 2.22 (s, 3H), 2.24–1.99 (m, 2H), 1.73–1.58 (m, 1H), 1.54–1.40 (m, 2H), 1.47 (s, 9H), 1.34–1.24 (m, 5H), 0.90 (t, *J* = 7.3 Hz, 3H). ^13^C­{^1^H} NMR (100 MHz, CDCl_3_): 172.0, 147.4, 133.9, 129.2, 127.0, 121.9, 119.3, 80.4, 65.0, 60.2, 54.4, 39.2, 38.8, 37.1, 29.7, 28.0. 27.6, 24.3, 20.7, 14.1. HRMS (ESI-TOF) *m*/*z*: [M + H]^+^ calcd for C_22_H_37_N_2_O_2_, 361.2850; found: 361.2848.

#### *N*,*N*-Dipropyl *trans*-*N*-Butyl-*N*,1-dimethyl-2,3,4,5-tetrahydro-1*H*-benzo­[*b*]­azepine-5-amino-2-acetamide (**9h**)

The residue was purified by column chromatography (basic Al_2_O_3_, gradient ranging from 3 to 9% EtOAc in hexanes) to provide 74 mg (62%) **9h** as a light-yellow viscous oil. ^1^H NMR (400 MHz, CDCl_3_): 7.30–7.25 (m, 1H), 7.22–7.17 (m, 1H), 7.02–6.93 (m, 1H), 6.95 (d, *J* = 7.8, 1H), 3.71–3.64 (m, 1H), 3.52–3.43 (m, 1H), 3.36–3.24 (m, 2H), 3.23–3.13 (m, 2H), 2.84 (s, 3H), 2.78–2.67 (m, 1H), 2.62–2.53 (m, 1H), 2.34–2.13 (m, 2H), 2.24 (s, 3H), 2.08–1.95 (m, 1H), 1.94–1.81 (m, 1H), 1.70–1.41 (m, 7H), 1.40–1.18 (m, 3H), (0.96–0.81 (m, 9H). ^13^C­{^1^H} NMR (100 MHz, CDCl_3_): 171.4, 147.8, 134.0, 128.8, 127.0, 121.8, 118.9, 65.0, 60.2, 54.5, 49.8, 47.7, 39.4, 38.9, 34.0, 29.7, 27.7, 24.5, 22.4, 20.9, 20.7, 14.1, 11.4, 11.2. HRMS (ESI-TOF) *m*/*z*: [M + H]^+^ calcd for C_24_H_42_N_3_O, 388.3322; found: 388.3296.

#### *trans*-*N*-Butyl-2-(2,2-dimethylhydrazineyliden3)-2-phenylethyl)-*N*,1-dimethyl-2,3,4,5-tetrahydro-1*H*-benzo­[*b*]­azepin-5-amine (**9i**)

The residue was purified by column chromatography (basic Al_2_O_3_, gradient ranging from 3 to 9% EtOAc in hexanes) to provide 83 mg (66%) of **9i** as a light-yellow viscous oil. ^1^H NMR (400 MHz, CDCl_3_): 7.70–7.66 (m, 2H), 7.41–7.35 (m, 3H), 7.42–7.27 (m,1H), 7.21–7.16 (m,1H), 7.02–6.96 (m, 1H), 6.95 (d, *J* = 8.2 Hz, 1H), 3.74–3.67 (m, 1H), 3.46–3.28 (m, 1H), 3.22–3.14 (m, 1H), 3.05–2.93 (m, 1H), 2.90 (s, 3H), 2.66–2.58 (m, 1H), 2.56 (s, 6H), 2.26 (s, 3H), 2.24–2.13 (m, 2H), 1.75–1.65 (m, 1H), 1.56–1.45 (m, 2H), 1.44–1.08 (m, 4H), 0.91 (t, *J* = 7.3 Hz, 3H). ^13^C­{^1^H} NMR (100 MHz, CDCl_3_): 167.9, 147.9, 138.2, 134.4, 129.2, 128.5, 127.8, 127.0, 126.7, 121.6, 118.4, 65.0, 60.2, 54.5, 49.8, 47.7, 39.4, 38.9, 29.7, 24.5, 22.4, 20.9, 20.7, 14.1. HRMS (ESI-TOF) *m*/*z*: [M + H]^+^ calcd for C_26_H_39_N_4_, 407.3169; found: 407.3169.

#### *trans*-*N*-Butyl-*N*,1-dimethyl-2-(1-octynyl)-2,3,4,5-tetrahydro-1*H*-benzo­[*b*]­azepine-5-amine (**9j**)

The residue was purified by column chromatography (basic Al_2_O_3_, gradient ranging from hexanes to 3% EtOAc in hexanes) to provide 78 mg (71%) of **9j** as a colorless oil. ^1^H NMR (400 MHz, CDCl_3_): 7.29–7.25 (m, 1H), 7.24–7.17 (m, 1H), 7.04–6.98 (m, 1H), 6.95 (d, *J* = 7.8 Hz, 1H), 3.85–3.78 (m, 1H), 3.63–3.57 (m, 1H), 2.89 (s, 3H), 2.67–2.56 (m, 1H), 2.27 (s, 3H), 2.24–2.14 (m, 3H), 1.95–1.75 (b, 1H), 1.66–1.44 (m, 7H), 1.44–1.35 (m, 2H), 1.34–1.23 (m, 6H), 0.94–86 (m, 6H). ^13^C­{^1^H} NMR (100 MHz, CDCl_3_): 147.1, 134.2, 128.4, 126.8, 122.0, 118.6, 83.8, 79.2, 64.3, 55.3, 54.6, 39.7, 39.1, 31.3, 29.4, 29.4, 28.9, 28.5, 25.0, 22.6, 20.7, 18.7, 14.1, 14.1. HRMS (ESI-TOF) *m*/*z*: [M + H]^+^ calcd for C_24_H_39_N_2_, 355.3108; found: 355.3108.

#### *trans*-*N*-Butyl-*N*,1-dimethyl-2-(2-methylpyridyl)-2,3,4,5-tetrahydro-1*H*-benzo­[*b*]­azepine-5-amine (**9k**)

The residue was purified by column chromatography (basic Al_2_O_3_, gradient ranging from hexanes to 6% EtOAc in hexanes) to provide 84 mg (80%) of **9k** as a light-yellow oil. ^1^H NMR (400 MHz, CDCl_3_): 8.56 (d, *J* = 4.6 Hz, 1H), 7.62 (m, 1H), 7.30–7.26 (m, 1H), 7.25–7.18 (m, 1H), 7.14–7.09 (m, 2H), 7.04–6.97 (m, 2H), 3.80–3.73 (m, 1H), 3.39–3.25 (m, 2H), 2.94 (s, 3H), 2.74–2.65 (m, 1H), 2.65–2.55 (m, 1H), 2.26 (s, 3H), 2.26–2.14 (m, 1H), 2.14–2.03 (m, 1H), 2.20–1.94 (m, 1H), 1.67–1.56 (m, 1H), 1.56–1.36 (m, 2H), 1.35–1.24 (m, 2H), 1.21–1.13 (m, 1H), 0.90 (t, *J* = 7.3 Hz, 3H). ^13^C­{^1^H} NMR (100 MHz, CDCl_3_): 160.3, 149.4, 148.0, 136.2, 134.1, 129.0, 127.0, 123.8, 121.7, 121.0, 119.1, 63.7, 65.0, 54.5, 39.5, 39.1, 38.9, 29.8, 26.8, 24.5, 20.7, 14.1. HRMS (ESI-TOF) *m*/*z*: [M + H]^+^ calcd for C_22_H_32_N_3_, 338.2591; found: 338.2591.

#### *trans*-*N*-Butyl-N,1-dimethyl-2-(1-pentyloxy)-2,3,4,5-tetrahydro-1*H*-benzo­[*b*]­azepine-5-amine (**9l**)

The organic extract was dried over Na_2_SO_4_, filtered, and the solvent was removed under reduced pressure. This provided 75 mg (72%) of **9l** as a light-yellow oil. ^1^H NMR (400 MHz, CDCl_3_): 7.36–7.31 (m, 1H), 7.24–7.15 (m,1H), 7.03–6.96 (m, 2H), 4.21–4.17 (m, 1H), 3.82 (dd, *J* = 10.1, 7.8 Hz, 1H), 3.53–3.41 (m, 2H), 2.99 (s, 3H), 2.61–2.53 (m, 1H), 2.26 (s, 3H), 2.25–2.16 (m, 1H), 2.13–2.05 (m, 1H), 1.74–1.62 (m, 3H), 1.56–1.32 (m, 8H), 1.32–1.25 (m, 2H), 0.98–0.84 (m, 6H). ^13^C­{^1^H} NMR (100 MHz, CDCl_3_): 146.4, 134.0, 128.0, 126.7, 121.7, 118.1, 93.2, 67.7, 63.6, 54.7, 39.0, 38.7, 29.9, 29.7, 28.6, 26.4, 24.1, 22.5, 20.7, 14.1, 14.1. HRMS (ESI-TOF) *m*/*z*: [M-OC_5_H_11_]^+^ calcd for C_16_H_25_N_2_, 245.2012; found: 245.2011.

#### *trans*-*N*-Butyl-*N*,1-dimethyl-2-(1-*tert*-butoxy)-2,3,4,5-tetrahydro-1*H*-benzo­[*b*]­azepine-5-amine (**9m**)

The organic extract was dried over Na_2_SO_4_, filtered, and the solvent was removed under reduced pressure. This provided 68 mg (68%) of **9m** as a light-yellow oil. ^1^H NMR (400 MHz, CDCl_3_): 7.31–7.27 (m, 1H), 7.21–7.15 (m, 1H), 7.01–6.94 (m, 2H), 4.48–4.44 (m, 1H), 3.85–3.78 (m, 1H), 2.92 (s, 3H), 2.65–2.57 (m, 1H), 2.52–2.41 (m, 1H), 2.27 (s, 3H), 2.26–2.20 (m, 1H), 2.17–2.08 (m, 1H), 1.61–1.40 (m, 5H), 1.30 (s, 9H), 1.31–1.25 (m, 1H), 0.91 (t, *J* = 7.2 Hz, 3H). ^13^C­{^1^H} NMR (100 MHz, CDCl_3_): 147.0, 133.8, 128.5, 126.6, 121.5, 119.0, 85.9, 72.5, 63.9, 54.5, 39.0, 38.2, 30.5, 29.7, 29.0, 24.5, 20.7, 14.1. HRMS (ESI-TOF) *m*/*z*: [M-OC_4_H_9_]^+^ calcd for C_16_H_25_N_2_, 245.2012; found: 245.2008.

#### *trans*-*N*-Butyl-*N*-methyl-1-allyl-2-methyl-2,3,4,5-tetrahydro-1*H*-benzo­[*b*]­azepine-5-amine (**11**)

*N*,*N*-Acetal **10** (1.39 g, 5.38 mmol) was placed in a Schlenk flask, evacuated for 10 min, then backfilled with Ar. THF (10.8 mL, 0.50 M) and dimethyl sulfate (612 μL, 6.46 mmol) were added, and the mixture was heated to 60 °C for 2 h. The mixture was cooled to −84 °C and CH_3_MgBr (2.7 mL of a 3.0 M solution Et_2_O, 8.1 mmol) was added. The mixture was allowed to warm to rt and stirred overnight. A 10% aqueous solution of K_2_CO_3_ (10 mL) was added along with Et_2_O (10 mL). The mixture was partitioned, and the aqueous phase was further extracted with Et_2_O (2 × 10 mL). The combined organic layers were concentrated under reduced pressure. The residue was purified by column chromatography (basic Al_2_O_3_, gradient ranging from hexanes to 3% EtOAc in hexanes) to provide 1.02 g (66%) of **11** as a light-yellow oil. ^1^H NMR (400 MHz, CDCl_3_): 7.24–7.15 (m, 2H), 7.03–6.96 (m, 1H), 6.91 (d, *J* = 7.8 Hz, 1H), 5.92–5.80 (m, 1H), 5.24 (dd, *J* = 17.4, 1.4 Hz, 1H), 5.14–5.09 (m, 1H), 3.82–3.74 (m, 3H), 3.24–3.17 (m, 1H), 2.68–2.59 (m, 1H), 2.25 (s, 3H), 2.25–2.17 (m, 1H), 2.07–1.98 (m, 1H), 1.84–1.75 (m, 1H), 1.71–1.61 (m, 1H), 1.53–1.45 (m, 2H), 1.37–1.27 (m, 2H), 1.15–1.05 (m, 1H), 0.95 (d, *J* = 6.0 Hz, 3H), 0.91 (t, *J* = 7.6 Hz, 3H). ^13^C­{^1^H} NMR (100 MHz, CDCl_3_): 146.3, 136.6, 134.4, 130.4, 126.7, 121.9, 121.3, 116.6, 66.0, 55.0, 54.0, 53.4, 38.6, 30.4, 30.2, 24.3, 20.7, 14.8, 14.1. HRMS (ESI-TOF) *m*/*z*: [M + H]^+^ calcd for C_19_H_31_N_2_, 287.2482; found: 287.2481.

#### *trans*-*N*-Butyl-*N*-methyl-1-benzoyl-2-methyl-2,3,4,5-tetrahydro-1*H*-benzo­[*b*]­azepine-5-amine (**12**)

A screw-capped vial was charged with bis­(dibenzylideneacetone)­palladium(0) (20.9 mg, 0.0363 mmol) and 1,4-bis­(diphenylphosphino)­butane (15.5 mg, 0.0363 mmol). After evacuating and backfilling the vial with argon, THF (3.6 mL) was added via syringe. The solution was stirred for 10 minutes, then transferred via syringe to a vial containing 2-mercaptobenzoic acid (123 mg, 0.799 mmol) and amine 11 (208 mg, 0.726 mmol) in THF (1.0 mL) under an argon atmosphere. The orange, homogeneous solution was heated to 60 °C overnight. The solution was cooled to room temperature and transferred to a separatory funnel with Et_2_O (4 mL) and extracted with a 10% aqueous solution of HCl (4 x 4 mL). The aqueous extracts were combined, the pH adjusted to 9 with a 10% aqueous solution of NaOH and then extracted with Et_2_O (4 x 5 mL). The combined extracts were dried over Na_2_SO_4_ and concentrated under reduced pressure to yield the crude deallylated product (128 mg, 0.519 mmol, 72%). To the deallylated intermediate (120 mg, 0.488 mmol) was added pyridine (197 μL, 2.44 mmol) and CH_2_Cl_2_ (2.4 mL). The mixture was cooled to 0 °C and benzoyl chloride (115 μL, 0.732 mmol) was added. The mixture was allowed to warm to rt and stirred for 6 h. A saturated aqueous solution of NaHCO_3_ (2 mL) was added along with 5 mL of Et_2_O. The mixture was partitioned, and the aqueous phase was further extracted with Et_2_O (2 × 5 mL). The combined organic layers were concentrated under reduced pressure. The residue was purified by column chromatography (SiO_2_, gradient ranging from 3% to 11% EtOAc in hexanes) to provide 130 mg (78%) of **12** as an off-white solid, mp 79–80 °C. ^1^H NMR (400 MHz, CDCl_3_): 7.61–7.41 (m, 2H), 7.36–7.29 (m, 1H), 7.25–7.01 (m, 4H), 6.99–6.86 (m, 1H), 6.65–6.52 (m, 1H), 5.29–5.15 (b, 1H, rotamer), 4.11–4.00 (b, 1H, rotamer), 3.64–3.56 (b, 1H, rotamer), 3.35–3.26 (b, 1H, rotamer), 2.74–2.50 (m, 1H), 2.47–2.35 (m, 1H), 2.34–2.09 (m, 4H), 2.07–1.83 (m, 1H), 1.67–1.22 (m, 5H), 0.98–0.83 (m, 6H). ^13^C­{^1^H} NMR (100 MHz, CDCl_3_, 2 rotamers): 171.5, 170.8, 138.4, 138.2, 137.8, 137.2, 137.0, 133.0, 132.7, 130.0, 129.0, 128.9, 128.8, 128.7, 128.5, 127.7, 127.3, 126.5, 126.5, 126.3, 125.8, 125.6, 69.7, 68.4, 54.4, 53.3, 50.8, 49.2, 40.4, 38.7, 29.3, 28.9, 27.8, 25.1, 24.4, 20.8, 20.7, 19.0, 16.9, 16.3, 14.1, 14.0. (HRMS (ESI-TOF) *m*/*z*: [M + H]^+^ calcd for C_23_H_31_N_2_O, 351.2431; found: 351.2433.

### General Procedure for the Activation/Ring Opening of **10** and **13a**–**13e** with Various Nucleophiles

*N*,*N*-Acetal **10** or **13a**–**13e** (0.35 mmol) was placed in a screw-capped vial, evacuated for 10 min, then backfilled with Ar. THF (0.70 mL, 0.50 M) and dimethyl sulfate (40 μL, 0.42 mmol, 1.2 equiv) were added, and the mixture was heated to 60 °C from 6 h (**10** and **13a**–**13c**) to 24 h (**13d** and **13e**) as required. The mixture was cooled to −84 °C and the appropriate nucleophile was added (0.57 mmol, 1.5 equiv) was added as a solution in Et_2_O or THF. The mixture was allowed to warm to rt, and stirred overnight. A 10% aqueous solution of K_2_CO_3_ (0.5 mL) was added along with 0.5 mL of Et_2_O. The mixture was partitioned, and the aqueous phase was further extracted with Et_2_O (2 × 0.5 mL). The combined organic layers were concentrated under reduced pressure.

#### *trans*-*N*-Butyl-*N*-methyl-1-allyl-2-phenyl-2,3,4,5-tetrahydro-1*H*-benzo­[*b*]­azepine-5-amine (**14a**)

The residue was purified by column chromatography (basic Al_2_O_3_, gradient ranging from hexanes to 2% EtOAc in hexanes) to provide 99 mg (82%) of **14a** as a colorless oil. ^1^H NMR (400 MHz, CDCl_3_): 7.35–7.15 (m, 7H), 7.07–7.02 (m, 1H), 6.80 (d, *J* = 7.8 Hz, 1H), 5.89–5.78 (m, 1H), 5.18–5.08 (m, 2H), 4.09–3.98 (m, 2H), 3.64–3.58 (m, 2H), 2.68–2.59 (m, 1H), 2.28–2.18 (m, 1H), 2.24 (s, 3H), 1.90–1.79 (m, 1H), 1.78–1.58 (m, 3H), 1.54–1.38 (m, 2H), 1.33–1.24 (m, 2H), 0.89 (*J* = 7.2 Hz, 3H). ^13^C­{^1^H} NMR (100 MHz, CDCl_3_): 146.0, 141.8, 135.9, 134.4, 129.0, 128.0, 127.7, 126.8, 126.7, 122.3, 120.8, 117.0, 65.2, 64.4, 54.4, 53.3, 39.0, 30.9, 29.6, 24.5, 20.7, 14.2. HRMS (ESI-TOF) *m*/*z*: [M + H]^+^ calcd for C_24_H_33_N_2_, 349.2638; found: 349.2637.

#### *trans*-*N*-Butyl-*N*-methyl-1-allyl-2-phenyl-2,3,4,5-tetrahydro-1*H*-naphthazepine-5-amine (**14b**)

The residue was purified by column chromatography (basic Al_2_O_3_, gradient ranging from hexanes to 2% EtOAc in hexanes) to provide 96 mg (69%) of **14b** as a colorless oil. ^1^H NMR (400 MHz, CDCl_3_): 7.86 (s, 1H), 7.83 (d, *J* = 8.4 Hz, 1H), 7.72 (d, *J* = 7.8 Hz, 1H), 7.46–7.34 (m, 6H), 7.33–7.28 (m, 1H), 7.24 (s, 1H), 5.96–5.84 (m, 1H), 5.24–5.17 (m, 1H), 5.15–5.10 (m, 1H), 4.25–4.11 (m, 2H), 3.92–3.84 (m, 1H), 3.80–3.72 (m, 1H), 2.77–2.67 (m, 1H), 2.34–2.22 (m, 1H), 2.31 (s, 3H), 1.94–1.82 (m, 1H), 1.78–1.166 (m, 2H), 1.62–1.45 (m, 3H), 1.37–1.24 (m, 2H), 0.91 (t, *J* = 7.3 Hz, 3H). ^13^C­{^1^H} NMR (100 MHz, CDCl_3_): 145.1, 141.8, 136.2, 135.5, 133.0, 130.0, 128.2, 127.6, 127.5, 126.8, 126.5, 126.4, 125.3, 123.9, 116.9, 66.0, 63.5, 54.9, 53.5, 39.4, 30.9, 29.2, 25.6, 20.7, 14.2. HRMS (ESI-TOF) *m*/*z*: [M + H]^+^ calcd for C_28_H_35_N_2_, 399.2795; found: 399.2791.

#### *trans*-*N*,*N*-Dimethyl-1-allyl-7-methoxy-2-butyl-2,3,4,5-tetrahydro-1*H*-benzo­[*b*]­azepine-5-amine (**14c**)

The residue was purified by column chromatography (basic Al_2_O_3_, gradient ranging from hexanes to 5% EtOAc in hexanes) to provide 82 mg (74%) of **14c** as a pale-yellow oil. ^1^H NMR (400 MHz, CDCl_3_): 6.85 (d, *J* = 8.7 Hz,1H), 6.80–6.76 (b, 1H), 6.72 (dd, *J* = 8.5, 3.0 Hz, 1H), 5.89–5.78 (m, 1H), 5.23–5.15 (m, 1H), 5.11–5.05 (m, 1H), 3.81–3.76 (b, 2H), 3.79 (s, 3H), 3.58–3.46 (b, 1H), 2.94–2.83 (b, 1H), 2.54 (s, 6H), 2.02–1.90 (b, 1H), 1.78–1.57 (b, 3H), 1.38–1.07 (b, 6H), 0.89 (t, *J* = 7.1 Hz, 3H). ^13^C­{^1^H} NMR (100 MHz, CDCl_3_): 154.7, 140.0, 137.0, 135.5, 122.3, 116.3, 115.4, 111.6, 67.7, 59.6, 55.3, 53.7, 42.8, 29.1, 28.7, 26.6, 24.6, 22.9, 14.1. HRMS (ESI-TOF) *m*/*z*: [M + H]^+^ calcd for C_20_H_33_N_2_O, 317.2587; found: 317.2585.

#### *trans*-*N*,8-Dibutyl-*N*-methyl-9-allyl-6,7,8,9-tetrahydro-5*H*-pyrido­[2,3-*b*]­azepine-5-amine (**14d**)

The combined organic layers were concentrated under reduced pressure. The residue was purified by column chromatography (SiO_2_, gradient ranging from hexanes to 3% to 11% EtOAc in hexanes) to provide 60 mg (52%) of **14d** as a colorless oil. ^1^H NMR (400 MHz, CDCl_3_): 8.17–8.13, (m, 1H), 7.57 (dd, *J* = 7.3, 1.4 Hz, 1H), 6.80 (dd, *J* = 7.3, 5.0 Hz, 1H), 6.06–5.94 (m, 1H), 5.22–5.15 (m, 1H), 5.12–5.07 (m, 1H), 4.13–4.07 (m, 2H), 3.57–3.50 (m, 1H), 3.23–3.15 (m, 1H), 2.52–2.43 (m, 1H), 2.26–2.17 (m, 1H), 2.22 (s, 3H), 2.06–1.95 (m, 1H), 1.79–1.57 (m, 4H), 1.53–1.40 (m, 4H), 1.38–1.10 (m, 5H), 0.94–0.86 (m, 6H). ^13^C­{^1^H} NMR (100 MHz, CDCl_3_): 158.5, 145.5, 136.9, 136.4, 127.4, 116.0, 116.0, 64.1, 58.7, 54.4, 51.8, 38.9, 31.2, 29.6, 28.7, 25.8, 23.8, 22.8, 20.6, 14.1, 14.1. HRMS (ESI-TOF) *m*/*z*: [M + H]^+^ calcd for C_21_H_36_N_3_, 330.2904; found: 330.2903.

#### *N*-Butyl-*N*-methyl-1-allyl-2,3,4,5-tetrahydro-1*H*-benzo­[*b*]­azepine-5-amine (**15a**)

The residue was purified by column chromatography (basic Al_2_O_3_, gradient ranging from hexanes to 2% EtOAc in hexanes) to provide 89 mg (93%) of **15a** as a colorless oil. ^1^H NMR (400 MHz, CDCl_3_): 7.40 (d, *J* = 7.2 Hz, 1H), 7.20–7.14 (m, 1H), 7.00–6.94 (m, 1H), 6.89 (d, *J* = 8.2 Hz, 1H), 5.98–5.8 5 (m, 1H), 5.31–5.25 (m, 1H), 5.19–5.14 (m, 1H), 3.89–3.83 (m, 1H), 3.82–3.76 (m, 1H), 3.74–3.67 (m, 1H), 3.15–3.07 (m, 1H), 2.87–2.79 (m, 1H), 2.59–2.49 (m, 1H), 2.37–2.27 (m, 1H), 2.31 (s, 3H), 2.04–1.95 (m, 1H), 1.66–1.47 (m, 5H), 1.38–1.25 (m, 2H), 0.92 (t, *J* = 7.3 Hz, 3H). ^13^C­{^1^H} NMR (100 MHz, CDCl_3_): 149.1, 136.3, 135.1, 128.0, 126.7, 120.9, 118.1, 116.6. 64.7, 56.6, 54.8, 52.1, 39.2, 29.9, 27.3, 25.1, 20.7, 14.2. HRMS (ESI-TOF) *m*/*z*: [M + H]^+^ calcd for C_18_H_29_N_2_, 273.2325; found: 273.2316.

#### *N*-Butyl-*N*-methyl-1-allyl-2,3,4,5-tetrahydro-1*H*-naphthazepine-5-amine (**15b**)

The residue was purified by column chromatography (basic Al_2_O_3_, gradient ranging from hexanes to 2% EtOAc in hexanes) to provide 107 mg (95%) of **15b** as a colorless oil. ^1^H NMR (400 MHz, CDCl_3_): 7.89 (s, 1H), 7.78 (d, *J* = 8.2 Hz, 1H), 7.71 (d, *J* = 7.8 Hz, 1H), 7.42–7.36 (m, 1H), 7.34–7.29 (m, 1H), 7.21 (s, 1H), 6.04–5.93 (m, 1H), 5.37–5.29 (m, 1H), 5.24–5.18 (m, 1H), 4.02 (dd, *J* = 14.5, 5.3 Hz, 1H), 3.98–3.92 (m, 1H), 3.79 (dd, *J* = 14.5, 6.4 Hz, 1H), 3.28–3.18 (m, 1H), 2.88–2.80 (m, 1H), 2.68–2.57 (m, 1H), 2.39 (s, 3H), 2.38–2.29 (m, 1H), 2.14–2.05 (m, 1H), 1.71–1.41 (m, 5H), 1.40–1.27 (m, 2H), 0.93 (t, *J* = 7.2 Hz, 3H). ^13^C­{^1^H} NMR (100 MHz, CDCl_3_): 147.8, 136.7, 136.1, 133.2, 129.5, 127.5, 126.1, 125.9. 125.3, 123.4, 116.8, 114.0, 64.2, 57.0, 55.2, 52.9, 39.4, 29.8, 28.8, 24.6, 20.7, 14.2. HRMS (ESI-TOF) *m*/*z*: [M + H]^+^ calcd for C_22_H_31_N_2_, 323.2489; found: 323.2483.

#### *N*,*N*-Dimethyl-1-allyl-7-methoxy-2,3,4,5-tetrahydro-1*H*-benzo­[*b*]­azepine-5-amine (**15c**)

The combined organic layers were concentrated under reduced pressure. The residue was purified by column chromatography (basic Al_2_O_3_, gradient ranging from 1% to 4% EtOAc in hexanes) to provide 75 mg (83%) of **15c** as a pale-yellow oil. ^1^H NMR (400 MHz, CDCl_3_): 6.98 (d, *J* = 2.6 Hz, 1H), 6.84 (d, *J* = 8.3 Hz, 1H), 6.73 (dd, *J* = 8.3, 2.6 Hz, 1H), 5.96–5.84 (m, 1H), 5.29–5.20 (m, 1H), 5.16–5.11 (m, 1H), 3.85–3.79 (m, 1H), 3.80 (s, 3H), 3.67–3.56 (m, 2H), 3.07–2.97 (m, 1H), 2.73–2.65 (m, 1H), 2.31 (s, 6H), 1.98–1.88 (m, 1H), 1.60 −1.33 (m, 3H). ^13^C­{^1^H} NMR (100 MHz, CDCl_3_): 154.8, 141.8, 136.9, 136.6, 119.5, 116.4, 113.3, 111.6, 66.1, 56.9, 55.4, 52.7, 43.8, 28.2, 24.4. HRMS (ESI-TOF) *m*/*z*: [M + H]^+^ calcd for C_16_H_25_N_2_O, 261.1961; found: 261.1962.

#### *N*-Butyl-*N*-methyl-9-allyl-6,7,8,9-tetrahydro-5*H*-pyrido­[2,3-*b*]­azepine-5-amine (**15d**)

The residue was purified by column chromatography (basic Al_2_O_3_, gradient ranging from 1 to 3% EtOAc in hexanes) to provide 52 mg (54%) of **15d** as a pale-yellow oil. ^1^H NMR (400 MHz, CDCl_3_): 8.05 (dd, *J* = 4.7, 1.4 Hz, 1H), 7.72 (d, *J* = 7.4 Hz, 1H), 6.69 (dd, *J* = 7.4, 4.7 Hz, 1H), 6.04–5.93 (m, 1H), 5.24–5.13 (m, 2H), 4.19 (dd, *J* = 15.2, 5.5 Hz, 1H), 4.06 (dd, *J* = 15.2, 6.2 Hz, 1H), 3.70–3.63 (m, 1H), 3.37–3.21 (m, 2H), 2.44–2.31 (m, 2H), 2.20 (s, 3H), 2.04–1.94 (m, 1H), 1.86–1.66 (m, 3 H), 1.53–1.42 (m 2H), 1.37–1.22 (m, 2H), 0.91 (t, *J* = 7.3 Hz, 3H). ^13^C­{^1^H} NMR (100 MHz, CDCl_3_): 159.8, 145.0. 137.5, 136.1, 125.1, 116.2, 114.2, 63.8, 54.1, 53.6, 49.1, 38.3, 30.0, 25.9, 25.3, 20.5, 14.1. HRMS (ESI-TOF) *m*/*z*: [M + H]^+^ calcd for C_17_H_28_N_3_, 274.2278; found: 274.2279.

#### *N*-Butyl-N,5-dimethyl-1-allyl-2,3,4,5-tetrahydro-1*H*-benzo­[*b*]­azepine-5-amine (**15e**)

The residue was purified by column chromatography (basic Al_2_O_3_, gradient ranging from hexanes to 1% EtOAc in hexanes) to provide 46 mg (46%) of **15e** as a colorless oil. ^1^H NMR (400 MHz, CDCl_3_): 7.75 (dd, *J* = 7.8, 1.8 Hz, 1H), 7.16–7.11 (m, 1H), 6.97–6.86 (m, 1H), 6.88 (d, *J* = 8.2 Hz, 1H), 5.97–5.85 (m, 1H), 5.30–5.23 (m, 1H), 5.19–5.14 (m, 1H), 3.80–3.75 (m, 2H), 3.01–2.95 (m, 2H), 2.35–2.16 (m, 3H), 2.19 (s, 3H), 1.77–1.55 (m, 3H), 1.52–1.32 (m, 2H), 1.45 (s, 3H), 1.31–1.19 (m, 2H), 0.87 (t, *J* = 7.3 Hz, 3H). ^13^C­{^1^H} NMR (100 MHz, CDCl_3_): 150.2, 140.0, 136.6, 129.6, 126.7, 121.1, 118.8, 116.6, 64.1, 56.6, 52.6, 50.7, 34.5, 31.2, 28.3, 28.1, 24.5, 20.6, 14.2. HRMS (ESI-TOF) *m*/*z*: [M-CH_3_)]^+^ calcd for C_18_H_27_N_2_, 271.2169; found: 271.2164.

#### *N*-Butyl-*N*,5-dimethyl-9-allyl-6,7,8,9-tetrahydro-5*H*-pyrido­[2,3-*b*]­azepine-5-amine (**15f**)

The residue was purified by column chromatography (basic Al_2_O_3_, gradient ranging from 1% to 3% EtOAc in hexanes) to provide 6 mg (6%) of **15f** as a pale-yellow oil. ^1^H NMR (400 MHz, CDCl_3_): 8.04–8.01 (m, 1H), 7.98–7.94 (m, 1H), 6.73–6.68 (m, 1H), 6.06–5.95 (m, 1H), 5.26–5.18 (m, 1H), 5.17–5.12 (m, 1H), 4.25 (dd, *J* = 15.0, 5.7 Hz, 1H), 4.00 (dd, *J* = 15.0, 6.4 Hz, 1H), 3.38–3.28 (m, 1H), 3.13–3.05 (m, 1H), 2.24–2.05 (m, 3H), 2.14 (s, 3H), 1.95–1.83 (m, 1H), 1.71–1.62 (m, 1H), 1.43 (s, 3H), 1.42–1.30 (m, 1H), 1.24–1.13 (m, 4H), 0.83 (t, *J* = 7.1 Hz, 3H). ^13^C­{^1^H} NMR (100 MHz, CDCl_3_): 160.7, 144.5, 138.9, 136.5, 130.4, 116.4, 114.6, 62.0, 53.6, 50.6, 49.7, 34.4, 31.2, 27.3, 25.9, 24.7, 20.4, 14.2. HRMS (ESI-TOF) *m*/*z*: [M-N­(Me)­Bu]^+^ calcd for C_13_H_17_N_2_, 201.1386; found: 201.1386.

#### *N*-Butyl-*N*-methyl-1-allyl-2-(1-octynyl)-2,3,4,5-tetrahydro-1*H*-benzo­[*b*]­azepine-5-amine (**16a**)

The residue was purified by column chromatography (basic Al_2_O_3_, gradient ranging from hexanes to 3% EtOAc in hexanes) to provide 62 mg (47%) of **16a** as a colorless oil. ^1^H NMR (400 MHz, CDCl_3_): 7.24–7.16 (m, 2H), 7.07–7.00 (m, 1H), 7.03 (d, *J* = 7.1 Hz, 1H), 5.92–5.78 (m, 1H), 5.35–5.26 (m, 1H), 5.19–5.14 (m, 1H), 3.91–3.78 (m, 4H), 2.71–2.61 (m, 1H), 2.26 (s, 3H), 2.30–2.19 (m, 1H), 2.19–2.14 (m, 2H), 2.13–2.06 (m, 1H), 1.91–1.74 (m, 2H), 1.54–1.41 (m, 4H), 1.41–1.23 (m, 9H), 0.94–0.86 (m, 6H). ^13^C­{^1^H} NMR (100 MHz, CDCl_3_): 146.1, 135.7, 133.8, 130.6, 127.1, 122.7, 121.4, 117.5, 84.4, 77.3, 66.1, 54.2, 53.9, 52.2, 38.6, 31.3, 30.0, 29.5, 29.0, 28.4, 24.6, 22.6, 20.7, 18.6, 14.1, 14.1. HRMS (ESI-TOF) *m*/*z*: [M + H]^+^ calcd for C_26_H_41_N_2_, 381.3264; found: 381.3263.

#### *N*-Butyl-*N*-methyl-1-allyl-2-(1-octynyl)-2,3,4,5-tetrahydro-1*H*-naphthazepine-5-amine (**16b**)

The residue was purified by column chromatography (basic Al_2_O_3_, gradient ranging from hexanes to 2% EtOAc in hexanes) to provide 63 mg (42%) of **16b** as a colorless oil. ^1^H NMR (400 MHz, CDCl_3_): 7.78 (d, *J* = 7.8 Hz, 1H), 7.72 (d, *J* = 8.2 Hz, 1H), 7.70 (s, 1H), 7.43–7.32 (m, 2H), 7.27 (s, 1H), 5.98–5.85 (m, 1H), 5.35 (dd, *J* = 16.9, 1.4 Hz, 1H), 5.23–5.17 (m, 1H), 4.09–3.87 (m, 4H), 2.77–2.64 (m, 1H), 2.33 (s, 3H), 2.32–2.20 (m, 2H), 2.20–2.11 (m, 2H), 1.87–1.71 (m, 2H), 1.58–1.40 (m, 5H), 1.39–1.17 (m, 8H), 0.95–0.83 (m, 6H). ^13^C­{^1^H} NMR (100 MHz, CDCl_3_): 145.1, 135.5, 134.9, 133.2, 130.0, 128.4, 127.4, 126.5, 125.4, 124.0, 117.6, 117.6, 84.5, 78.4, 65.6, 54.5, 54.2, 52.8, 38.9, 31.3, 29.7, 29.3, 28.9, 28.4, 25.8, 22.5, 20.8, 18.6, 14.2, 14.1. HRMS (ESI-TOF) *m*/*z*: [M + H]^+^ calcd for C_30_H_43_N_2_, 431.3421; found: 431.3414

#### *N*,*N*-Dimethyl-1-allyl-7-methoxy-2-(1-octynyl)-2,3,4,5-tetrahydro-1*H*-benzo­[*b*]­azepine-5-amine (**16c**)

The residue was purified by column chromatography (basic Al_2_O_3_, gradient ranging from hexanes to 3% EtOAc in hexanes) to provide 110 mg (77%) of **16c** as a colorless oil. ^1^H NMR (400 MHz, CDCl_3_): 6.88 (d, *J* = 8.7 Hz, 1H), 6.78 (d, *J* = 2.7 Hz, 1H), 6.77–6.72 (m, 1H), 5.89–5.78 (m, 1H), 5.27 (dd, *J* = 17.2, 1.1 Hz, 1H), 5.17–5.11 (m, 1H), 3.80 (s, 3H), 3.80–3.64 (m, 4H), 2.29 (s, 6H), 2.19–2.13 (m, 2H), 2.13–2.05 (m, 1H), 1.88–1.69 (m, 2H), 1.53–1.42 (m, 2H), 1.42–1.24 (m, 7H), 0.91 (t, *J* = 6.9 Hz, 3H). ^13^C­{^1^H} NMR (100 MHz, CDCl_3_): 155.3, 139.2, 135.9, 134.5, 122.3, 117.3, 116.1, 111.9, 84.4, 78.5, 67.7, 55.3, 54.4, 52.3, 42.3, 31.3, 29.5, 29.0, 28.4, 25.0, 22.6, 18.6, 14.1. HRMS (ESI-TOF) *m*/*z*: [M + H]^+^ calcd for C_24_H_37_N_2_O, 369.2900; found: 369.2902.

#### (5S*,8R*,10S*)-9-Allyl-10-butyl-5,10-dimethyl-6,7,8,9-tetrahydro-5*H*-5,8-epipyrido­[2,3-*b*]­azepinium Methyl Sulfate (**17**)

To a mixture of acetal **13e** (75 mg, 0.28 mmol) in THF (0.56 mL, 0.50 M), was added dimethyl sulfate (32 μL, 0.33 mmol). The mixture was heated to 60 °C in a closed vessel for 24 h. After cooling to rt, the volatiles removed under reduced pressure. Hexane (0.5 mL) was added, the mixture stirred, and the liquid was removed. This washing was repeated. The remaining solvent was removed under reduced pressure to afford 86 mg (78%) of **17** as an white solid, mp = 115–116 °C. ^1^H NMR (400 MHz, CDCl_3_): 8.15 (dd, *J* = 4.7, 1.4 Hz, 1H), 7.39 (dd, *J* = 7.6, 1.4 Hz, 1H), 6.75 (dd, *J* = 7.6, 4.7 Hz, 1H), 6.01–5.88 (m, 1H), 5.66 (d, *J* = 5.9 Hz, 1H), 5.45 (d, *J* = 17.4 Hz, 1H), 5.24 (d, *J* = 10.1 Hz, 1H), 4.34 (dd, *J* = 15.1, 6.4 Hz, 1H), 4.21 (dd, *J* = 15.1, 6.8 Hz, 1H), 3.70 (s, 3H), 3.40 (s, 3H), 3.16–3.05 (m, 1H), 2.94–2.76 (m, 2H), 2.69–2.59 (m, 1H), 2.46–2.28 (m, 2H), 2.09–1.95 (m, 1H), 1.88 (s, 3H), 1.85–1.71 (m, 1H), 1.38–1.27 (m, 1H), 1.25–1.16 (m, 1H), 0.87 (t, *J* = 7.3 Hz, 3H). ^13^C­{^1^H} NMR (100 MHz, CDCl_3_): 150.5, 148.7, 132.6, 132.3, 119.8, 119.4, 114.9, 84.0, 79.9. 54.2, 54.2, 49.6, 41.7, 40.7, 30.6, 25.8, 19.9, 15.0, 13.4; Methyl sulfate anion: 54.2. HRMS (ESI-TOF) *m*/*z*: [M]^+^ calcd for C_18_H_28_N_3_, 286.2278; found: 286.2281.

#### 1-Allyl-5-methyl-10-ethyl-10-butyl-2,3,4,5-tetrahydro-1*H*-2,5-aminobenzo­[*b*]­azepinium Methyl Sulfate (**18**)

To a mixture of acetal **13d** (0.35 mmol) in CH_2_Cl_2_ (0.7 mL, 0.50 M), was added 50 μL (0.39 mmol) of EtOTf. The mixture was stirred at rt for 24 h then for 1 h at 30 °C. Hexane (0.5 mL) was added, the mixture stirred, and the liquid was removed. This washing was repeated. The remaining solvent was removed under reduced pressure to afford 133 mg (85%) of **18** as a tan solid. ^1^H NMR (400 MHz, CDCl_3_): Major diastereomer: 7.31–7.23 (m, 1H), 7.10 (dd, *J* = 7.8, 1.4 Hz, 1H), 6.91–6.83 (m, 1H), 6.72 (d, *J* = 8.0 Hz, 1H), 5.97–5.83 (m, 1H), 5.43–5.25 (m, 2H), 5.09 (d, *J* = 5.9 Hz, 1H), 4.17–4.09 (m, 1H), 3.97 (d, *J* = 5.5 Hz, 1H), 3.18–3.03 (m, 1H), 2.93 (td, *J* = 12.3, 4.6 Hz, 1H), 2.74 (td, *J* = 12.3, 5.0 Hz, 1H), 2.69–2.56 (m, 1H), 2.56–2.41 (m, 2H), 2.26–2.18 (m, 1H), 2.06–1.93 (m, 1H), 1.87 (s, 3H), 1.84–1.66 (m, 2H), 1.60 (t, *J* = 7.1 Hz, 3H), 1.45–1.21 (m, 2H), 0.98–0.92 (m, 3H). Minor diastereomer: 7.31–7.23 (m, 1H), 7.15 (d, *J* = 7.3, 1H), 6.91–6.83 (m, 1H), 6.75 (d, *J* = 8.4 Hz, 1H), 5.97–5.83 (m, 1H), 5.43–5.25 (m, 3H), 4.17–4.09 (m, 1H), 3.84–3.77 (m, 1H), 3.72–3.65 (m, 1H), 3.59–3.47 (m, 1H), 3.39–3.25 (m, 1H), 2.69–2.56 (m, 2H), 2.56–2.41 (m, 1H), 1.92 (s, 3H), 1.84–1.66 (m, 1H), 1.57–1.48 (m,1H), 1.45–1.21 (m, 4H), 1.07 (t, *J* = 7.3 Hz, 3H), 0.91–0.86 (m, 3H).^13^C­{^1^H} NMR (100 MHz, CDCl_3_): Major diastereomer: 139.5, 132.2, 130.3, 125.0, 124.3, 119.9, 118.6, 112.5, 86.7, 81.5, 53.3, 52.7, 49.2, 43.9, 42.5, 30.3, 26.5, 20.3, 13.6, 10.9. Minor diastereomer: 140.2, 132.7, 129.6, 124.5, 119.1, 118.0, 112.7, 111.9, 87.0, 81.3, 50.8, 46.4, 42.0, 28.0, 27.0, 20.2, 19.2, 16.9, 13.4, 10.6. Triflate:120.5 (q, *J* = 310 Hz). HRMS (ESI-TOF) *m*/*z*: [M]^+^ calcd for C_20_H_31_N_2_, 299.2482; found: 299.2482.

### General Procedure for the Quaternization and Ring Opening of **13c**–**13e** with EtOTf

*N*,*N*-Acetal **13c**–**13e** (0.35 mmol) was placed in a screw-capped vial, evacuated for 10 min, then backfilled with Ar. CH_2_Cl_2_ (0.70 mL, 0.50 M) and EtOTf (50 μL, 0.39 mmol, 1.1 equiv) are added, and the mixture is stirred at rt for 24 h, then heated to 30 °C for 1 h. After cooling to rt, the volatiles were removed under reduced pressure. After the addition of THF (0.4 mL), the mixture is cooled to −84 °C and the appropriate nucleophile (0.52 mmol, 1.5 equiv) as a solution in Et_2_O or THF is added. The mixture is allowed to warm to rt, stirred overnight, then heated to 30 °C for 1h. A 10% aqueous solution of K_2_CO_3_ (0.5 mL) is added along with Et_2_O (0.5 mL). The mixture is partitioned, and the aqueous phase is further extracted with Et_2_O (2 × 0.5 mL). The combined organic layers were concentrated under reduced pressure.

#### *trans*-*N*,8-Dibutyl-*N*-ethyl-9-allyl-6,7,8,9-tetrahydro-5*H*-pyrido­[2,3-*b*]­azepine-5-amine (**19a**)

The residue was purified by column chromatography (SiO_2_, gradient ranging from hexanes to 2% EtOAc in hexanes) to provide 91 mg (76%) of **19a** as a colorless oil. ^1^H NMR (400 MHz, CDCl_3_): 8.16–8.11 (m, 1H), 7.59 (dd, *J* = 7.5, 1.6 Hz, 1H), 6.79 (dd, *J* = 7.3, 4.6 Hz, 1H), 6.05–5.93 (m, 1H), 5.21–5.14 (m, 1H), 5.13–5.07 (m, 1H), 4.17–4.03 (m, 2H), 3.84–3.76 (m, 1H), 3.24–3.15 (m, 1H), 2.70–2.59 (m, 1H), 2.58–2.41 (m, 3H), 2.04–1.92 (m, 1H), 1.81–1.56 (m, 3H), 1.54–1.11 (m, 10H), 1.00 (t, *J* = 7.1 Hz, 3H), 0.94–0.85 (m, 6H). ^13^C­{^1^H} NMR (100 MHz, CDCl_3_):158.4, 145.3, 136.9, 136.2, 128.1, 115.9, 115.9, 60.2, 58.8, 51.9, 49.0, 43.4, 31.6, 29.5, 28.7, 26.1, 23.5, 22.8, 20.6, 14.1, 14.1, 12.1. HRMS (ESI-TOF) *m*/*z*: [M + H]^+^ calcd for C_22_H_38_N_3_, 344.3060; found: 344.3067.

#### *trans*-*N*-Butyl-*N*-ethyl-9-allyl-6,7,8,9-tetrahydro-5*H*-pyrido­[2,3-*b*]­azepine-5-amine (**19b**)

The residue was purified by column chromatography (basic Al_2_O_3_, gradient ranging from hexanes to 2% EtOAc in hexanes) to provide 76 mg (76%) of **19b** as a colorless oil. ^1^H NMR (400 MHz, CDCl_3_): 8.03 (dd, *J* = 4.8, 1.6 Hz, 1H), 7.89 (d, *J* = 7.4 Hz, 1H), 6.70 (dd, *J* = 7.4, 4.8 Hz, 1H), 6.03–5.93 (m, 1H), 5.24–5.16 (m, 1H), 5.16–5.12 (m, 1H), 4.18 (dd, *J* = 15.2, 5.5 Hz, 1H), 4.06 (dd, *J* = 15.2, 6.2 Hz, 1H), 3.97–3.91 (m, 1H), 3.42–3.32 (m, 1H), 3.16–3.07 (m, 1H), 2.57–2.38 (m, 4H), 2.04–1.95 (m, 1H), 1.94–1.85 (m, 1H), 1.81–1.61 (m, 2H), 1.48–1.39 (m, 2H), 1.38–1.23 (m, 2H), 1.02 (t, *J* = 7.1 Hz, 3H), 0.91 (t, *J* = 7.3 Hz, 3H). ^13^C­{^1^H} NMR (100 MHz, CDCl_3_):160.3, 144.7, 137.1, 136.1, 126.1, 116.3, 114.2, 59.6, 53.5, 49.2, 48.8, 43.4, 30.6, 26.9, 24.8, 20.7, 14.1, 13.3. HRMS (ESI-TOF) *m*/*z*: [M + H]^+^ calcd for C_18_H_30_N_3_, 288.2434; found: 288.2435.

#### (2S*,5S*)-*N*-Butyl-*N*-ethyl-1-allyl-2,5-dimethyl-2,3,4,5-tetrahydro-1*H*-benzo­[*b*]­azepine-5-amine (**19d**)

The residue was purified by column chromatography (basic Al_2_O_3_, gradient ranging from hexanes to 1% EtOAc in hexanes) to provide 96 mg (87%) of **19d** as a colorless oil. ^1^H NMR (400 MHz, CDCl_3_): 7.46 (d, *J* = 7.8 Hz, 1H), 7.18–7.10 (m, 1H), 7.03–6.96 (m, 1H), 6.84 (d, *J* = 8.2 Hz, 1H), 5.91–5.76 (m, 1H), 5.31–5.23 (m, 1H), 5.13–5.08 (m, 1H), 3.82–3.68 (m, 2H), 3.33–3.22 (m, 1H), 2.74–2.37 (m, 5H), 1.97–1.84 (m, 1H), 1.64–1.54 (m, 1H), 1.49 (s, 3H), 1.40–1.11 (m, 4H), 1.09–0.96 (m, 1H), 0.90 (t, *J* = 7.1 Hz, 3H), 0.85–0.76 (m, 6H).^13^C­{^1^H} NMR (100 MHz, CDCl_3_): 145.9. 140.3, 137.2, 130.5, 126.6, 123.2, 122.2, 116.6, 65.5, 55.1, 54.9, 50.2, 44.4, 33.9, 32.2, 30.7, 29.7, 20.6, 17.0, 14.1, 13.8. HRMS (ESI-TOF) *m*/*z*: [M + H]^+^ calcd for C_21_H_35_N_2_, 315.2795; found: 315.2781.

#### *trans*-*N*-Butyl-*N*-ethyl-1-allyl-5-methyl-2,3,4,5-tetrahydro-1*H*-benzo­[*b*]­azepine-5-amine (**19e**)

The residue was purified by column chromatography (basic Al_2_O_3_, gradient ranging from hexanes to 3% EtOAc in hexanes) to provide 99 mg (94%) of **19e** as a colorless oil. ^1^H NMR (400 MHz, CDCl_3_): 7.83 (dd, *J* = 8.0, 1.6 Hz, 1H), 7.15–7.10 (m, 1H), 6.97–6.91 (m, 1H), 6.87 (d, *J* = 7.8 Hz, 1H), 5.98–5.87 (m, 1H), 5.31–5.24 (m, 1H), 5.21–5.16 (m, 1H), 3.81–3.75 (m, 2H), 3.16–2.96 (m, 2H), 2.63–2.51 (m, 2H), 2.45–2.35 (m, 1H), 2.31–2.22 (m, 2H), 1.77–1.58 (m, 3H), 1.51–1.39 (m, 2H), 1.46 (s, 3H), 1.32–1.18 (m, 2H), 1.01 (t, *J* = 7.1 Hz, 3H), 0.89 (t, *J* = 7.3 Hz, 3H). ^13^C­{^1^H} NMR (100 MHz, CDCl_3_): 150.3, 140.7, 136.6, 130.0, 126.5, 121.0, 118.4, 116.6, 65.1, 56.5, 52.4, 50.2, 44.8, 34.5, 30.2, 28.7, 24.8, 20.7, 16.9, 14.2. HRMS (ESI-TOF) *m*/*z*: [M-N­(Et)­Bu]^+^ calcd for C_14_H_18_N, 200.1434; found: 200.1435.

#### (2R*,5S*)-*N*-Butyl-*N*-ethyl-1-allyl-5-methyl-2-(1-octynyl)-2,3,4,5-tetrahydro-1*H*-benzo­[*b*]­azepine-5-amine (**19f**)

The residue was purified by column chromatography (basic Al_2_O_3_, gradient ranging from hexanes to 2% EtOAc in hexanes) to provide 51 mg (36%) of **19f** as a colorless viscous oil. ^1^H NMR (400 MHz, CDCl_3_): 7.43 (dd, *J* = 7.8, 0.9 Hz, 1H), 7.18–7.11 (m, 1H), 7.04–6.97 (m, 1H), 6.87 (d, *J* = 7.8 Hz, 1H), 5.86–5.73 (m, 1H), 5.34–5.26 (m, 1H), 5.16–5.10 (m, 1H), 3.91–3.82 (m, 1H), 3.80–3.66 (m, 2H), 2.69–2.49 (m, 3H), 2.48–2.36 (m, 2H), 2.15–2.02 (m, 3H),1.67–1.57 (m, 1H), 1.46 (s, 3H), 1.44–1.09 (m, 13H), 0.92–0.8 5 (m, 6H), 0.78 (*J* = 7.1 Hz, 3H). ^13^C­{^1^H} NMR (100 MHz, CDCl_3_): 146.1, 140.2 136.2, 130.1, 126.8, 123.0, 122.9, 117.6, 84.7, 78.5, 65.3, 55.4, 52.5, 50.1, 44.3, 33.9, 32.3, 31.3, 29.8, 29.7, 29.6, 29.0, 28.4, 22.5, 20.6, 18.6, 16.9, 14.1. HRMS (ESI-TOF) *m*/*z*: [M + H]^+^ calcd for C_28_H_45_N_2_, 409.3577; found: 409.3578.

#### (2R*,5S*)-*tert*-Butyl *N*-Butyl-*N*-ethyl-2-allyl-5-methyl-2,3,4,5-tetrahydro-1*H*-benzo­[*b*]­azepine-5-amino-2-acetate (**19g**)

The residue was purified by column chromatography (basic Al_2_O_3_, gradient ranging from hexanes to 3% EtOAc in hexanes) to provide 39 mg (27%) **19g** as a colorless oil. ^1^H NMR (400 MHz, CDCl_3_): 7.45 (d, *J* = 7.8 Hz, 1H), 7.16–7.10 (m, 1H), 7.02–6.97 (m, 1H), 6.82 (t, *J* = 7.8 Hz, 1H), 5.90–5.76 (m, 1H), 5.33–5.25 (m, 1H), 5.17–5.11 (m, 1H), 3.84–3.68 (m, 2H), 3.67–3.54 (m, 1H), 2.67–2.33 (m, 7H), 2.12–2.01 (m, 2H), 1.83–1.76 (m, 1H), 1.64–1.46 (m, 1H), 1.62 (s, 3H), 1.43 (s, 9H), 1.33–1.05 (m, 3H), 0.88 (t, *J* = 7.1 Hz, 3H), 0.80 (t, *J* = 7.1 Hz, 3H).^13^C­{^1^H} NMR (100 MHz, CDCl_3_): 172.2, 145.7, 140.6, 136.3, 130.6, 126.8, 123.2, 122.8, 117.4, 80.2, 65.3, 57.0, 55.3, 50.2, 44.3, 36.3, 33.8, 31.7, 29.7, 28.2, 28.0, 20.5, 16.9, 14.1. HRMS (ESI-TOF) *m*/*z*: [M + H]^+^ calcd for C_26_H_43_N_2_O_2_, 415.3319; found: 415.3316.

#### (2R*,5S*)-*N*-Butyl-*N*-ethyl-1-allyl-5-methyl-2-(2-methylpyridyl)-2,3,4,5-tetrahydro-1*H*-benzo­[*b*]­azepine-5-amine (**19h**)

The residue was purified by column chromatography (basic Al_2_O_3_, gradient ranging from hexanes to 9% EtOAc in hexanes) to provide 23 mg (17%) of **19h** as a colorless oil. ^1^H NMR (400 MHz, CDCl_3_): 8.53 (d, *J* = 5.0 Hz, 1H), 7.58–7.47 (m, 1H), 7.48 (d, *J* = 7.8 Hz, 1H), 7.21–7.15 (m, 1H), 7.13–7.07 (m, 1H), 7.06–6.98 (m, 2H), 6.92 (d, *J* = 7.8 Hz, 1H), 5.91–5.79 (m, 1H), 5.27–5.19 (m, 1H), 5.15–5.11 (m, 1H), 3.85 (dd, *J* = 14.0, 5.3 Hz, 1H), 3.79–3.71 (m, 1H), 3.71–3.63 (m, 1H), 3.16 (dd, *J* = 12.8, 4.6 Hz, 1H), 2.69–2.29 (m, 6H), 1.78–1.66 (m, 2H), 1.59–1.51 (m, 1H), 1.49 (s, 3H), 1.37–1.29 (m, 1H), 1.25–1.08 (m, 3H), 0.87 (t, *J* = 7.3 Hz, 3H), 0.79 (t, *J* = 7.1 Hz, 3H).^13^C­{^1^H} NMR (100 MHz, CDCl_3_): 160.6, 149.3, 146.1, 140.5, 136.7, 136.0, 130.6, 126.8, 123.7, 123.5, 122.5, 120.9, 117.1, 65.4, 60.4, 55.6, 50.2, 33.4, 38.9, 33.9, 31.8, 29.7, 28.3, 20.5, 16.9, 14.1. HRMS (ESI-TOF) *m*/*z*: [M + H]^+^ calcd for calcd for C_26_H_38_N_3_, 392.3060; found: 392.3090.

#### *N*-Butyl-*N*-ethyl-9-allyl-5-methyl-6,7,8,9-tetrahydro-5*H*-pyrido­[2,3-*b*]­azepine-5-amine (**19j**)

The residue was purified by column chromatography (basic Al_2_O_3_, gradient ranging from hexanes to 3% EtOAc in hexanes) to provide 72 mg (68%) of **19j** as a colorless oil. ^1^H NMR (400 MHz, CDCl_3_): 8.07–8.01 (m, 2H), 6.71 (dd, *J* = 7.3, 4.6 Hz, 1H), 6.06–5.95 (m, 1H), 5.25–5.17 (m, 1H), 5.15–5.12 (m, 1H), 4.26 (dd, *J* = 15.1, 5.9 Hz, 1H), 3.97 (dd, *J* = 15.1, 6.4 Hz, 1H), 3.37–3.28 (m, 1H), 3.11–3.01 (m, 1H), 2.56–2.45 (m, 2H), 2.28–2.17 (m, 2H), 2.16–2.07 (m, 1H), 1.94–1.84 (m, 1H), 1.69–1.57 (m, 2H), 1.44 (s, 3H), 1.43–1.30 (m, 2H), 1.28–1.13 (m, 2H), 0.94 (t, *J* = 7.1 Hz, 3H) 0.85 (t, *J* = 7.3 Hz, 3H).^13^C­{^1^H} NMR (100 MHz, CDCl_3_): 160.7, 144.4, 139.1, 136.5, 131.1, 116.4, 114.7, 63.0, 53.6, 50.4, 49.7, 44.8, 35.5, 27.8, 27.7, 24.8, 20.6, 17.1, 14.2. HRMS (ESI-TOF) *m*/*z*: [M – N­(Et)­Bu]^+^ calcd for C_13_H_17_N_2_, 201.1386; found: 201.1388.

#### 1-Allyl-10,10-dimethyl-2,3,4,5-tetrahydro-1*H*-2,5-epiaminobenzo­[*b*]­azepine (**21**)

Aldehyde **20** (133 mg, 0.667 mmol), toluene (2.7 mL), *p*-TsOH (5.8 mg, 0.034 mmol) and MeNH_2_ (1.0 mL of a 2.0 M solution of THF, 2.0 mmol) was heated to 80 °C for 16 h in a screw-cap pressure tube. After cooling, the mixture was transferred to beaker using diethyl ether (5 mL) and water (5 mL). Solid CaCO_3_ was added until the aqueous layer had pH ≥ 9. This mixture was transferred to a separatory funnel. The ether layer was saved, and the aqueous layer was further extracted with Et_2_O (3 × 5 mL). The combined organic layers were dried over Na_2_SO_4_. The dried ether solution was concentrated under reduced pressure to provide 130 mg (91%) of **21** as a light-yellow oil that was used without further purification. ^1^H NMR (400 MHz, CDCl_3_): 7.12–7.06 (m, 1H), 6.88 (dd, *J* = 7.3, 1.4 Hz, 1H), 6.63–6.57 (m, 1H), 6.48 (d, *J* = 8.2 Hz, 1H), 5.93–5.85 (m, 1H), 5.30 (dd, *J* = 16.9, 1.7 Hz, 1H), 5.17 (dd, *J* = 10.3, 1.7 Hz, 1H), 4.14 (d, *J* = 5.0 Hz, 1H), 3.92–3.83 (m, 1H), 3.83–3.75 (m, 2H), 2.39 (s, 3H), 2.35–2.24 (m, 1H), 2.23–2.13 (m, 1H), 2.10–1.96 (m, 2H). ^13^C­{^1^H} NMR (100 MHz, CDCl_3_) : 142.4, 134.7, 127.8, 126.7, 124.1, 115.9, 115.7, 109.6, 78.5, 63.9, 51.8, 35.9, 34.8, 34.2. HRMS (ESI-TOF) *m*/*z*: [M + H]^+^ calcd for C_14_H_19_N_2_, 215.1543; found: 215.1543.

#### *N*,*N*-Dimethyl-1-allyl-2,3,4,5-tetrahydro-1*H*-benzo­[*b*]­azepine-5-amine (**22**)

*N*,*N*-Acetal **21** (0.110 g, 0.513 mmol) was placed in a screw-capped vial, evacuated for 10 min, then backfilled with Ar. THF (1.0 mL) and dimethyl sulfate (58 μL, 0.62 mmol) were added, and the mixture was heated to 60 °C for 6 h. The mixture was cooled to −84 °C and LiBHEt_3_ (1.0 mL of a 1.0 M solution of in THF, 1.0 mmol) was added. The mixture was allowed to warm to room temperature, with stirring, overnight. A 10% aqueous solution of NaOH (1.0 mL) was added along with Et_2_O (2.0 mL). The mixture was partitioned, and the aqueous phase was further extracted with Et_2_O (2 × 5 mL). The combined organic layers were concentrated under reduced pressure. The residue was purified by column chromatography (basic Al_2_O_3_, gradient ranging from hexanes to 5% EtOAc in hexanes) to provide 104 mg (88%) of **22** as a colorless oil. ^1^H NMR (400 MHz, CDCl_3_): 7.38–7.32 (m, 1H), 7.20–7.14 (m, 1H), 6.98–6.93 (m, 1H), 6.89 (d, *J* = 7.8 Hz, 1H), 5.98–5.87 (m, 1H), 5.26 (dd, *J* = 17.2, 1.6 Hz, 1H), 5.17 (dd, *J* = 10.3, 1.6 Hz, 1H), 3.88 (dd, *J* = 14.7, 5.3 Hz, 1H), 3.71 (dd, *J* = 14.7, 6.4 Hz, 1H), 3.59–3.52 (m, 1H), 3.13–3.06 (m, 1H), 2.93–2.85 (m, 1H), 2.31 (s, 6H), 2.01–1.89 (m, 1H), 1.66–1.46 (m, 3H). ^13^C­{^1^H} NMR (100 MHz, CDCl_3_): 148.8, 136.1, 134.1, 128.2, 126.9, 120.9, 118.2, 116.6, 66.6, 56.6, 52.3, 43.8, 27.9, 24.4. HRMS (ESI-TOF) *m*/*z*: [M-N­(Me)_2_]^+^ calcd for C_13_H_16_N, 186.1283; found: 186.1279.

**Mozavaptan** (**1**). A 10 mL Schlenk flask was charged with tetrahydrobenzazepine **22** (0.159 g, 0.690 mmol), 1,3-dimethylbarbituric acid (0.305 g, 1.95 mmol), palladium acetate (0.006 g, 0.03 mmol), and triphenylphosphine (0.031 g, 0.12 mmol). The septum-capped flask was evacuated for five minutes, then backfilled with argon. The evacuation and backfill cycle was repeated two more times. Dried and degassed methylene chloride (6.5 mL) was added via syringe. The flask was sealed and placed in a 30 °C oil bath for 18 h. Upon cooling, a saturated solution of K_2_CO_3_ (10 mL) was added to the reaction. The mixture was partitioned, and the aqueous phase was further extracted with methylene chloride (3 × 5 mL). The combined organic layers were shaken with brine (2 × 5 mL), then concentrated under reduced pressure. The residue was purified by column chromatography (basic Al_2_O_3_, solvent gradient ranging from 2% to 20% EtOAc in hexanes) to provide 106 mg (81%) of *N*,*N*-dimethyl-2,3,4,5-tetrahydro-1*H*-benzo­[*b*]­azepine-5-amine as a viscous, pale-yellow oil. ^1^H NMR (400 MHz, CDCl_3_): 7.16 (dd, *J* = 7.5, 1.1 Hz 1H), 7.09 (dt, *J* = 7.5, 1.7 Hz, 1H), 6.86–6.82 (m, 1H), 6.73 (d, *J* = 7.8 Hz, 1H), 3.84–3.62 (br s, 1H), 3.39 (m, 1H), 3.13 (br s, 1H), 2.90 (m, 1H), 2.17–2.13 (m, 8H), 1.74–1.63 (m, 2H). ^13^C­{^1^H} NMR (100 MHz, CDCl_3_): 149.5, 133.4, 131.9, 127.6, 120.4, 120.2, 71.8, 48.8, 44.4, 29.1, 25.0. HRMS (ESI-TOF) *m*/*z*: [M-N­(Me)_2_]^+^ calcd for C_10_H_12_N, 146.0964; found: 146.0962. A 25 mL round-bottom flask was charged with *N*,*N*-dimethyl-2,3,4,5-tetrahydro-1*H*-benzo­[*b*]­azepine-5-amine (0.050 g, 0.26 mmol), pyridine (0.11 mL, 1.3 mmol), and methylene chloride (5 mL). After cooling to 0 °C, a solution of acid chloride **23** (0.123 g, 0.516 mmol) in methylene chloride (5 mL) was added via syringe. The reaction was allowed to slowly warm to room temperature and stirred overnight. The reaction mixture was transferred to a separatory funnel and washed with saturated and aqueous NaHCO_3_ (3 × 10 mL) and brine (2 × 5 mL). The organic layer was concentrated under reduced pressure and the residue purified by column chromatography (silica gel, solvent gradient ranging from methylene chloride to 5% methanol in methylene chloride) to yield 87 mg (77%) of mozavaptan, **1** as a white, microcrystalline solid, mp 206.0 – 207.5 °C (lit.^50^ 207–208 °C). Mozavaptan exists as a 3:1 mixture of conformational diastereomers.^64^ Spectral data for the major conformational diastereomer: ^1^H NMR (400 MHz, CDCl_3_): 7.98 (br s, 1H), 7.56 (d, *J* = 7.8 Hz, 1H), 7.46 (m, 2H), 7.42 (d, *J* = 7.3 Hz, 1H), 7.36 (m, 1H), 7.30–7.18 (m, 5H), 6.99 (m, 1H), 6.60 (d, *J* = 7.8 Hz, 1H), 3.94 (m, 1H), 3.56 (dd, *J* = 11.0, 6.0 Hz, 1H), 3.49 (m, 1H), 2.45 (s, 3H), 2.44 (s, 6H), 2.11 (m, 1H), 1.85 (m, 1H), 1.43 (m, 1H), 1.21 (m, 1H). ^13^C­{^1^H} NMR (100 MHz, CDCl_3_): 169.0, 168.0, 139.6, 139.4, 136.6, 131.5, 131.3, 130.4, 130.3, 129.7, 128.6, 127.8, 127.5, 127.1, 126.5, 126.3, 125.8, 118.4, 65.2, 46.5, 44.0, 28.9, 23.1, 19.8. HRMS (ESI-TOF) *m*/*z*: [M + H]^+^ calcd for C_27_H_30_N_3_O_2_: 428.2333; found: 428.2332.

## Supplementary Material





## Data Availability

The data underlying this study are available in the published article and its .

## Notes

General remarks, preparation of starting materials, NMR spectra for synthesized compounds, and single-crystal X-ray data (PDF)
FAIR data, including the primary NMR FID files, for compound(s) **1**, **6**−**22**, and **SI-1**−**SI-5** (ZIP)
