# Supplementary material for: Synthesis of trans-2,5-Disubstituted Tetrahydro-1-benzazepines via Nucleophilic Ring Opening of Selectively Quaternized N,N‑Acetals
Source: J Org Chem. 2026 Jan 23;91(5):1895–911. doi: 10.1021/acs.joc.5c01958 (PMC12888014; doi:10.1021/acs.joc.5c01958)
Supplement: Supplementary file 1 [file jo5c01958_si_069.pdf]

Supporting Information

for

Synthesis of *trans*-2,5 Disubstituted Tetrahydro-1-Benzazepines via Nucleophilic Ring Opening of Selectively Quaternized *N*,*N*-Acetals

Chriss E. McDonald,* Holly D. Bendorf,* William G. Dougherty, Juan M. Martínez, Zoie V. Dodson, Cameron L. Upcraft, Dylan J. McGowan, Lilith M. Taylor, Josue Urbina

Department of Chemistry and Biochemistry, Lycoming College, One College Place, Williamsport, PA 17701-5192

Department of Chemistry, Susquehanna University, Selinsgrove PA 17870.

Corresponding authors

Email addresses: *mcdonald@lycoming.edu*, *bendorf@lycoming.edu*

Table of Contents

1. General Remarks……………………………………………………………S3
2. Procedures for Generation of Nucleophiles……………………..S3
3. Synthesis of Substrates **10, 13a-e**………………………………….S4
4. Figure 1. Comparison of Structure and NMR Signals at C-5 of Benzazepines **8** and **9a-k**………………………………………..S11
5. Figure 2. Correlation of Stereochemistry of **11** and **9a**……….S13
6. Additional X-ray Information for **12**…………………..…………..S14
7. Table 1. Crystal Data and Structure Refinement for **12**……………………………………………………………………………..S15
8. Supplement References………………………………………………..S17
9. NMR Spectra of Synthesized Compounds………………………..S18

General Remarks.

Allyl bromide, 2-amino-3-acetylpyridine, 2-amino-5-methoxybenzoic acid, 3-amino-2-naphthoic acid, 2-aminopyridine-3-carboxylic acid, diisopropylamine, DIAB, $LiAlH_4$, TEMPO and *p*-xylene were purchased and used as received.

Procedures for the Generation of Nucleophiles.

2-Lithio tert-butyl acetate

Following a modification of the procedure of Rathke,[1] a THF solution (1.3 mL) of diisopropylamine (154 μL, 1.10 mmol) was cooled to -84 ˚C. A 2.5 M solution of BuLi in hexanes (0.40 mL, 1.0 mmol) was added, and the mixture was allowed to warm to rt providing a solution of LDA. The mixture was re-cooled to -84 ˚C and *tert*-butyl acetate (147 μL, 1.09 mmol) was added over 5 min. This mixture was stirred for 30 min providing a 0.50 M solution of the title compound.

2-Lithio N,N-dibutylacetamide

A THF solution (1.0 mL) of diisopropylamine (154 μL, 1.10 mmol) was cooled to -84 ˚C. A 2.5 M solution of BuLi in hexanes (0.40 mL, 1.0 mmol) was added, and the mixture was allowed to warm to rt providing a solution of LDA. The mixture was re-cooled to -84 ˚C and a THF solution (0.4 mL) of *N,N*-dibutylacetamide[2] (143 mg, 1.00 mmol) was added over 5 min. This mixture was stirred for 30 min providing a 0.50 M solution of the title compound.

2-Lithio acetophenone dimethylhydrazone

Following the procedure of Clardy,[3] A THF solution (1.0 mL) of diisopropylamine (154 μL, 1.10 mmol) was cooled to -84 ˚C. A 2.5 M solution of BuLi in hexanes (0.40 mL, 1.0 mmol) was added, and the mixture was allowed to warm to rt providing a solution of LDA. The mixture was re-cooled to -84 ˚C and a THF solution (0.4 mL) of *acetophenone dimethylhydrazone* (162 mg, 1.00 mmol) was added over 5 min. This mixture was allowed to warm to rt and stirred for 30 min providing a 0.50 M solution of the title compound.

1-Lithio-1-octyne

Following the procedure of Fang,[4] a THF solution (1.5 mL) of 1-octyne (147 μl, 1.00 mmol) was cooled to -84 ˚C. A 2.5 M solution of BuLi in hexanes (0.40 mL, 1.00 mmol) was added, and the mixture was allowed to warm to rt providing a 0.50 M solution of the title compound.

2-Lithio-2-methylpyridine

Following the procedure of Taber,[5] a THF solution (1.5 mL) of 2-methylpyridine (99 μl, 1.0 mmol) was cooled to -84 ˚C. A 2.5 M solution of BuLi in hexanes (0.40 mL, 1.0 mmol) was added, and the mixture was allowed to warm to rt providing a 0.50 M solution of the title compound.

Lithium 1-pentoxide

A THF solution (1.5 mL) of 1-pentanol (147 μl, 1.00 mmol) was cooled to -84 ˚C. A 2.5 M solution of BuLi in hexanes (0.40 mL, 1.00 mmol) was added, and the mixture was allowed to warm to rt providing a 0.50 M solution of the title compound.

Synthesis of Compounds 10,13a-e.

1-Allyl-10-butyl-2,3,4,5-tetrahydro-1H-2,5-epiaminobenzo[b]azepine (**10**).

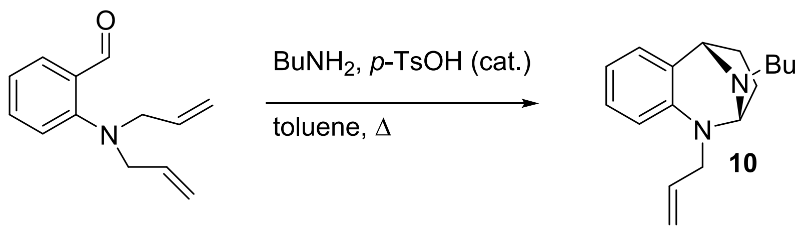

2-*N*,*N*-Diallylaminobenzaldehyde[6] (3.30 g, 16.4 mmol), toluene (85 mL), *p*-TsOH (156 mg, 0.82 mmol), and $BuNH_2$ (3.0 mL, 30.1 mmol), were refluxed for 2 h. A still-head was fitted onto the reaction vessel and 20 mL of the volume was distilled to azeotrope off water. The volume was replaced by toluene (15 mL) and $BuNH_2$ (1.5 mL). This was refluxed for an additional 2 h then 15 mL of the volume was again removed by distillation. After cooling, $NaHCO_3$ (3.0 g) was added and the mixture was stirred for 5 min. Water was added and the mixture was partitioned. The aqueous layer was further extracted with Et_2O (2 x 15 mL). The combined organic layers were concentrated under reduced pressure. The residue was purified by column chromatography (SiO_2, gradient ranging from 1 to 8% EtOAc in hexanes) to provide 3.77 g (89%) of the title compound **10** as a colorless oil. ^1H NMR (400 MHz, $CDCl_3$): 7.09-7.04 (m, 1H), 6.87 (dd, *J* = 7.3, 1.6 Hz, 1H), 6.61-6.55 (m, 1H), 6.46 (d, *J* = 7.8 Hz, 1H), 5.94-5.83 (m, 1H), 5.34-5.26 (m, 1H), 5.19-5.13 (m, 1H), 4.25 (d, *J* = 5.0 Hz, 1H), 3.91-3.73 (m, 3H), 2.62-2.54 (m, 1H), 2.52-2.44 (m, 1H), 2.34-2.22 (m, 1H), 2.21-2.11 (m, 1H), 2.09-2.04 (m, 1H), 2.02-1.94 (m, 1H), 1.61-1.48 (m, 2H), 1.38-1.27 (m, 2H), 0.91 (t, *J* = 7.3 Hz, 3H) . $^{13}C\{1H\}$ NMR (100 MHz, $CDCl_3$): 142.9, 134.8, 127.6, 126.4, 124.6, 115.7, 115.6, 109.5, 76.4, 62.1, 51.6, 46.6, 35.4, 34.3, 30.7, 20.8, 14.1 ppm. HRMS (ESI-TOF) *m/z*: $[M+H]^+$ calcd for $C_{17}H_{25}N_2$, 257.2012; found: 257.2011.

1-Allyl-12-butyl-2,3,4,5-tetrahydro-1H-2,5-epiaminonaphtho[2,3-b]azepine (**13a**)

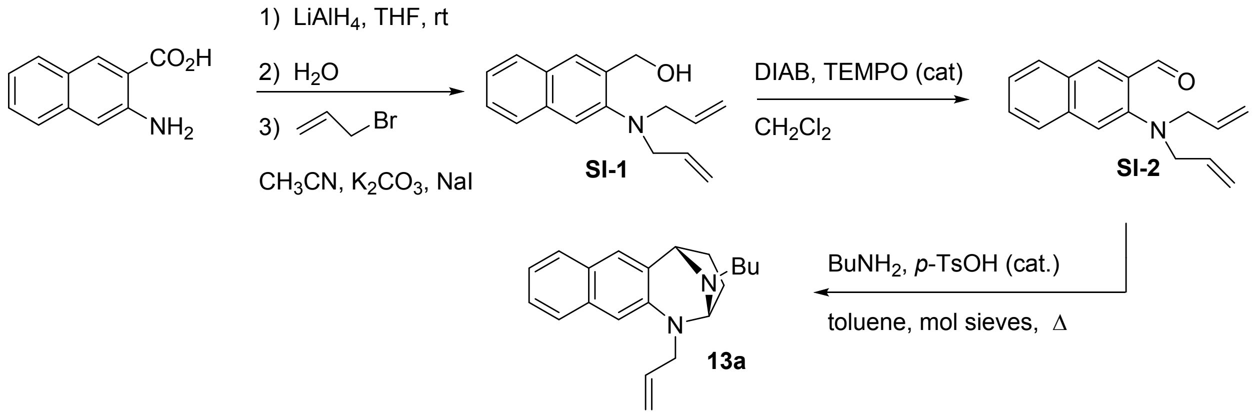

LiAlH$_4$ (0.636 g, 16.7 mmol) was added to ice-cold THF (20 mL). A solution of the carboxylic acid (1.05 g, 5.58 mmol) in THF (30 mL) was added slowly. The mixture was allowed to warm to rt and stirred for 16 h. Water (1 mL), 15% NaOH$_{(aq)}$ (1.5 mL), and water (3 mL) were added sequentially. The mixture was filtered, the solid being rinsed with Et$_2$O. The filtrate was dried over Na$_2$SO$_4$, filtered, and concentrated under reduced pressure to afford the corresponding aminoalcohol as a white solid. The crude alcohol (0.943 g, 5.44 mmol) was dissolved in CH$_3$CN (20 mL). To this was added K$_2$CO$_3$ (3.75 g, 27.2 mmol), NaI (4.08 g, 27.2 mmol), and allyl bromide (2.35 mL, 27.2 mmol). This mixture was stirred overnight at rt. The mixture was diluted with water (50 mL) and extracted with Et$_2$O (3 x 20 mL). The combined organic layers were washed with saturated Na$_2$S$_2$O$_{3(aq)}$ and concentrated under reduced pressure. The residue was purified by column chromatography (SiO$_2$, gradient ranging from 3 to 10 % EtOAc in hexanes) to provide 1.04 g (76%) of **SI-1** as a yellow oil. ^{1}H NMR (400 MHz, CDCl$_3$): 7.81-7.75 (m, 2H), 7.70 (s, 1H), 7.54 (s, 1H), 7.49-7.41 (m, 2H), 5.93-5.81 (m, 2H), 5.29-5.17 (m, 4H), 4.99-4.92 (b, 1H), 4.97 (s, 2H), 3.74 (d, *J* = 6.4 Hz, 4H). ^{13}C{1H} NMR (100 MHz, CDCl$_3$): 147.1, 135.7, 133.7, 133.0, 130.8, 127.6, 127.5, 127.0, 126.0, 125.4, 120.9, 118.8, 64.9, 56.5. HRMS (ESI-TOF) *m/z*: [M+H]$^+$ calcd for C$_{17}$H$_{20}$NO, 254.1539; found: 254.1537. CH$_2$Cl$_2$ (17 mL) was added to alcohol **SI-1** (887 mg, 3.51 mmol). DIAB (1.81 g, 5.62 mmol) and TEMPO (137 mg, 0.878 mmol) were added, and the mixture was stirred at rt overnight. The mixture was diluted with water and partitioned. The aqueous layer was extracted with Et$_2$O (3 x 20 mL). The combined organic layers were washed with saturated Na$_2$S$_2$O$_{3(aq)}$ and concentrated under reduced pressure. The residue was purified by column chromatography (SiO$_2$, gradient ranging from hexanes to 1% EtOAc in hexanes) to provide 661 mg (75%) of **SI-2** as an orange oil. ^{1}H NMR (400 MHz, CDCl$_3$): 10.52 (s, 1H), 8.38 (s, 1H), 7.90 (d, *J* = 8.2 Hz, 1H), 7.75 (d, *J* = 8.2 Hz, 1H), 7.58-7.52 (m, 1H), 7.43-7.38 (m, 2H), 5.94-5.82 (m, 2H), 5.31-5.17 (m, 4H), 3.85 (d, *J* = 6.4 Hz, 4H). ^{13}C{1H} NMR (100 MHz, CDCl$_3$): 192.2, 149.0, 136.5, 133.7, 131.3, 129.7, 129.6, 128.9, 128.8, 126.8, 125.1, 118.2, 118.2, 57.0. HRMS (ESI-TOF) *m/z*: [M+H]$^+$ calcd for C$_{17}$H$_{18}$NO, 252.1383; found: 252.1378.

Aldehyde **SI-2** (562 mg, 2.24 mmol), 5.6 mL of toluene, *p*-TsOH (21 mg, 0.11 mmol), and 1.3 mL of $BuNH_2$ (11 mmol). A still-head was fitted onto the reaction vessel and 3 mL of the volume was distilled to azeotrope off water. The volume was replaced by 2.0 mL of toluene and 1.0 mL of $BuNH_2$. This was refluxed for an additional 2 h then 3 mL of the volume was again removed by distillation. After cooling, 3.0 g of $NaHCO_3$ was added and the mixture was stirred for 5 min. The mixture was vacuum filtered. Water (3 mL) was added to the filtrate was added water was added and the mixture was partitioned. The aqueous layer was further extracted with Et_2O (2 x 5 mL). The combined organic layers were concentrated under reduced pressure. The residue was purified by column chromatography (SiO_2, gradient ranging from 1.5 % to 10% EtOAc in hexanes) to provide 548 mg (80%) of the title compound **13a** as a yellow oil. ^1H NMR (400 MHz, $CDCl_3$): 7.68-7.61 (m, 2H), 7.39-7.33 (m, 1H), 7.36 (s, 1H), 7.24-7.17 (m, 1H), 6.73 (s, 1H), 6.06-5.95 (m, 1H), 5.43-5.37 (m, 1H), 5.29-5.23 (m, 1H), 4.37 (d, *J* = 4.6 Hz, 1H), 4.15 (d, *J* = 6.9 Hz, 1H), 4.05-3.97 (m, 1H), 3.95-3.87 (m, 1H), 2.71-2.62 (m 1H), 2.61-2.53 (m, 1H), 2.48-2.37 (m, 1H), 2.27-2.2-17 (m, 1H), 2.16-2.07 (m, 1H), 2.04-1.97 (m, 1H), 1.67-1.54 (m, 2H), 1.41-1.27 (m, 2H), 0.98-0.91(m, 3H). ^{13}C $^{13}C\{1H\}$ NMR (100 MHz, $CDCl_3$): 141.6, 134.5, 134.1, 128.1, 126.9, 126.6, 125.6, 125.4, 124.6, 121.4, 115.9, 102.7, 76.5, 62.3, 51.6, 46.7, 34.3, 33.9, 30.6, 20.7, 14.0. HRMS (ESI-TOF) *m/z*: $[M+H]^+$ calcd for $C_{21}H_{27}N_2$, 307.0273; found: 307.0275.

1-Allyl-7-methoxy-10-butyl-2,3,4,5-tetrahydro-1H-2,5-epiaminobenzo[b]azepine (**13b**).

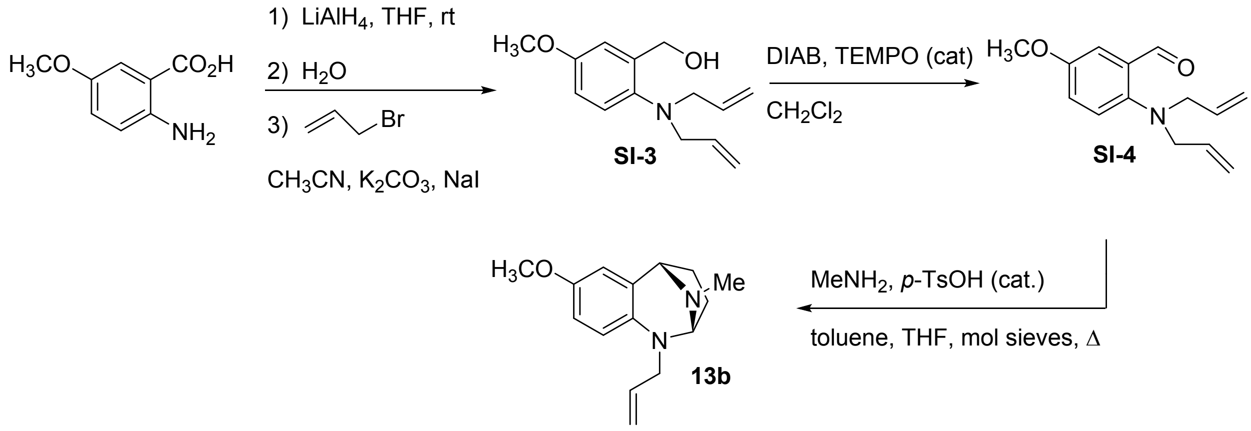

$LiAlH_4$ (0.694 g, 18.3 mmol) was added to ice-cold THF (20 mL). A solution of the carboxylic acid (1.02 g, 6.10 mmol) in THF (30 mL) was added slowly. The mixture was allowed to warm to rt and stirred for 16 h. Water (1 mL), 15% $NaOH_{(aq)}$ (1.5 mL), and water (3 mL) were added sequentially. The mixture was filtered, the solid being rinsed with Et_2O. The filtrate was dried over Na_2SO_4, filtered, and concentrated under reduced pressure to afford **SI-3** as a white solid. The crude alcohol (0.893 g, 5.83 mmol) was dissolved in CH_3CN (20 mL). To this was added K_2CO_3 (4.03 g, 29.1 mmol), NaI (4.37 g, 29.1 mmol), and allyl bromide (2.52 mL, 29.1 mmol). This mixture was stirred overnight at rt. The mixture was diluted with water (50 mL) and extracted with Et_2O (3 x

20 mL). The combined organic layers were washed with saturated $Na_2S_2O_{3(aq)}$ and concentrated under reduced pressure. The residue was purified by column chromatography (SiO_2, gradient ranging from 3 to 15 % EtOAc in hexanes) to provide 809 mg (62.2%) of **SI-3** as a yellow oil. ^{1}H NMR (400 MHz, $CDCl_3$): 7.13 (d, J = 9.1 Hz, 1H), 6.79 (m, 1H), 6.70 (m, 1H), 5.90-5.77 (m, 2H), 5.23-5.14 (m, 4H), 4.78 (s, 2H), 3.79 (s, 3H), 3.57 (d, J = 6.9 Hz, 4H), 1.89-1.57 (b, 1H). ^{13}C{1H} NMR (100 MHz, $CDCl_3$,): 156.8, 141.4, 138.1, 133.8, 124.5, 118.8, 113.2, 112.9, 65.0, 57.5, 55.3. HRMS (ESI-TOF) *m/z*: $[M+H]^+$ calcd for $C_{14}H_{20}NO_2$, 234.1489; found: 234.1487. CH_2Cl_2 (17 mL) was added to alcohol **SI-3** (799 mg, 3.43 mmol). DIAB (1.77 g, 3.49 mmol) and TEMPO (134 mg, 0.858 mmol) were added, and the mixture was stirred at rt overnight. The mixture was diluted with water and partitioned. The aqueous layer was extracted with Et_2O (3 x 15 mL). The combined organic layers were washed with saturated $Na_2S_2O_{3(aq)}$ and concentrated under reduced pressure. The residue was purified by column chromatography (Al_2O_3, gradient ranging from hexanes to 15 % EtOAc in hexanes) to provide 642 mg (80%) of **SI-4** as a yellow oil. ^{1}H NMR (400 MHz, $CDCl_3$): 10.49 (s, 1H), 7.32 (d, J = 2.7 Hz, 1H), 7.18-7.08 (m, 2H), 5.89-5.74 (m, 2H), 5.23-5.14 (m, 4H), 3.83 (s, 3H), 3.69 (d, J = 5.9 Hz, 4H). ^{13}C{1H} NMR (100 MHz, $CDCl_3$): 192.0, 155.6, 148.4, 134.1, 131.4, 124.1, 122.3, 118.1, 110.1, 58.2, 55.5. HRMS (ESI-TOF) *m/z*: $[M+H]^+$ calcd for $C_{14}H_{18}NO_2$, 232.1332; found: 232.1320.

Aldehyde **SI-4** (474 mg, 2.05 mmol), 4.1 mL of toluene, *p*-TsOH (17 mg, 0.089 mmol), 1.15 g of crushed molecular sieves, and 5.0 mL of a 2.0 M solution of $MeNH_2$ in THF (10.0 mmol) were heated to 100 °C overnight in a sealed pressure vessel. After cooling, 1.0 g of $NaHCO_3$ was added and the mixture was stirred for 5 min. The mixture was filtered and partitioned between 15 mL Et_2O and 5 mL water. The aqueous layer was further extracted with Et_2O (2 x 5 mL). The combined organic layers were concentrated under reduced pressure. The residue was purified by column chromatography (basic Al_2O_3, gradient ranging from hexanes to 4% EtOAc in hexanes) to provide 403 mg (81%) of the title compound **13b** as a light-yellow oil. ^{1}H NMR (400 MHz, $CDCl_3$): 6.71-6.66 (m, 1H), 6.53 (d, J = 2.7 Hz, 1H), 6.45 (d, J = 8.7 Hz, 1H), 5.94-5.83 (m, 1H), 5.35-5.26 (m, 1H), 5.19-5.14 (m, 1H), 4.19 (d, J = 5.5 Hz, 1H), 3.89-3.71 (m, 3H), 3.75 (s, 3H), 2.43 (s, 3H), 2.38-2.28 (m, 1H), 2.24-2.12 (m, 1H), 2.09-1.98 (m, 2H). ^{13}C{1H} NMR (100 MHz, $CDCl_3$):150.7, 136.6, 135.2, 125.3, 115.2, 113.1, 112.3, 110.5, 78.3, 63.8, 55.5, 52.3, 35.8,34.1, 33.9. HRMS (ESI-TOF) *m/z*: $[M+H]^+$ calcd for $C_{15}H_{21}N_2O$, 245.1648; found: 245.1648.

9-Allyl-10-butyl-6,7,8,9-tetrahydro-5H-5,8-epiaminopyrido[2,3-b]azepine (**13c**).

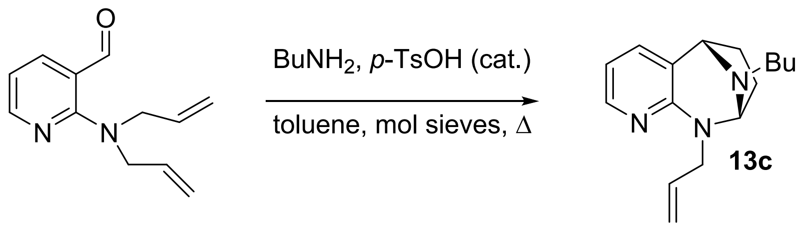

13c

2-*N*,*N*-diallylpyridine-3-carbaldehyde[7] (564 mg, 2.82 mmol), 7.0 mL of toluene, *p*-TsOH (26 mg, 0.136 mmol), 940 μL of $BuNH_2$ (8.46 mmol), and 3.00 g of molecular sieves were stirred at 105 °C for 4 h. After cooling, 3.0 g of $NaHCO_3$ was added and the mixture was stirred for 5 min. The mixture was vacuum filtered. Water (20 mL) was added to the filtrate and the mixture was partitioned. The aqueous layer was further extracted with Et_2O (2 x 30 mL). The combined organic layers were concentrated under reduced pressure. The residue was purified by column chromatography (SiO_2, gradient ranging from 1.5 % to 10% EtOAc in hexanes) to provide 590 mg (81%) of the title compound **13c** as a yellow oil. ^{1}H NMR (400 MHz, $CDCl_3$): 7.98-7.95 (m, 1H), 7.04 (dd, *J* = 7.3, 1.8 Hz, 1H), 6.43 (dd, *J* = 7.1, 5.3 Hz, 1H), 5.97-5.86 (m, 1H), 5.28-5.22 (m, 1H), 5.18-5.13 (m, 1H), 4.47-4.38 (m, 1H), 4.36 (d, *J* = 5.0 Hz, 1H), 3.95-3.86 (m, 1H), 3.85 (d, *J* = 6.4 Hz, 1H), 2.53-2.45 (m, 1H), 2.44-2.35 (m, 1H), 2.30-2.21 (m, 1H), 2.20-2.05 (m,2H), 1.93-1.86 (m, 1H), 1.54-1.43 (m, 2H), 1.35-1.22 (m, 2H), 0.89 (t, *J* = 7.3 Hz, 3H). ^{13}C{1H} NMR (100 MHz, $CDCl_3$):153.6, 146.3, 135.1, 132.9, 120.2, 116.4, 111.5, 74.4, 61.7, 48.0, 46.3, 35.0, 34.6, 30.6, 20.7, 14.0. HRMS (ESI-TOF) *m/z*: $[M+H]^+$ calcd for $C_{16}H_{24}N_3$, 258.1965; found: 258.1964.

1-Allyl-5-methyl-10-butyl-2,3,4,5-tetrahydro-1H-2,5-epiaminobenzo[b]azepine (**13d**).

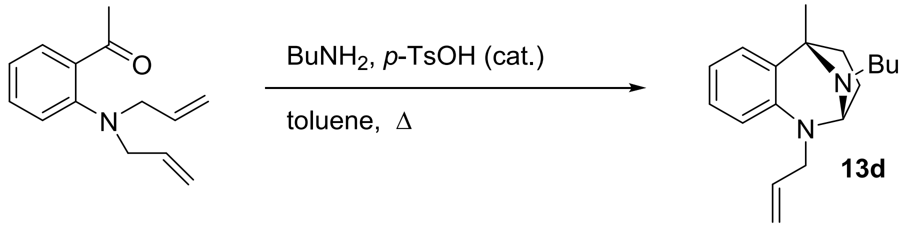

13d

2-*N*,*N*-diallylaminoacetophenone[8] (1.80 g, 8.37 mmol), 30 mL of toluene, *p*-TsOH (80 mg, 0.42 mmol), 2.9 mL of $BuNH_2$ (25 mmol), were refluxed for 2 h. A still-head was fitted onto the reaction vessel and 12 mL of the volume was distilled to azeotrope off water. The volume was replaced by 10 mL of toluene and 1.5 mL of $BuNH_2$. This was refluxed for an additional 2 h then 10 mL of the volume was again removed by distillation. After cooling, 3.0 g of $NaHCO_3$ was added and the mixture was stirred for 5 min. Water was added and the mixture was partitioned. The aqueous layer was further extracted with Et_2O (2 x 15 mL). The combined organic layers were concentrated under reduced pressure. The residue was purified by column chromatography (SiO_2, gradient ranging from 0.5 % to 4% EtOAc in hexanes) to provide 1.19 g (41%) of the title

compound **13d** as a colorless oil. ^{1}H NMR (400 MHz, $CDCl_3$): 7.08 (m, 1H), 6.99 (dd, *J* = 7.5, 1.1 Hz, 1H), 6.71-6.57 (m, 1H), 6.45 (d, *J* = 8.2 Hz, 1H), 5.94-5.83 (m, 1H), 5.33-5.26 (m, 1H), 5.18-5.14 (m, 1H), 4.38 (d, *J* = 5.0 Hz, 1H), 3.88-3.77 (m, 1H), 3.78-3.70 (m, 1H), 2.47-2.39 (m, 1H), 2.35-2.27 (m, 1H), 2.13- 2.02 (m, 2H), 2.01- 1.93 (m, 2H) 1.64-1.46 (m, 2H), 1.53 (s, 3H), 1.38-1.25 (m, 2H), 0.91 (t, *J* = 7.3 Hz, 3H). ^{13}C{1H} NMR (100 MHz, $CDCl_3$):143.2, 134.8, 128.2, 127.3, 124.6, 115.7, 115.6, 109.5, 76.3, 62.8, 51.8, 43.2, 43.1, 32.2, 31.1, 21.9, 20.9, 14.1. HRMS (ESI-TOF) *m/z*: $[M+H]^+$ calcd for $C_{18}H_{27}N_2$, 271.2169; found: 271.2167.

9-Allyl-10-butyl-5-methyl-6,7,8,9-tetrahydro-5H-5,8-epiaminopyrido[2,3-b]azepine (**13e**).

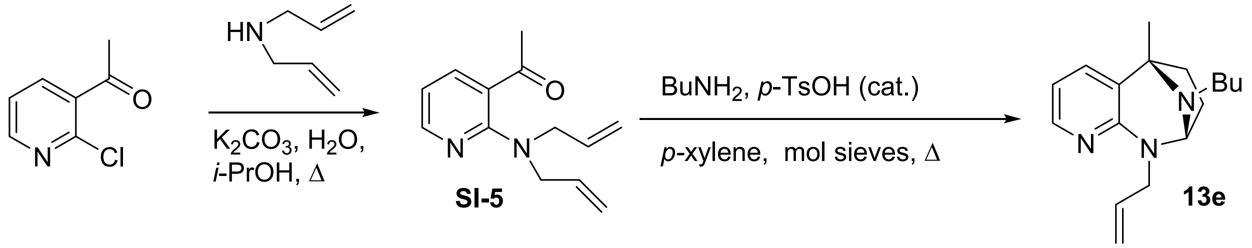

To the chloropyridyl ketone (960 mg, 6.2 mmol) was added diallylamine (3.04 mL, 24.6 mmol), K_2CO_3 (3.40 g, 24.6 mmol), water (3.1 mL), and *i*-PrOH (3.1 mL). This mixture was refluxed for 24 h. After cooling, the mixture was extracted with Et_2O (3 x 15 mL). The combined organic layers were concentrated under reduced pressure. The residue was purified by column chromatography (SiO_2, gradient ranging from 1 to 3 % EtOAc in hexanes) to provide 1.01 g (76%) of **SI-5** as a pale-yellow oil. ^{1}H NMR (400 MHz, $CDCl_3$): 8.27 (dd, *J* = 4.8, 1.6 Hz, 1H), 7.73 (dd, *J* = 7.5, 1.6 Hz, 1H), 6.78-6.74 (m, 1H), 5.88-5.77 (m, 2H), 5.23-5.16 (m, 4H), 3.98 (d, *J* = 5.9 Hz, 4H), 2.55 (s, 3H). ^{13}C{1H} NMR (100 MHz, $CDCl_3$): 200.6, 157.8, 149.8, 138.3, 133.8, 123.2, 118.0, 113.9, 53.0, 28.1. HRMS (ESI-TOF) *m/z*: $[M+H]^+$ calcd for $C_{13}H_{17}N_2O$, 217.1341; found: 217.1335.

Ketone **SI-5** (732 mg, 3.39 mmol), 17 mL of *p*-xylene, *p*-TsOH (32 mg, 0.17 mmol), 1.97 mL of $BuNH_2$ (17 mmol) and 2.00 g of molecular sieves were stirred at 130 ^{o}C for 48 h. After cooling, 2.0 g of $NaHCO_3$ was added and the mixture was stirred for 5 min and vacuum filtered. Water (10 mL) was added to the filtrate and the mixture was partitioned. The aqueous layer was further extracted with Et_2O (2 x 15 mL). The combined organic layers were concentrated under reduced pressure. The residue was purified by column chromatography (SiO_2, gradient ranging from hexanes to 10% EtOAc in hexanes) to provide 610 mg (62%) of the title compound **13e** as a colorless oil. ^{1}H NMR (400 MHz, $CDCl_3$): 7.95-7.92 (m, 1H), 7.11 (dd, *J* = 7.1, 1.6 Hz, 1H), 6.46-6.42 (m, 1H), 5.98-5.86 (m, 1H), 5.29-5.14 (m, 2H), 4.48 (d, *J* = 4.8 Hz, 1H), 4.47-4.38 (m, 1H), 3.93-3.84 (m, 1H), 2.41-2.33 (m, 1H), 2.24-2.15 (m, 1H), 2.13-1.92 (m, 4H), 1.58-1.42 (m, 2H), 1.49 (s, 3H), 1.37-1.24 (m, 2H), 0.90 (t, *J* = 7.3 Hz, 3H). ^{13}C{1H} NMR (100 MHz, $CDCl_3$): 153.5, 145.8, 135.1, 131.3, 124.0, 116.5, 111.5, 74.1, 62.9, 48.2, 42.7,

42.4, 32.8, 31.1, 21.2, 20.8, 14.0. HRMS (ESI-TOF) *m/z*: $[M+H]^+$ calcd for $C_{17}H_{26}N_3$, 272.2126; found: 272.2121.

trans-N-Butyl-N,1,2-trimethyl-2,3,4,5-tetrahydro-1H-benzo[b]azepine-5-amine (**9a**, alternative synthesis). The *N*-de-allylated intermediate derived from **11** (128 mg, 0.520 mmol) is diluted with 1.0 mL of THF and cooled to -84°C. A 2.5 M solution of BuLi in hexanes (0.25 mL, 0.62 mmol) was added, and the mixture was allowed to warm to rt over 30 min. The mixture was recooled to -84°C and iodomethane (39 uL, 0.62 mmol) was added. The mixture was allowed to warm to rt and stirred for 12 h. A 10% aqueous solution of K_2CO_3 (0.5 mL) was added along with 0.5 mL of Et_2O. The mixture was partitioned, and the aqueous phase was further extracted with Et_2O (2 x 1 mL). The combined organic layers were concentrated under reduced pressure. The residue was purified by column chromatography (basic Al_2O_3, gradient ranging from hexanes to 2% EtOAc in hexanes) to provide 63 mg (78%) of **9a** as a colorless oil. ^1H NMR (400 MHz, $CDCl_3$): 7.31 (d, *J* = 7.8 Hz, 1H), 7.23-7.17 (m, 1H), 7.02-6.93 (m, 2H), 3.74-3.68 (m, 1H), 2.94-2.84 (m, 1H), 2.82 (s, 3H), 2.65-2.58 (m, 1H), 2.28 (s, 3H), 2.24-2.14 (m, 1H), 2.12-2.02 (m, 1H), 1.89-1.83 (m, 1H), 1.58-1.41 (m, 4H), 1.33-1.20 (m, 2H), 1.16 (d, *J* = 6.4 Hz, 3H), 0.91 (t, *J* = 7.3 Hz, 3H). $^{13}C\{1H\}$ NMR (100 MHz, $CDCl_3$): 148.4, 134.5, 127.9, 126.6, 121.3, 118.3, 63.9, 58.0, 54.8, 39.2, 39.0, 29.8, 29.7, 24.4, 20.7, 16.5, 14.1. HRMS (ESI-TOF) *m/z*: $[M+H]^+$ calcd for $C_{17}H_{29}N_2$, 261.2325; found: 261.2321.

Figure 1. Comparison of Structure and NMR Signals at C-5 of Benzazepines 8 and 9a-k

Compound	Structure	^C ^{13}C ppm	***H** ^{1}H NMR tabulation	Signal Itself
**8**	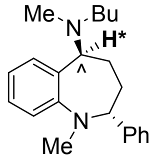	63.1	3.88 (t, *J* = 4.8 Hz)	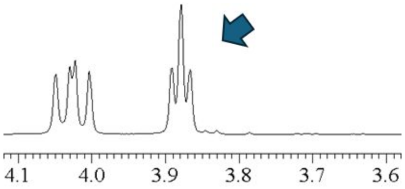
**9a**	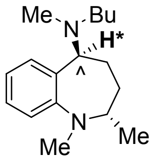	63.9	3.71 ppm, (dd, *J* = 10.5, 7.3 Hz)	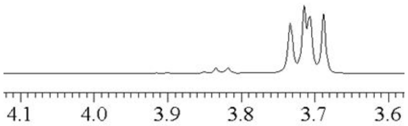
**9b**	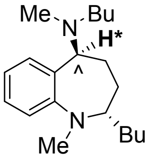	63.0	3.63 ppm, (dd, *J* = 10.3, 7.1 Hz)	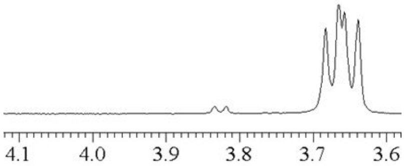
**9c**	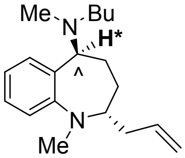	64.3	3.69 ppm, (dd, *J* = 10.3, 7.5 Hz)	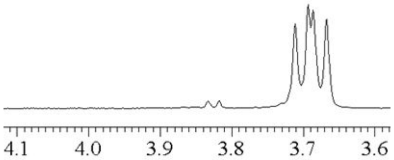
**9d**	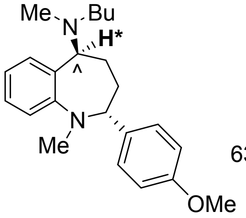	63.3	3.83 ppm, (t, *J* = 4.8 Hz)	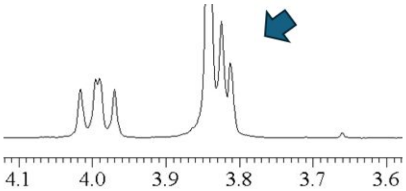
**9e**	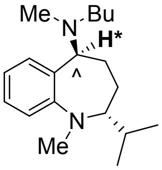	67.0	3.69 ppm, (dd, *J* = 8.9, 7.1 Hz)	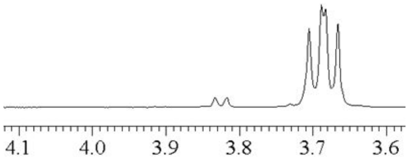
**9g**	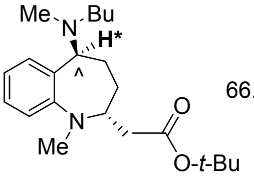	66.0	3.68 ppm, (t, *J* = 7.6 Hz)	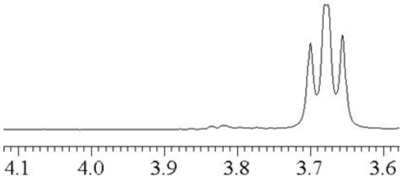
**9h**	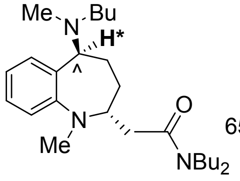	65.0	3.67 ppm, (dd, *J* = 9.6, 7.8 Hz)	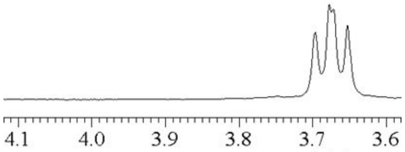

Compound	Structure	^C ^{13}C ppm	***H** ^1H NMR tabulation	Signal Itself
**9i**	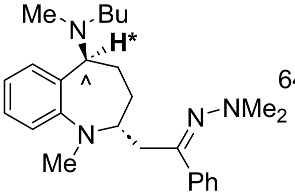	64.1	3.71 ppm, (dd, *J* = 10.5, 7.3 Hz)	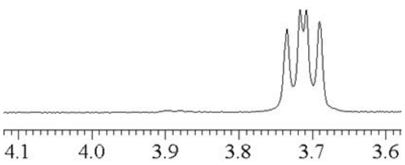
**9j**	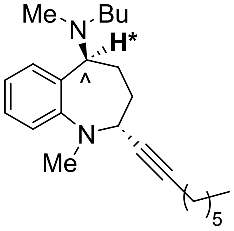	64.3	3.81 ppm, (t, *J* = 9.2 Hz)	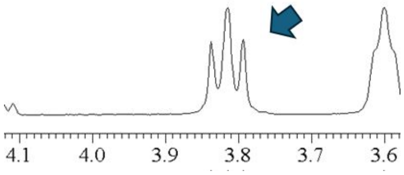
**9k**	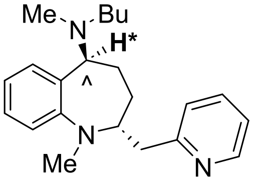	65.0	3.76 ppm, (dd, *J* = 10.1, 7.8 Hz)	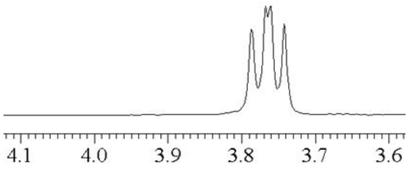

In each instance the **H*** proton signal appears as either a *dd* or an apparent *t*, depending on the compound, in a chemical shift range of 3.61 to 3.88 ppm. Similarly, the **^C** carbon signal appears over a narrow range of 63.1 to 67.0 ppm. We consider this compelling evidence for the same diastereomer being present in each case. Please see Figure 2, p. S13 for further explanation.

Figure 2. Correlation of the Structure of Benzamide 12 and Ring-Opened Product 9a

This is useful given that the *N*-allylic CH_2 obscures the C-5 proton of **11**, the precursor of **12**.

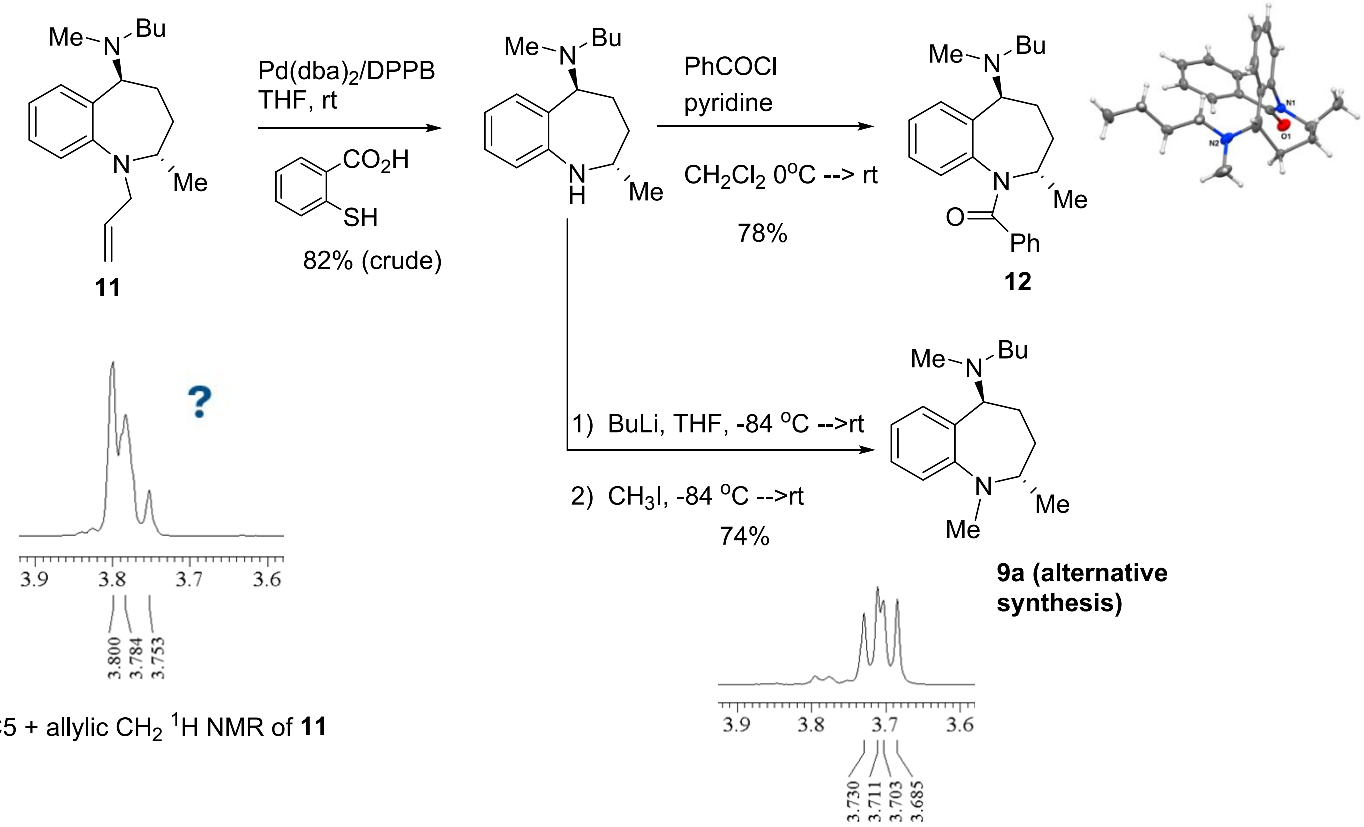

C5 + allylic CH_2 ^{1}H NMR of **11**

C5 ^{1}H NMR of **9a** via **11**

Note the congruence of the C5 proton signals of **9a** constructed via ring-opening of **7b** with MeMgBr (Table 2, entry 1, also p. S11) and **9a** constructed via **11**, whose relative stereochemistry has been unambiguously assigned via the crystal structure of **12**. Please see pp. S27-28 for full ^{1}H NMR spectra of **9a** and **9a** prepared via the alternative synthetic route.

Additional X-ray information for 12

The central, fused-ring system of the molecule is disordered over two positions; most noticeable in C1-C2-C3-C4 where two positions (~60:40) were easily located from the difference map, refined on their own free variable, and restrained using SADI commands to keep the two parts similar. The disorder likely extends into the aromatic portion of the fused-ring system as indicated by the overall look of the ellipsoids in this region and the B-Level Hirshfeld alert in the checkcif report for two carbons in this ring. All attempts to model disorder in this aromatic ring resulted in unstable refinements that required a large number of restraints and constraints for little to no improvement in the R-factor of the final model. The disorder in this region does not indicate a misassignment of the final structure, nor does it affect the relative stereochemistry of the three substituents on the 7-membered ring which is the major takeaway from this structure. Thus, it was left unmodelled in the final solution.

Table 1. Crystal data and structure refinement for Compound 12.

CCDC number	2452689

Empirical formula	$C_{23}H_{30}N_2O$
Formula weight	350.49
Temperature [K]	114(2)
Crystal system	monoclinic
Space group (number)	$P2_1/c$ (14)
a [Å]	8.0420(11)
b [Å]	15.2769(15)
c [Å]	16.1649(17)
α [°]	90
β [°]	95.255(8)
γ [°]	90
Volume [Å^3]	1977.6(4)
Z	4
ρcalc [gcm^{-3}]	1.177
μ [mm^{-1}]	0.554
F(000)	760
Crystal size [mm^3]	0.30 × 0.35 × 0.40
Crystal color	colorless
Crystal shape	block
Radiation	CuK_α (λ = 1.5418 Å)
2θ range [°]	7.98 to 136.92 (0.83 Å)
Index ranges	−9 ≤ h ≤ 9
	−18 ≤ k ≤ 18
	−19 ≤ l ≤ 19
Reflections collected	34316
Independent reflections	3623
R_{int} =	0.0475

R_{sigma} =	0.0219
Completeness to θ = 67.680°	99.9
Data / Restraints / Parameters	3623 / 3 / 258
Absorption correction T_{min}/T_{max} (method)	0.809 / 0.851 (Multi-Scan)
Goodness-of-fit on F^2	1.041
Final R indexes [I≥2σ(I)]	R_1 = 0.0478
	wR_2 = 0.1112
Final R indexes [all data]	R_1 = 0.0534
	wR_2 = 0.1165
Largest peak/hole [eÅ^{-3}]	0.20/−0.30
Extinction coefficient	0.0034(3)

Supplement References.

1. Rathke, M.; Sullivan, D. Isolation and Characterization of Lithio *tert*-Butyl Acetate, a Stable Ester Enolate. *J. Am. Chem. Soc.* **1973**, *95*, 3050-3051.

2. Tang, R.; Xie, Y.; Xie, Y.; Xiang, J.; Li, J. TBHP-Mediated Oxidative Thiolation of an sp^3 C-H Bond Adjacent to a Nitrogen Atom in an Amide. *Chem. Comm*. **2011**, *47*, 12867-12869.
3. Collum, D.; Kahne, D.; Gut, S.; DePue, R.; Mohamadi, F.; Wanat, R. Clardy, J.; Van Duyne, G. Substituent Effects on the Stereochemistry of Substituted Cyclohexanone Dimethylhydrazone Alkylations. An X-ray Crystal Structure of Lithiated Cyclohexanone Dimethylhydrazone. *J. Am. Chem. Soc*. **1984**, *106*, 4865-4869.
4. Feng, K.; Ban, Y.; Yuan, P.; Lei, W.; Liu, Q.; Fang, R. Synthesis of 4-Oxoisoxazoline, N-Oxides via Pd-Catalyzed Cyclization of Propargylic Alcohols with *tert*-Butyl Nitrite. *Org. Lett*. **2019**, *21*, 3131-3135.
5. Taber, D.; Guo, P.; Pirnot, M. Conjugate Addition of Lithiated Methyl Pyridines to Enones. *J. Org. Chem*. **2010**, *75*, 5737-5739.
6. Zhang, Y.; Yang, F.; Zheng, L.; Dang, Q.; Bai, X. A Cascade of Acid-Promoted C-O Bond Cleavage and Redox Reactions: From Oxa-Bridged Benzazepines to Benzazepinones. *Org. Lett*. **2014**, *16*, 6041-6043.
7. Zhang, Y.; Zheng, L.; Yang, F.; Zhang, Z.; Dang, Q.; Bai, X. Substituent-Directed Reduction of Cyclic Aminals Leading to Two Different Heterocycles Selectively: Synthesis of Functionalized Nicotines and Pyrido[2,3.*b*]azepines. *Tetrahedron* **2015**, *71*, 1930-1939.
8. Brinson, R.; Jones, P. *N*-Allyl-1,3-Oxazines via a Facile Keto-ene/Cyclization Tandem Reaction. *Tetrahedron Lett*., **2004**, *45*, 6155-6158.

NMR Spectra of Synthesized Compounds.

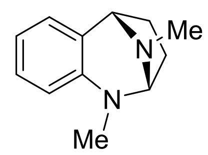

6a

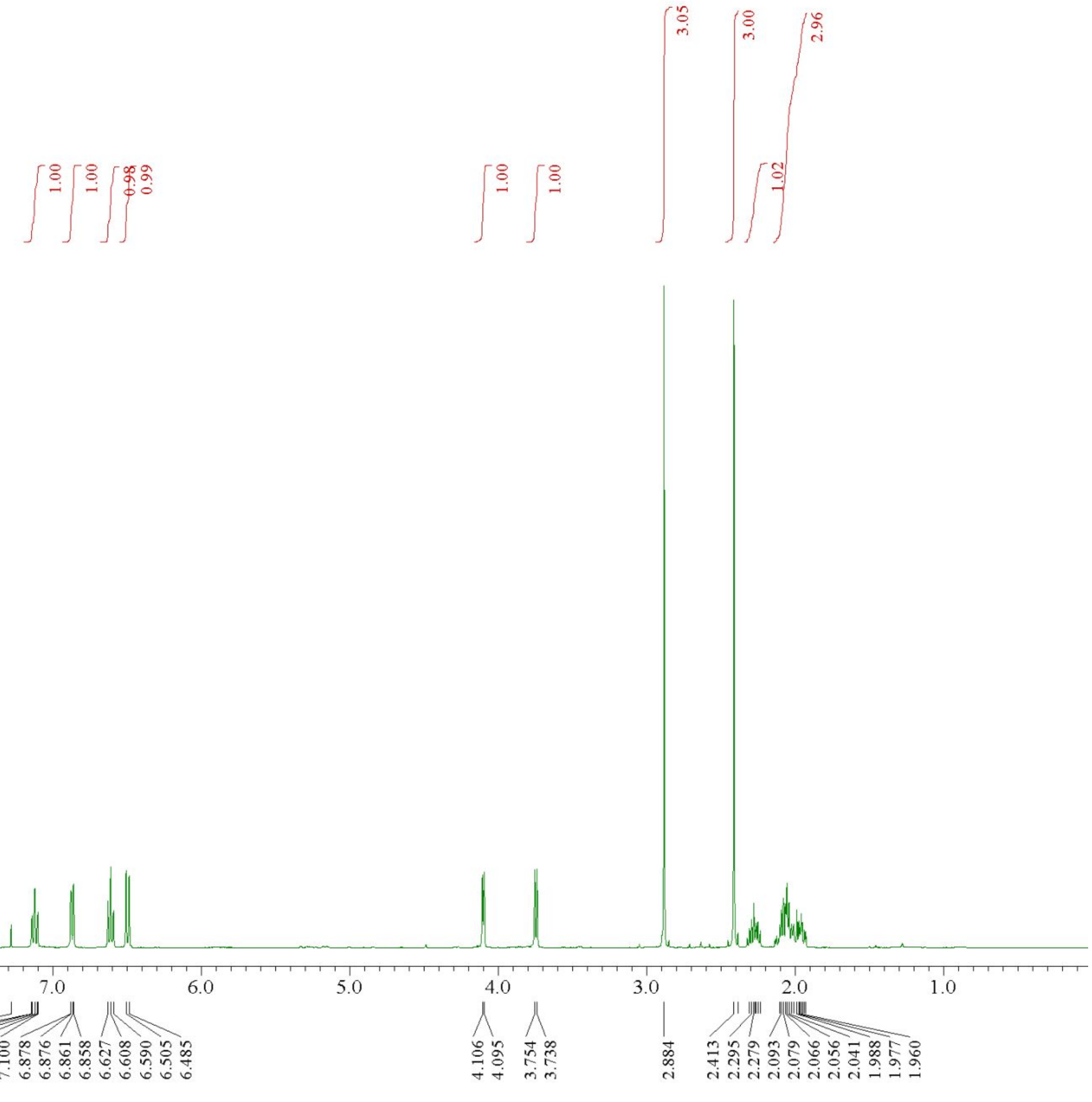

^{1}H NMR, 400 MHz, $CDCl_3$

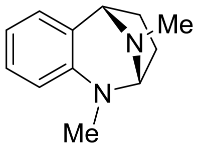

6a

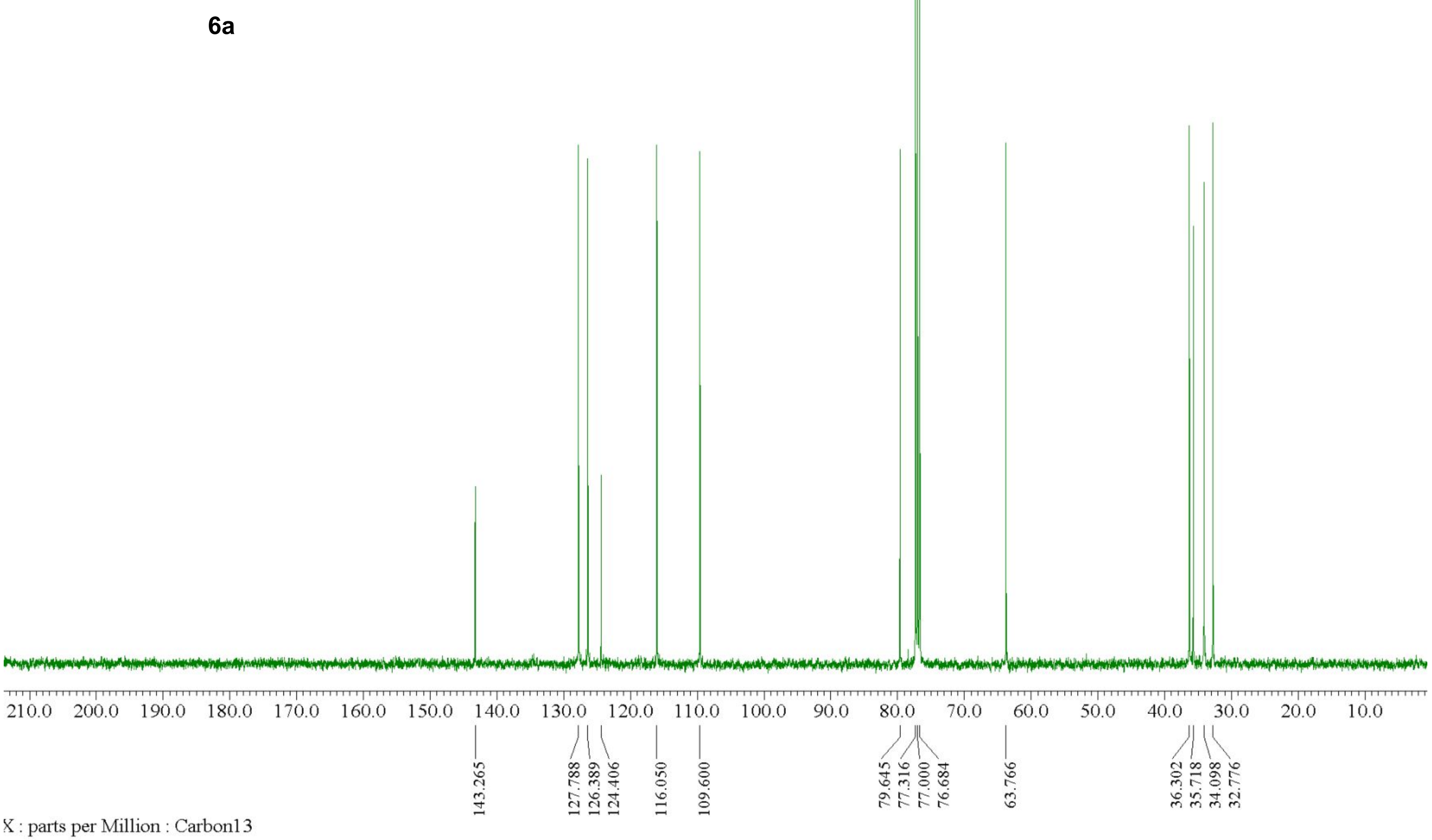

$^{13}C\{1H\}$ NMR, 100 MHz, $CDCl_3$

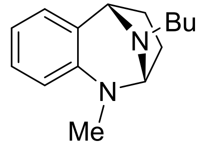

6b

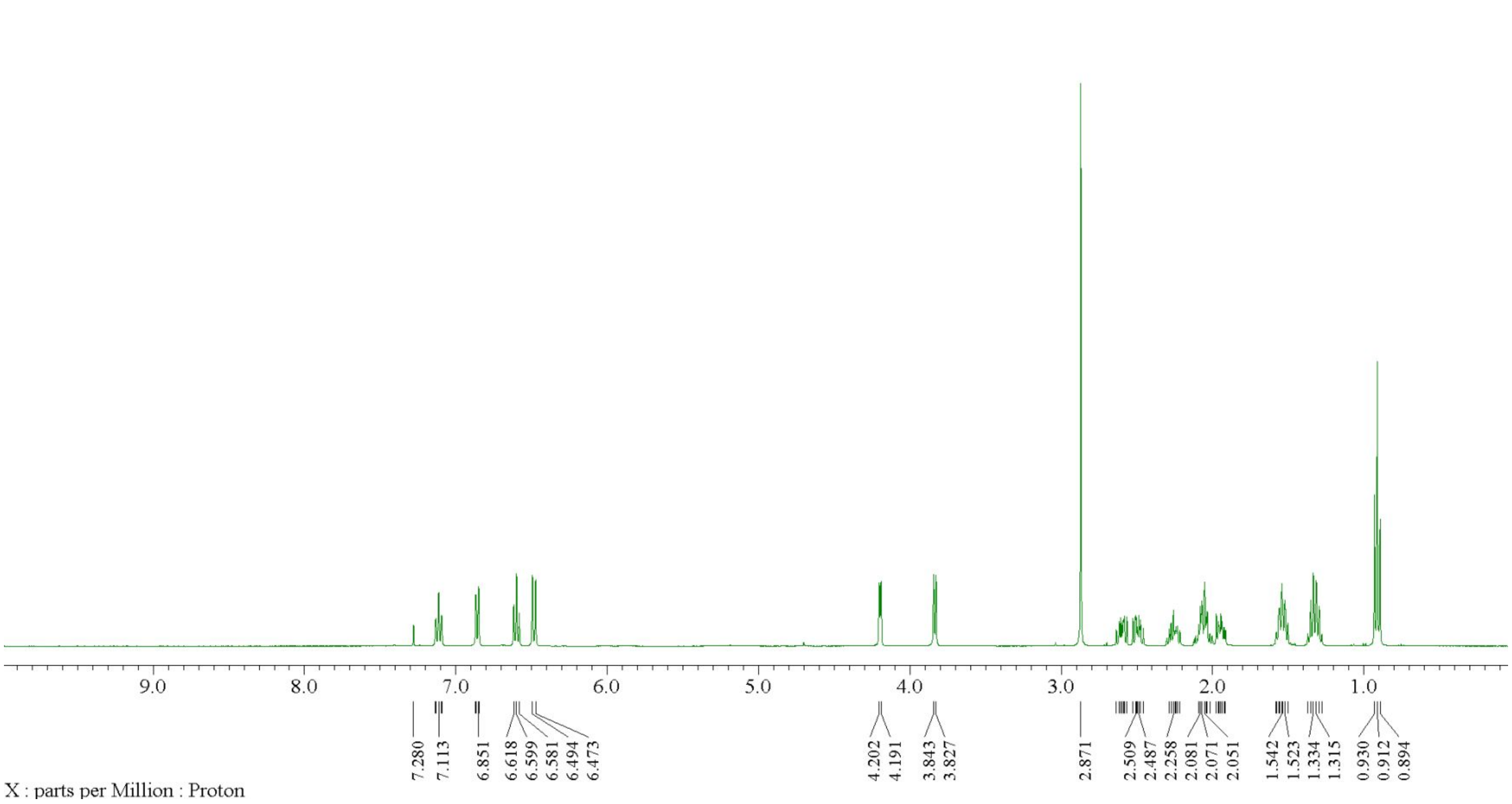

^{1}H NMR, 400 MHz, $CDCl_3$

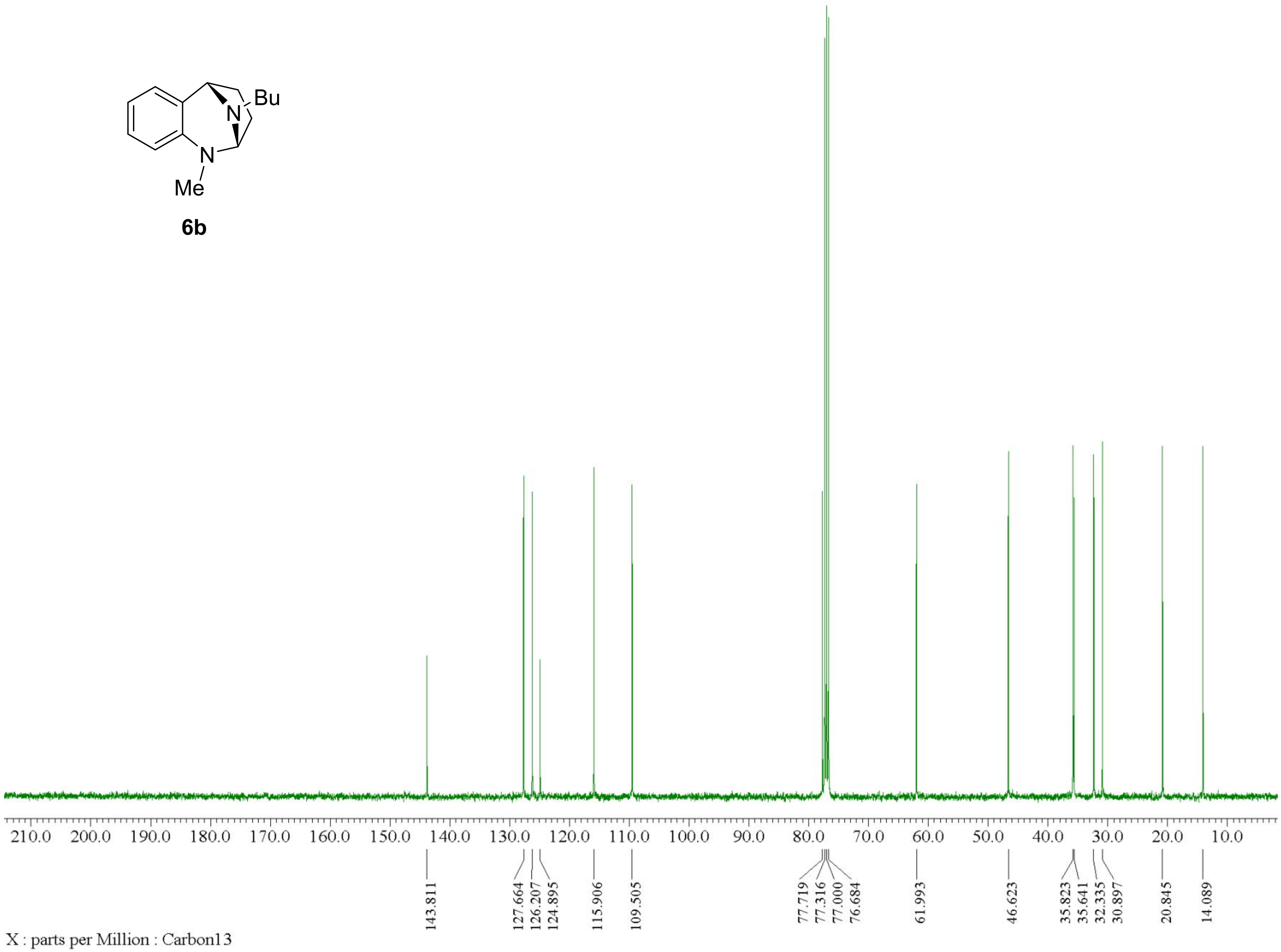

$^{13}C\{^1H\}$ NMR, 100 MHz, $CDCl_3$

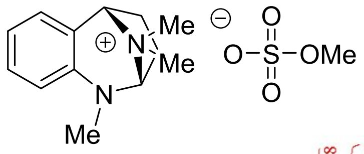

7a

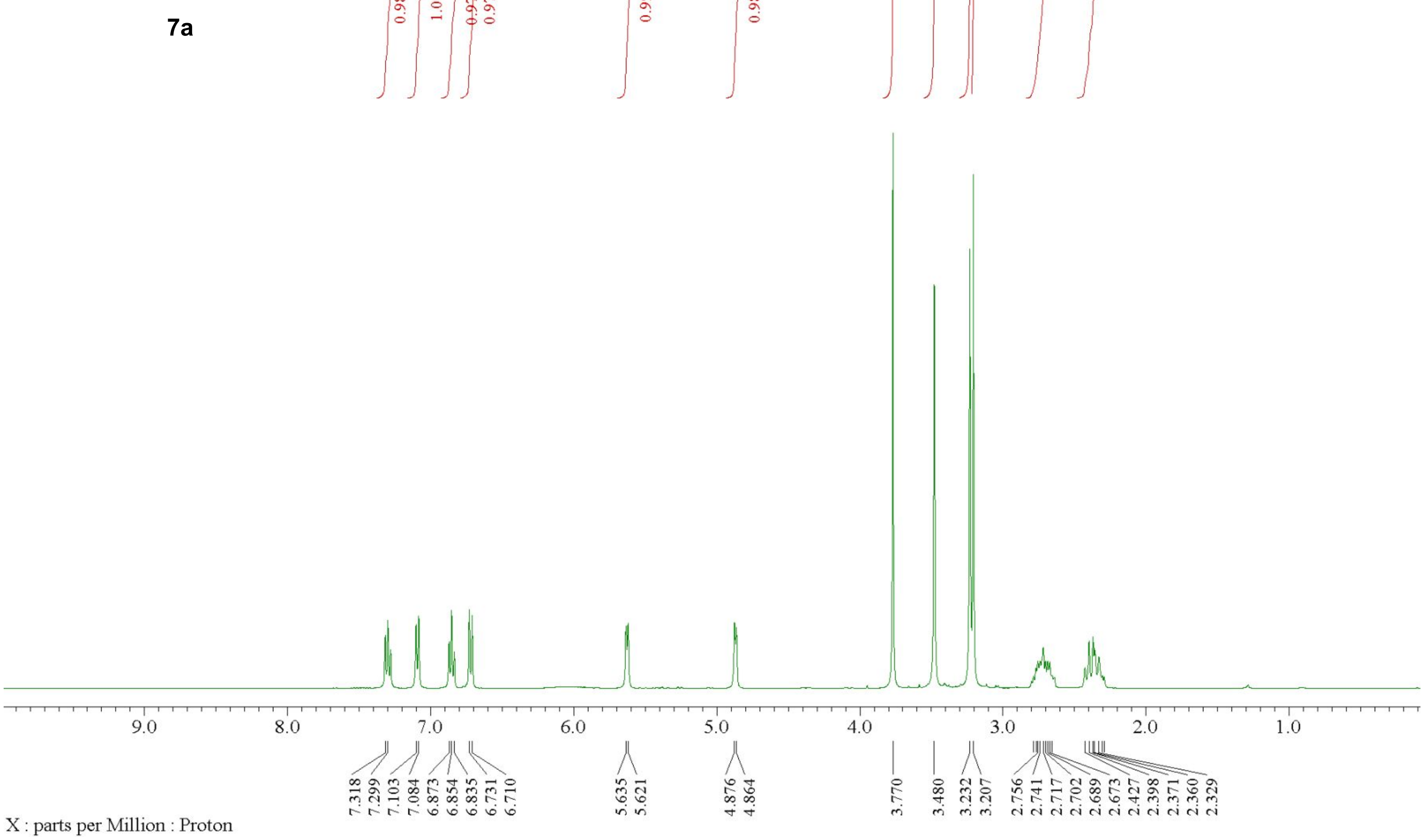

^{1}H NMR, 400 MHz, $CDCl_3$

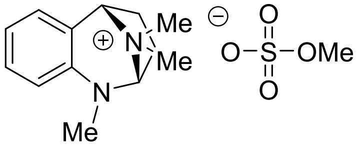

7a

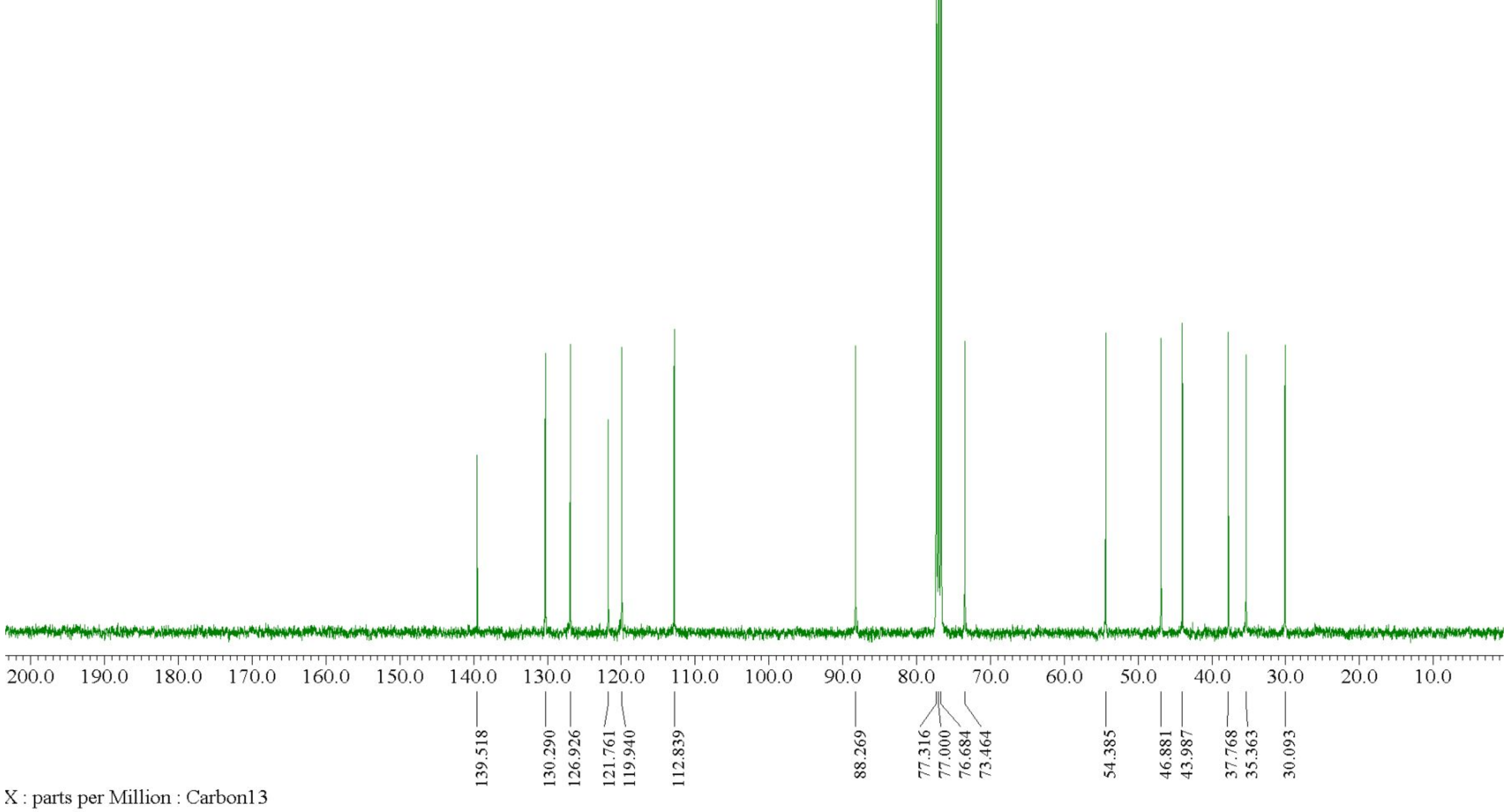

$^{13}C\{1H\}$ NMR, 100 MHz, $CDCl_3$

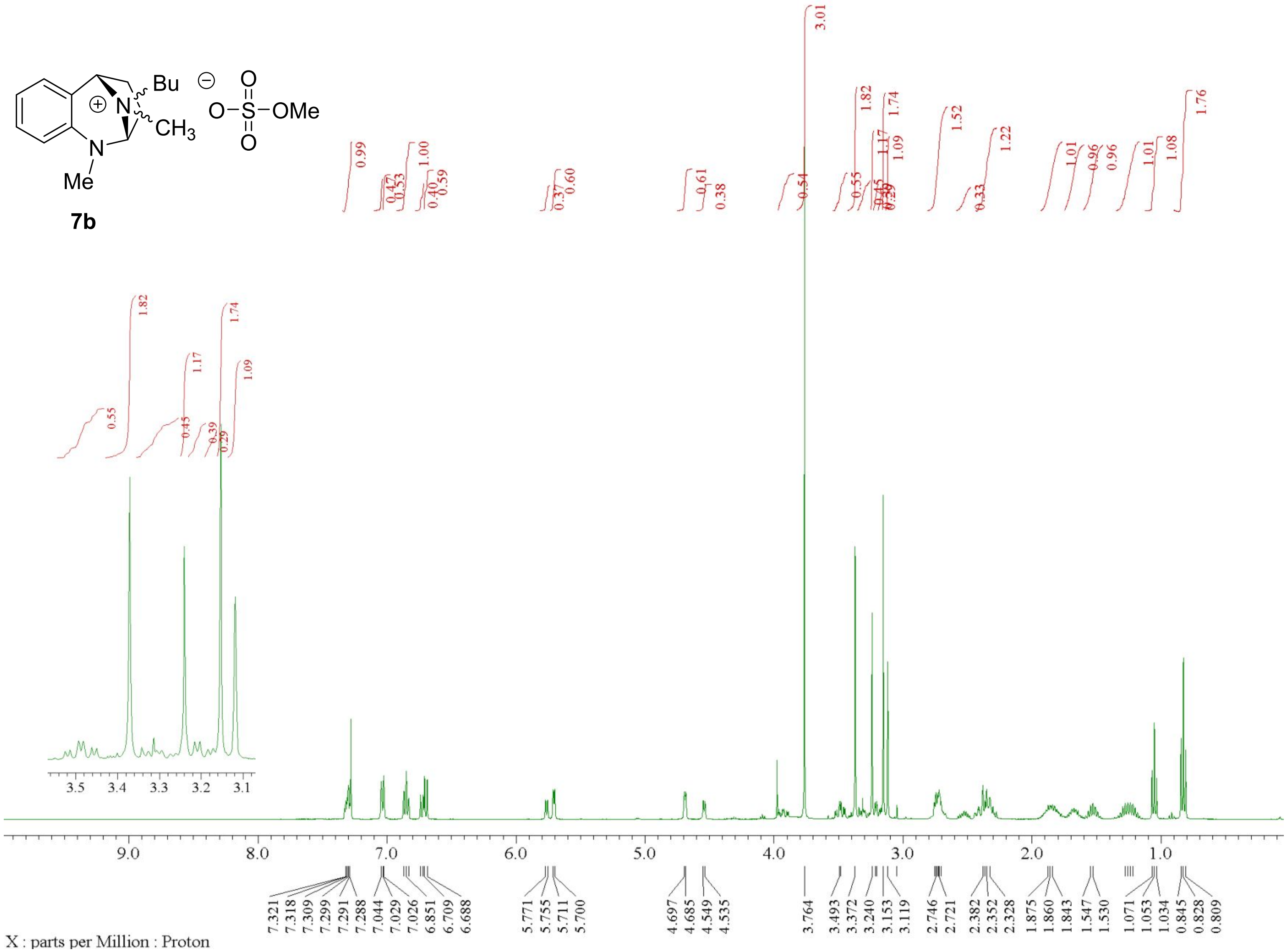

7b

X : parts per Million : Proton

^{1}H NMR, 400 MHz, $CDCl_3$

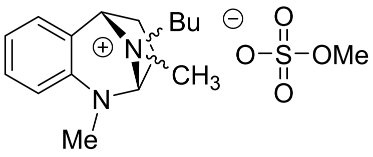

7b

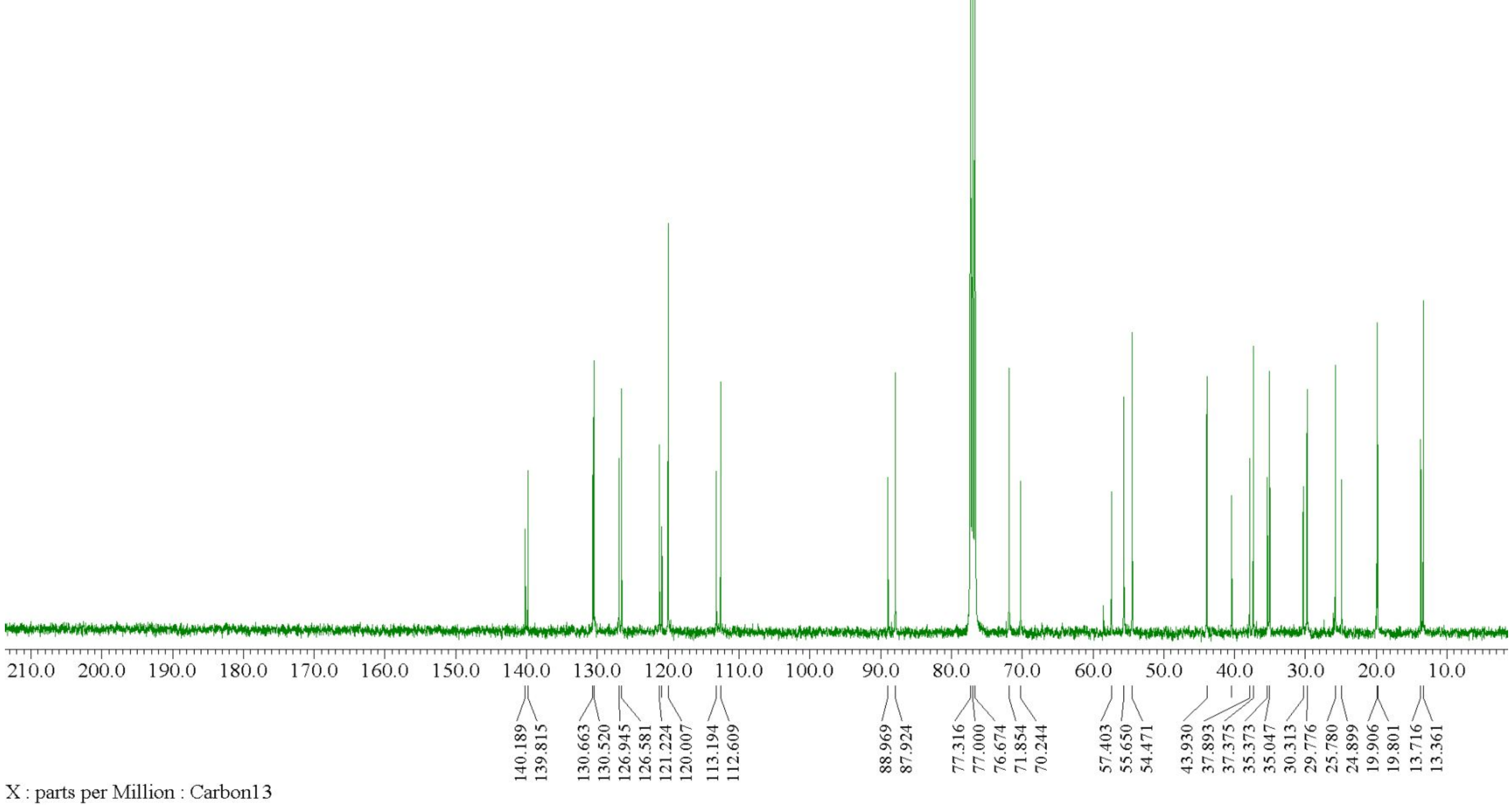

$^{13}C\{1H\}$ NMR, 100 MHz, $CDCl_3$

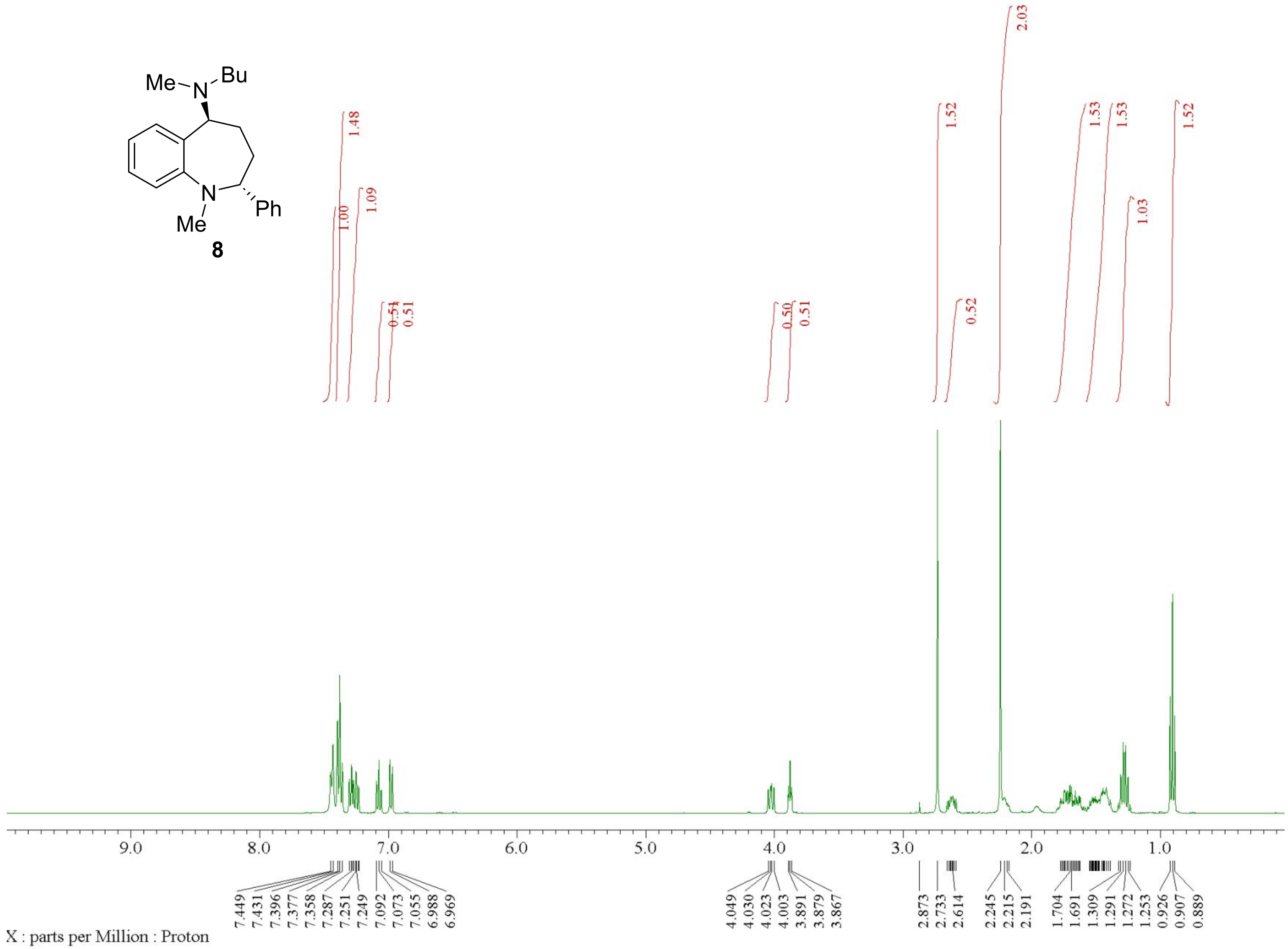

^{1}H NMR, 400 MHz, $CDCl_3$

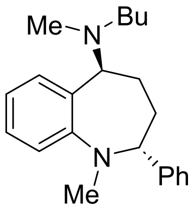

8

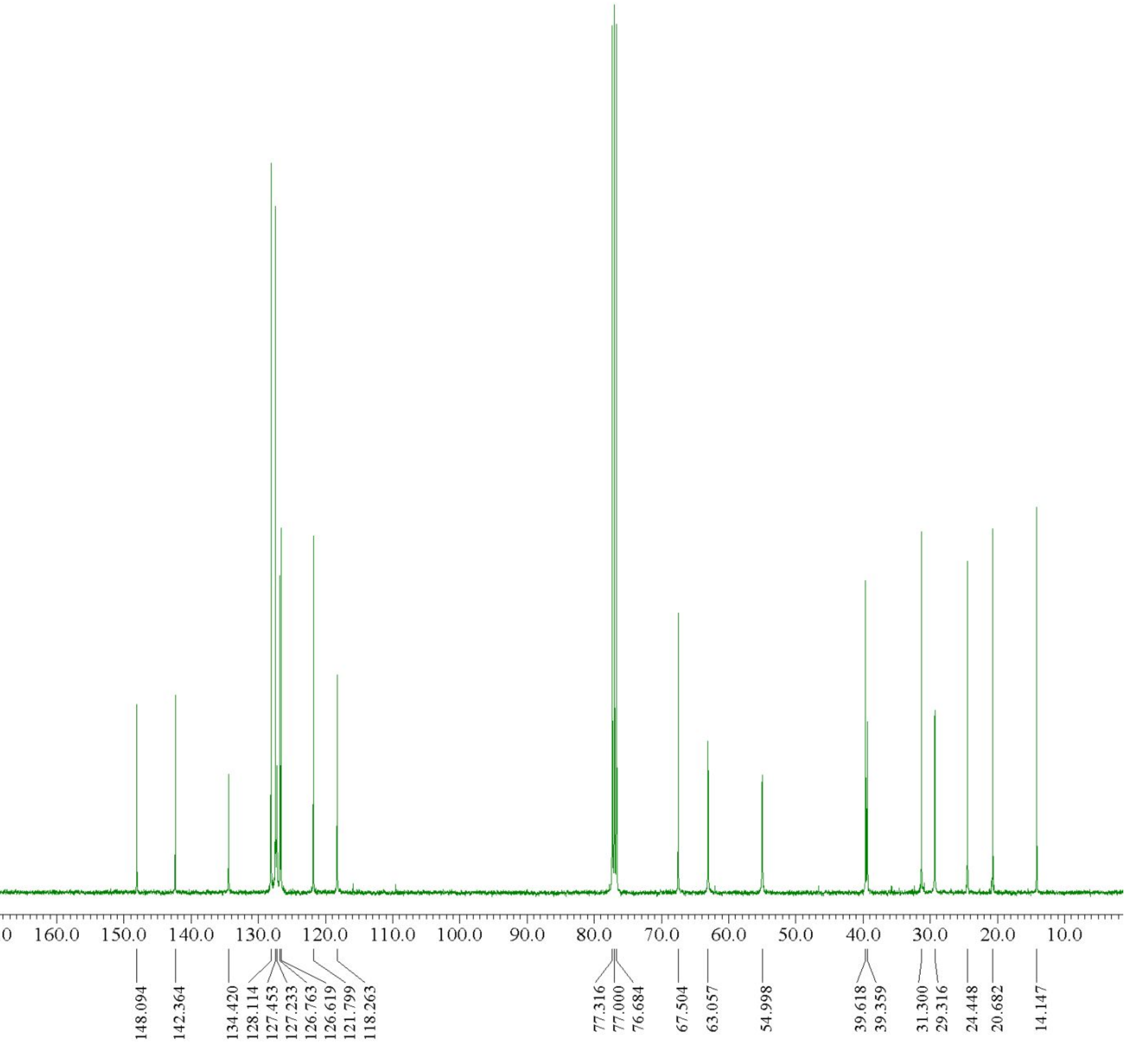

$^{13}C\{1H\}$ NMR, 100 MHz, $CDCl_3$

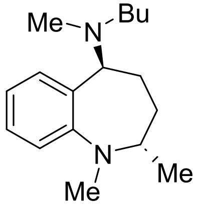

9a

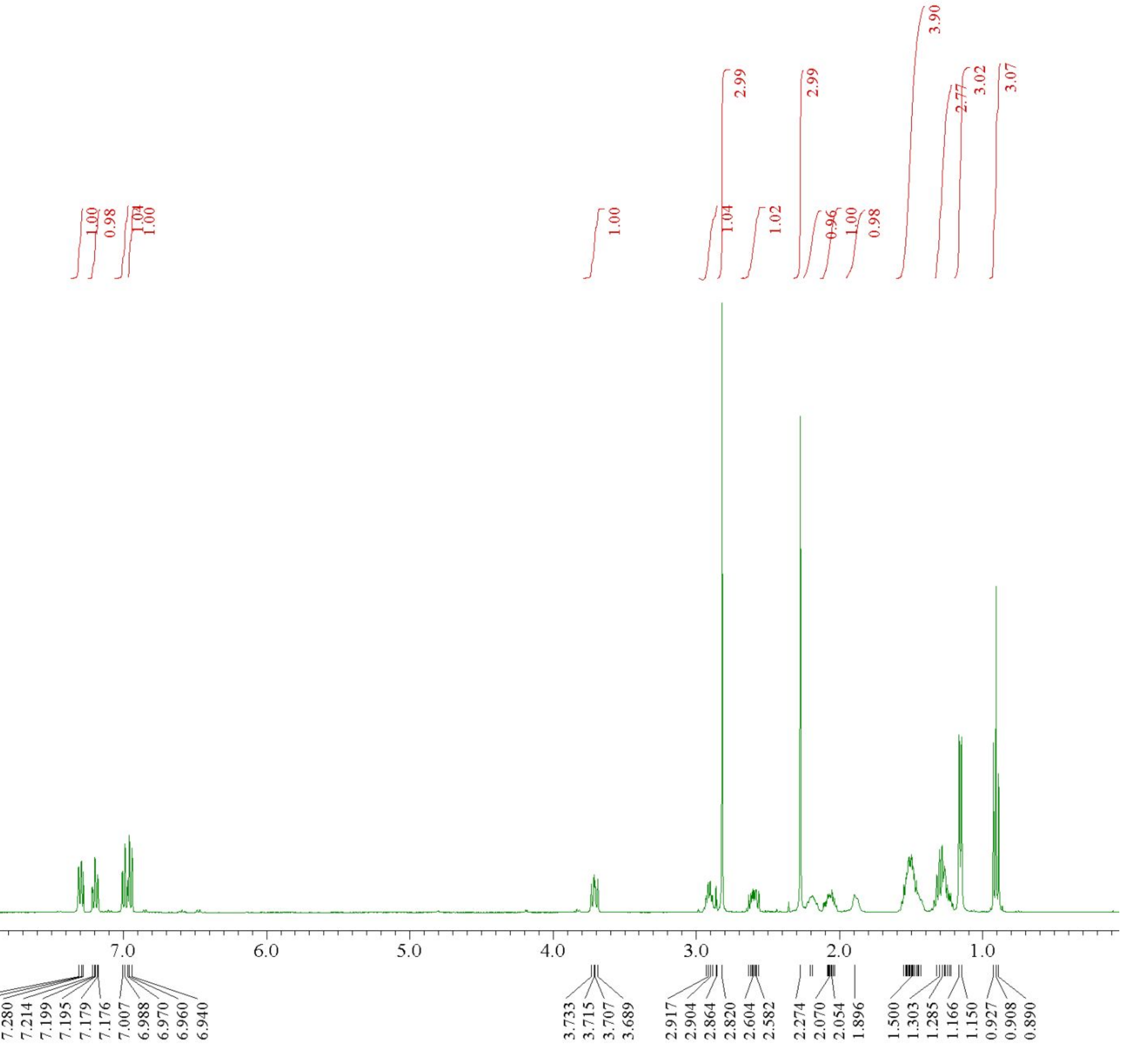

^1H NMR, 400 MHz, $CDCl_3$

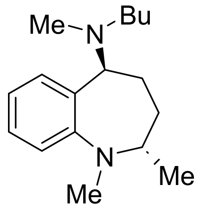

9a
(alternative synthesis)

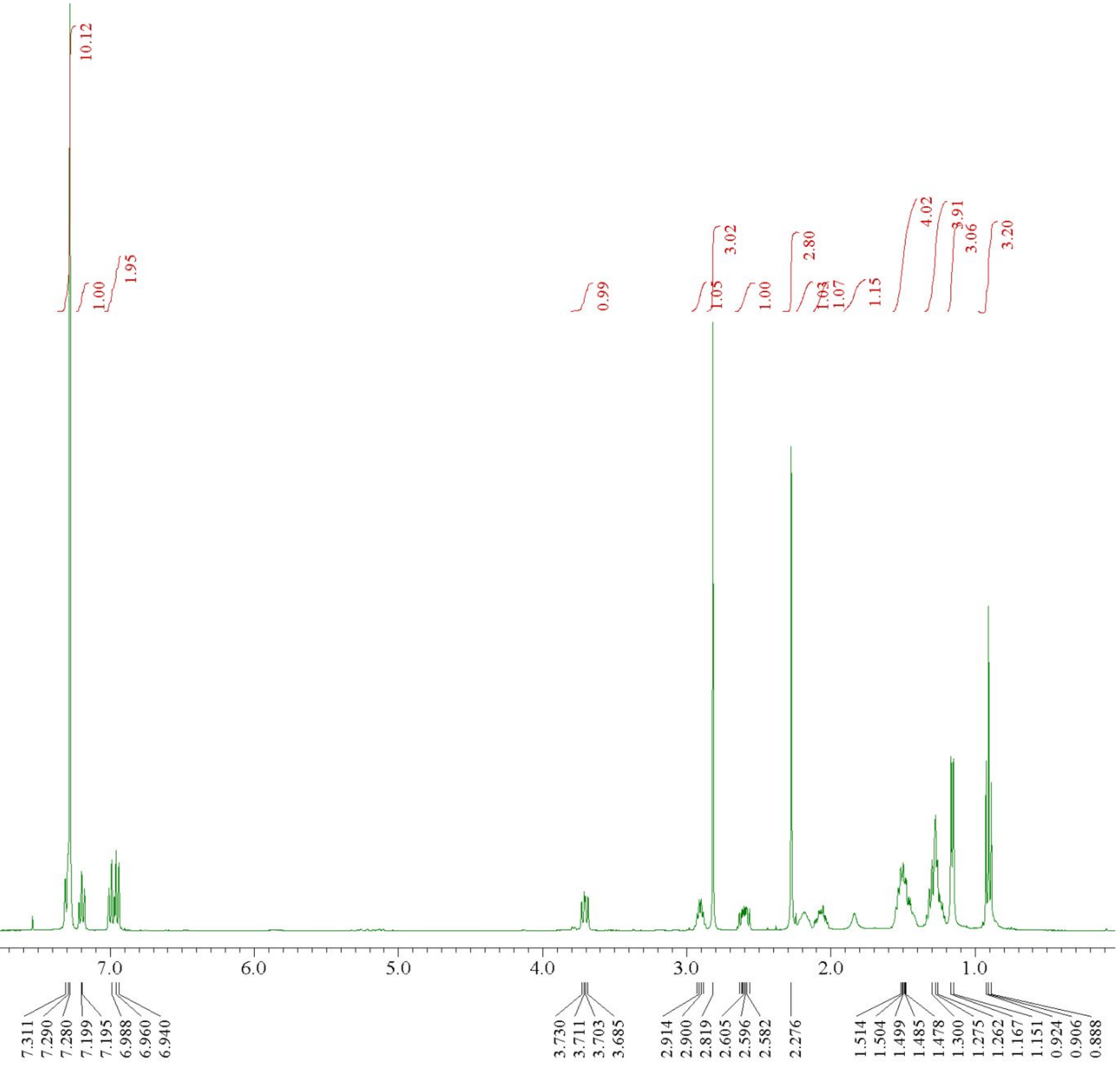

^{1}H NMR, 400 MHz, $CDCl_3$

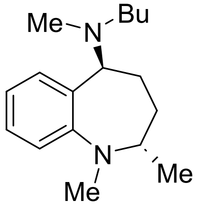

9a

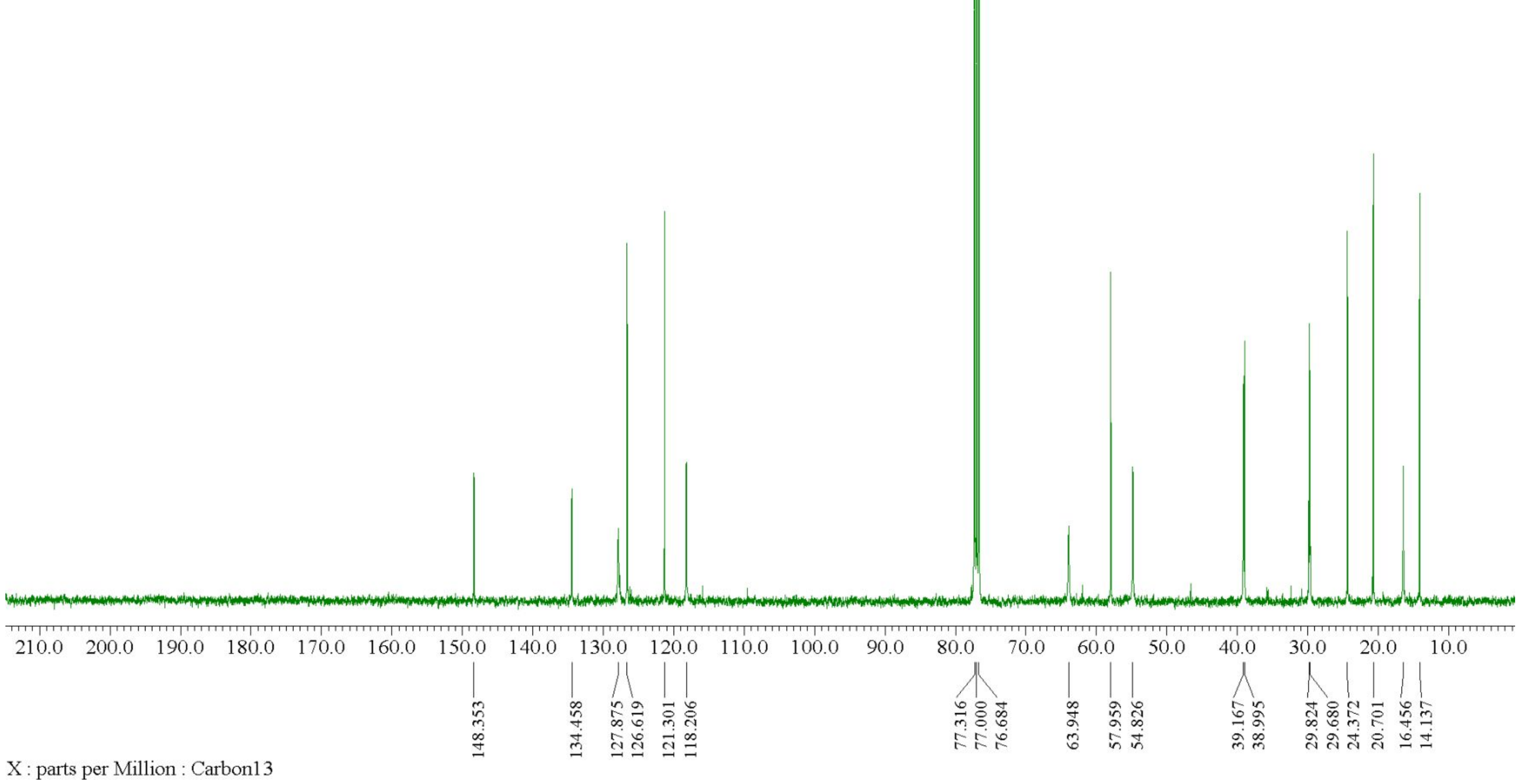

$^{13}C\{1H\}$ NMR, 100 MHz, $CDCl_3$

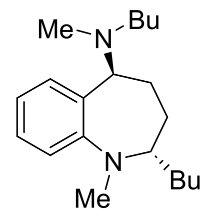

9b

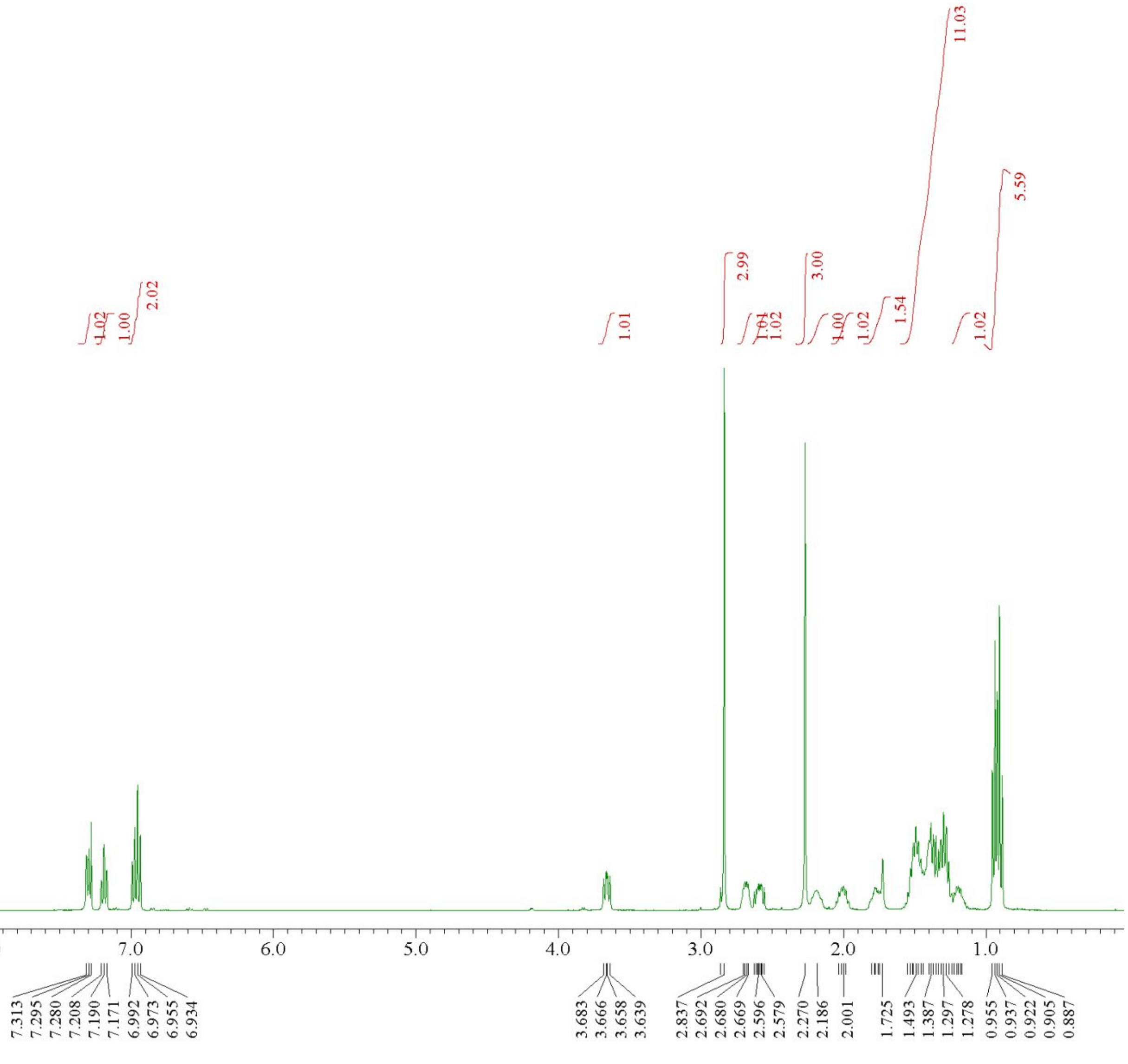

^{1}H NMR, 400 MHz, $CDCl_3$

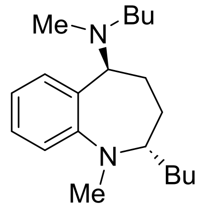

9b

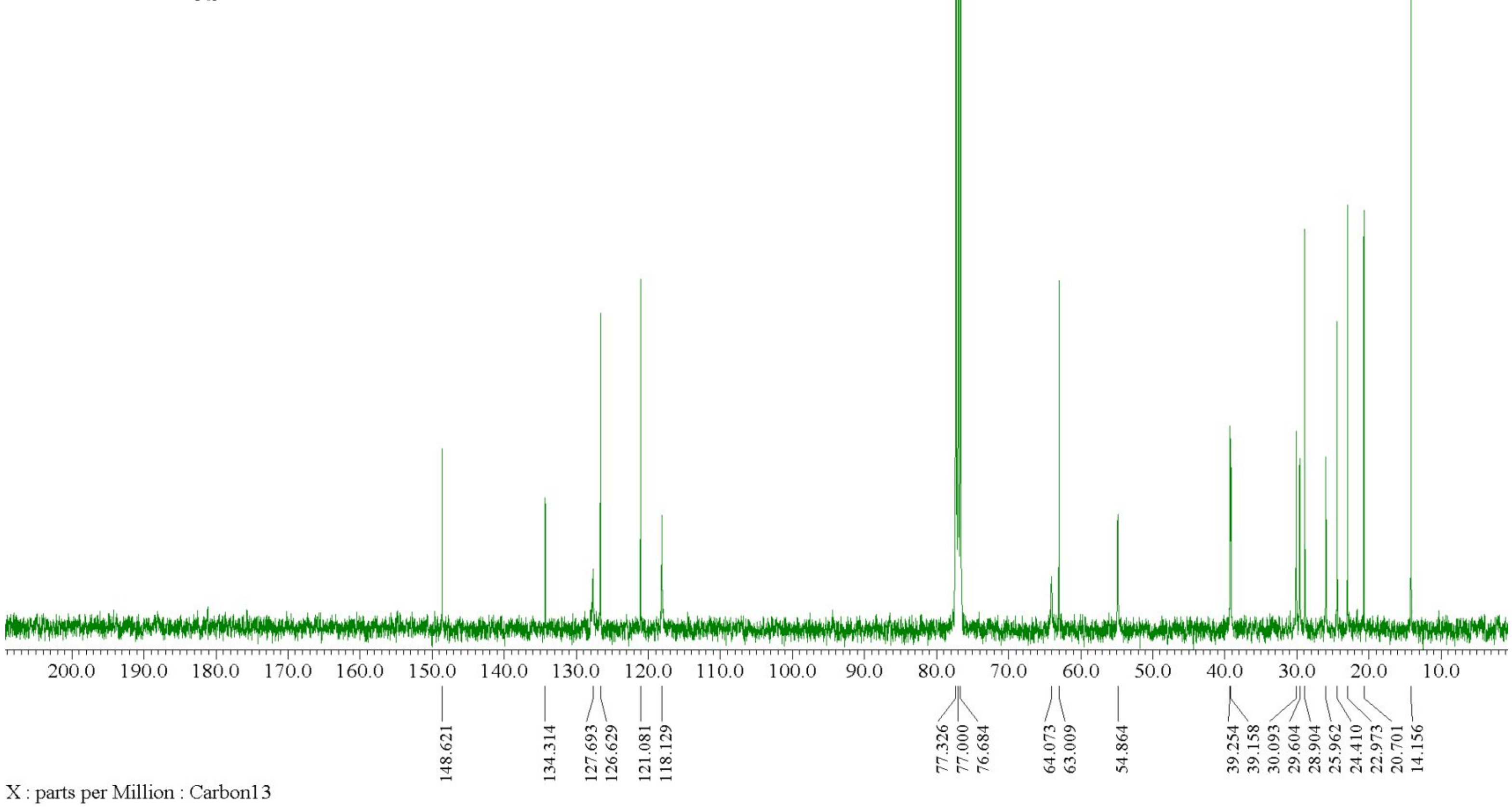

$^{13}C\{1H\}$ NMR, 100 MHz, $CDCl_3$

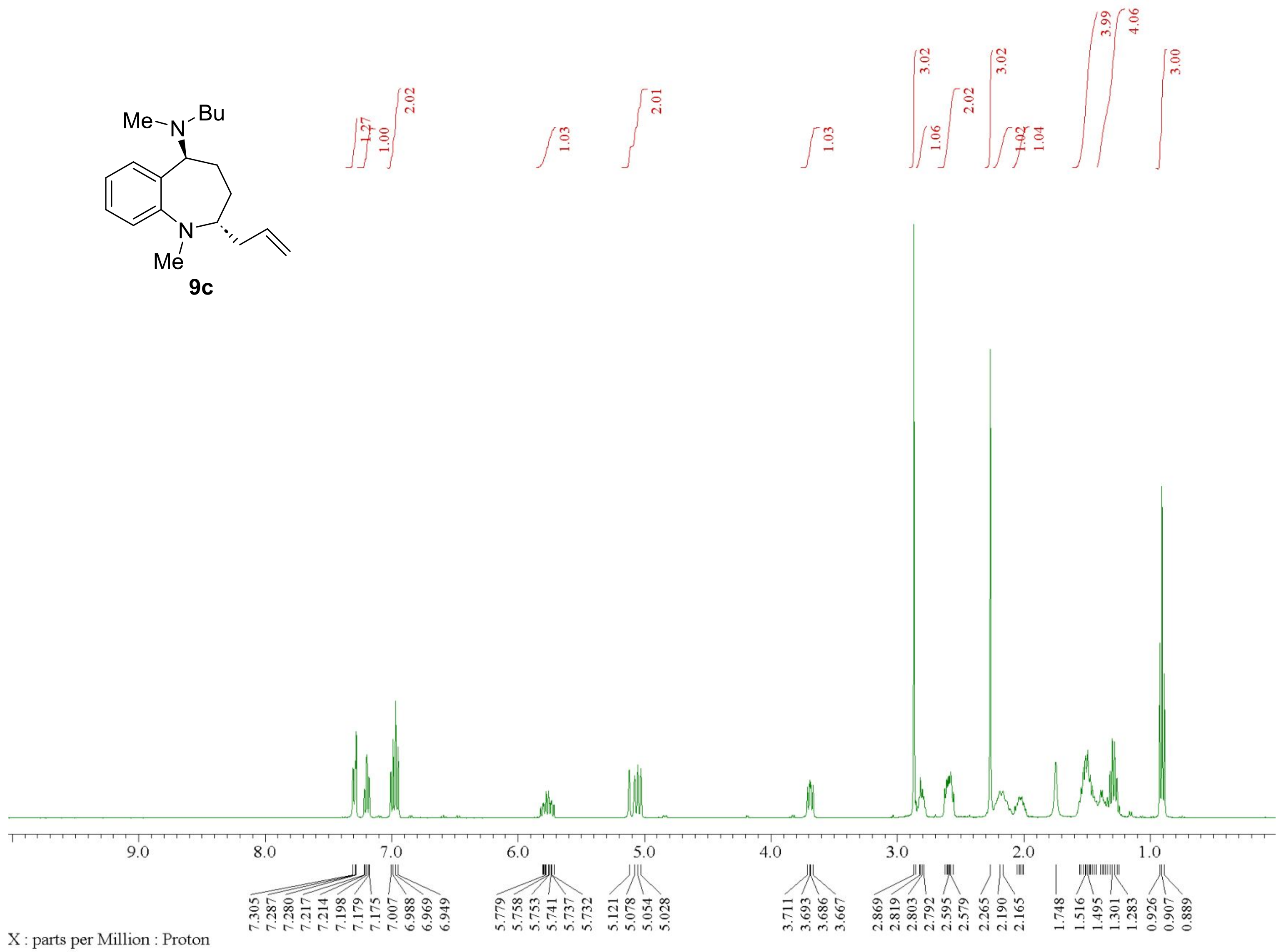

^1H NMR, 400 MHz, $CDCl_3$

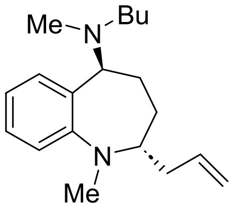

9c

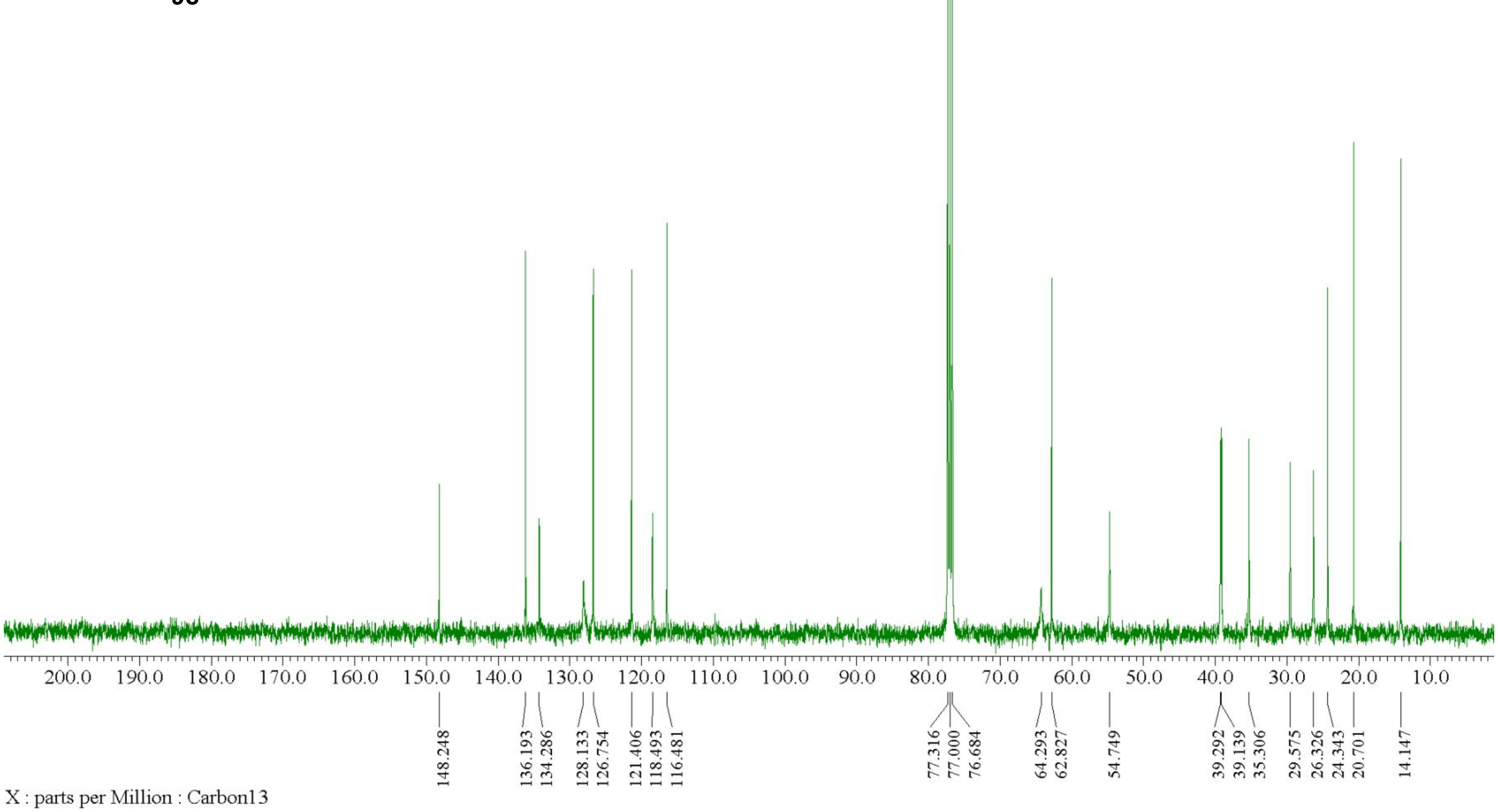

$^{13}C\{1H\}$ NMR, 100 MHz, $CDCl_3$

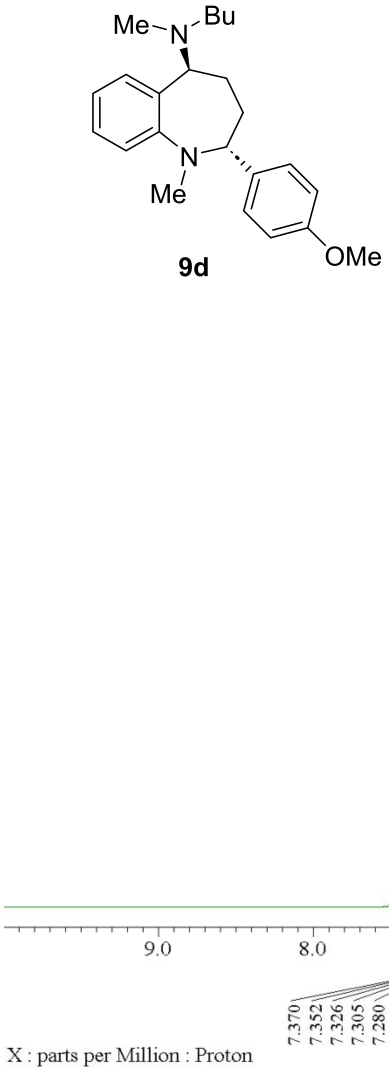

9d

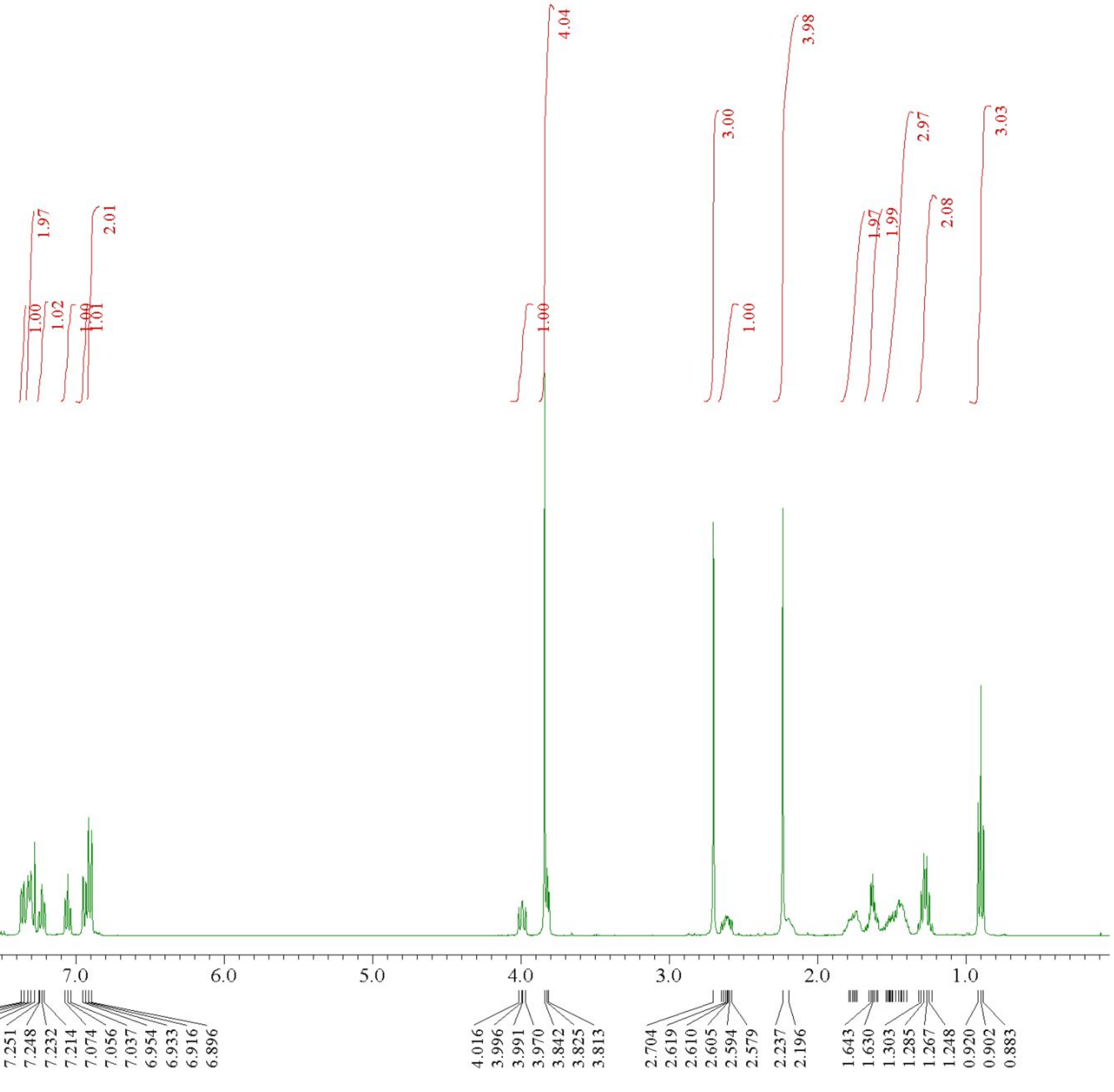

^1H NMR, 400 MHz, $CDCl_3$

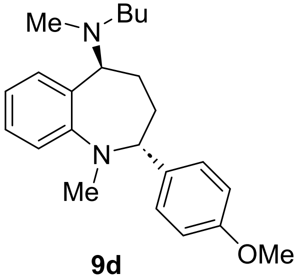

Me
N
Bu
N
Me
OMe

9d

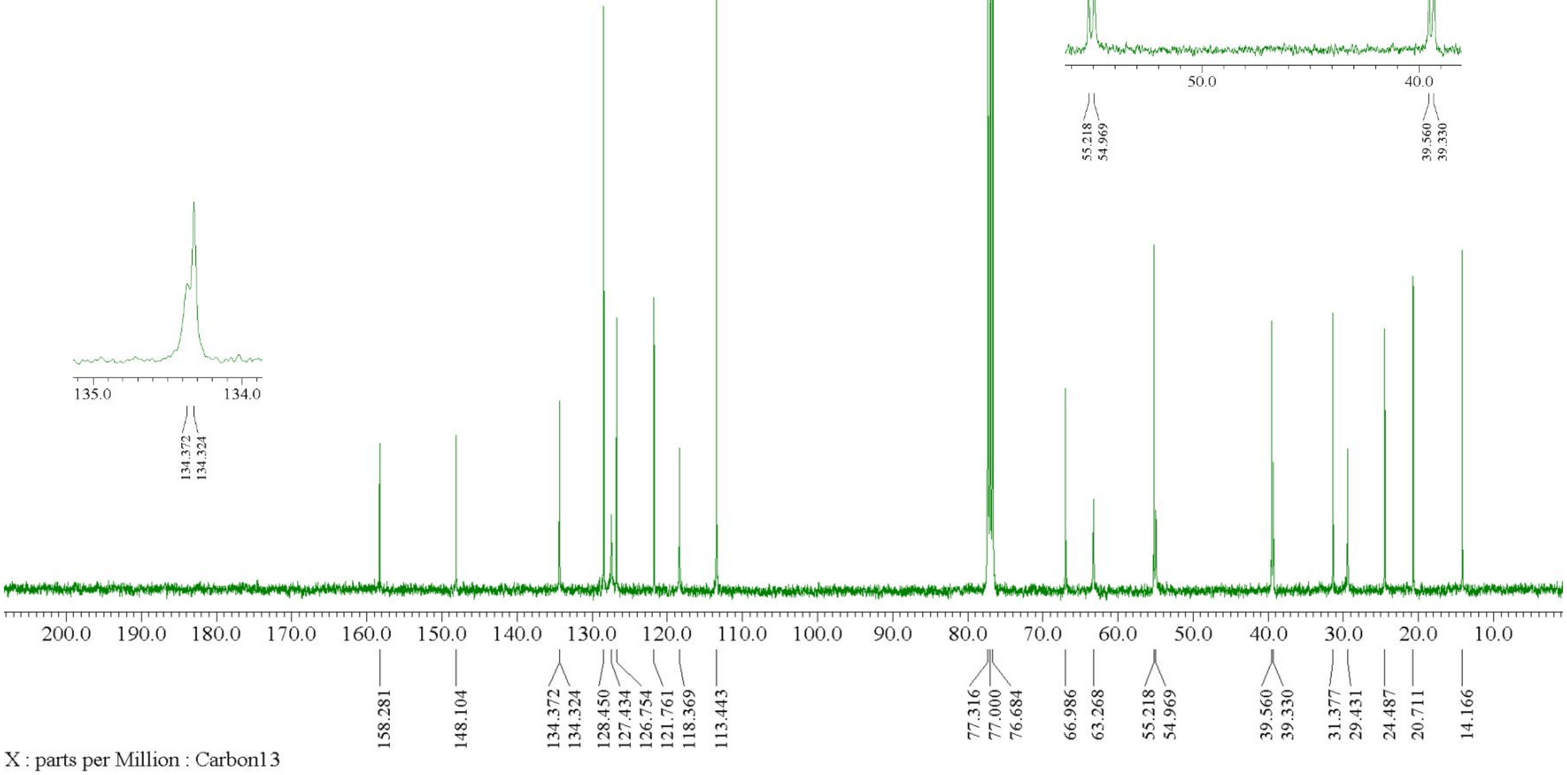

135.0
134.0
134.372
134.324
50.0
40.0
55.218
54.969
39.560
39.330
200.0 190.0 180.0 170.0 160.0 150.0 140.0 130.0 120.0 110.0 100.0 90.0 80.0 70.0 60.0 50.0 40.0 30.0 20.0 10.0
158.281
148.104
134.372
134.324
128.450
127.434
126.754
121.761
118.369
113.443
77.316
77.000
76.684
66.986
63.268
55.218
54.969
39.560
39.330
31.377
29.431
24.487
20.711
14.166
X : parts per Million : Carbon13

$^{13}C\{1H\}$ NMR, 100 MHz, $CDCl_3$

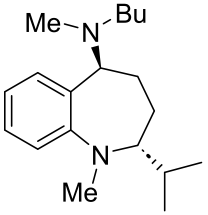

9e

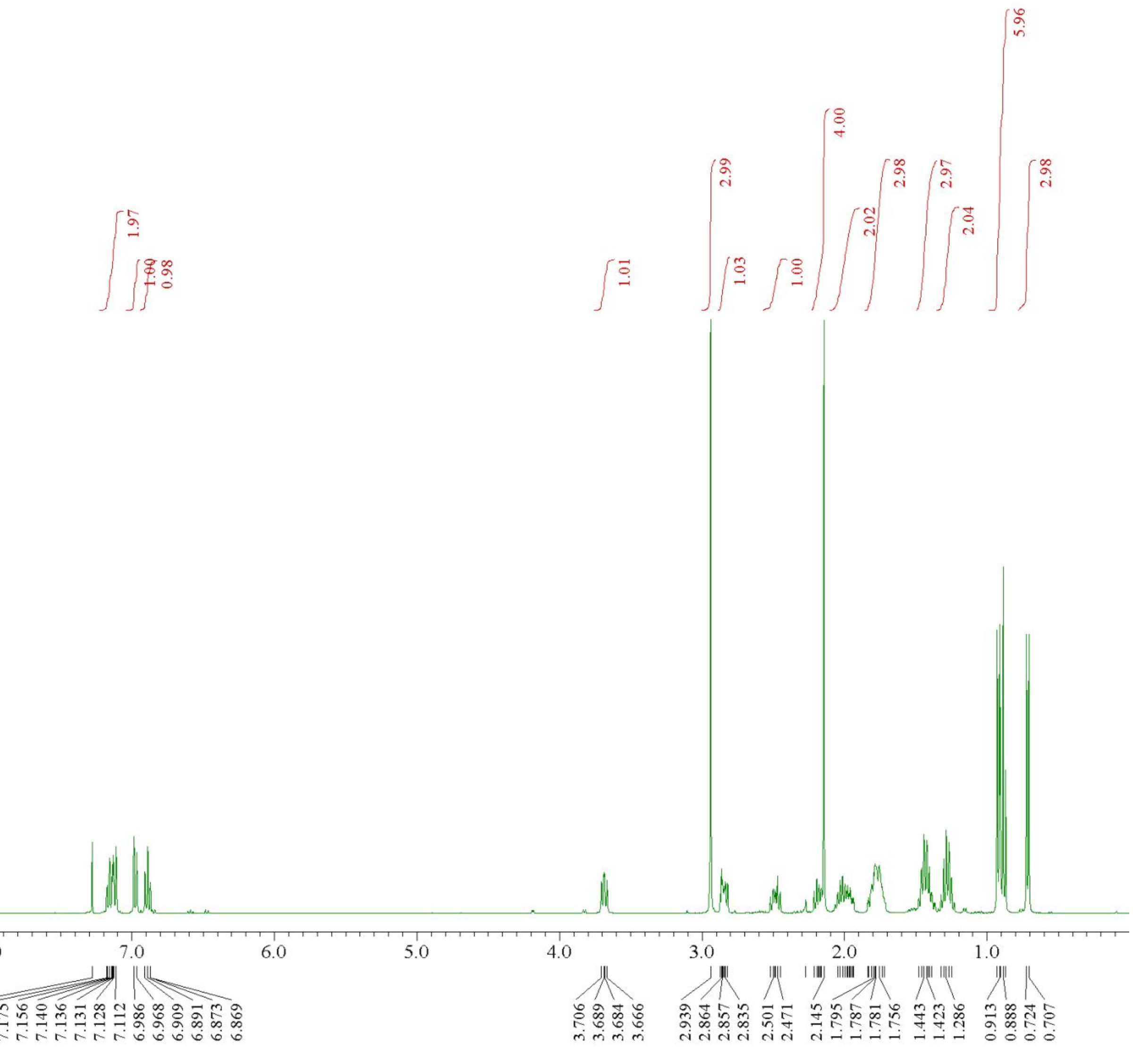

^1H NMR, 400 MHz, $CDCl_3$

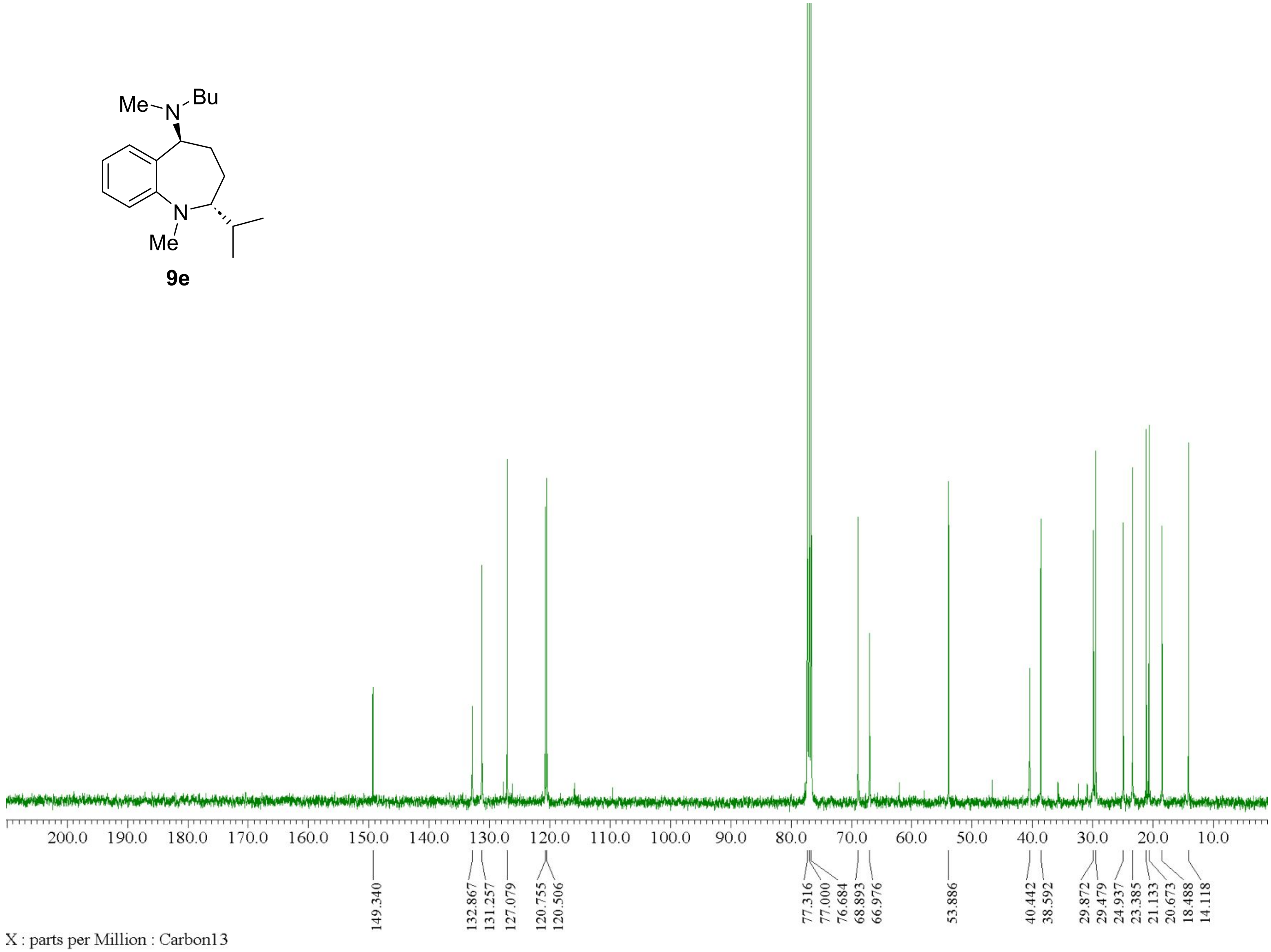

9e

$^{13}C\{1H\}$ NMR, 100 MHz, $CDCl_3$

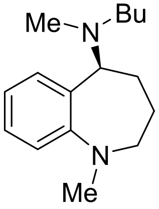

9f

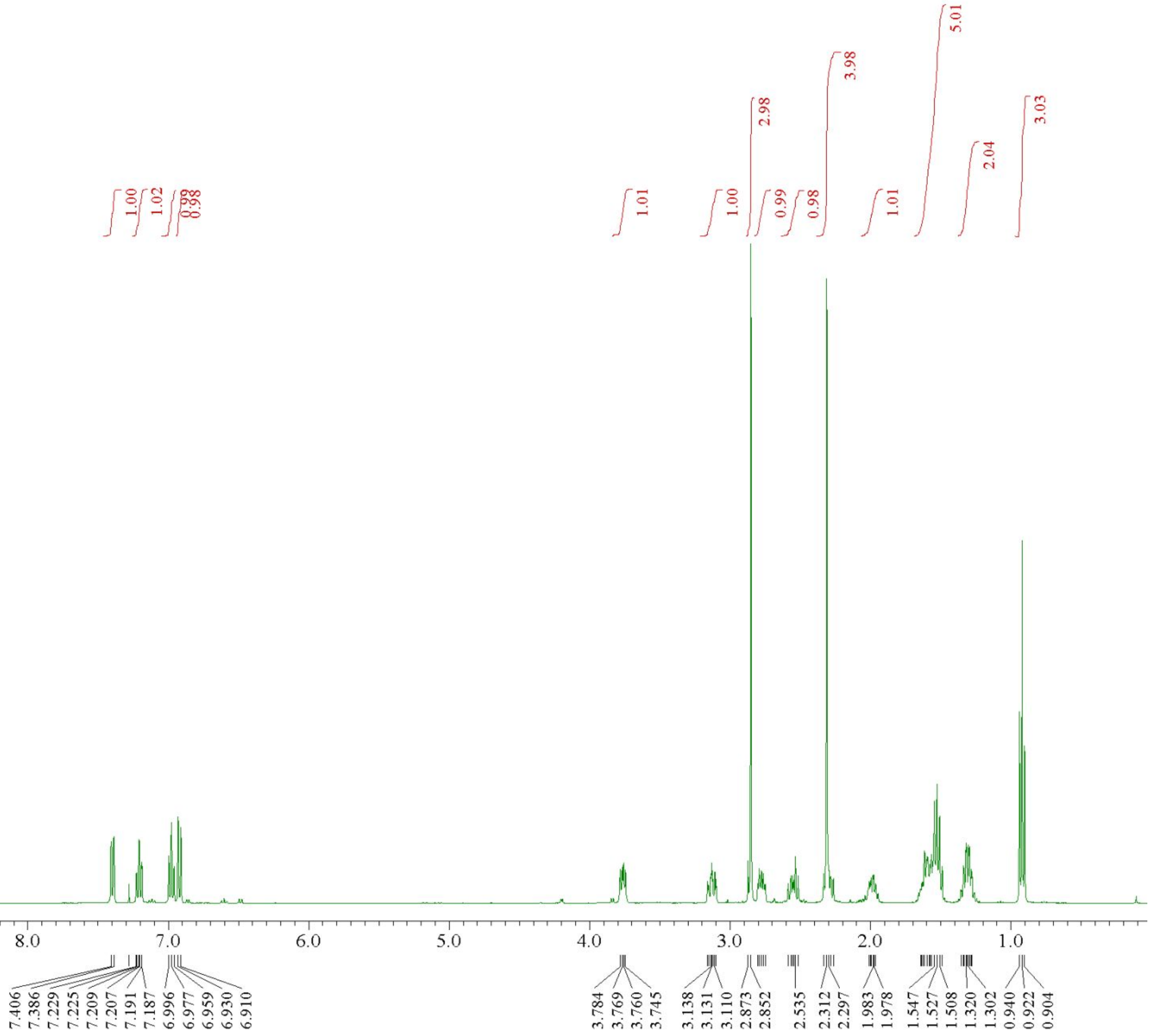

^1H NMR, 400 MHz, $CDCl_3$

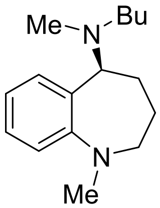

9f

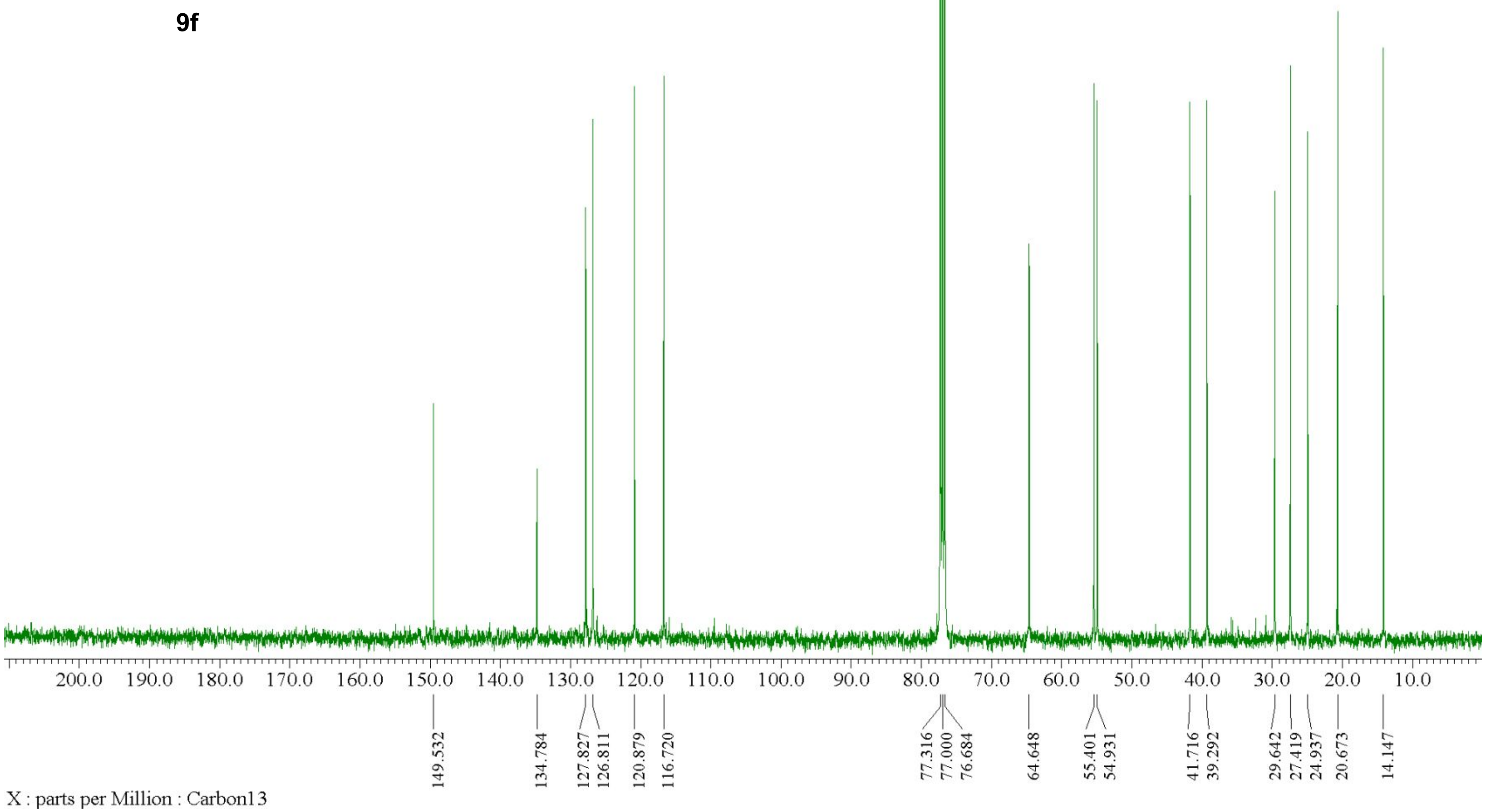

$^{13}C\{1H\}$ NMR, 100 MHz, $CDCl_3$

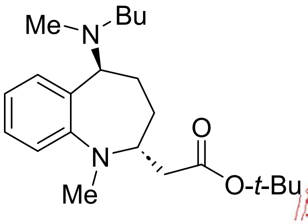

9g

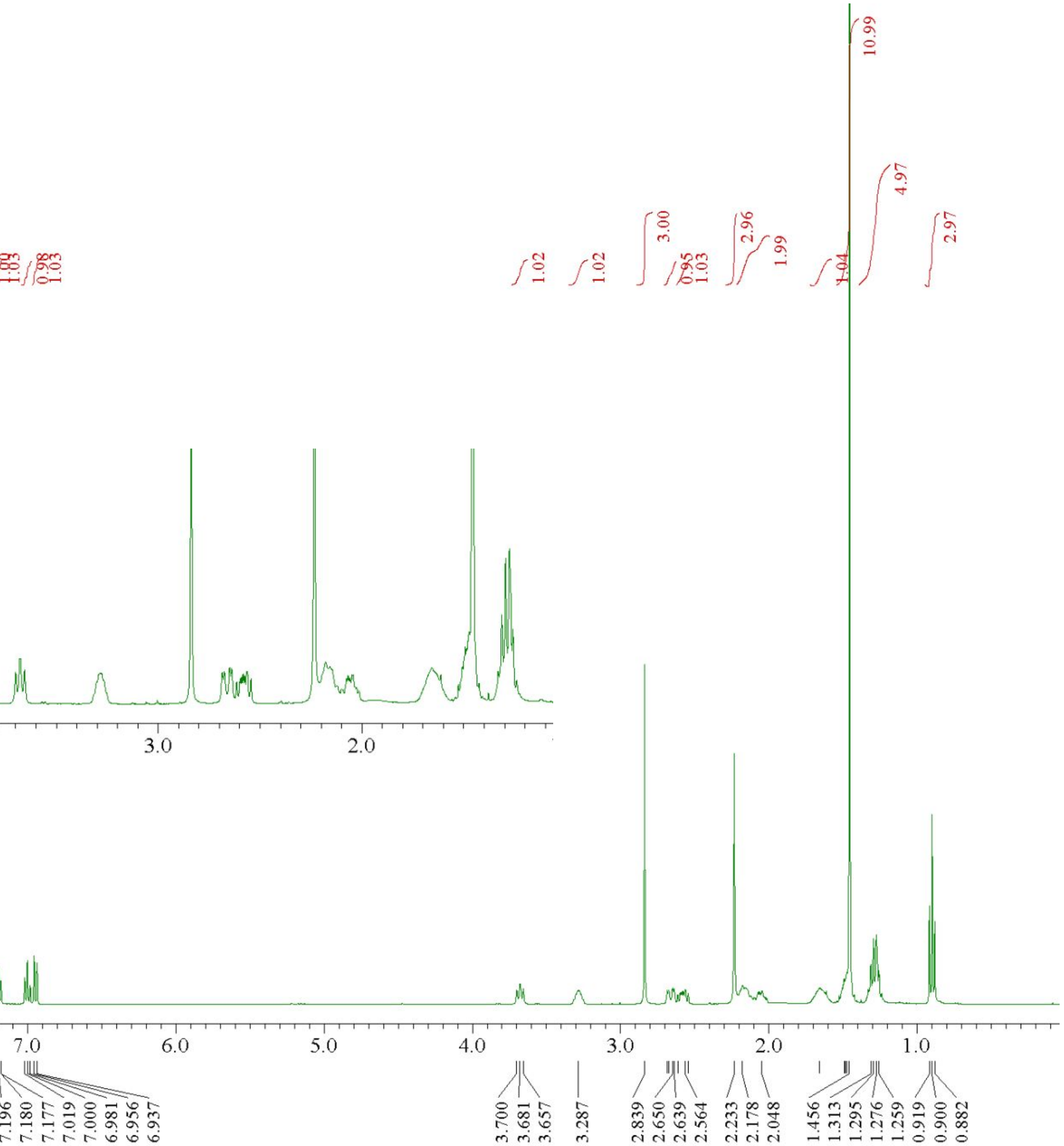

^{1}H NMR, 400 MHz, $CDCl_3$

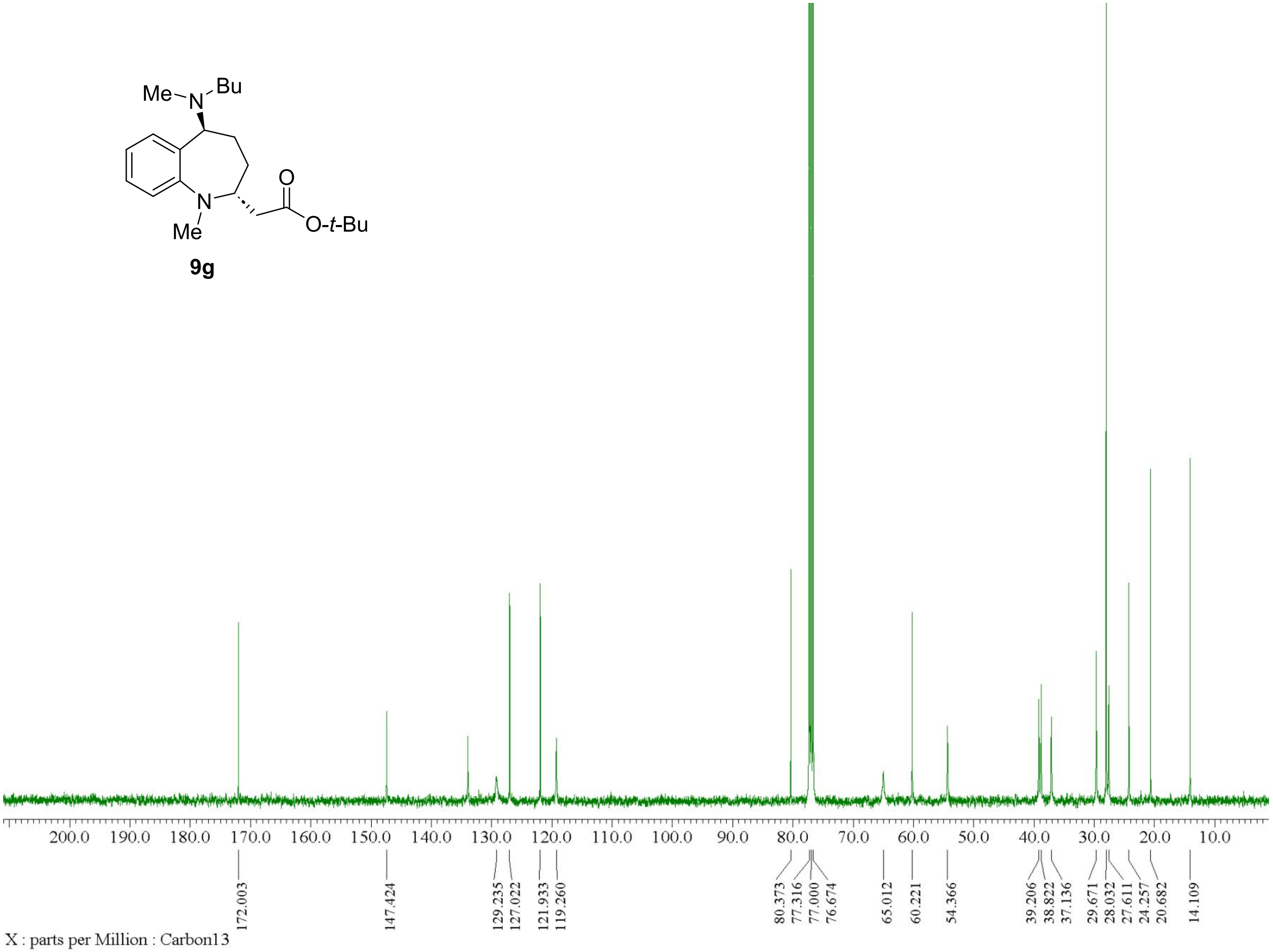

$^{13}C\{1H\}$ NMR, 100 MHz, $CDCl_3$

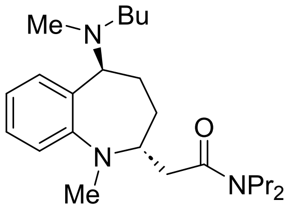

9h

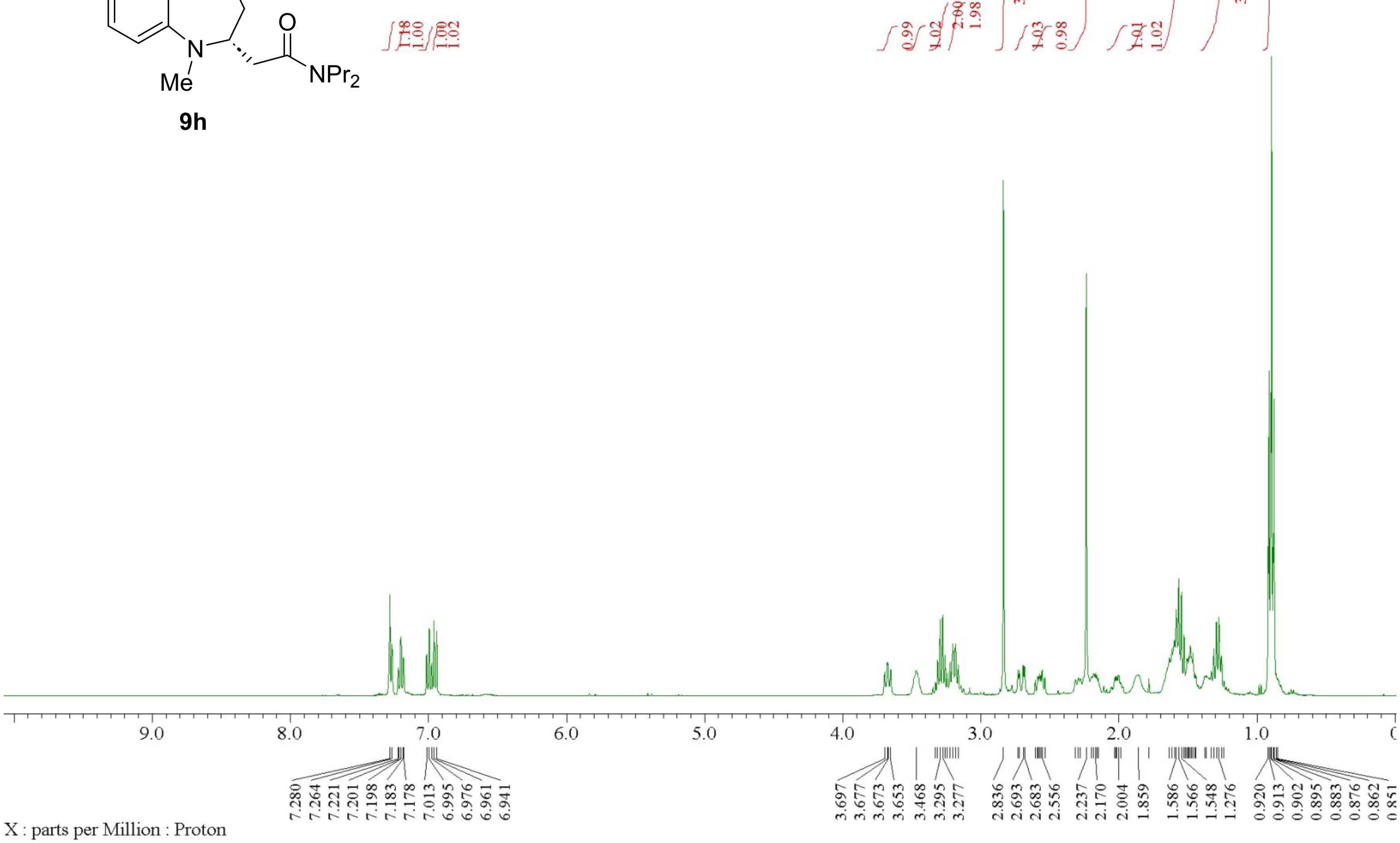

^{1}H NMR, 400 MHz, $CDCl_3$

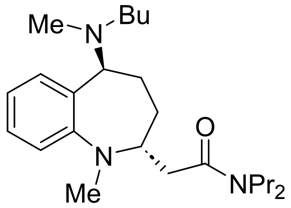

9h

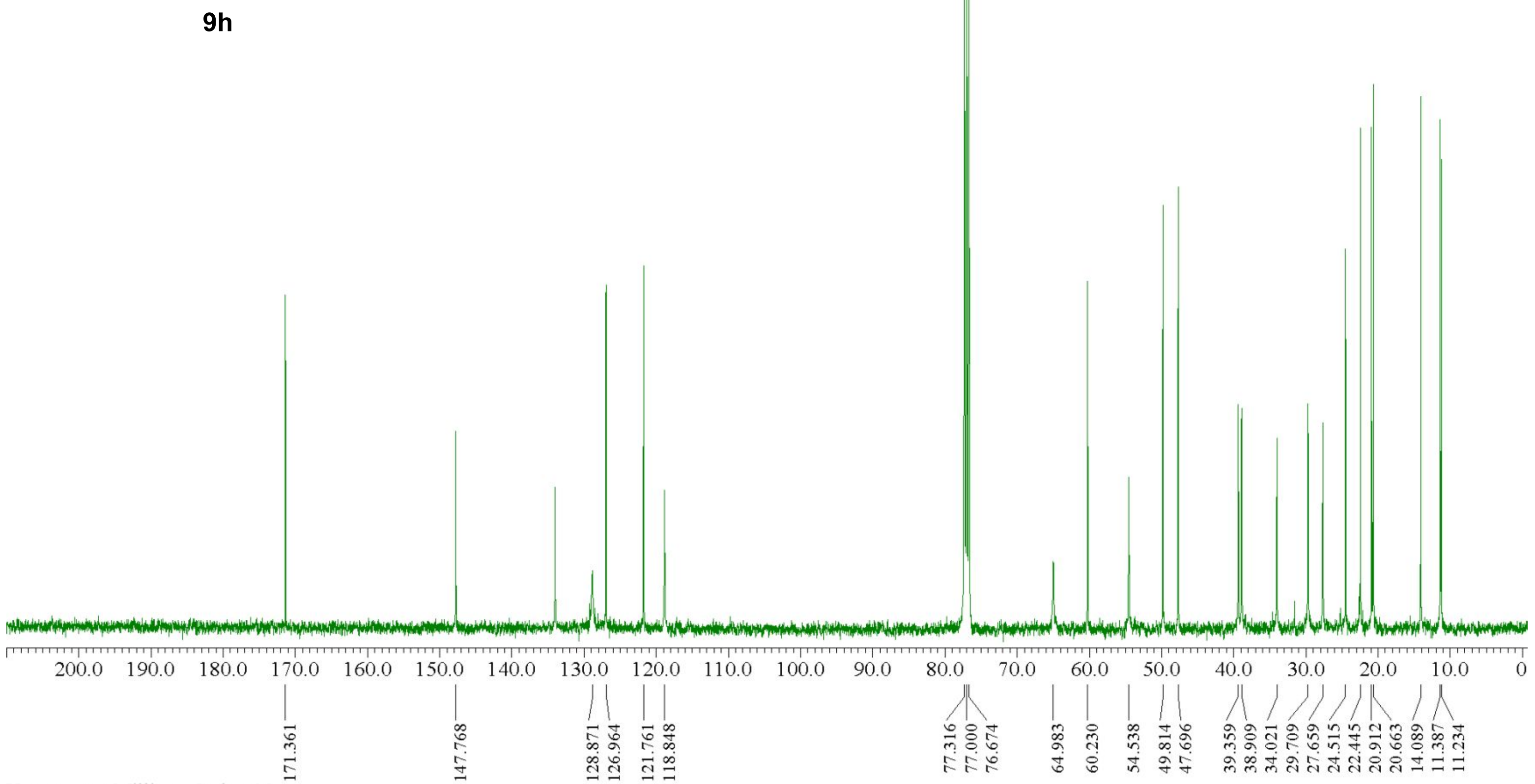

^{13}C{1H} NMR, 100 MHz, $CDCl_3$

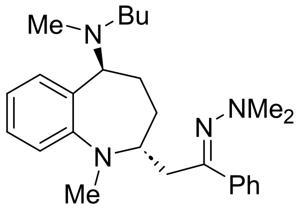

9i

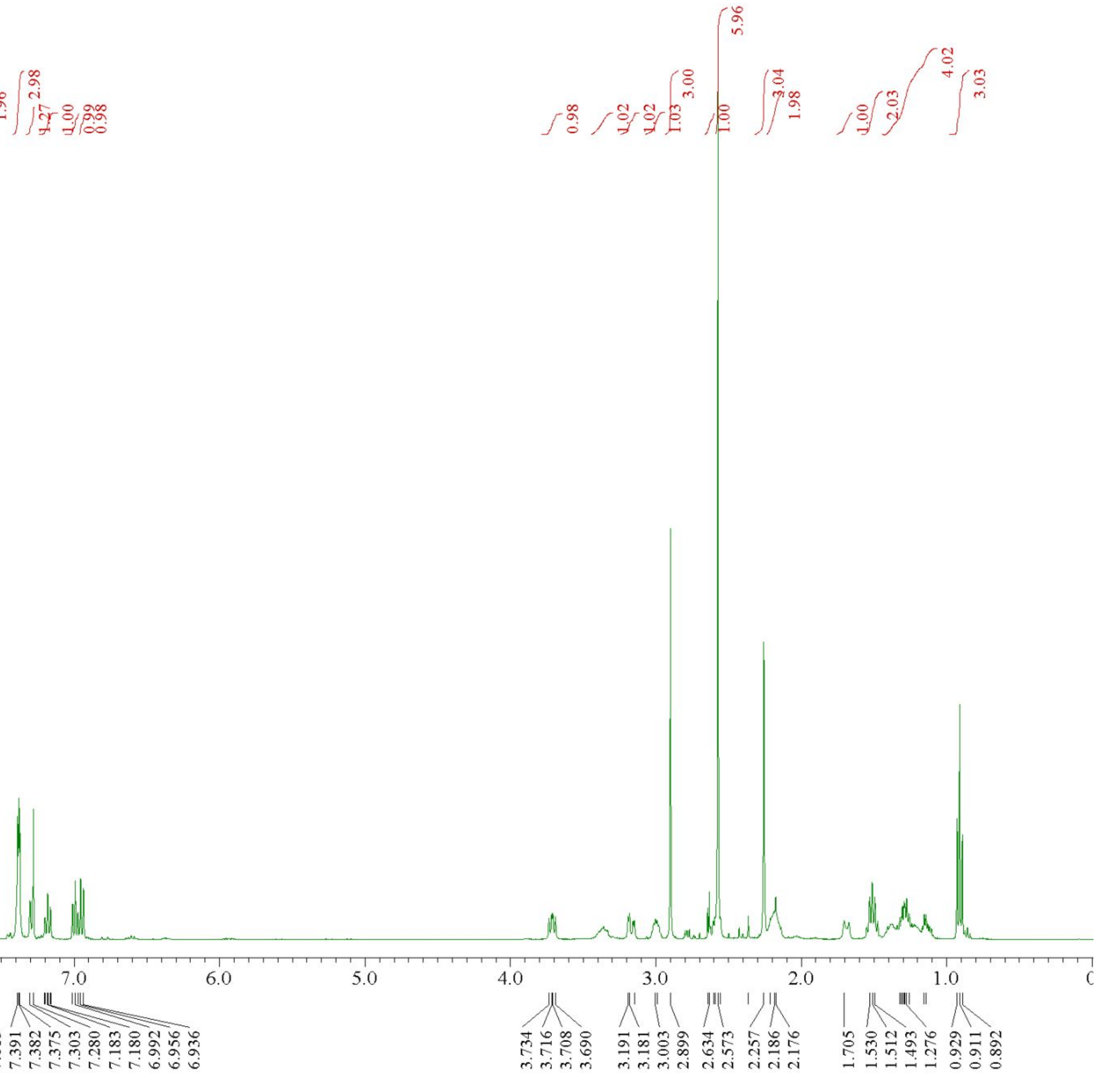

^{1}H NMR, 400 MHz, $CDCl_3$

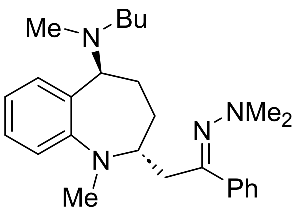

9i

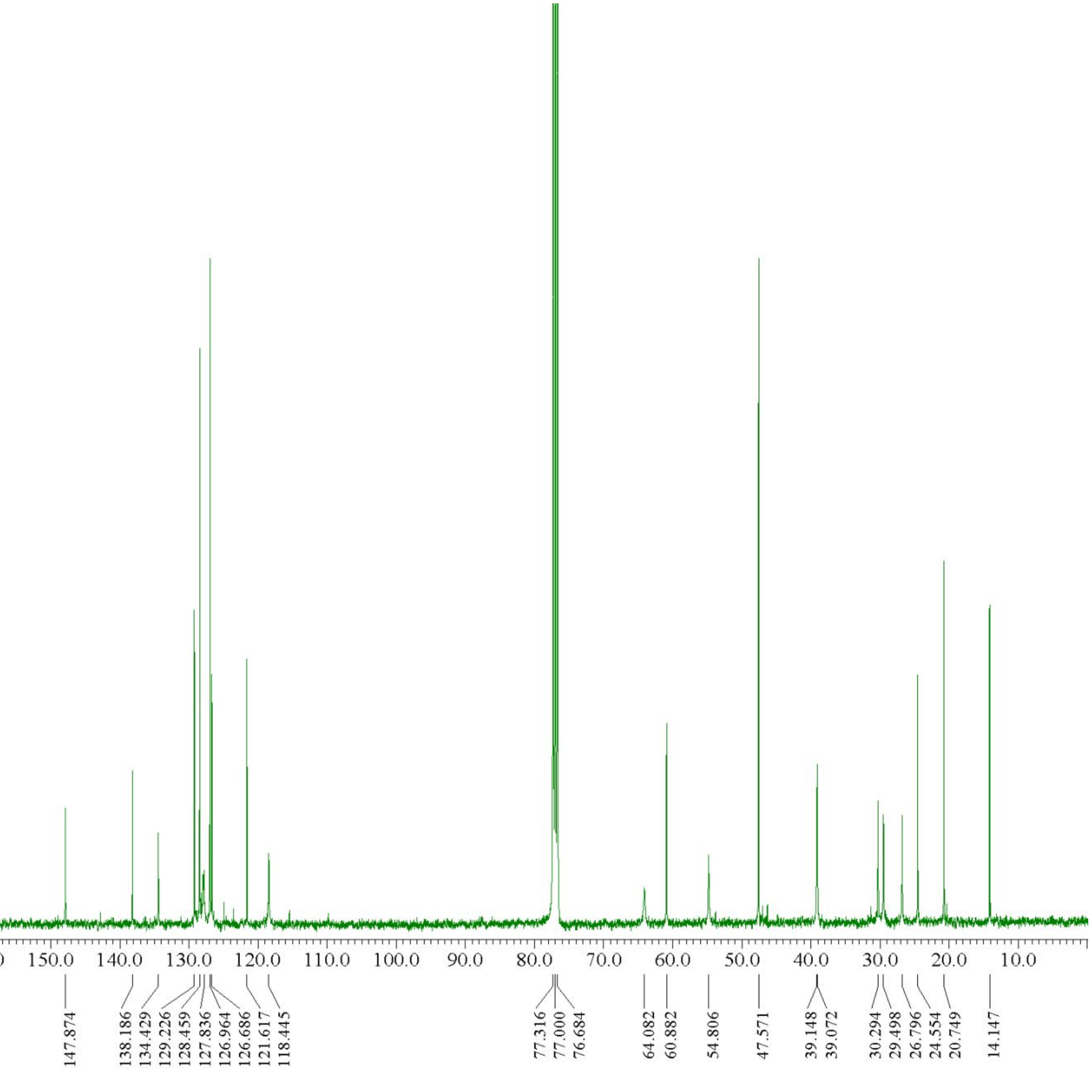

$^{13}C\{^1H\}$ NMR, 100 MHz, $CDCl_3$

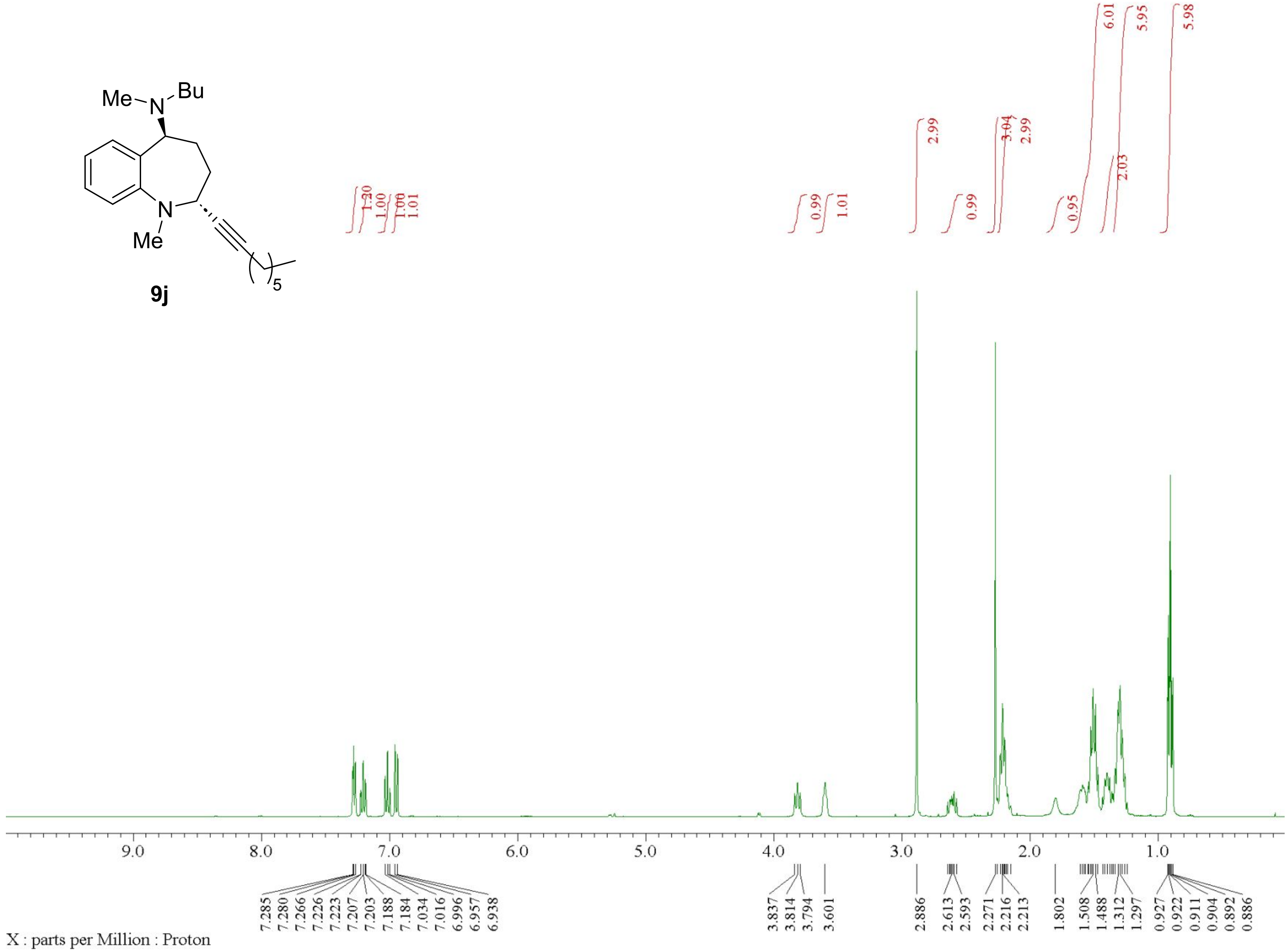

^{1}H NMR, 400 MHz, $CDCl_3$

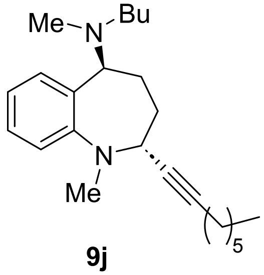

9j

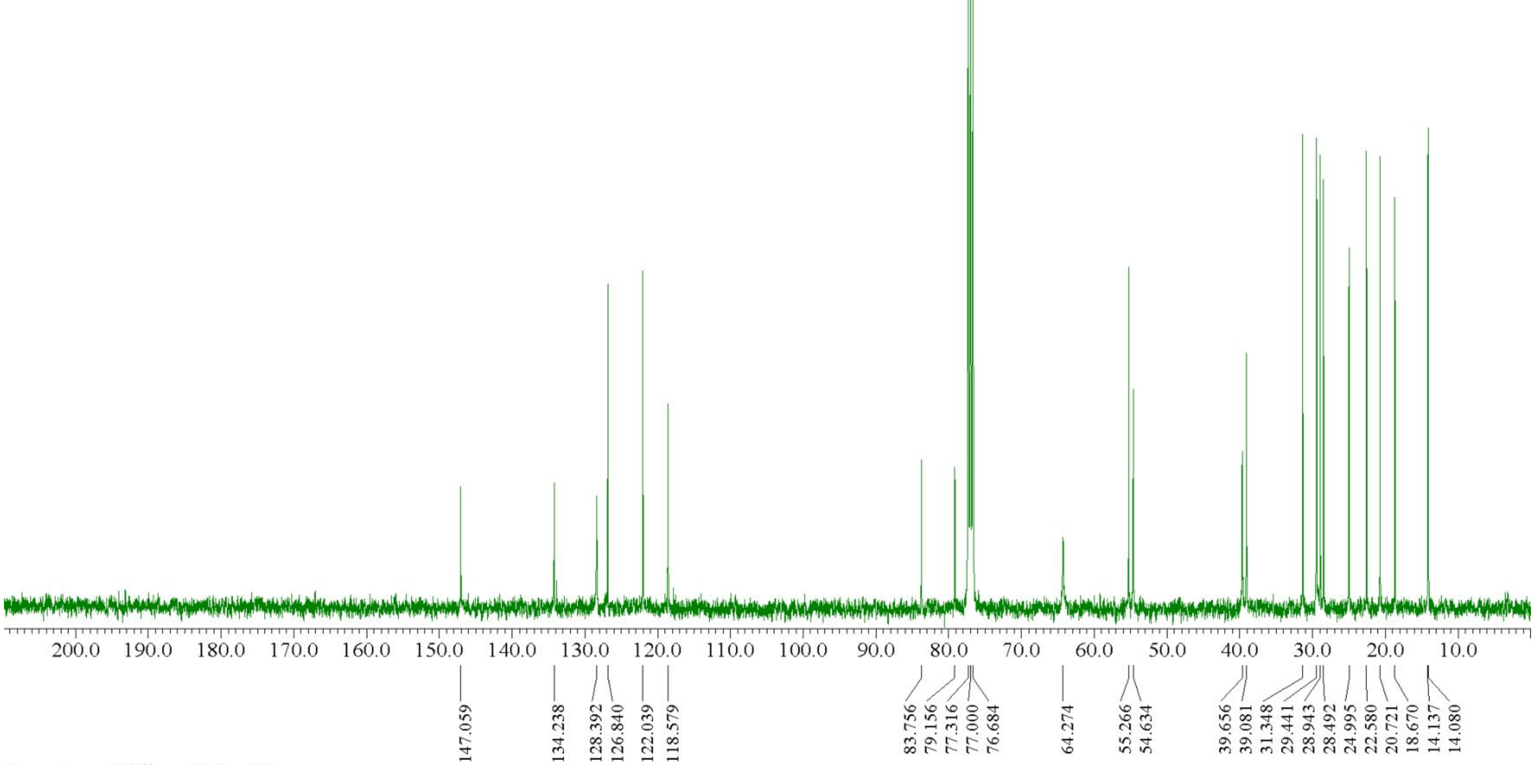

X : parts per Million : Carbon13

$^{13}C\{1H\}$ NMR, 100 MHz, $CDCl_3$

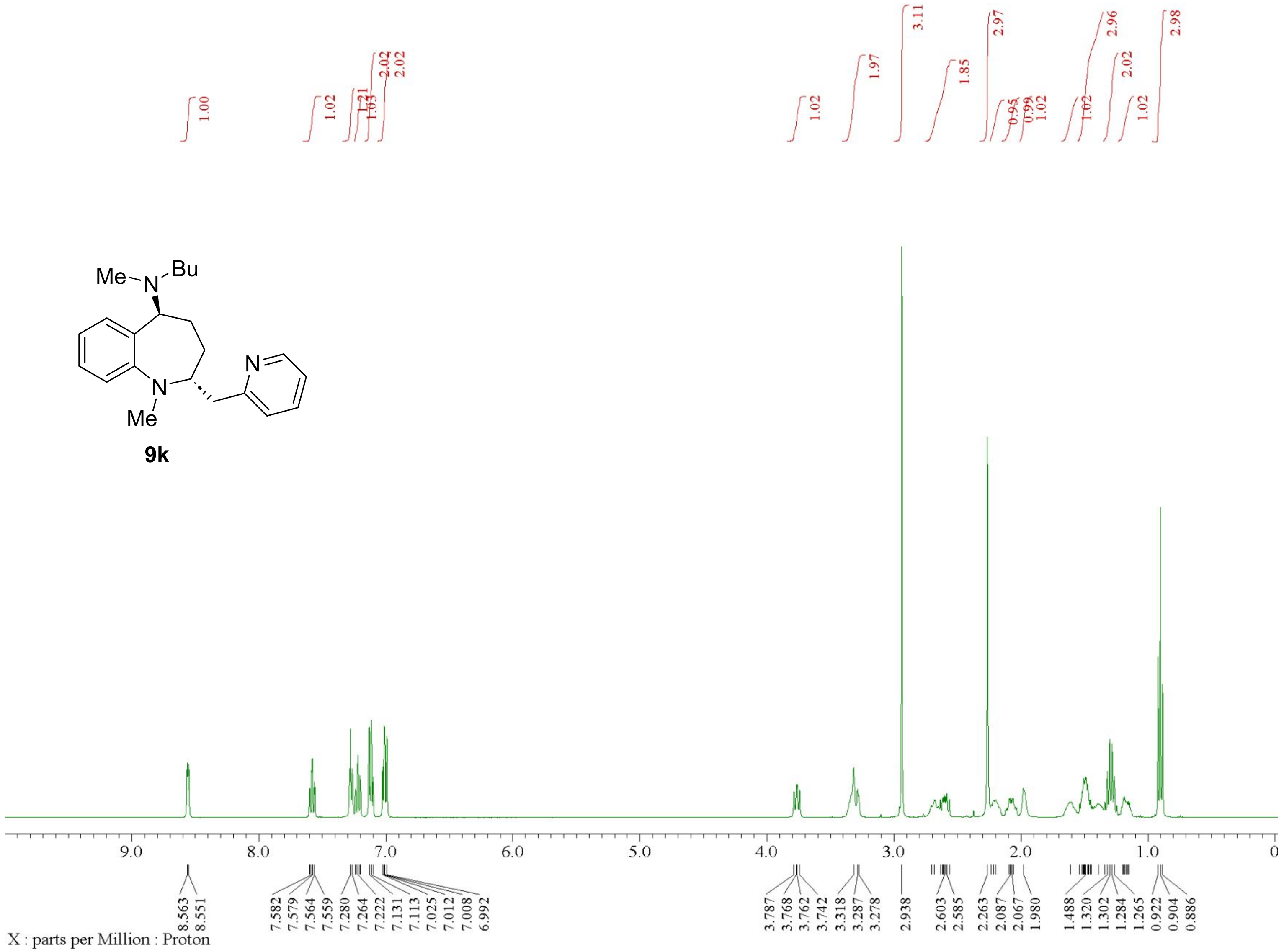

^{1}H NMR, 400 MHz, $CDCl_3$

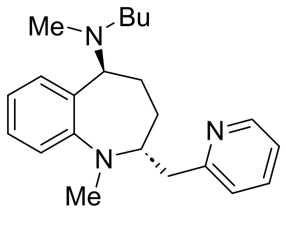

9k

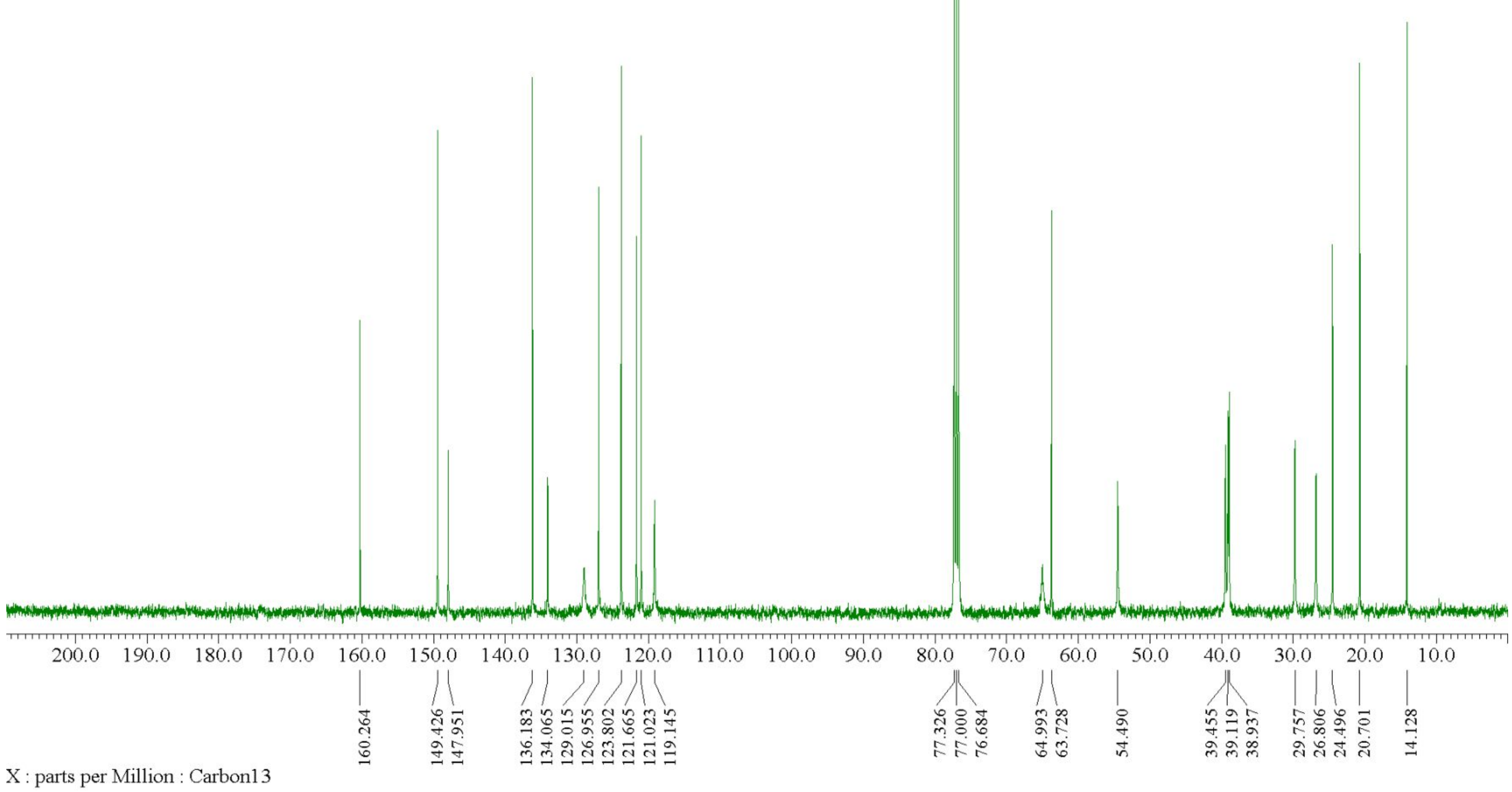

$^{13}C\{^1H\}$ NMR, 100 MHz, $CDCl_3$

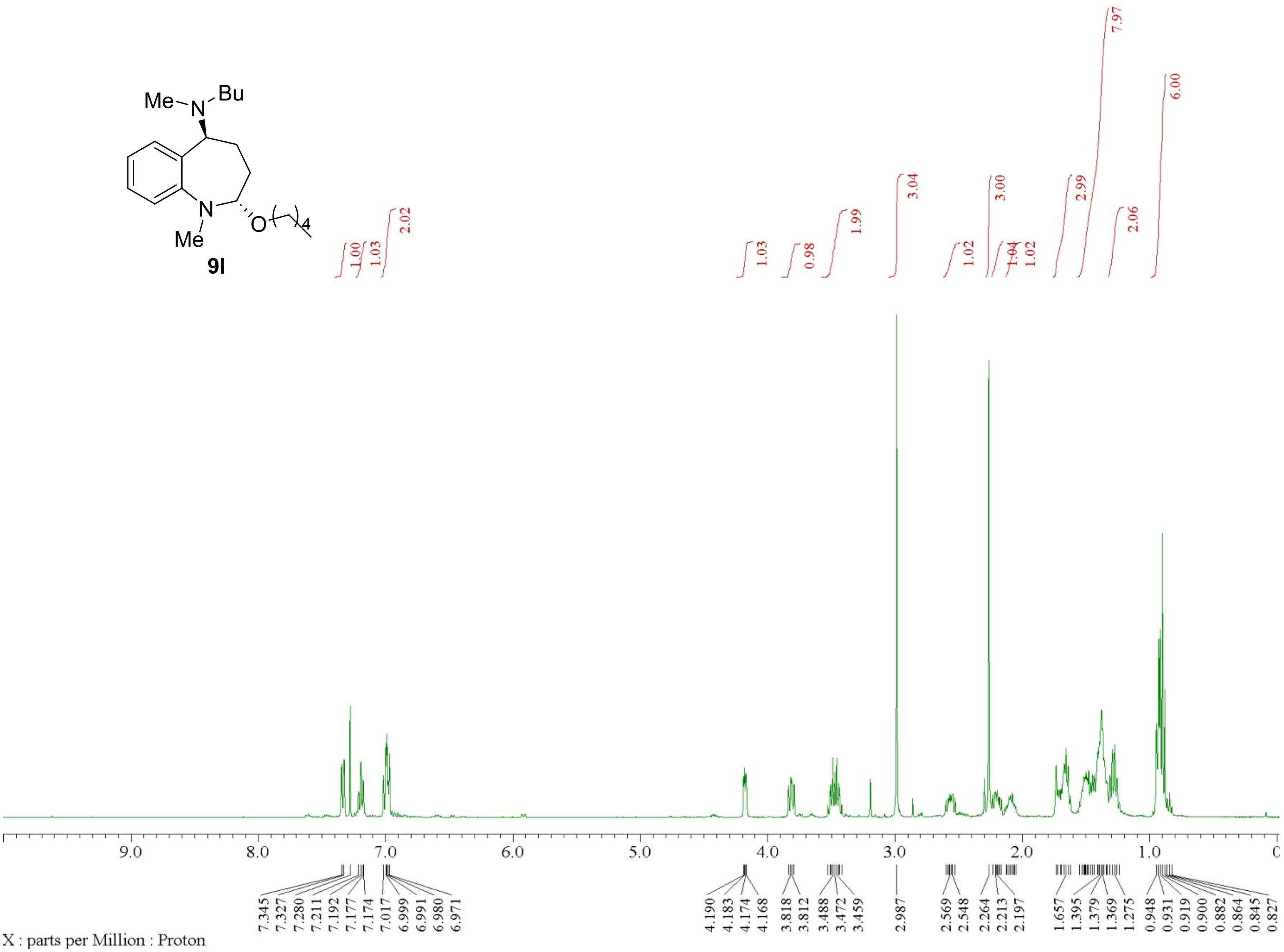

^{1}H NMR, 400 MHz, $CDCl_3$

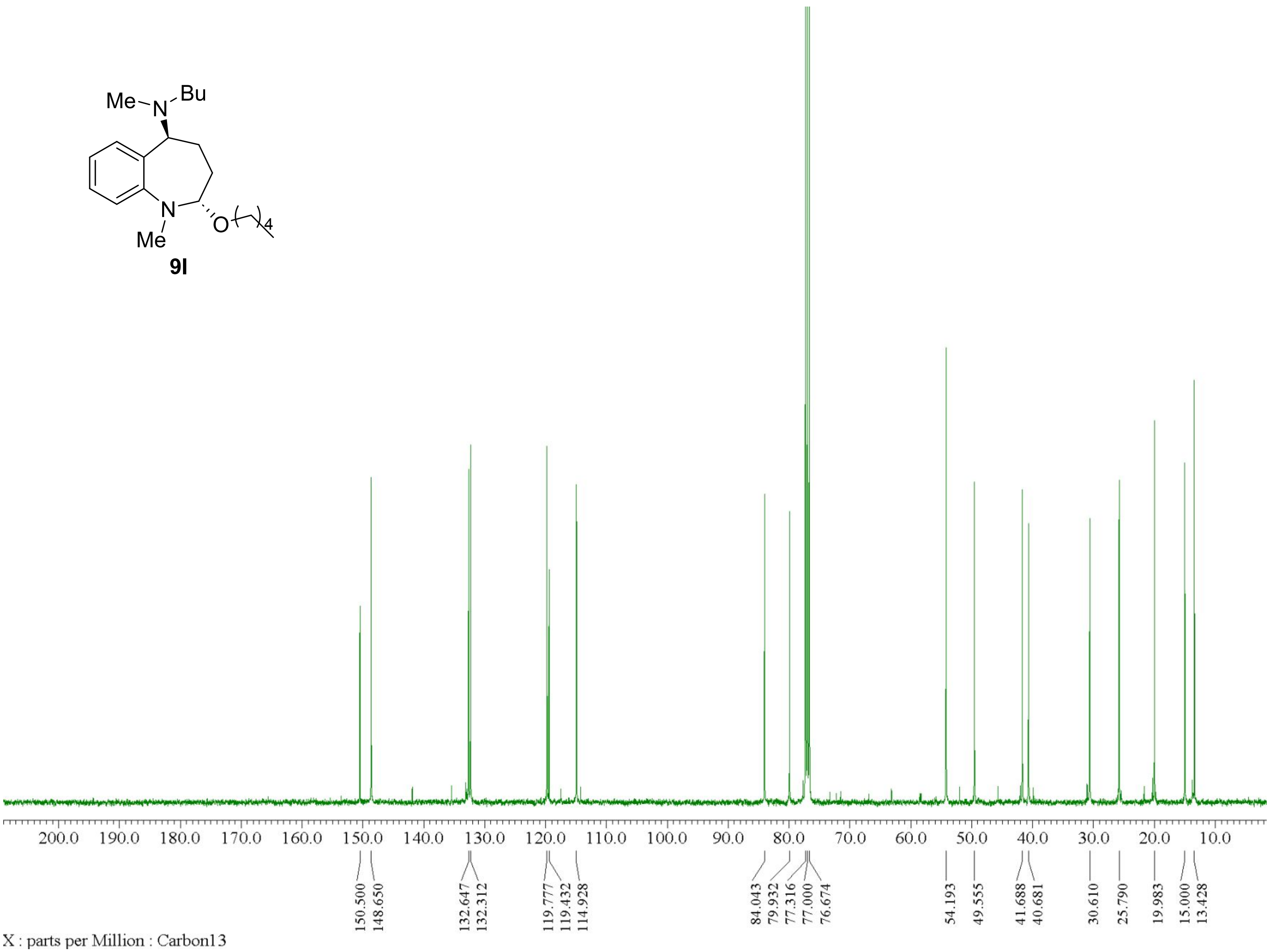

$^{13}C\{1H\}$ NMR, 100 MHz, $CDCl_3$

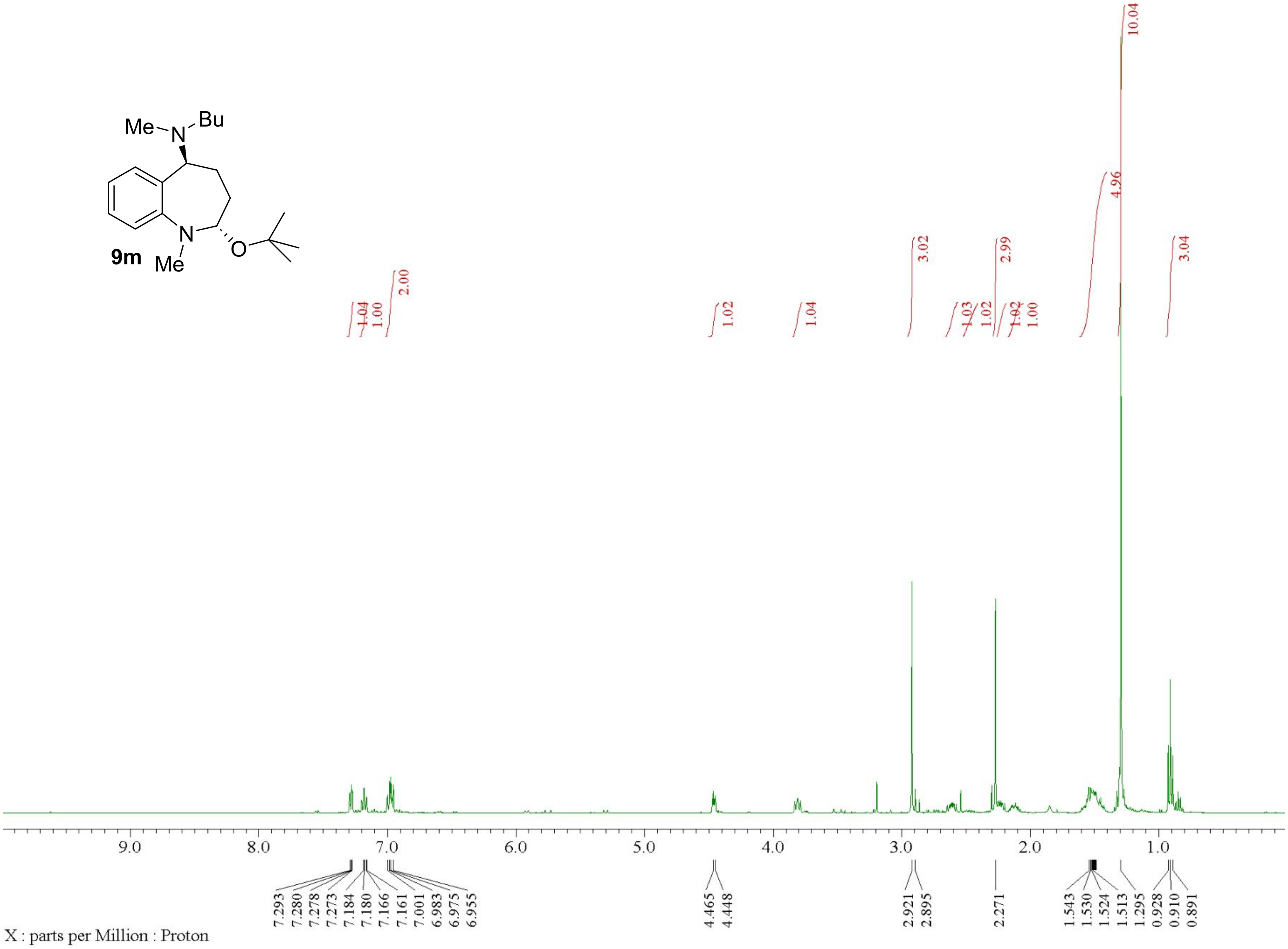

^{1}H NMR, 400 MHz, $CDCl_3$

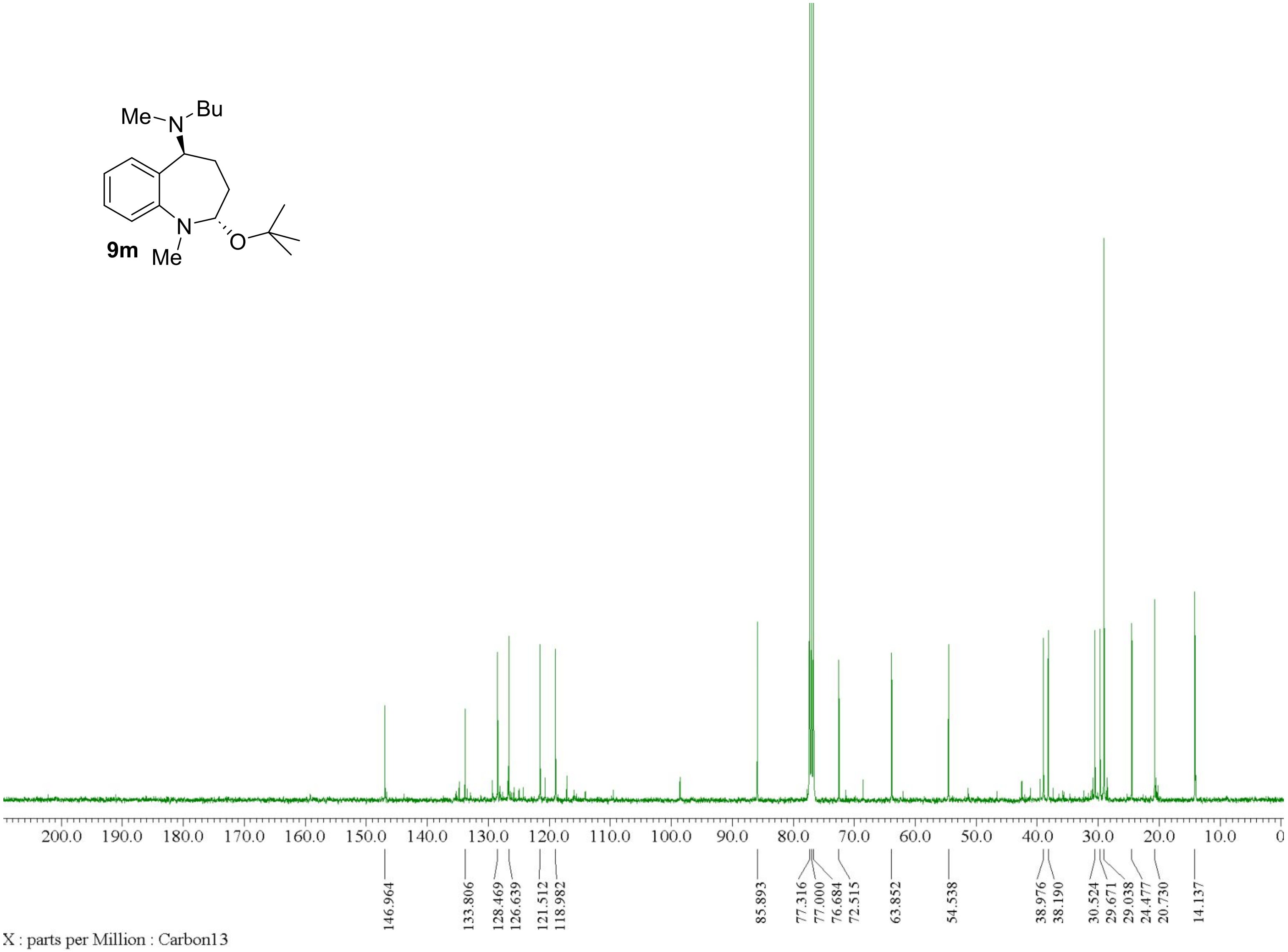

$^{13}C\{^1H\}$ NMR, 100 MHz, $CDCl_3$

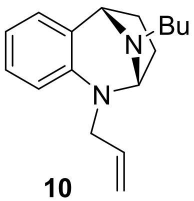

10

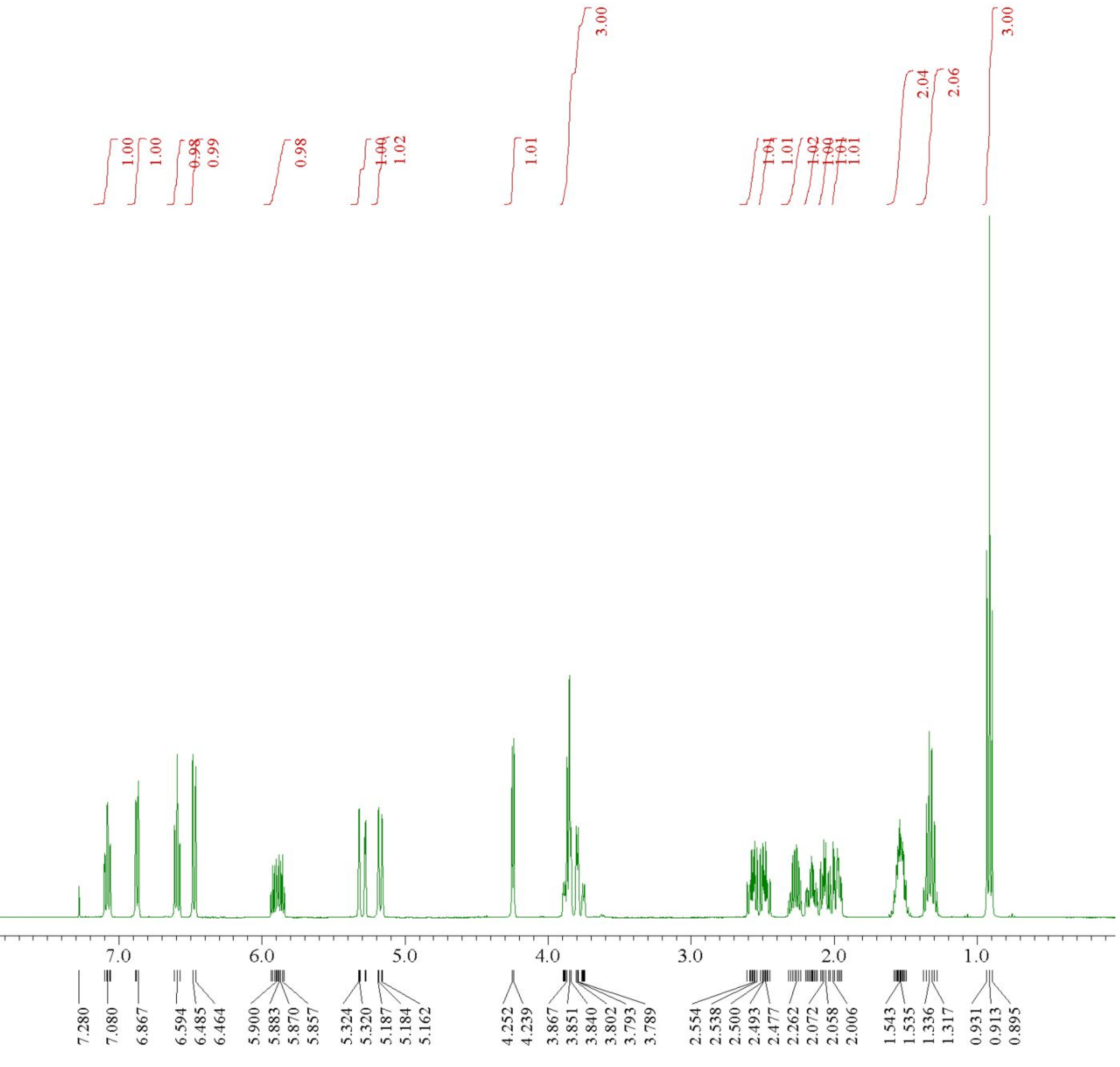

^{1}H NMR, 400 MHz, $CDCl_3$

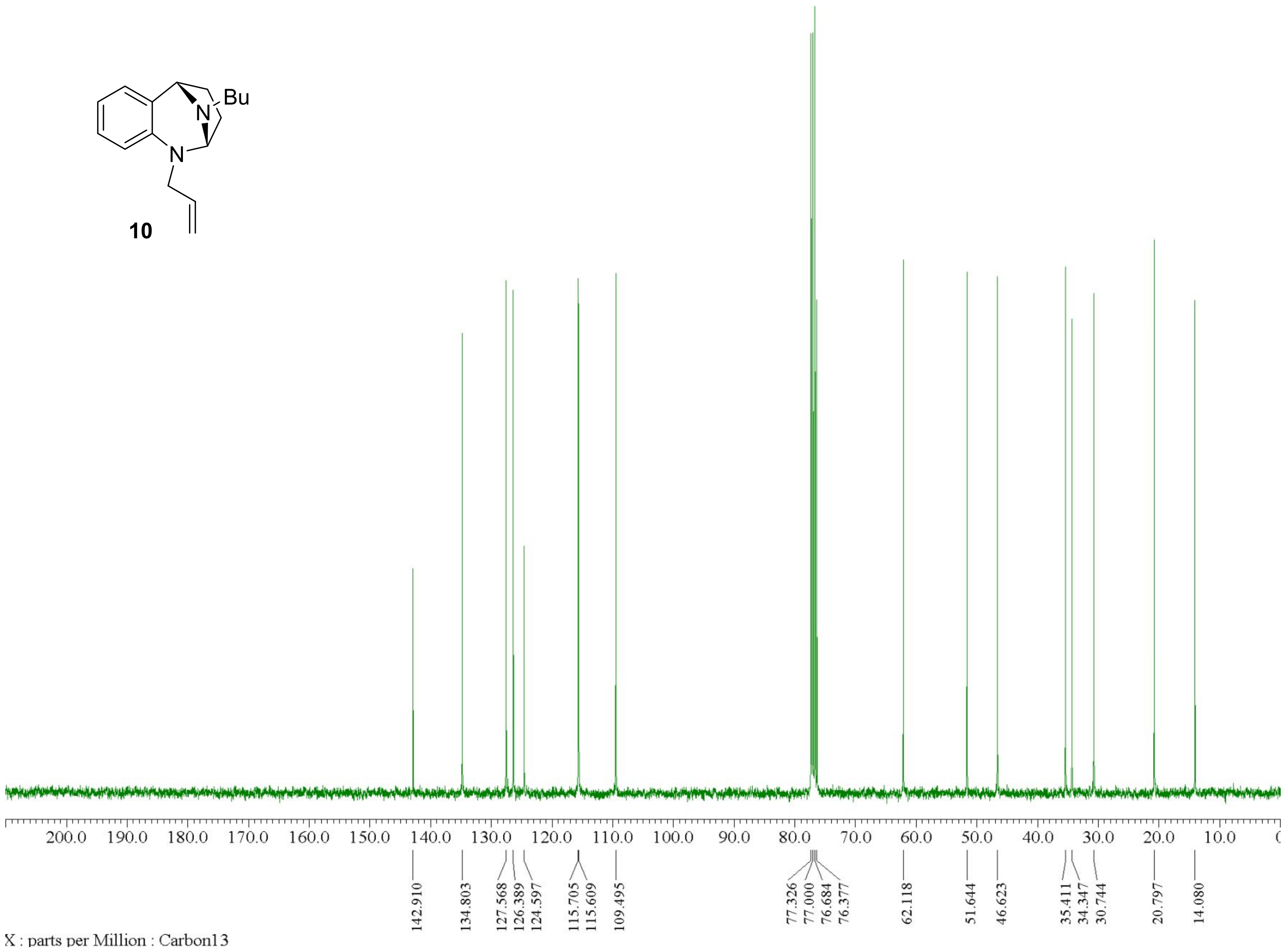

$^{13}C\{1H\}$ NMR, 100 MHz, $CDCl_3$

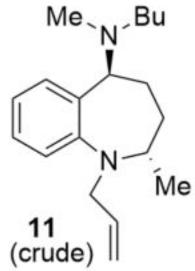

11
(crude)

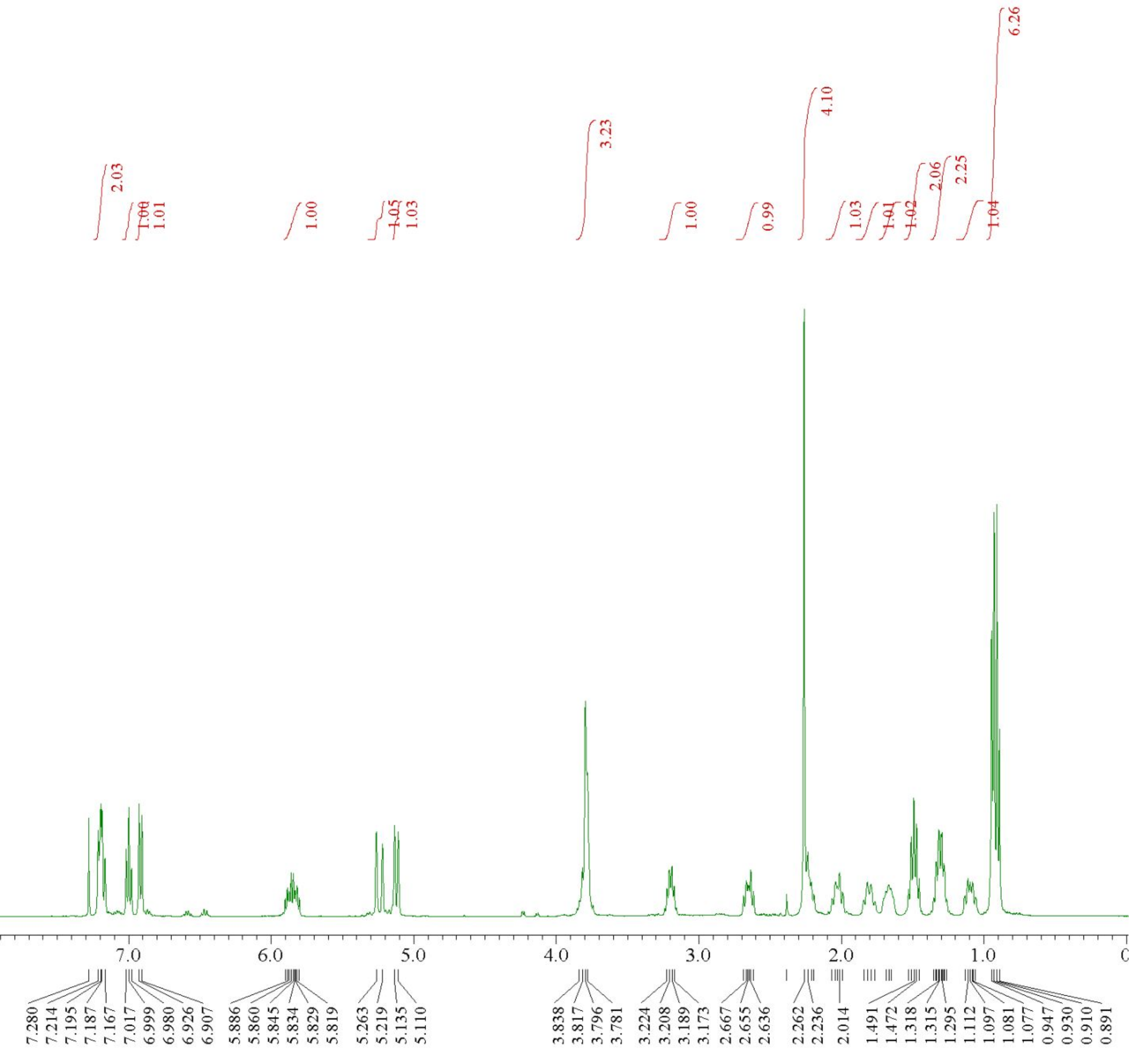

^{1}H NMR, 400 MHz, $CDCl_3$

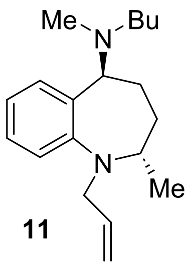

11

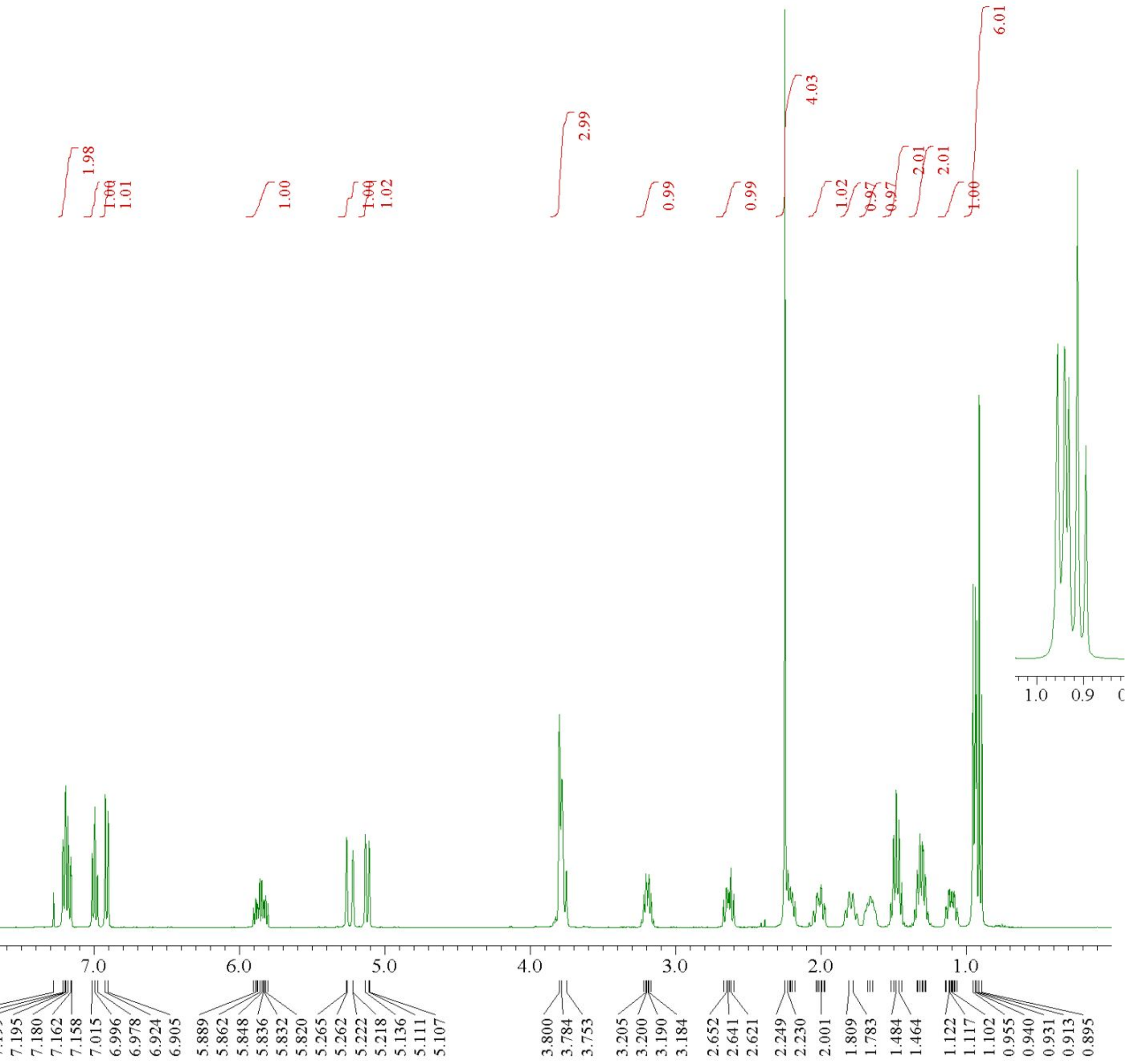

^{1}H NMR, 400 MHz, $CDCl_3$

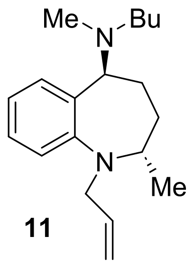

11

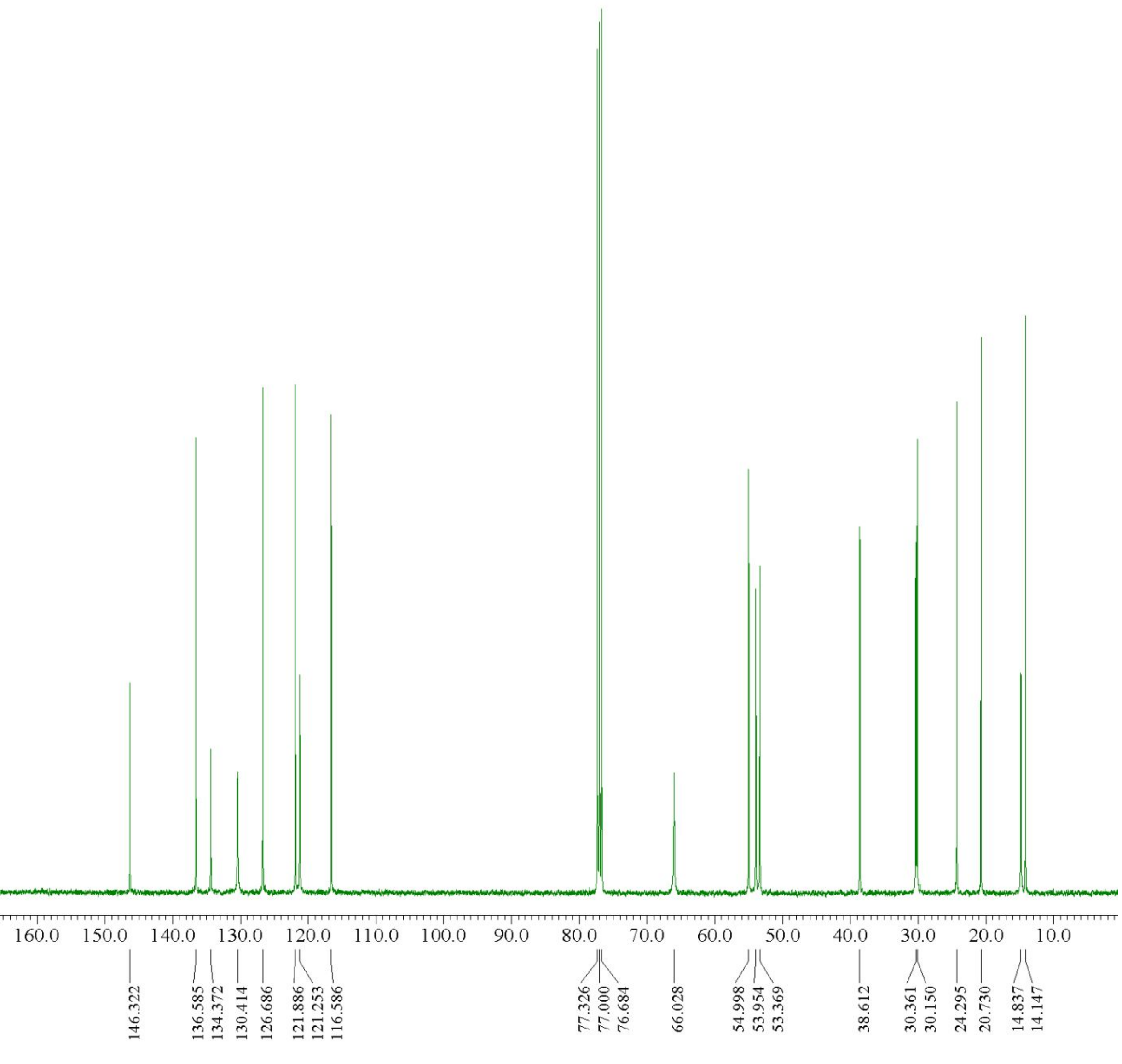

$^{13}C\{1H\}$ NMR, 100 MHz, $CDCl_3$

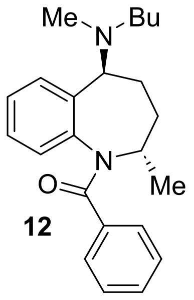

12

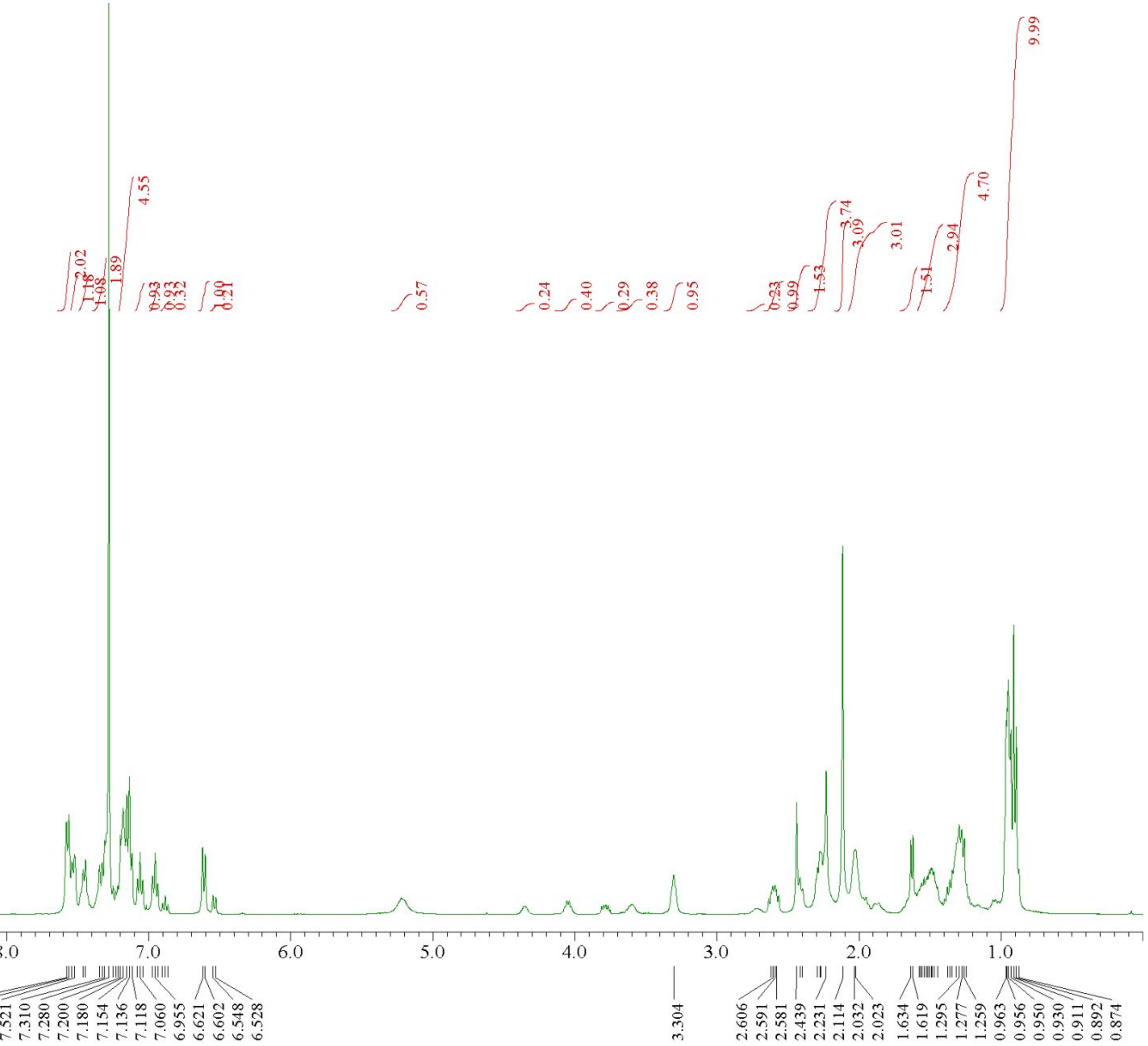

^{1}H NMR, 400 MHz, $CDCl_3$

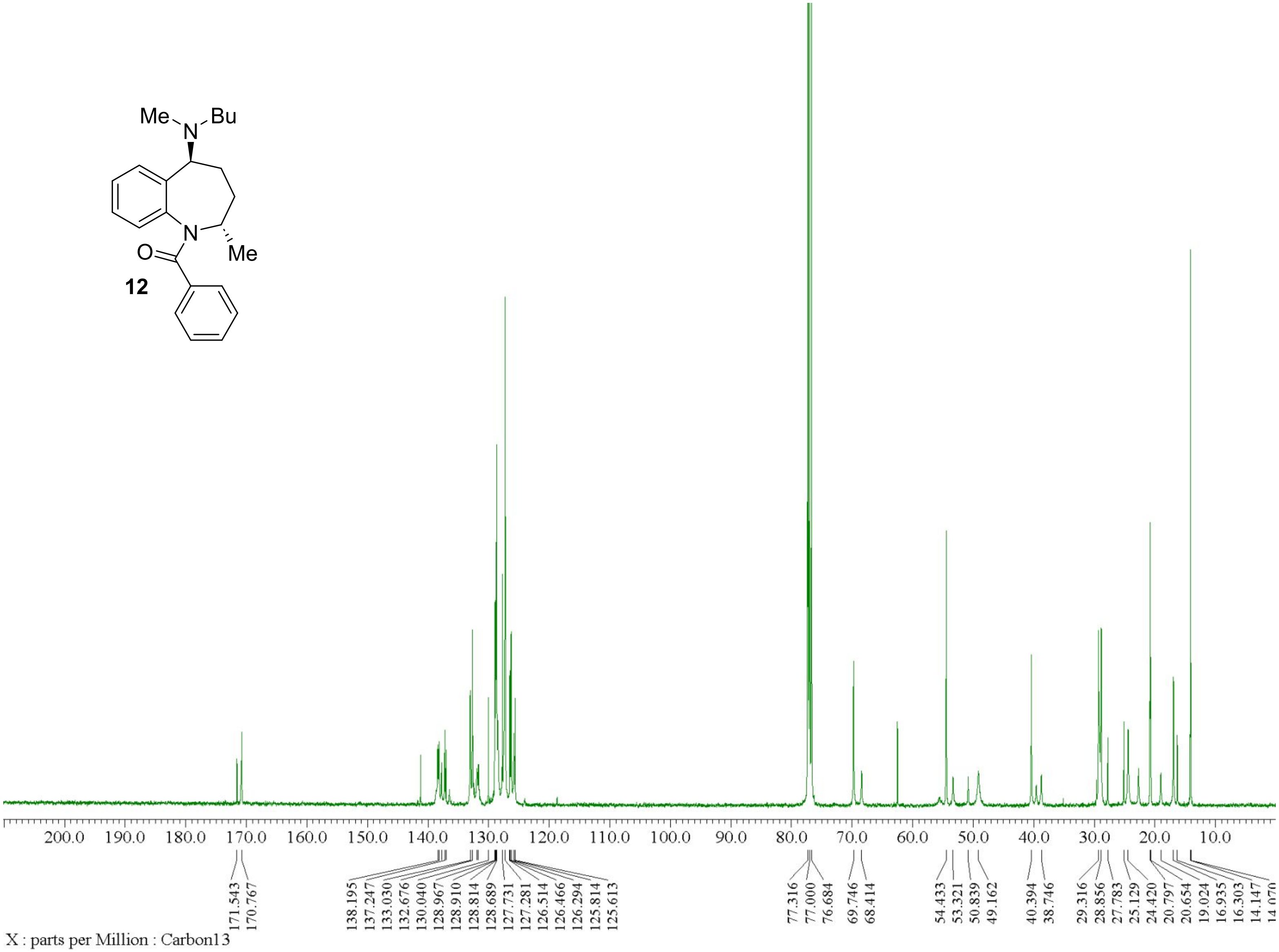

$^{13}C\{1H\}$ NMR, 100 MHz, $CDCl_3$

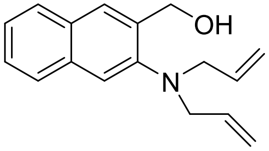

SI-1

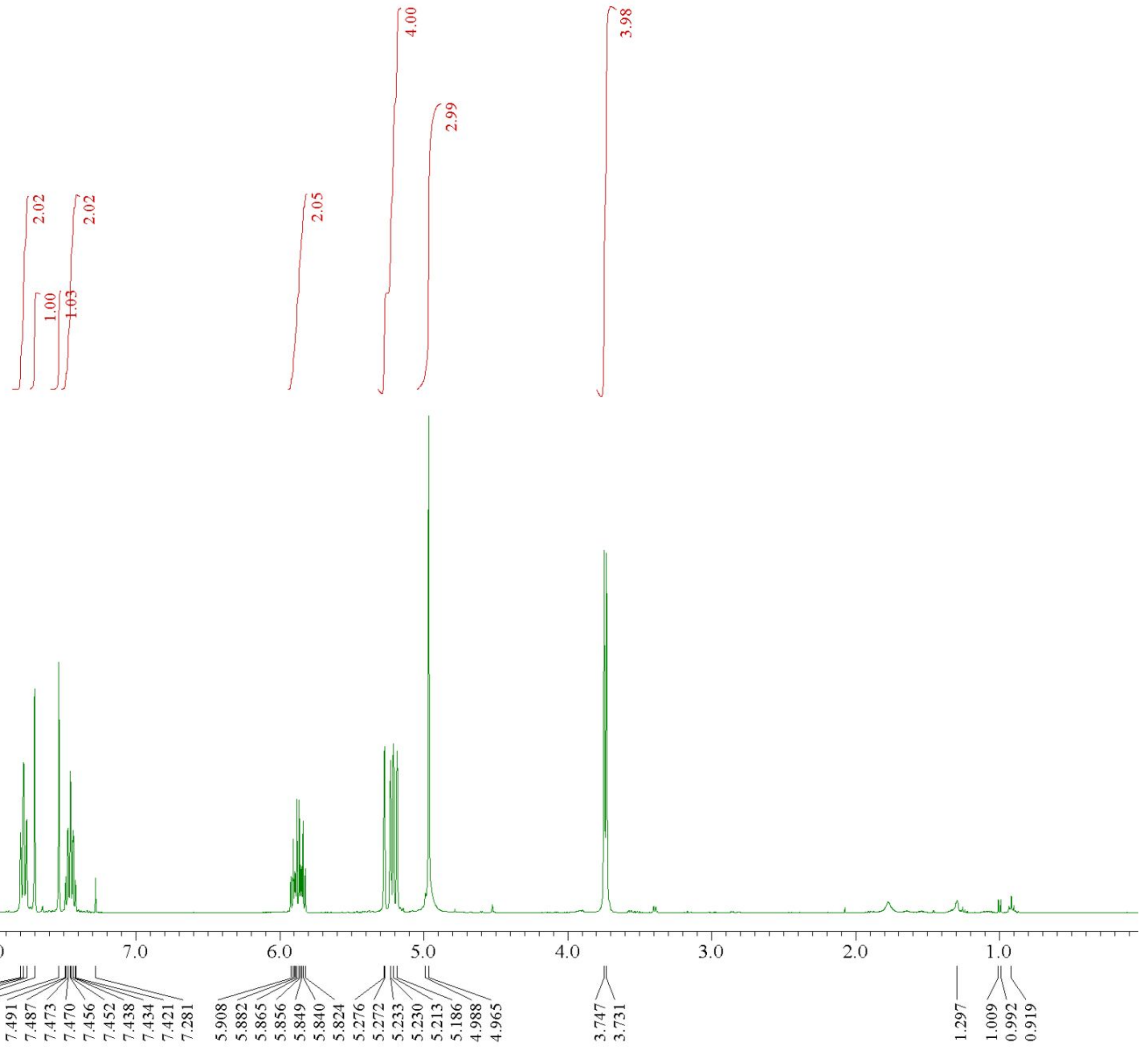

^{1}H NMR, 400 MHz, $CDCl_3$

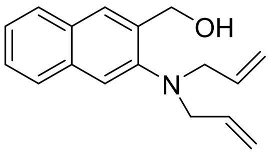

SI-1

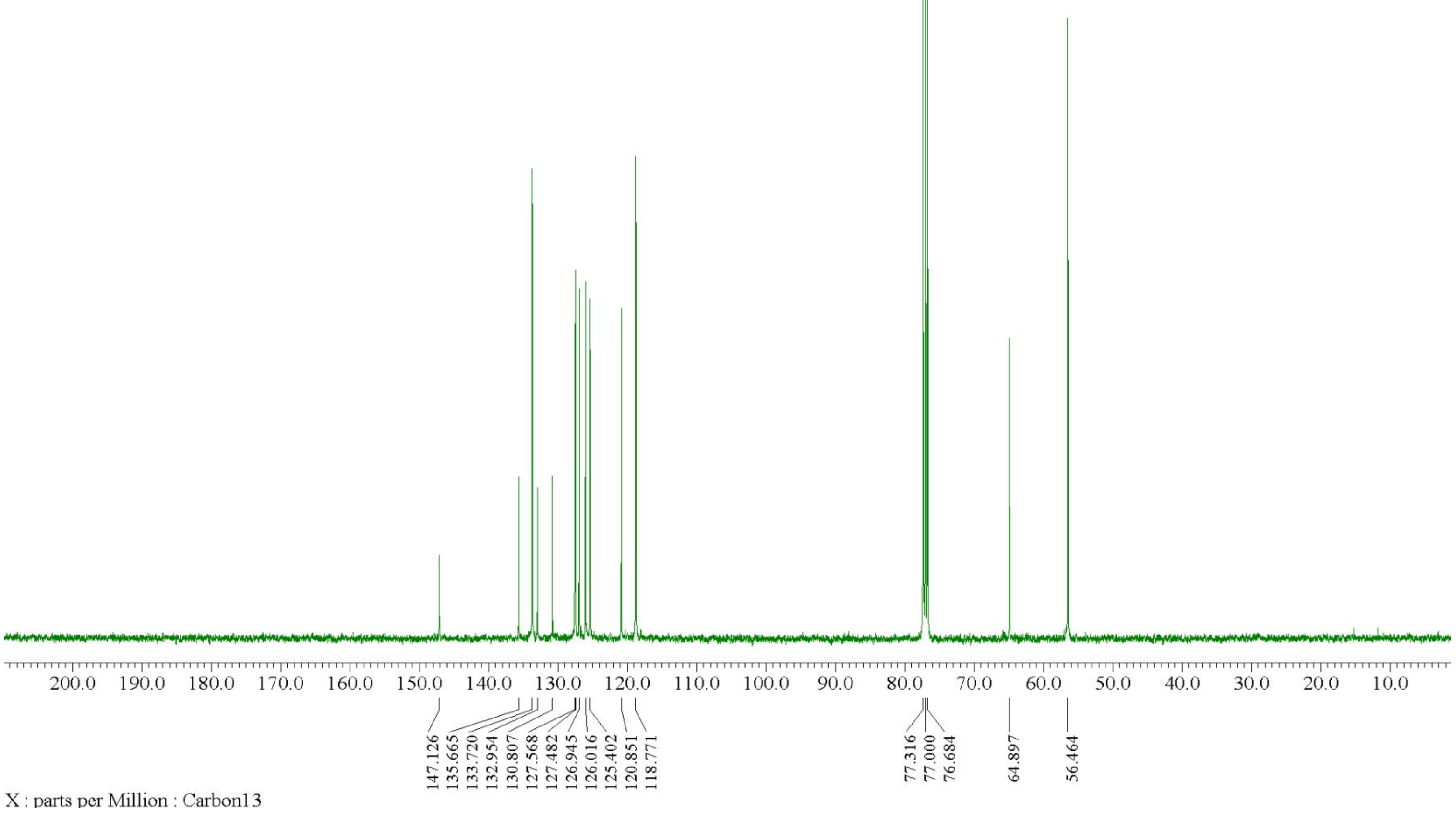

$^{13}C\{1H\}$ NMR, 100 MHz, $CDCl_3$

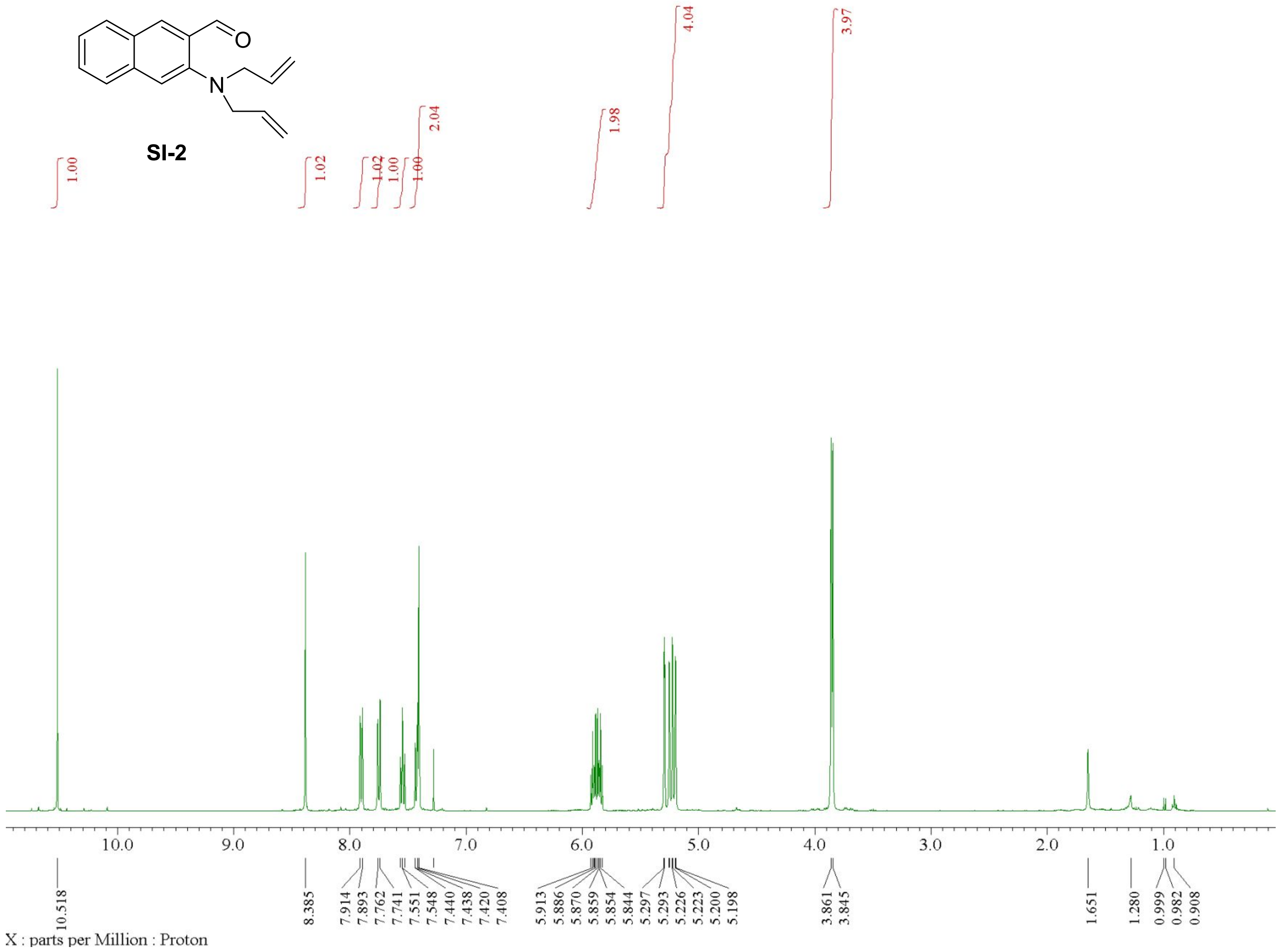

^{1}H NMR, 400 MHz, $CDCl_3$

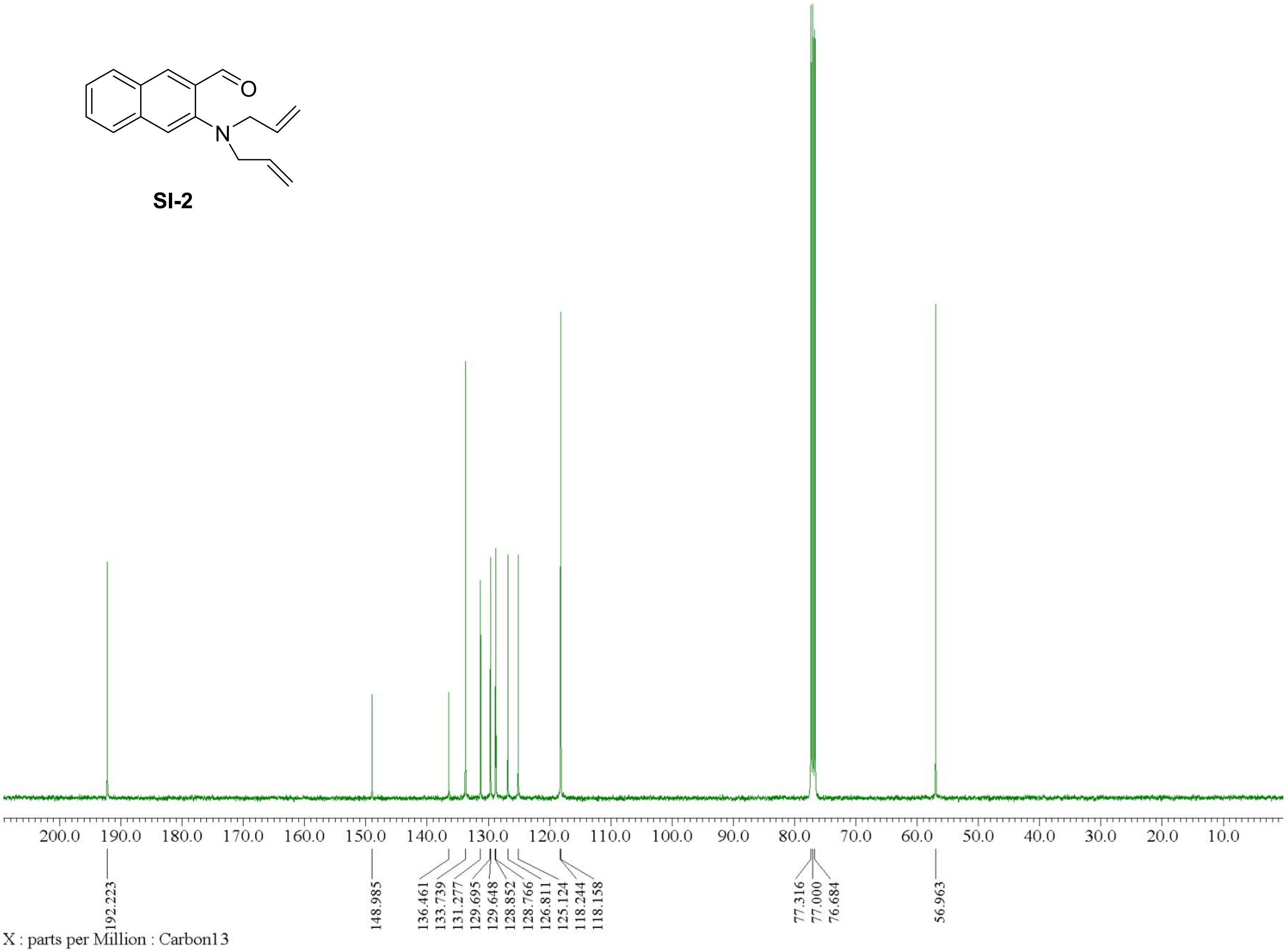

SI-2

$^{13}C\{1H\}$ NMR, 100 MHz, $CDCl_3$

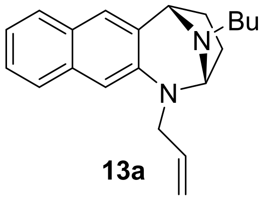

13a

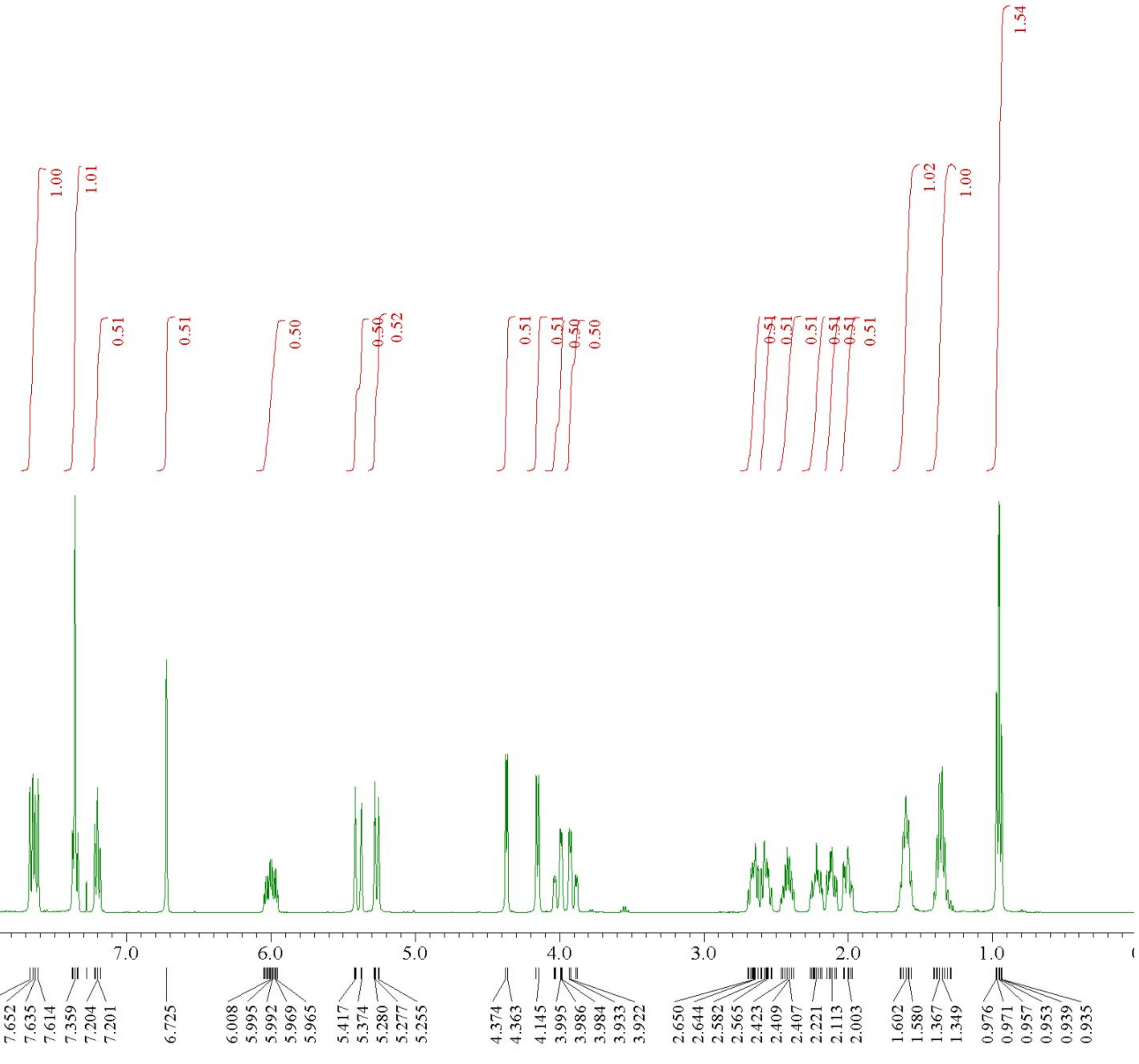

^1H NMR, 400 MHz, $CDCl_3$

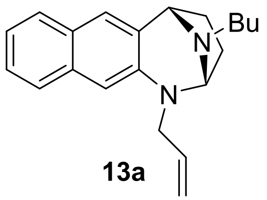

13a

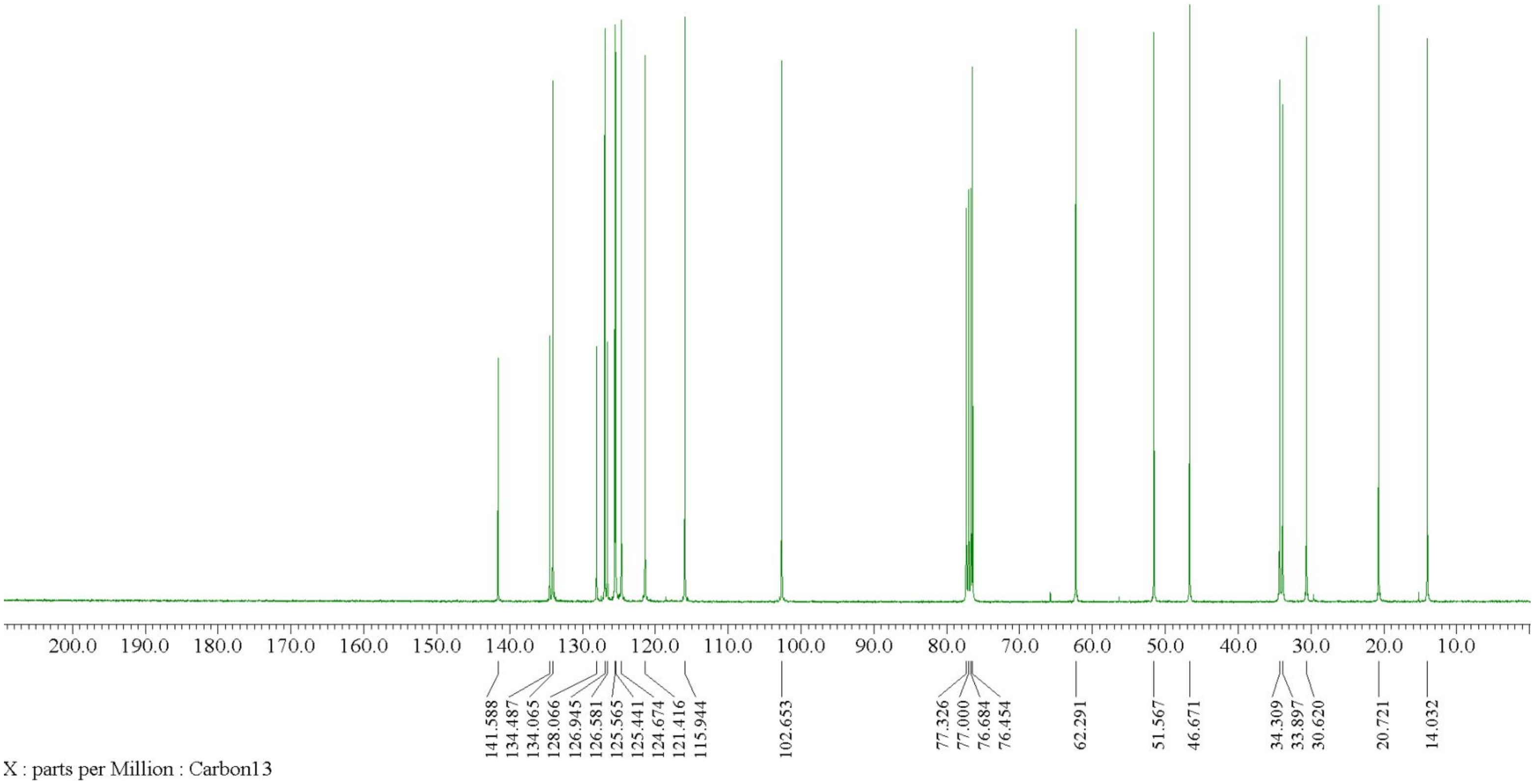

^{13}C NMR, 100 MHz, $CDCl_3$

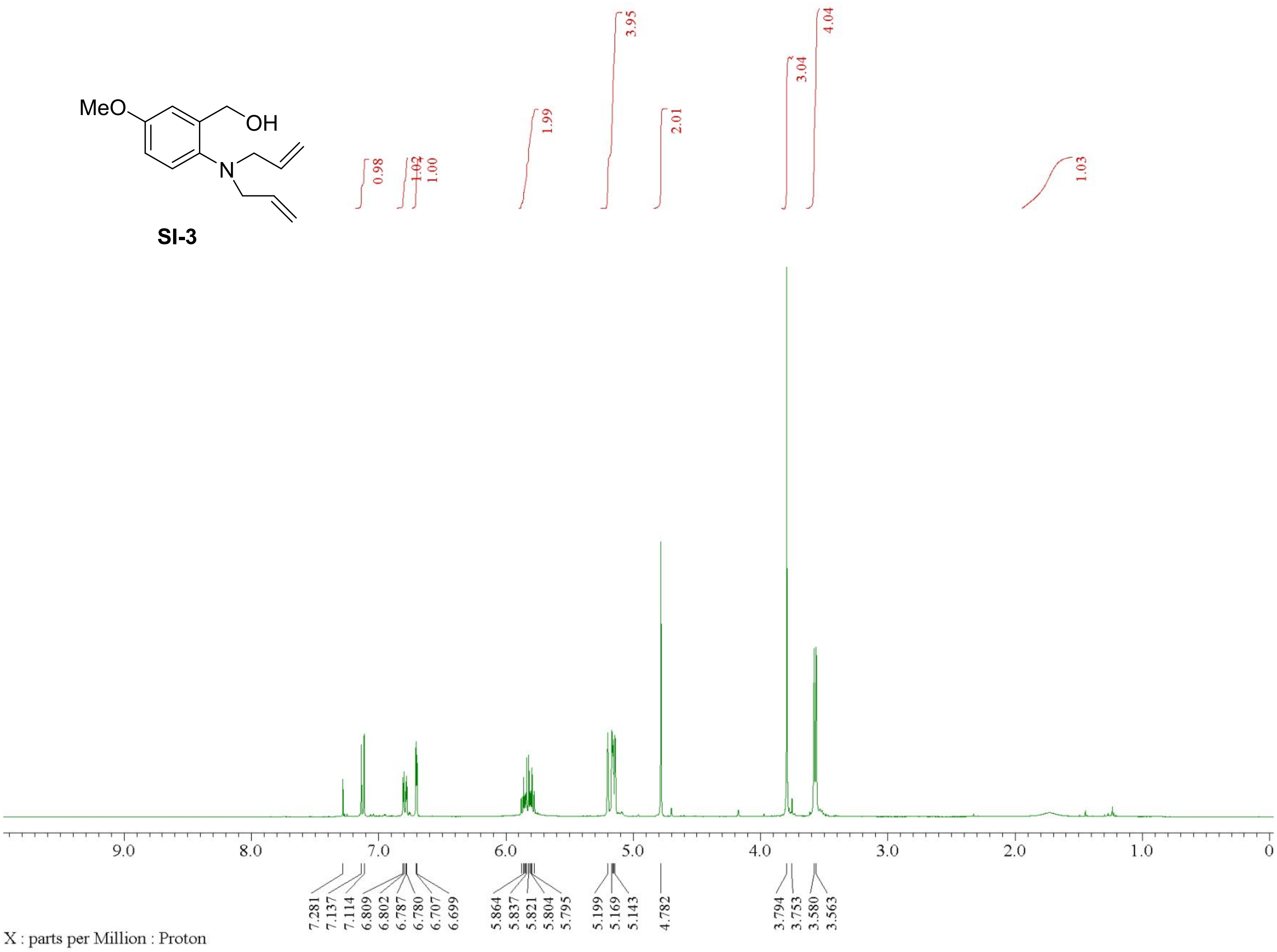

^{1}H NMR, 400 MHz, $CDCl_3$

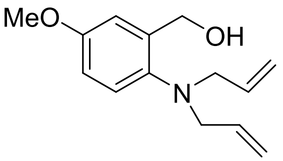

SI-3

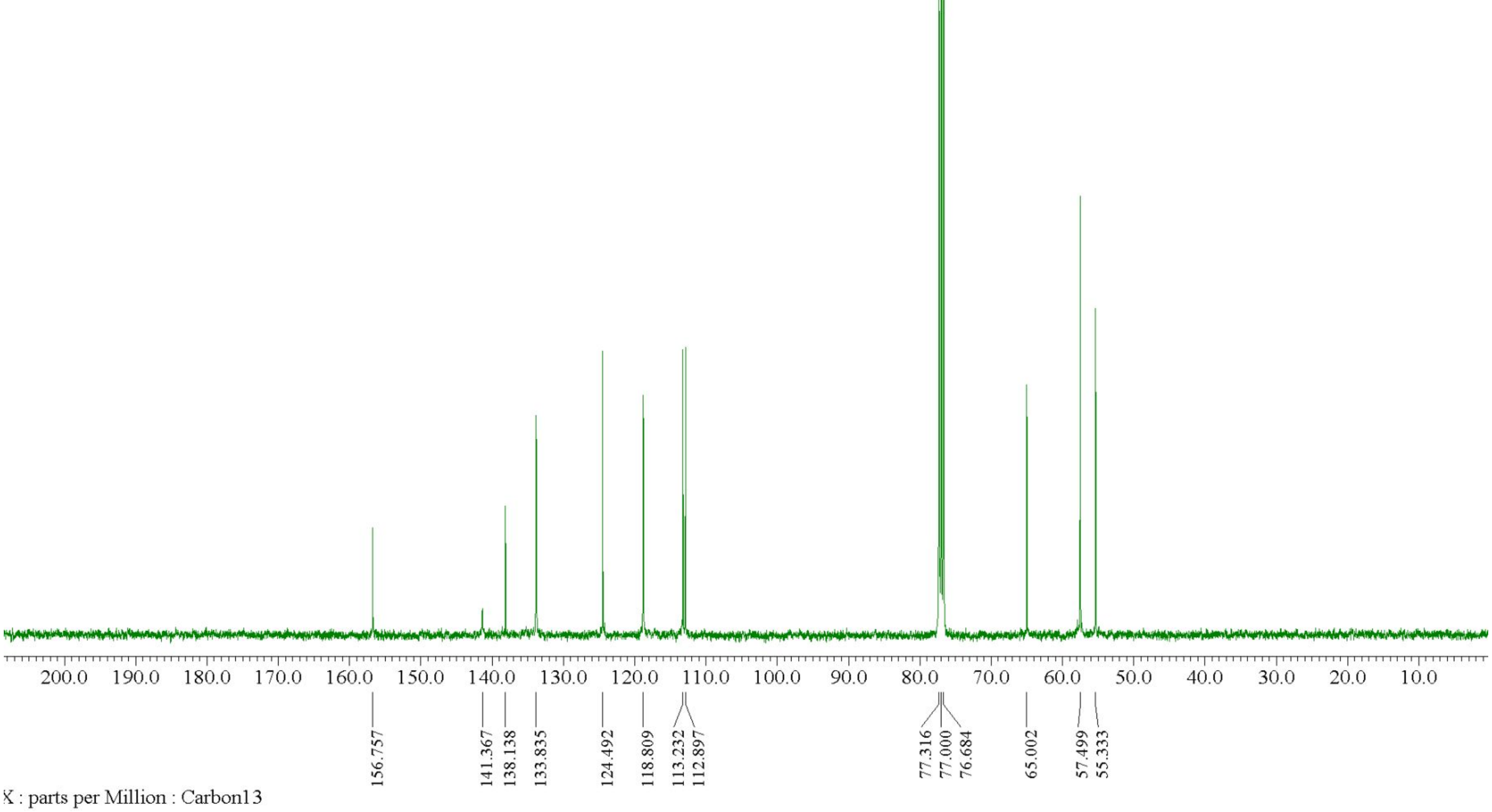

$^{13}C\{1H\}$ NMR, 100 MHz, $CDCl_3$

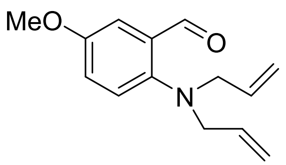

SI-4

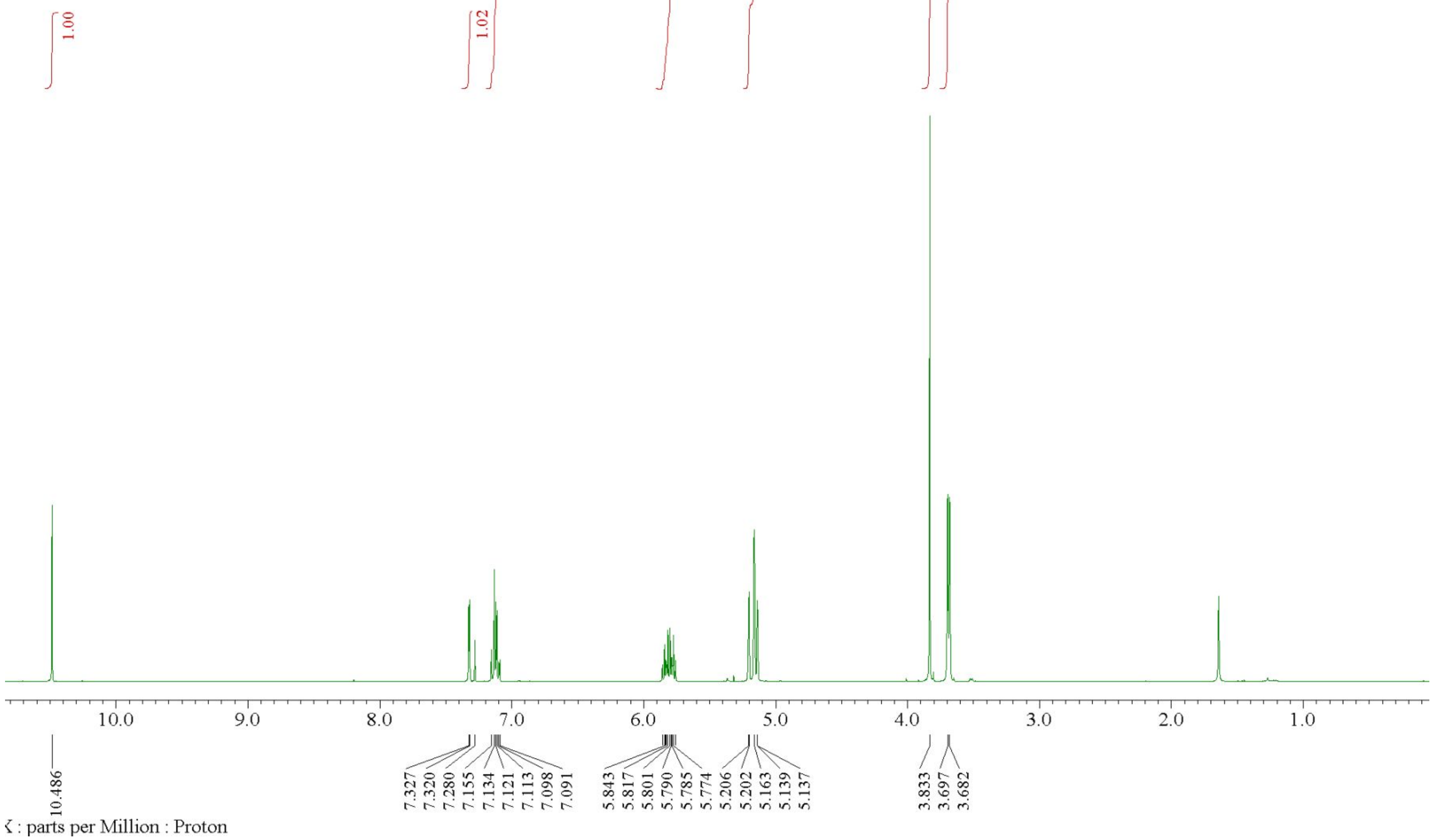

^{1}H NMR, 400 MHz, $CDCl_3$

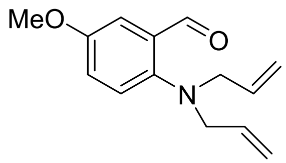

SI-4

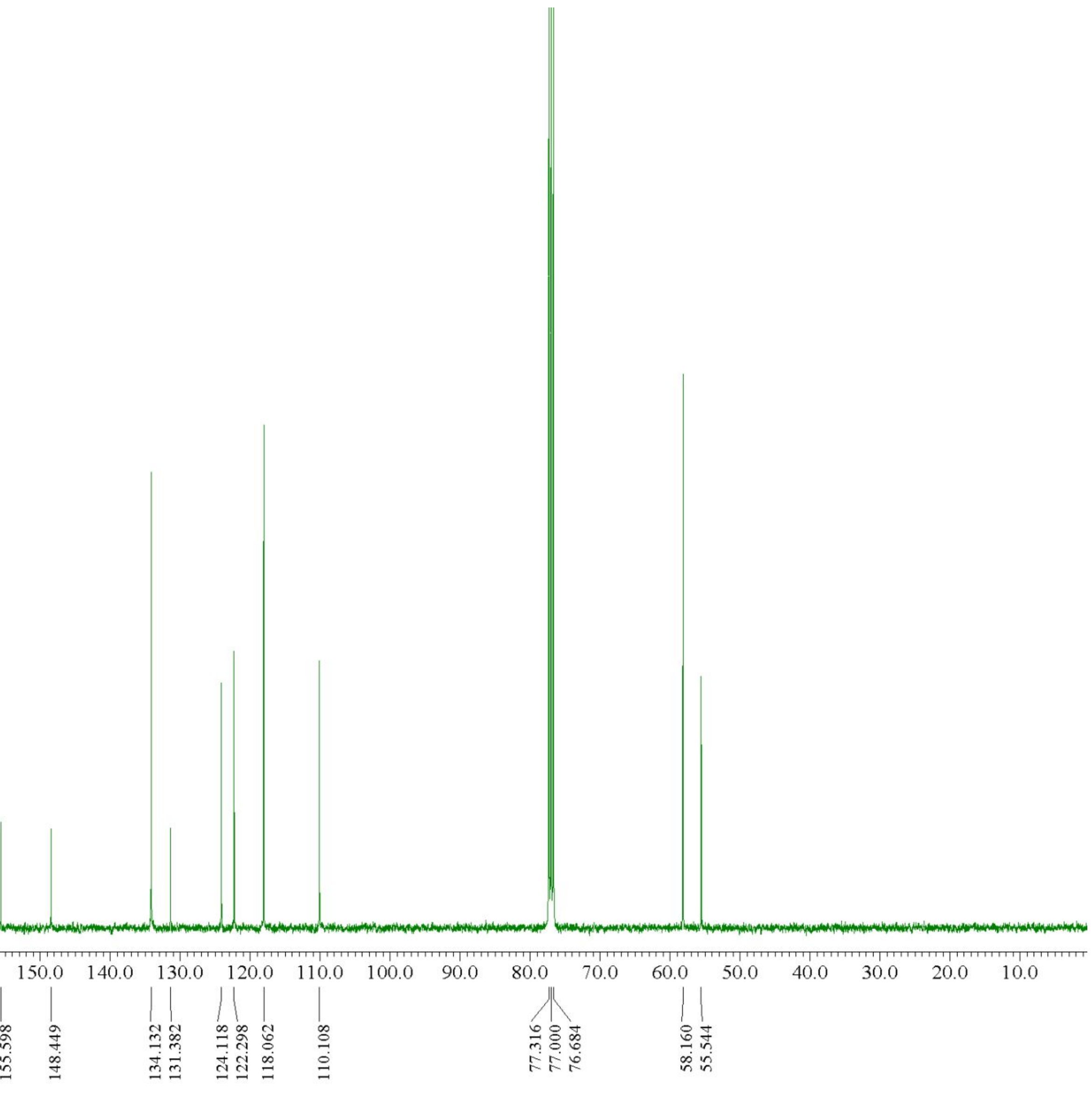

^{13}C NMR, 100 MHz, $CDCl_3$

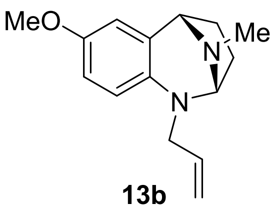

13b

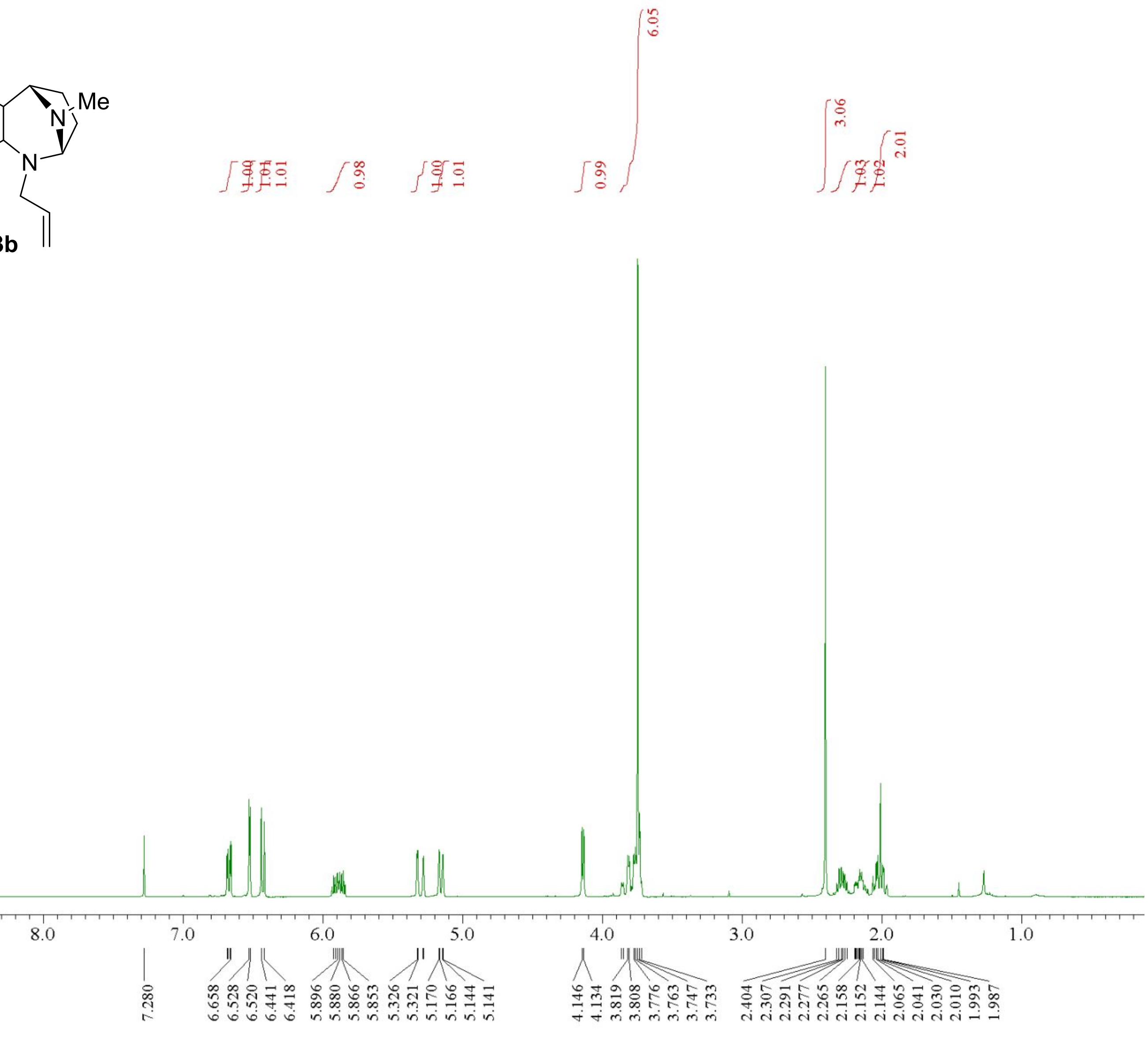

^{1}H NMR, 400 MHz, $CDCl_3$

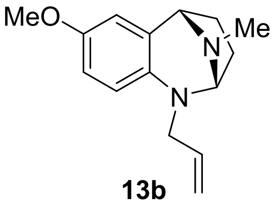

13b

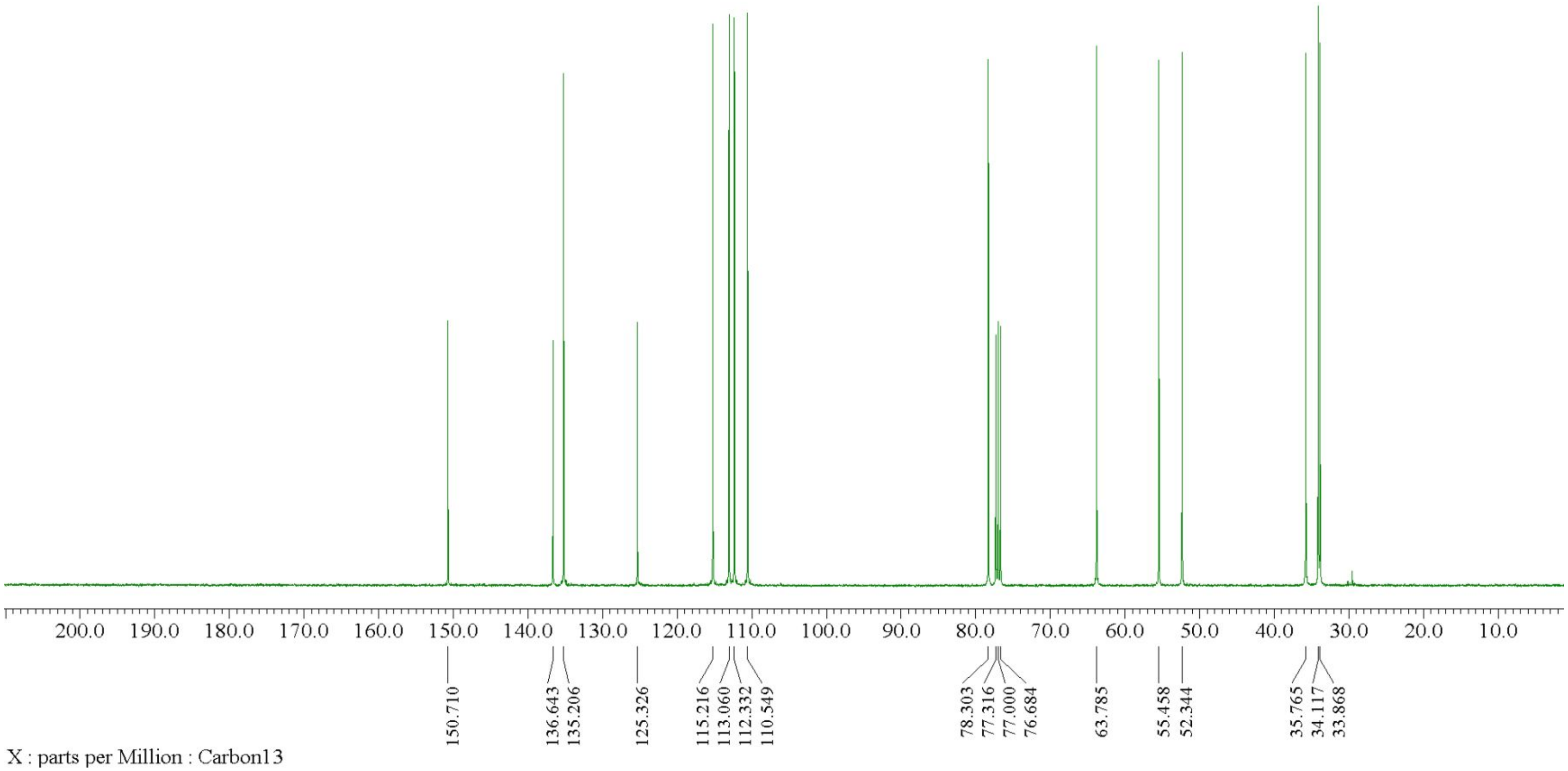

$^{13}C\{1H\}$ NMR, 100 MHz, $CDCl_3$

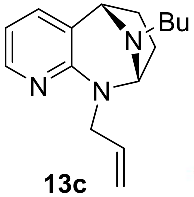

13c

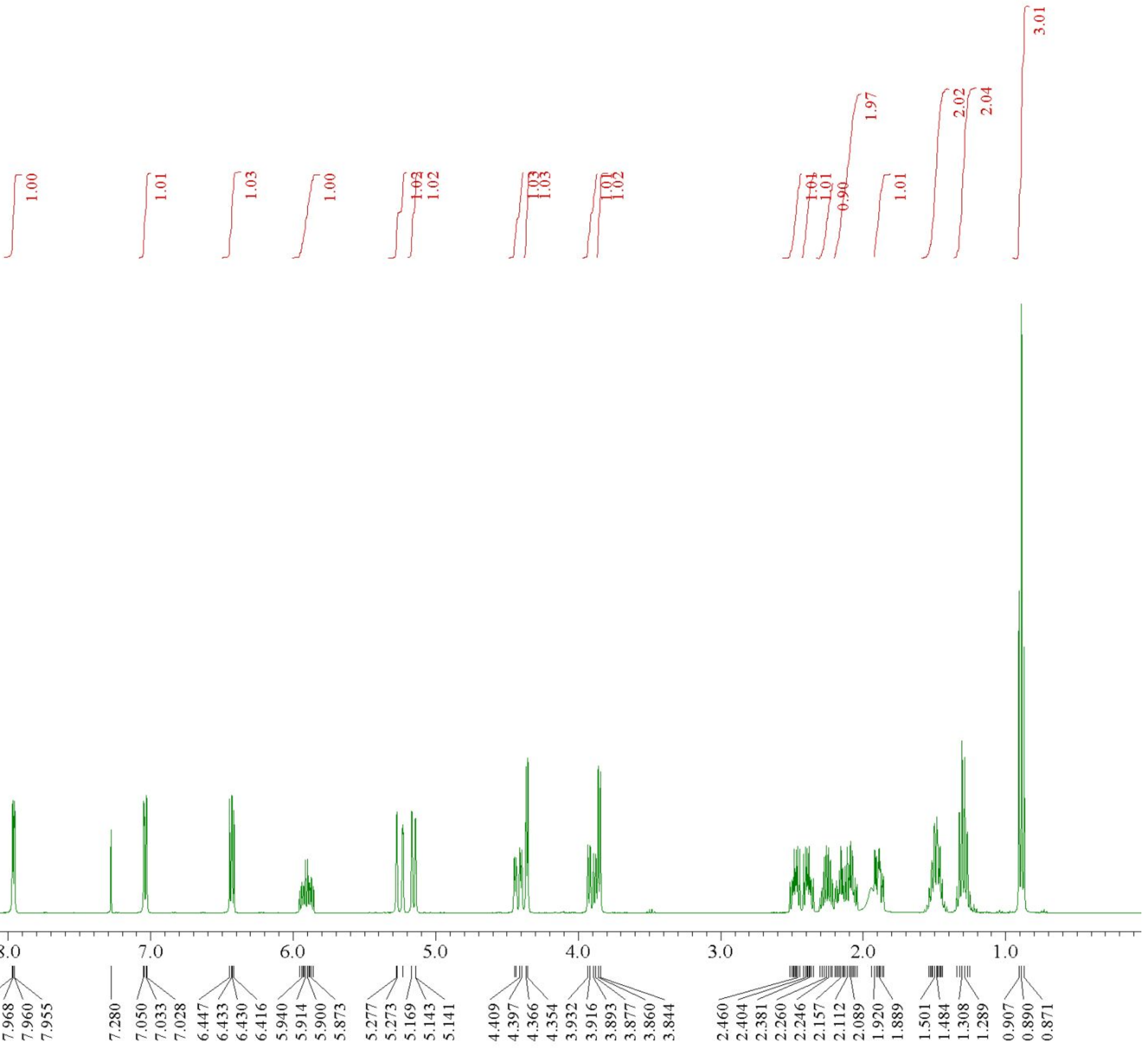

^{1}H NMR, 400 MHz, $CDCl_3$

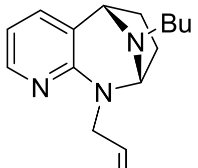

13c

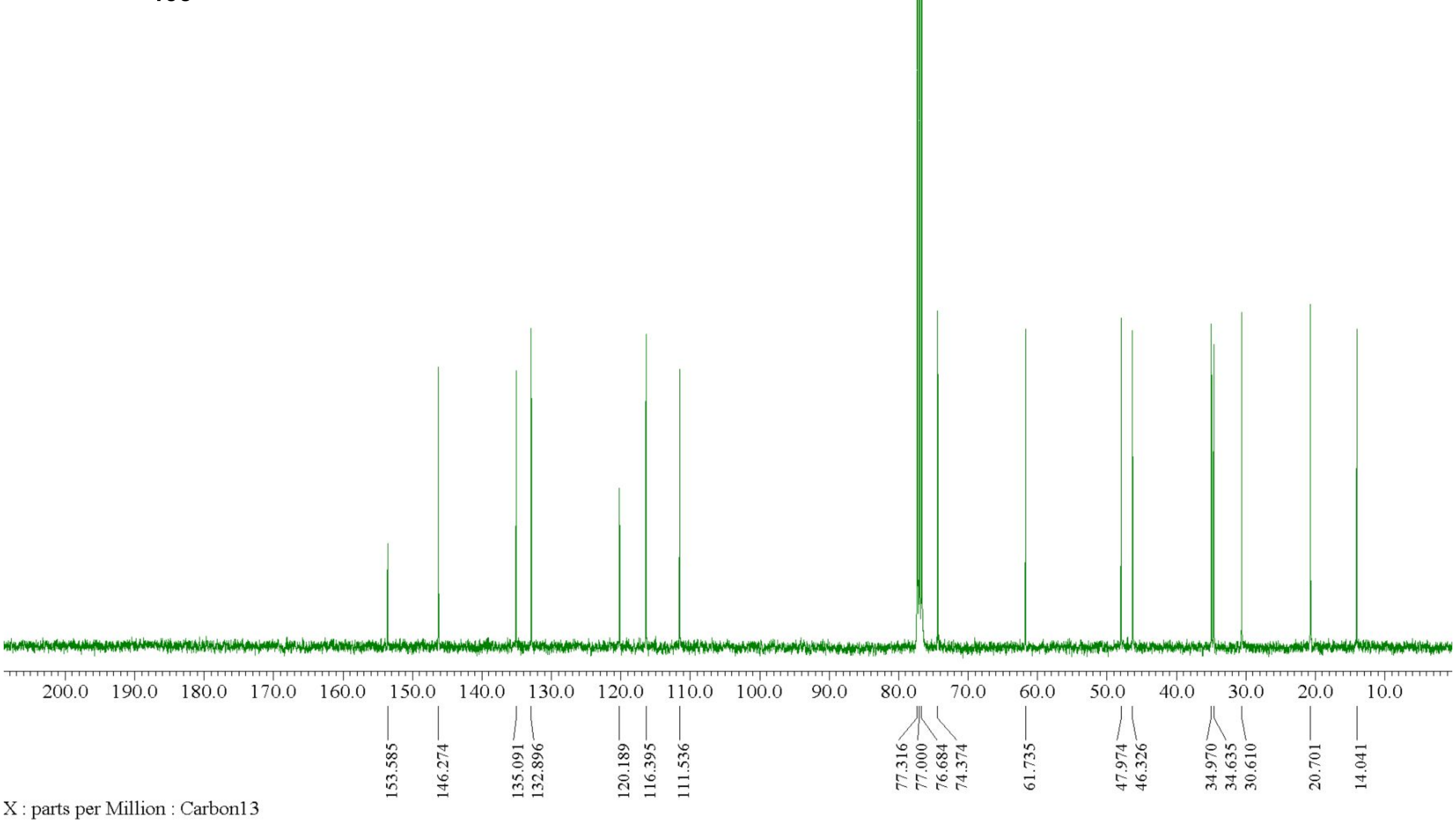

$^{13}C\{1H\}$ NMR, 100 MHz, $CDCl_3$

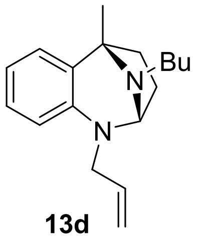

13d

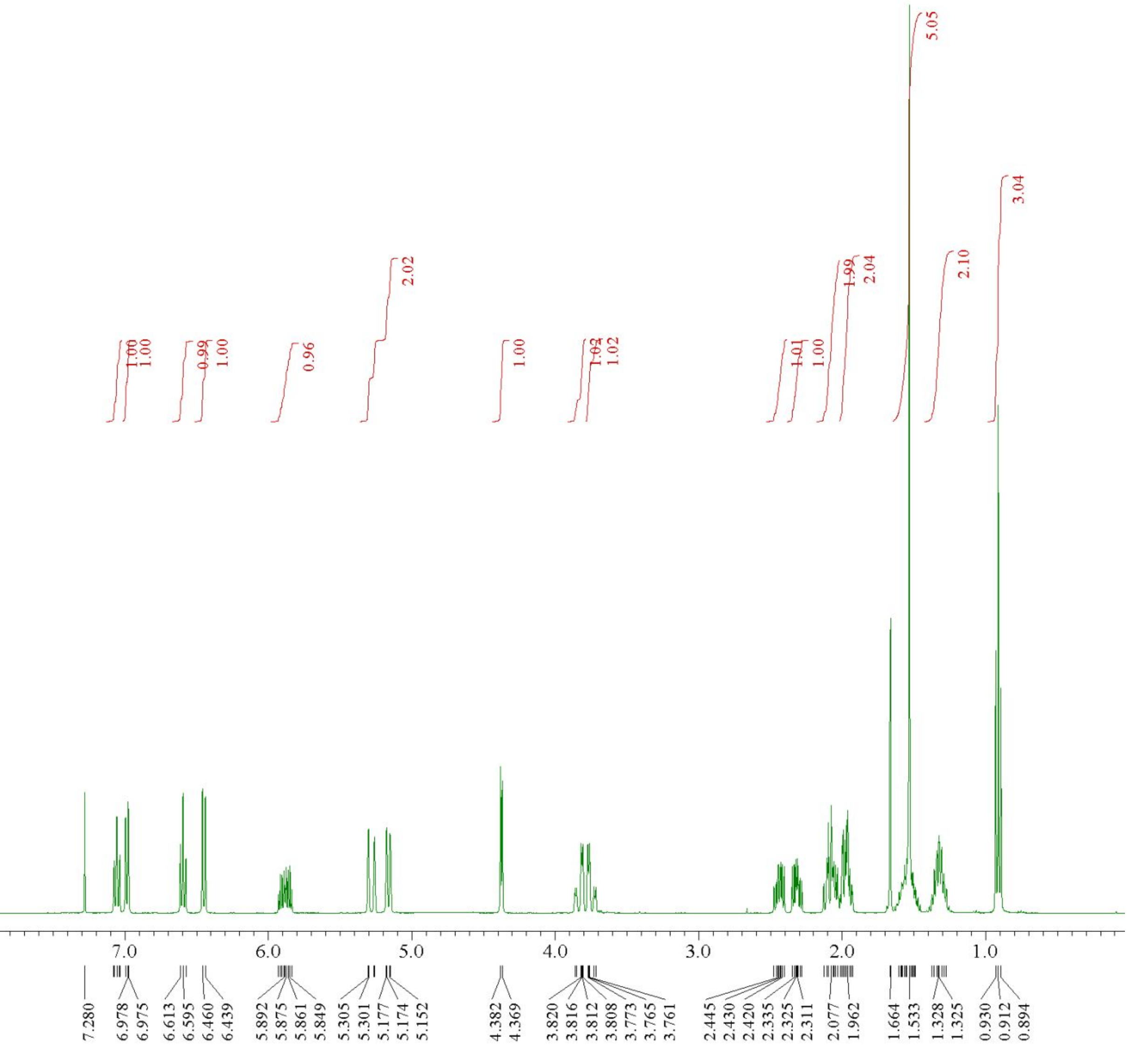

^{1}H NMR, 400 MHz, $CDCl_3$

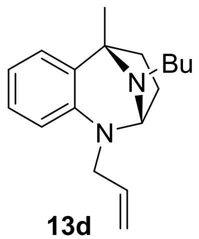

13d

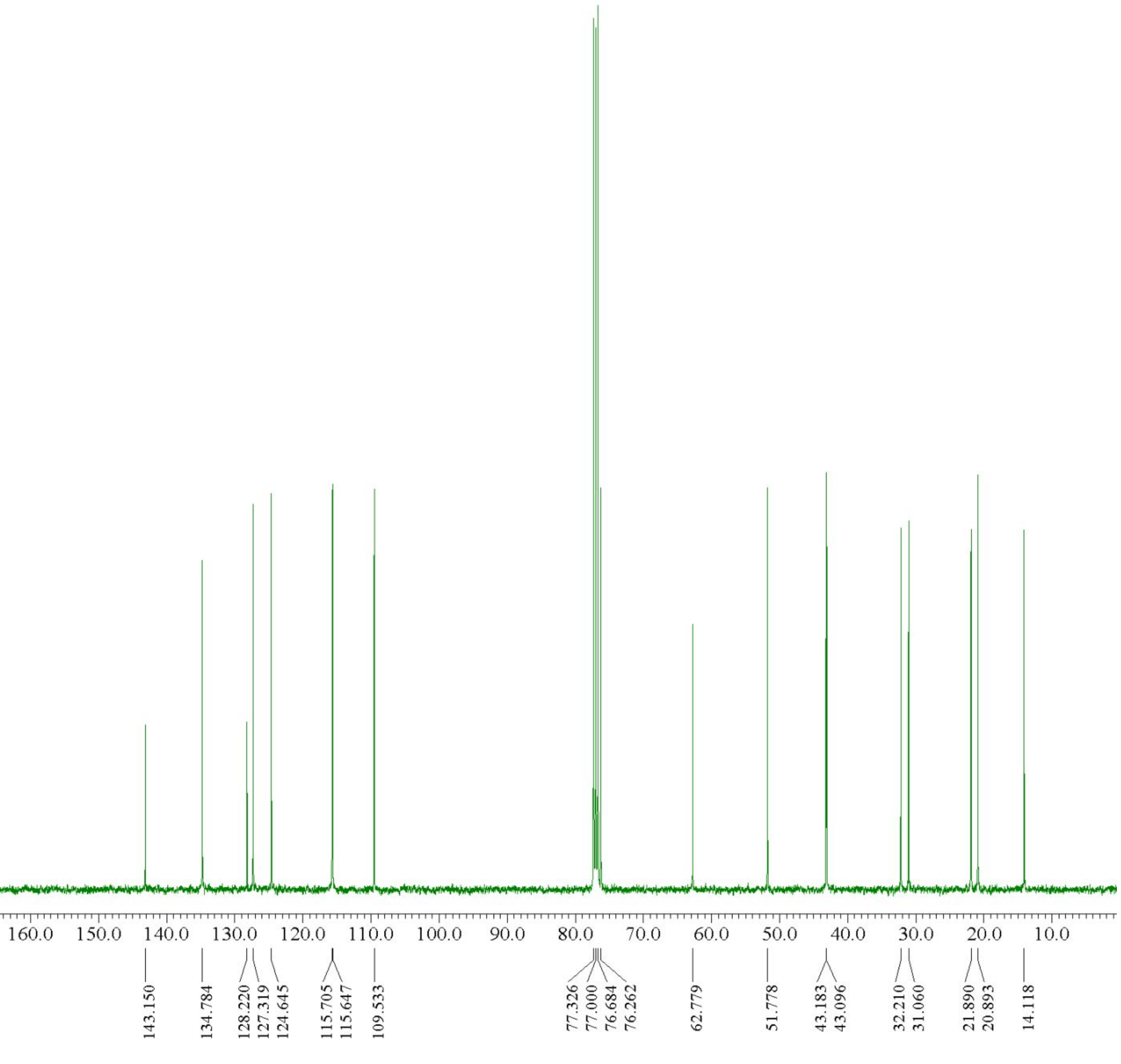

^{13}C NMR, 100 MHz, $CDCl_3$

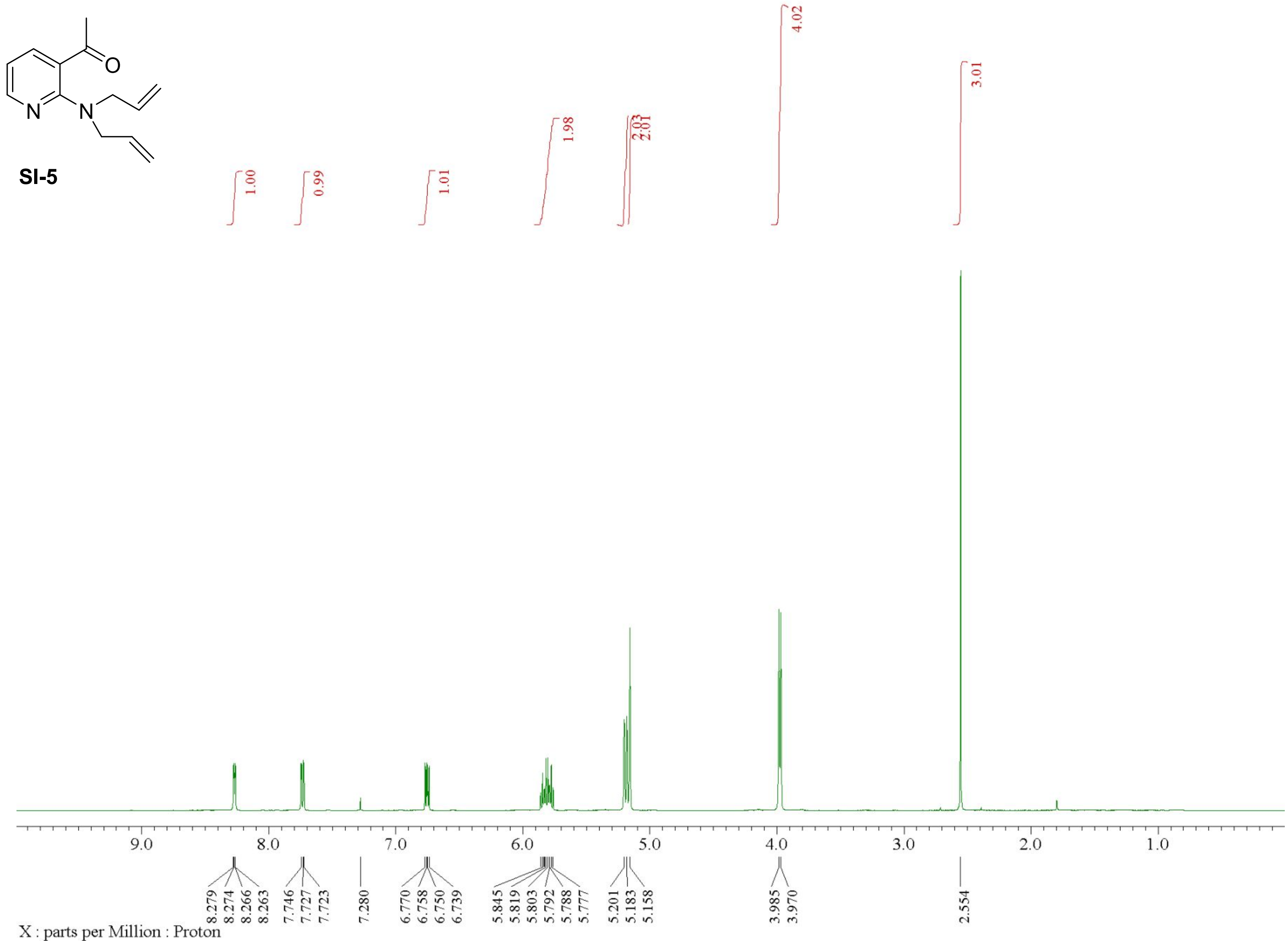

^{1}H NMR, 400 MHz, $CDCl_3$

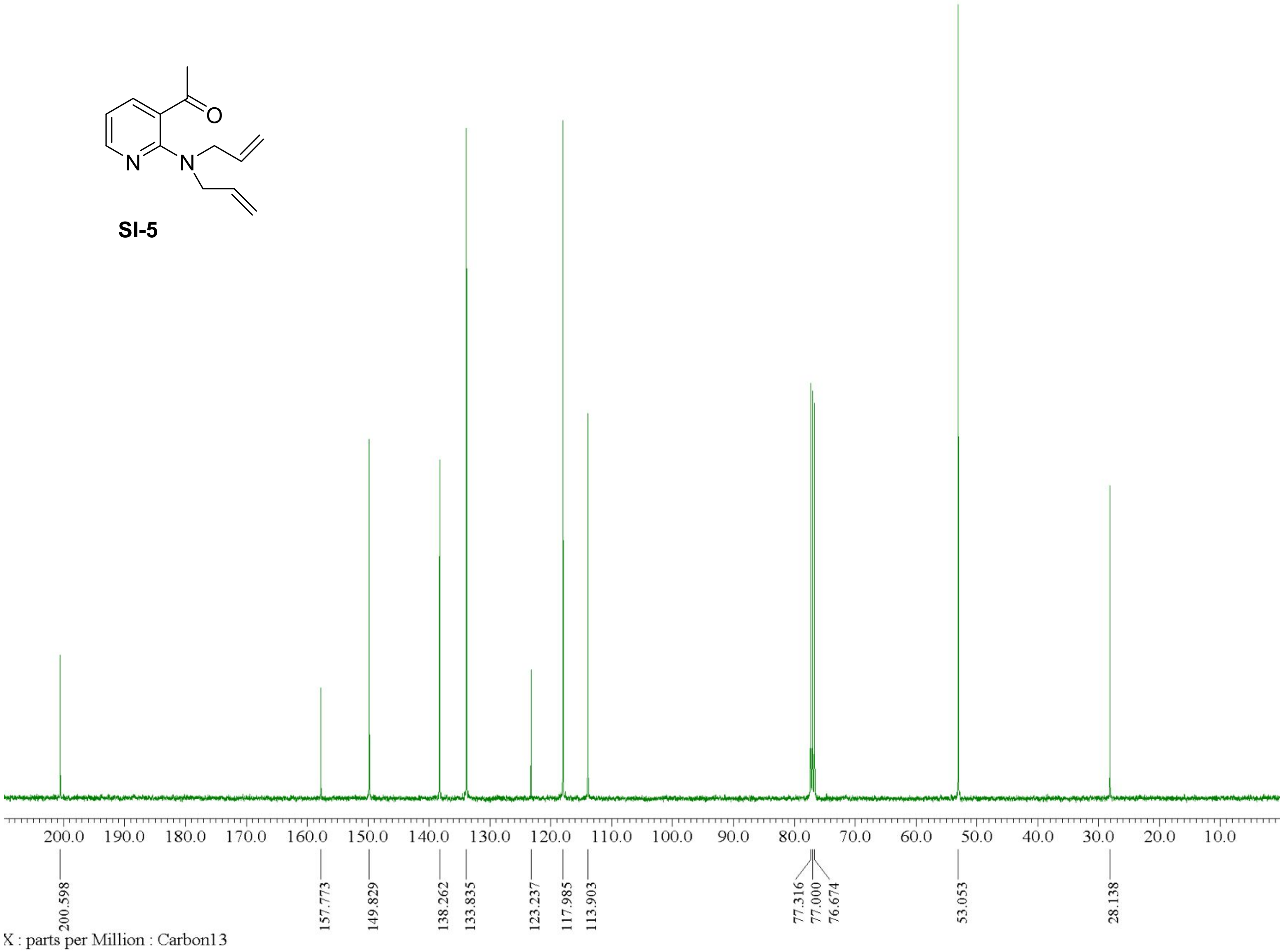

$^{13}C\{1H\}$ NMR, 100 MHz, $CDCl_3$

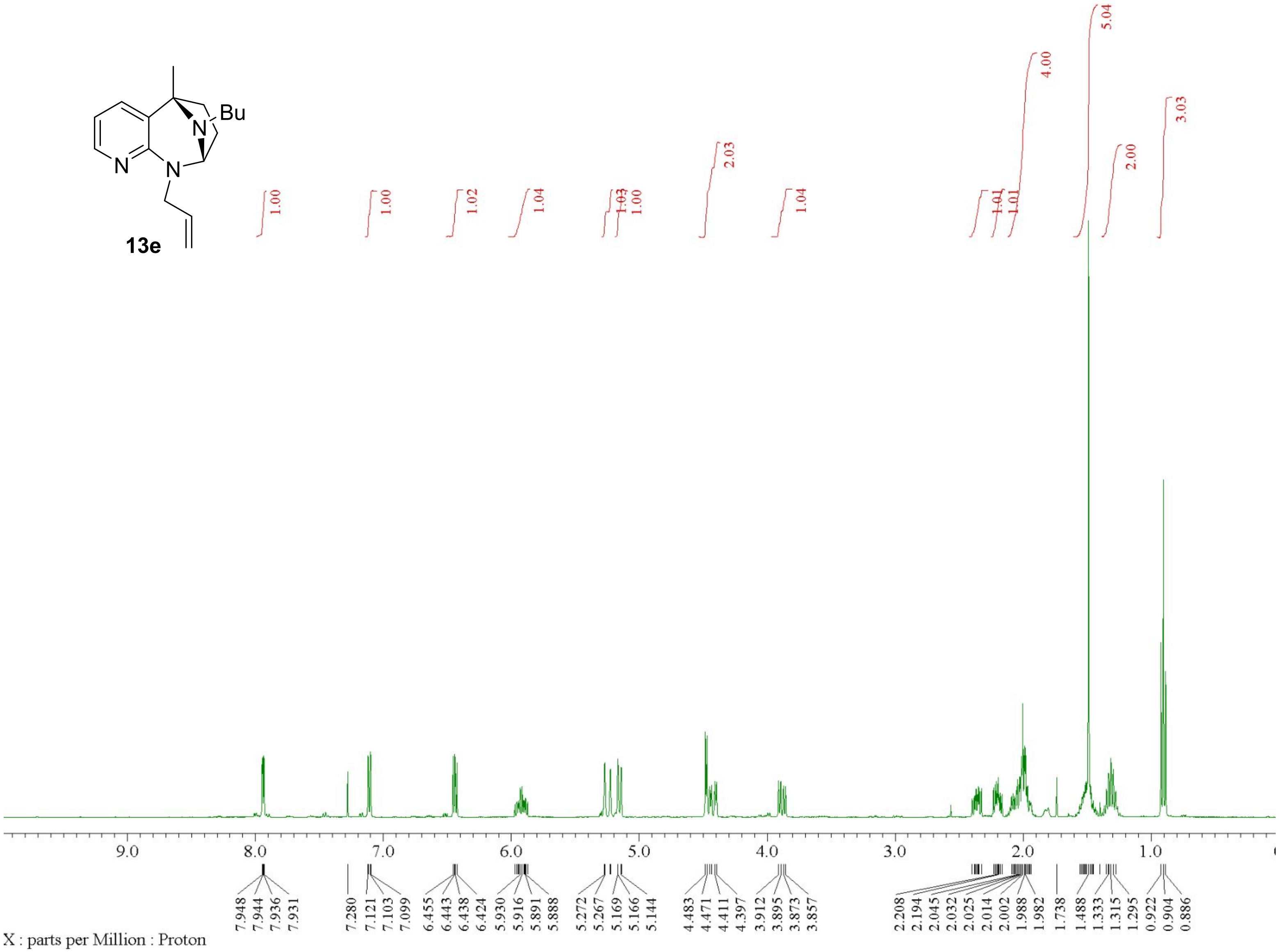

^{1}H NMR, 400 MHz, $CDCl_3$

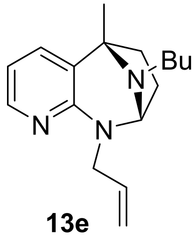

13e

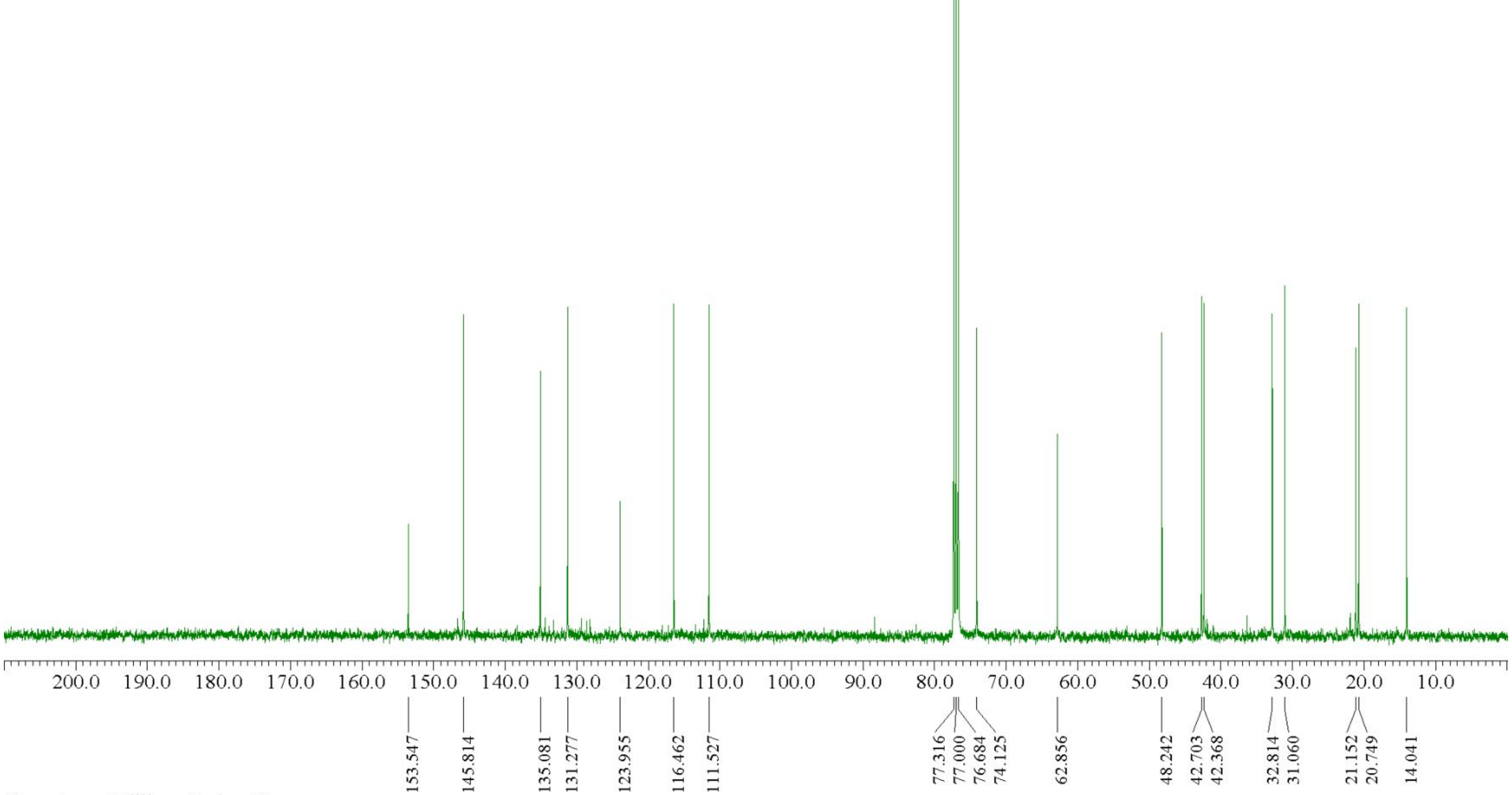

X : parts per Million : Carbon13

$^{13}C\{1H\}$ NMR, 100 MHz, $CDCl_3$

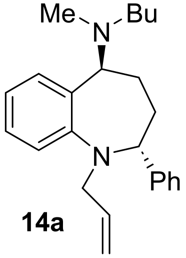

14a

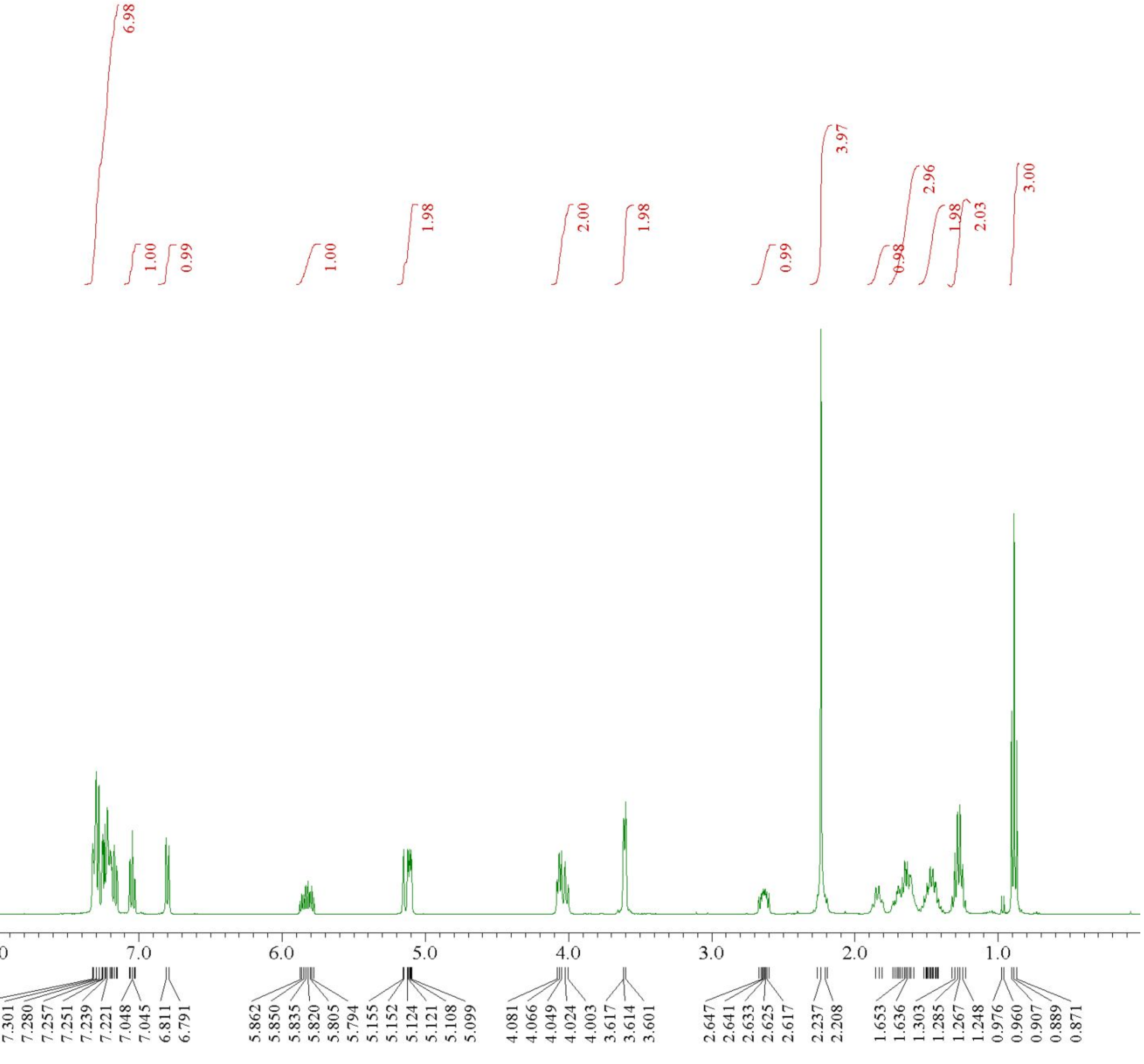

^1H NMR, 400 MHz, $CDCl_3$

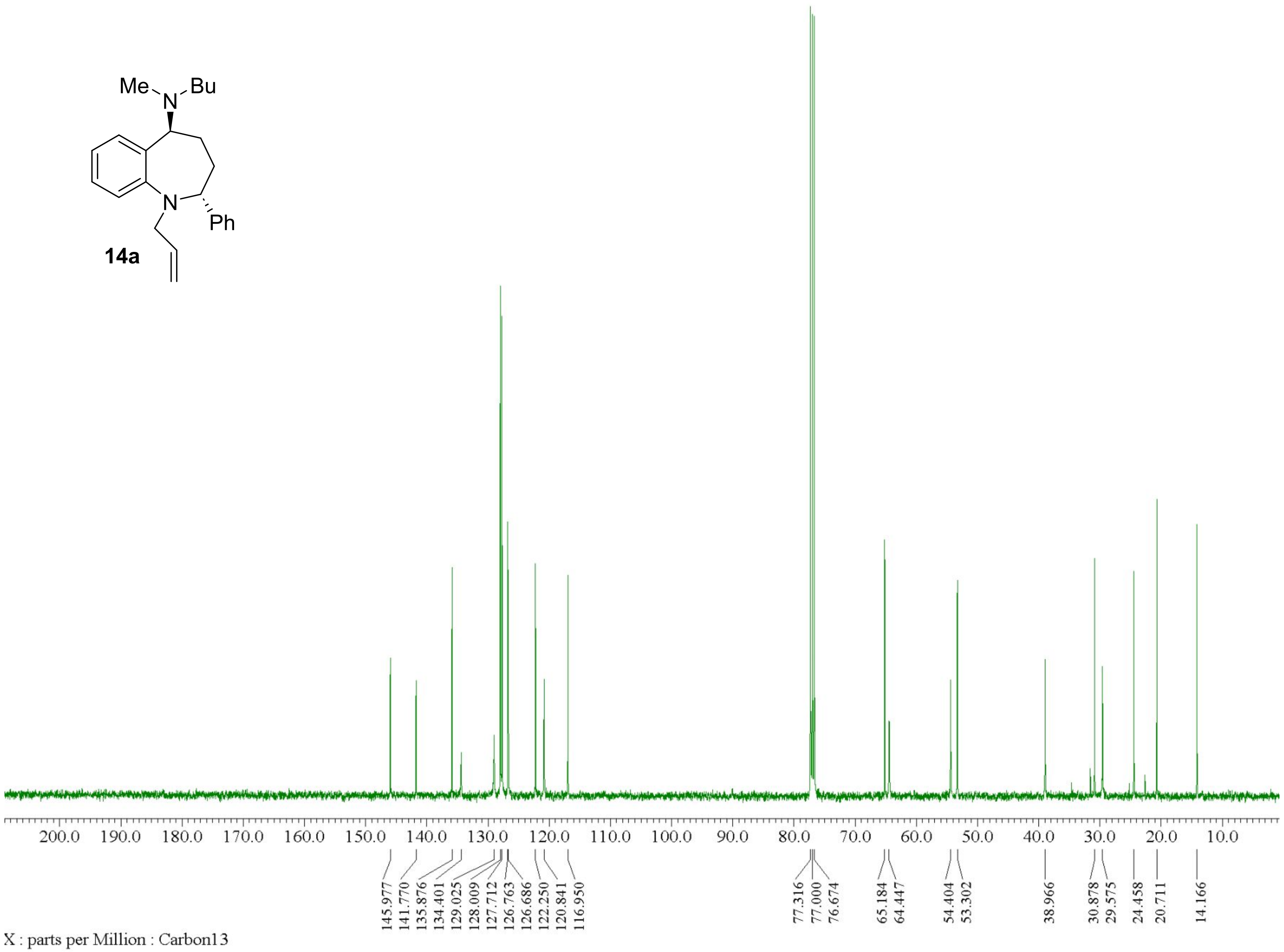

$^{13}C\{^1H\}$ NMR, 100 MHz, $CDCl_3$

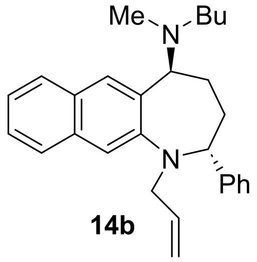

14b

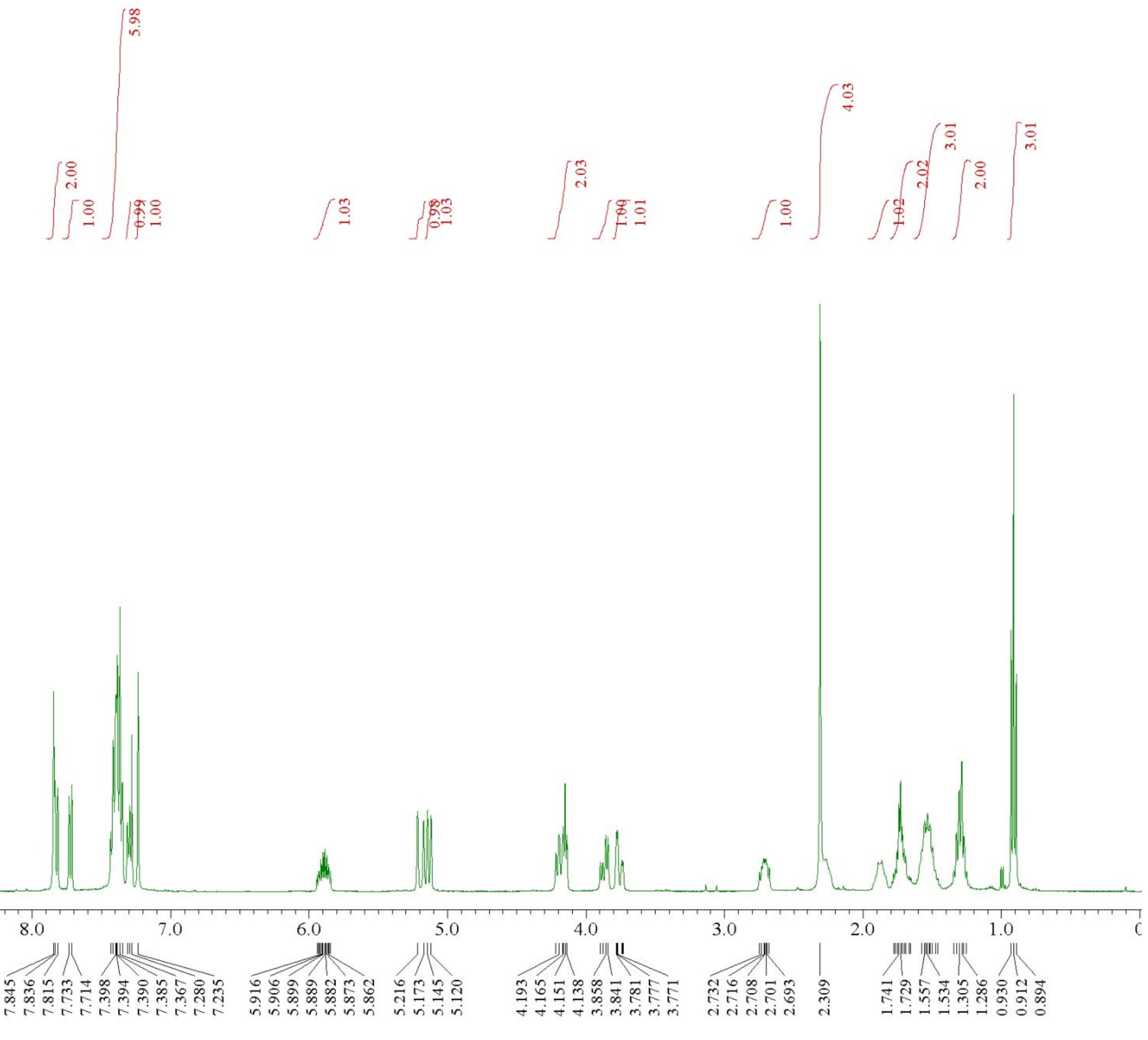

^{1}H NMR, 400 MHz, $CDCl_3$

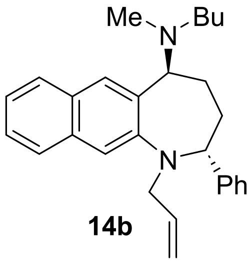

14b

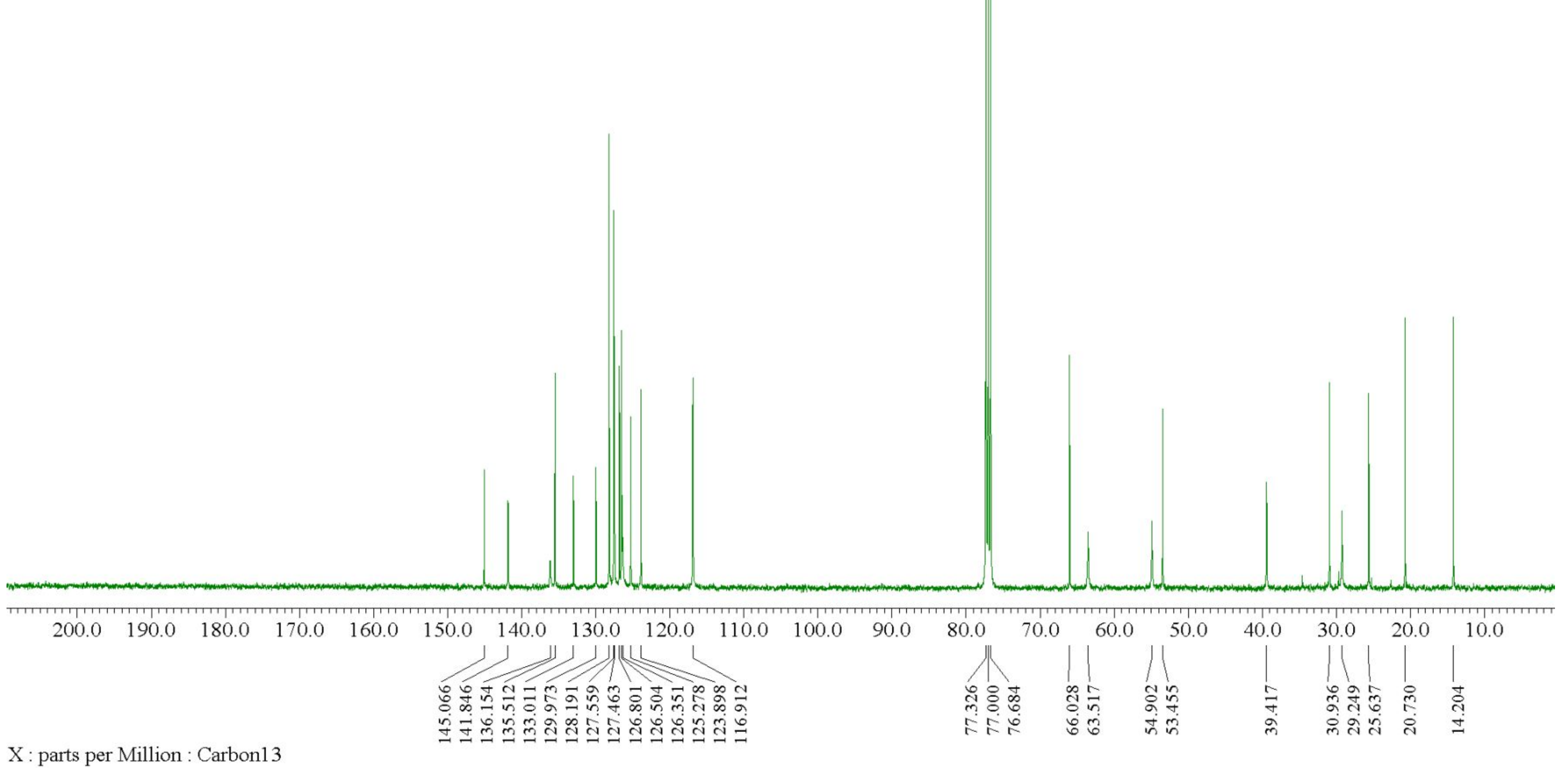

$^{13}C\{^1H\}$ NMR, 100 MHz, $CDCl_3$

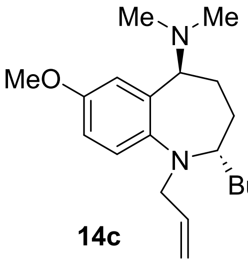

14c

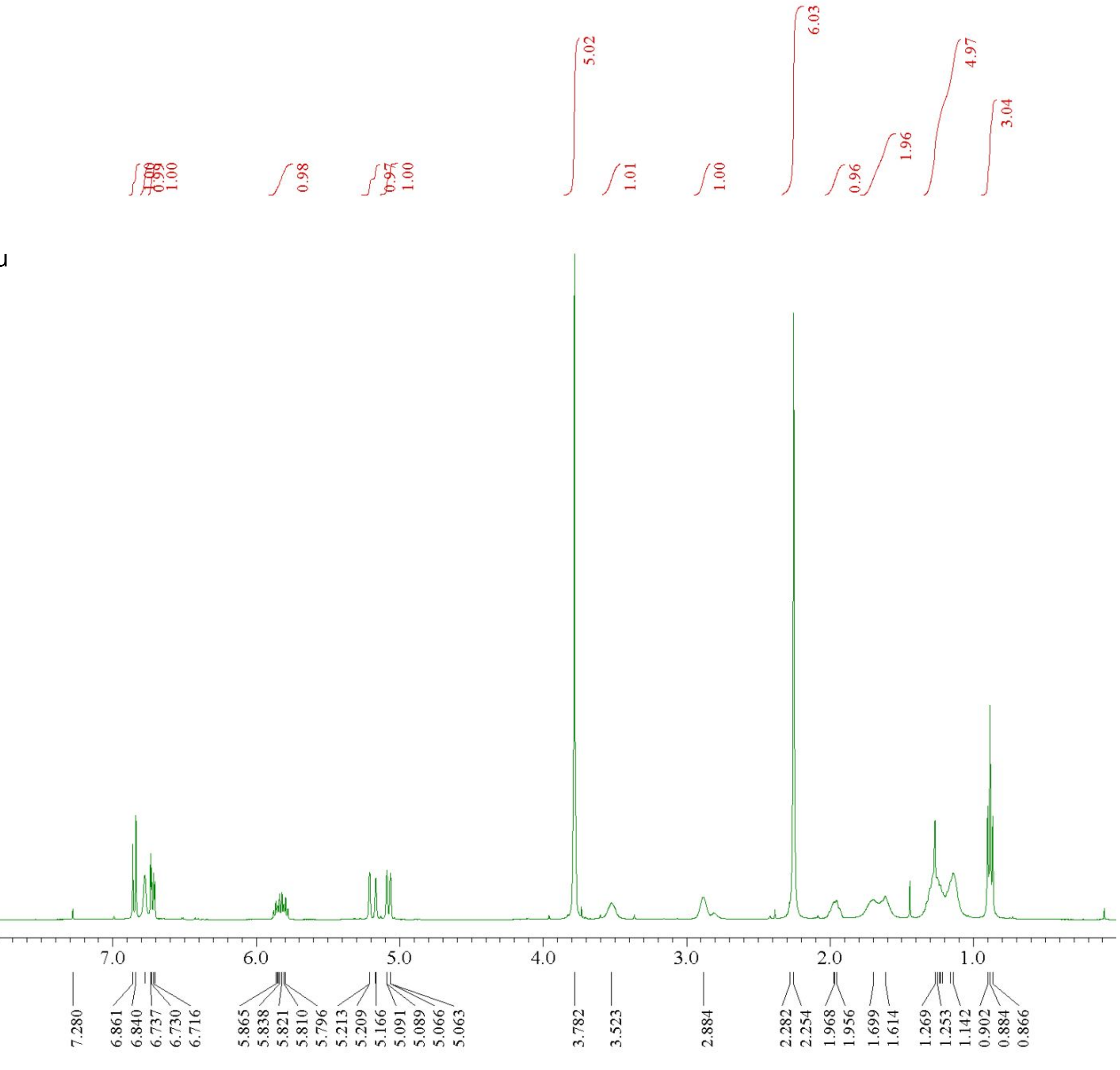

^1H NMR, 400 MHz, $CDCl_3$

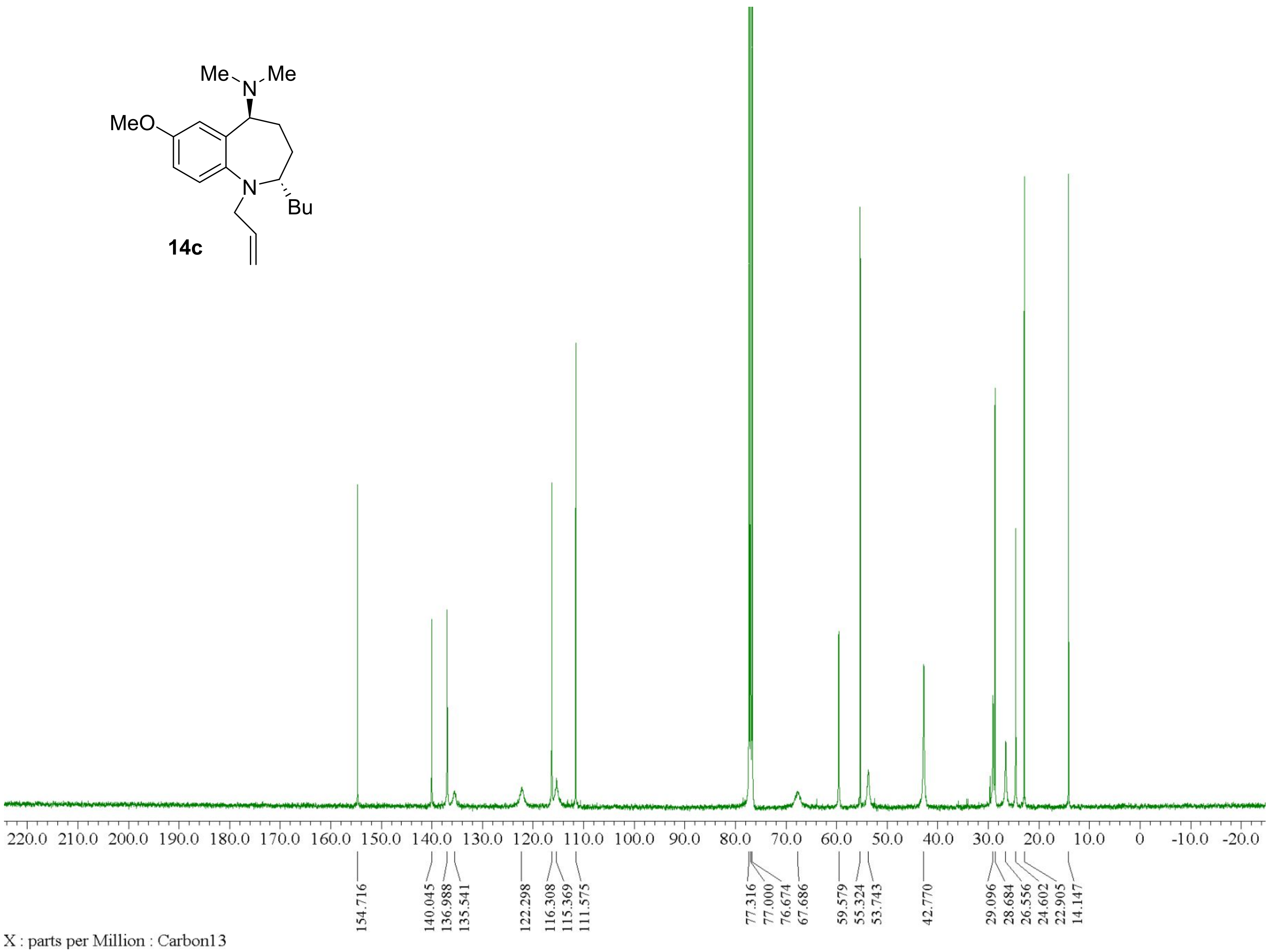

$^{13}C\{^1H\}$ NMR, 100 MHz, $CDCl_3$

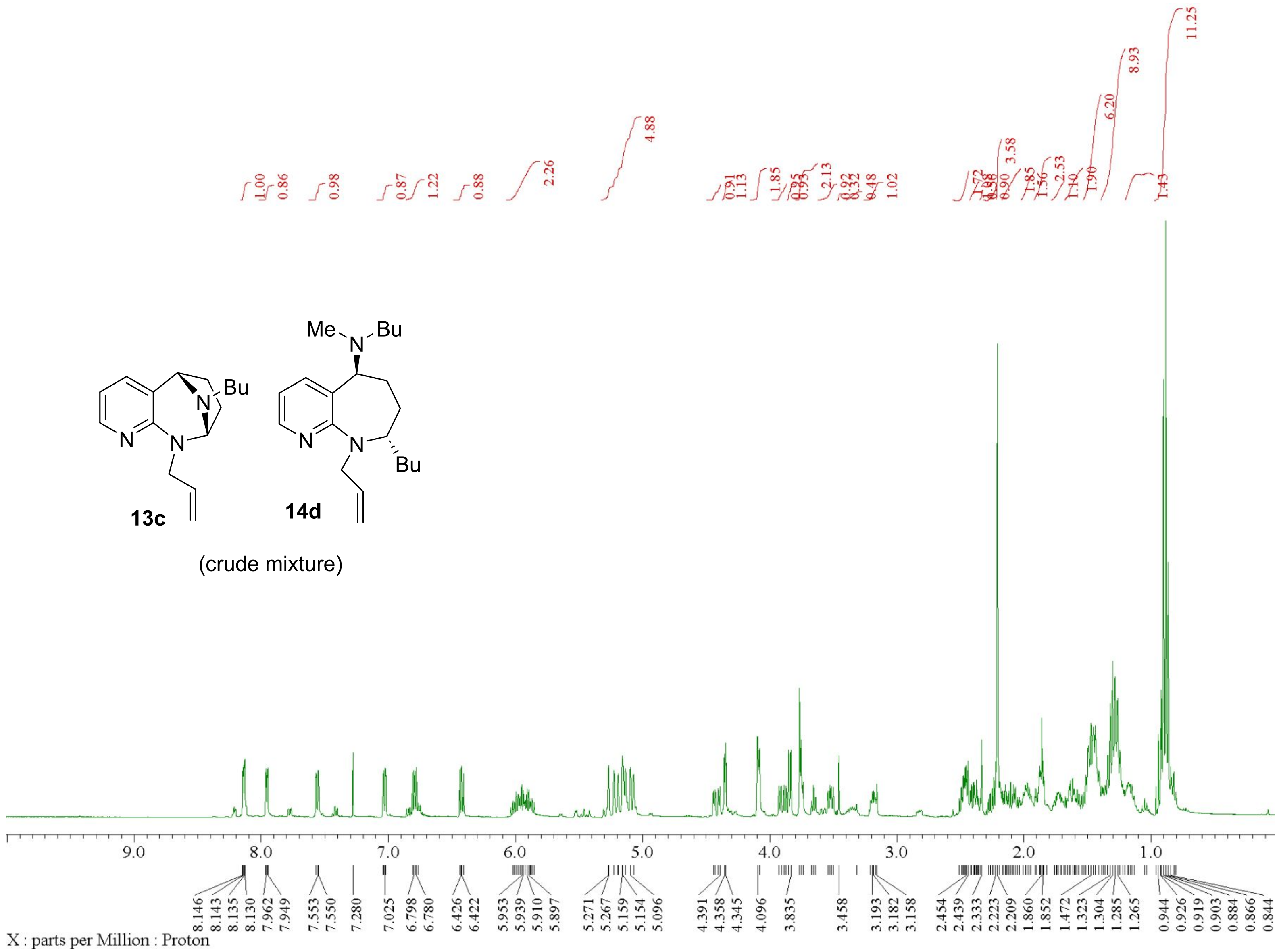

(crude mixture)

^{1}H NMR, 400 MHz, $CDCl_3$

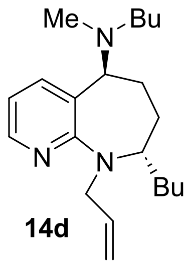

14d

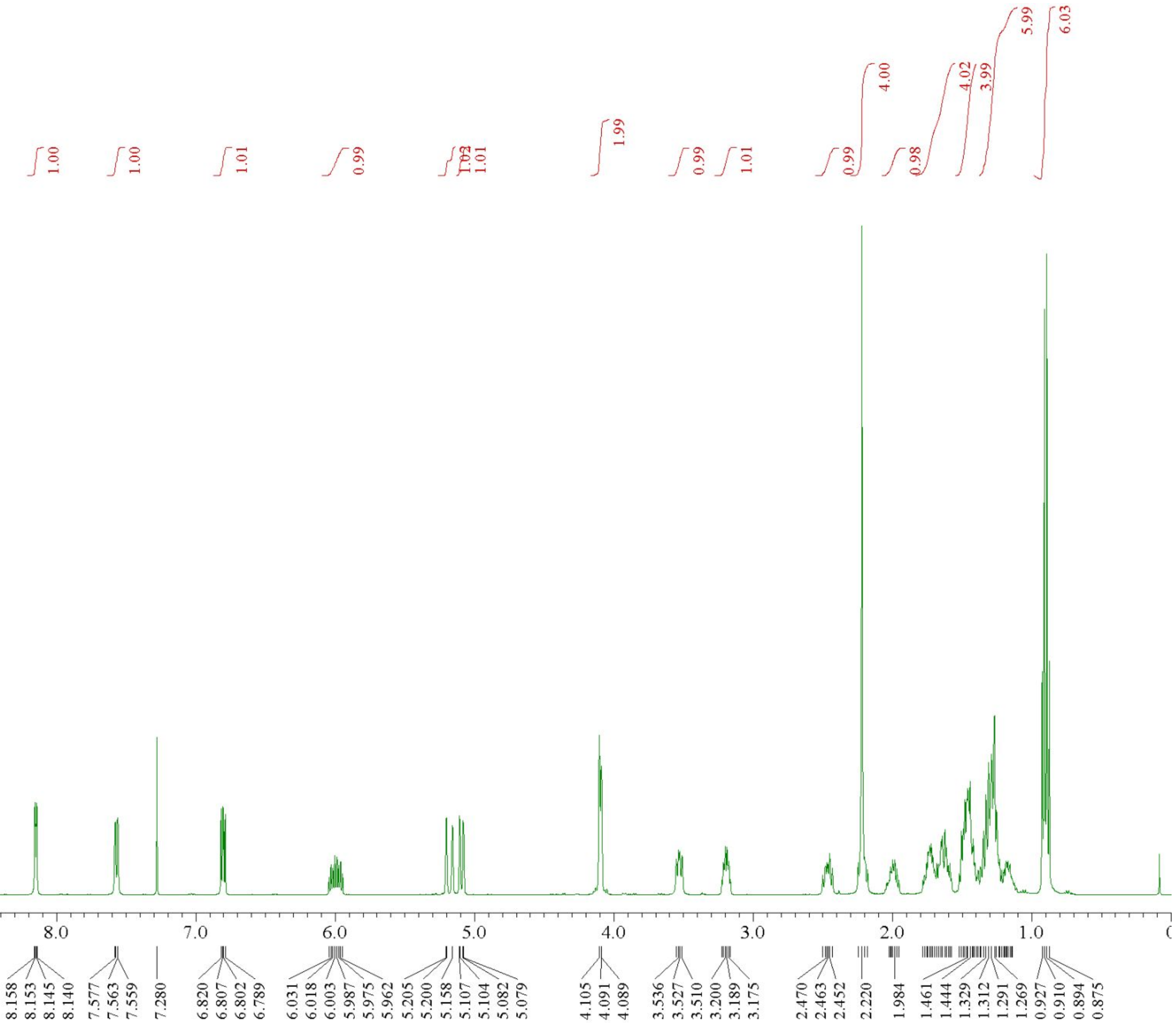

^{1}H NMR, 400 MHz, $CDCl_3$

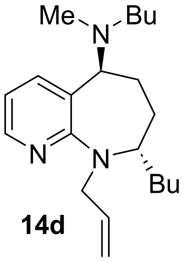

14d

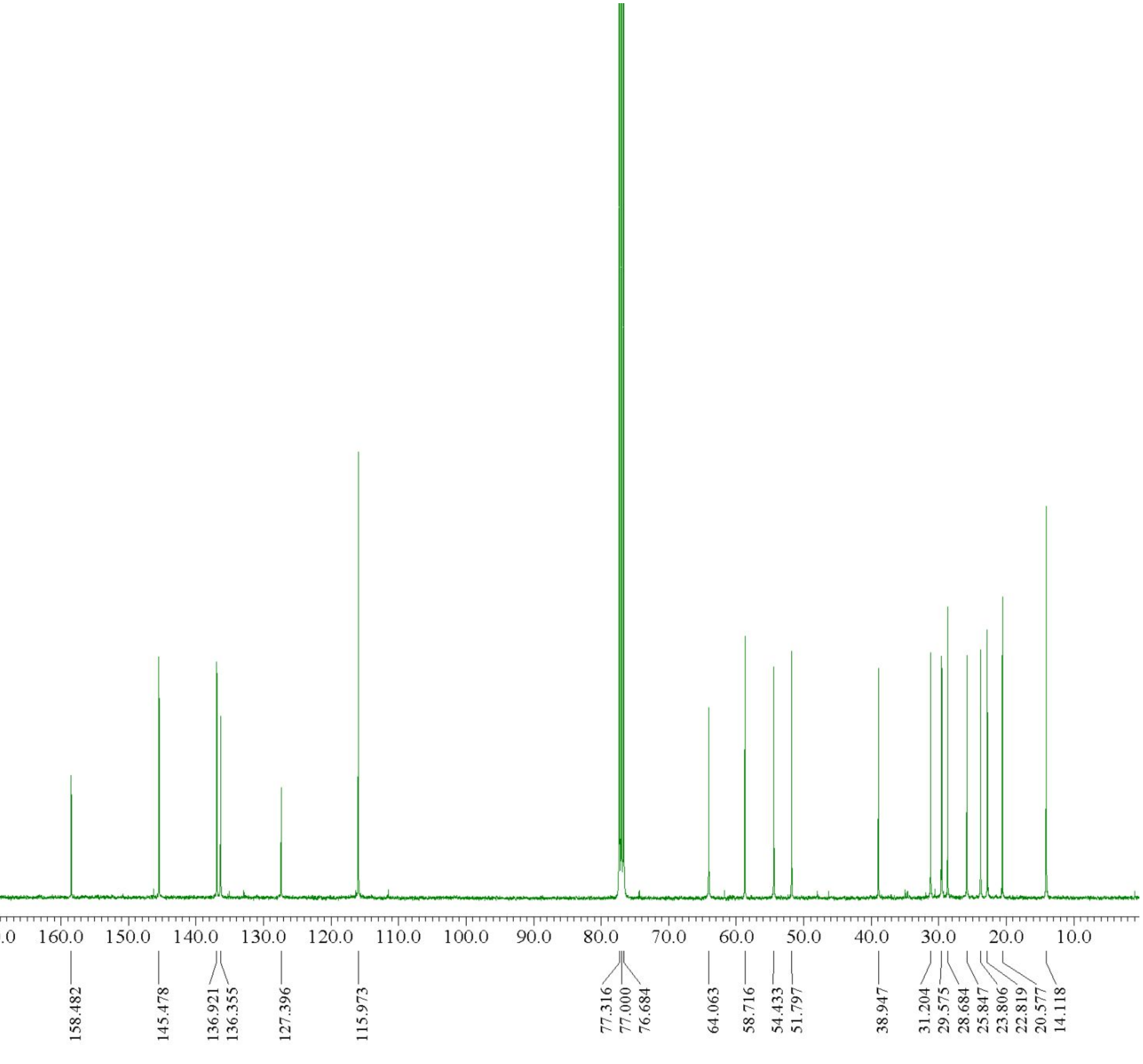

$^{13}C\{^1H\}$ NMR, 100 MHz, $CDCl_3$

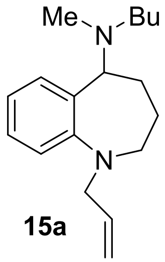

15a

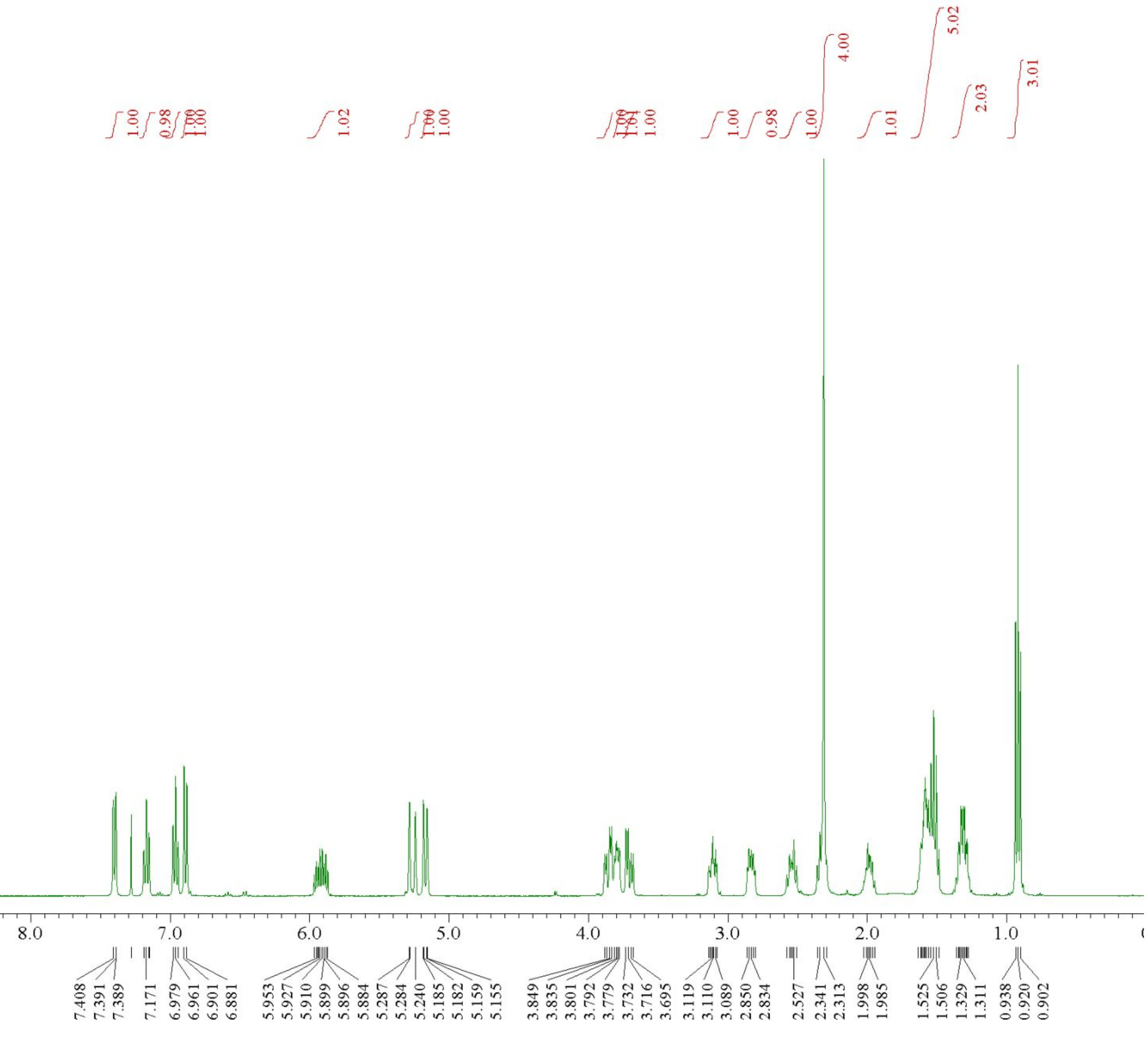

^{1}H NMR, 400 MHz, $CDCl_3$

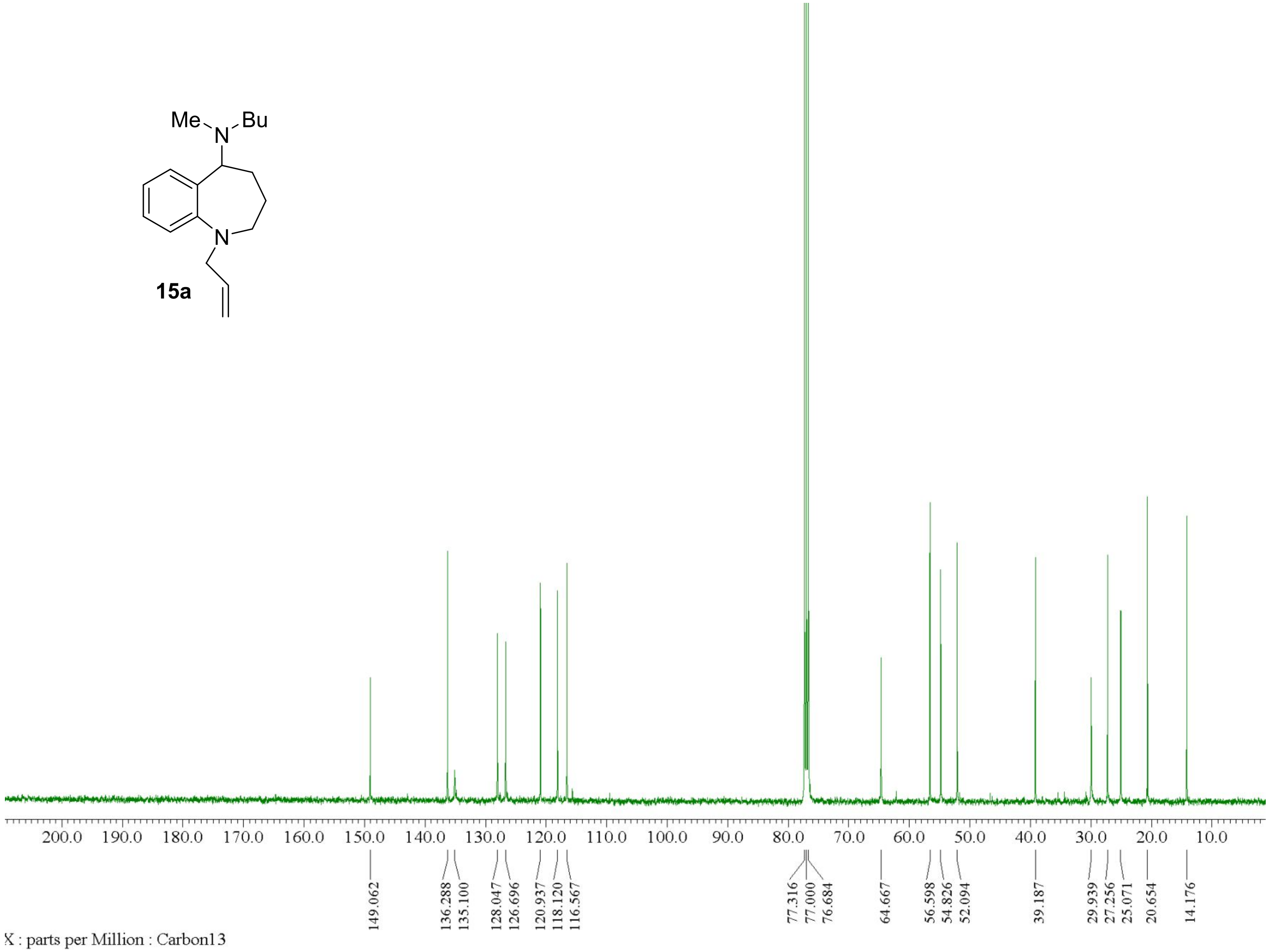

$^{13}C\{1H\}$ NMR, 100 MHz, $CDCl_3$

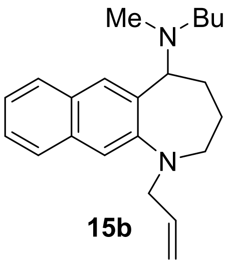

15b

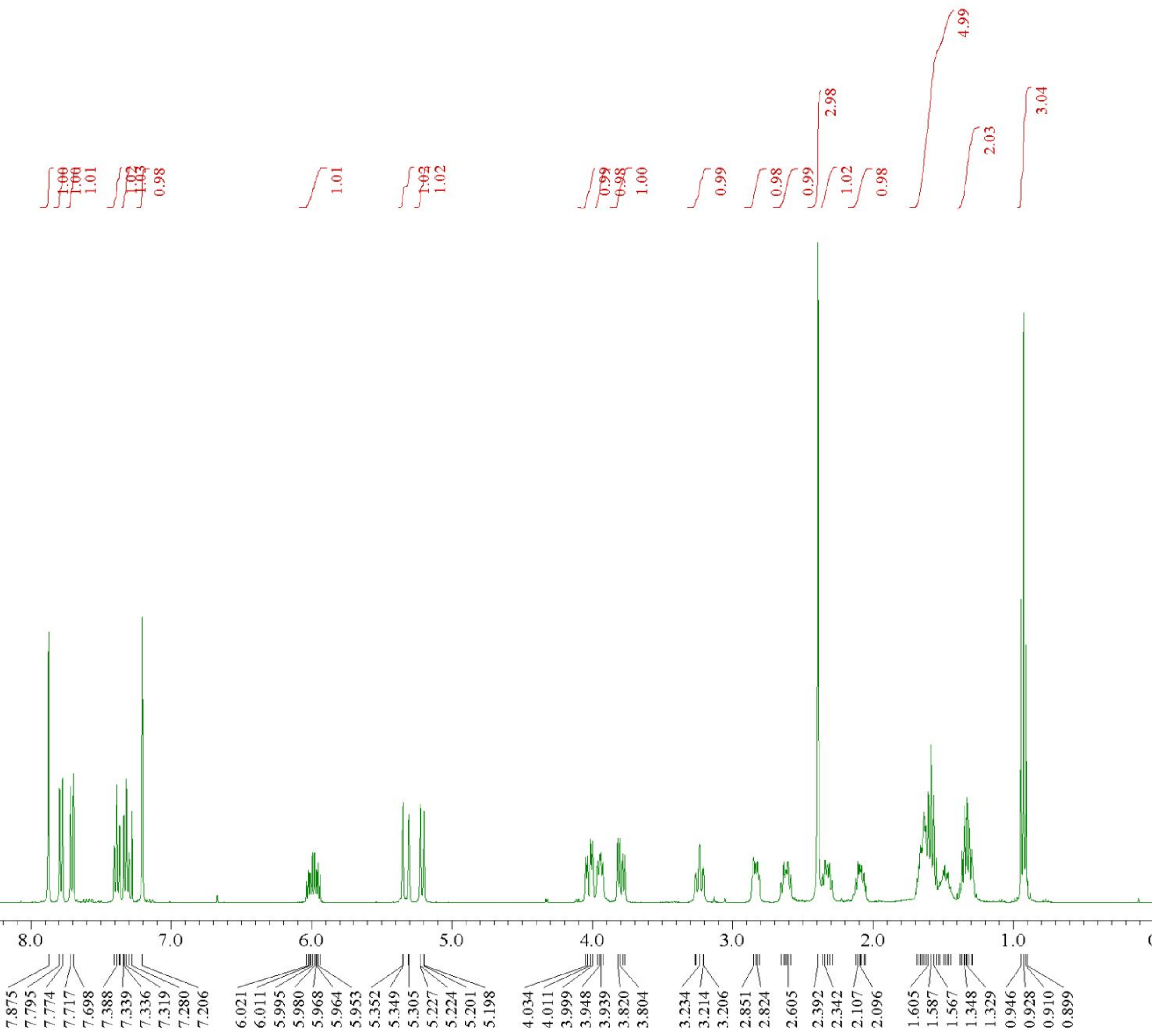

^{1}H NMR, 400 MHz, $CDCl_3$

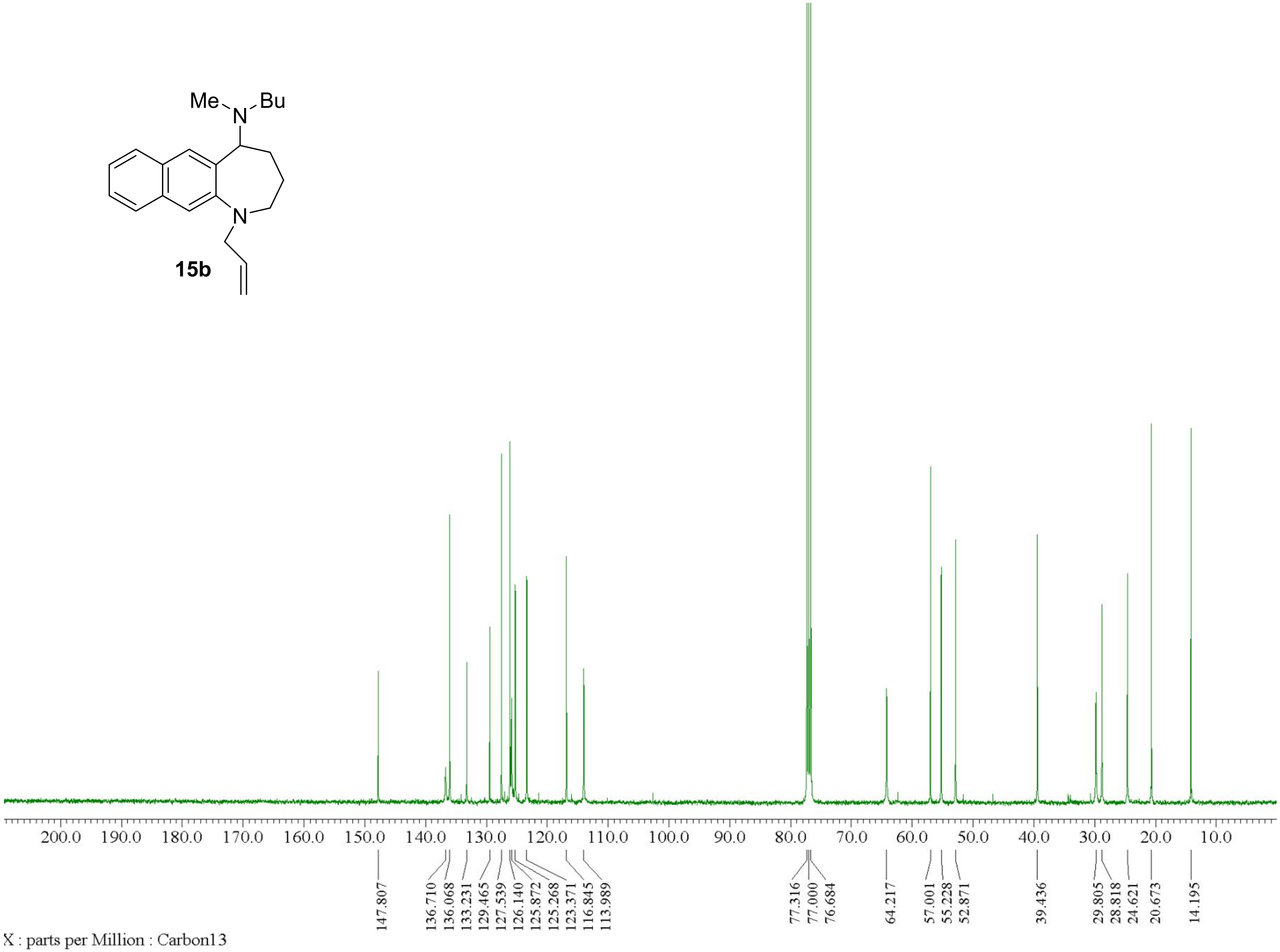

$^{13}C\{1H\}$ NMR, 100 MHz, $CDCl_3$

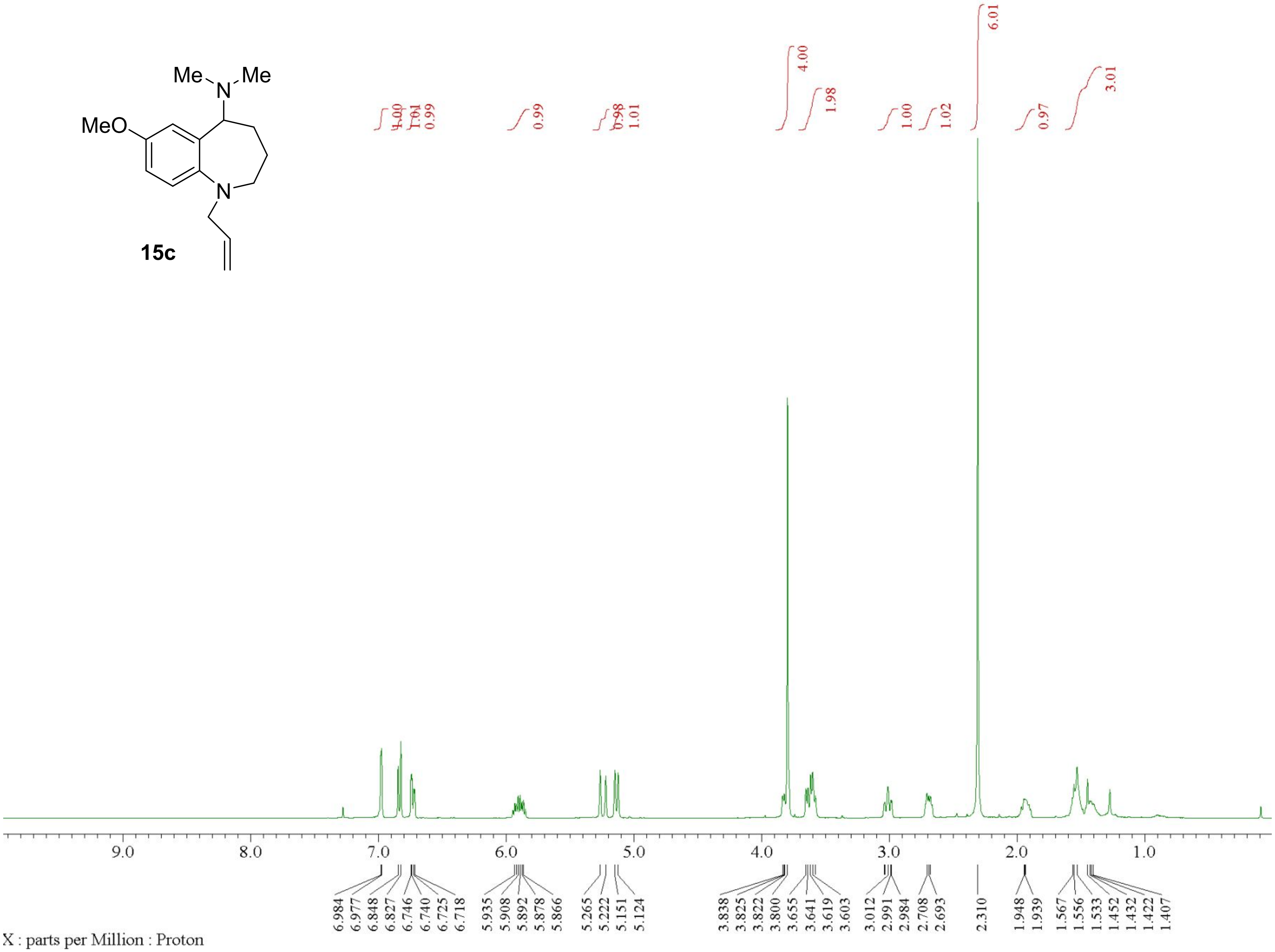

^{1}H NMR, 400 MHz, $CDCl_3$

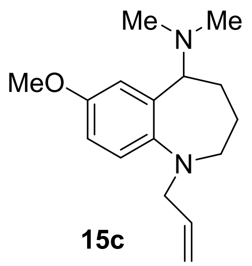

15c

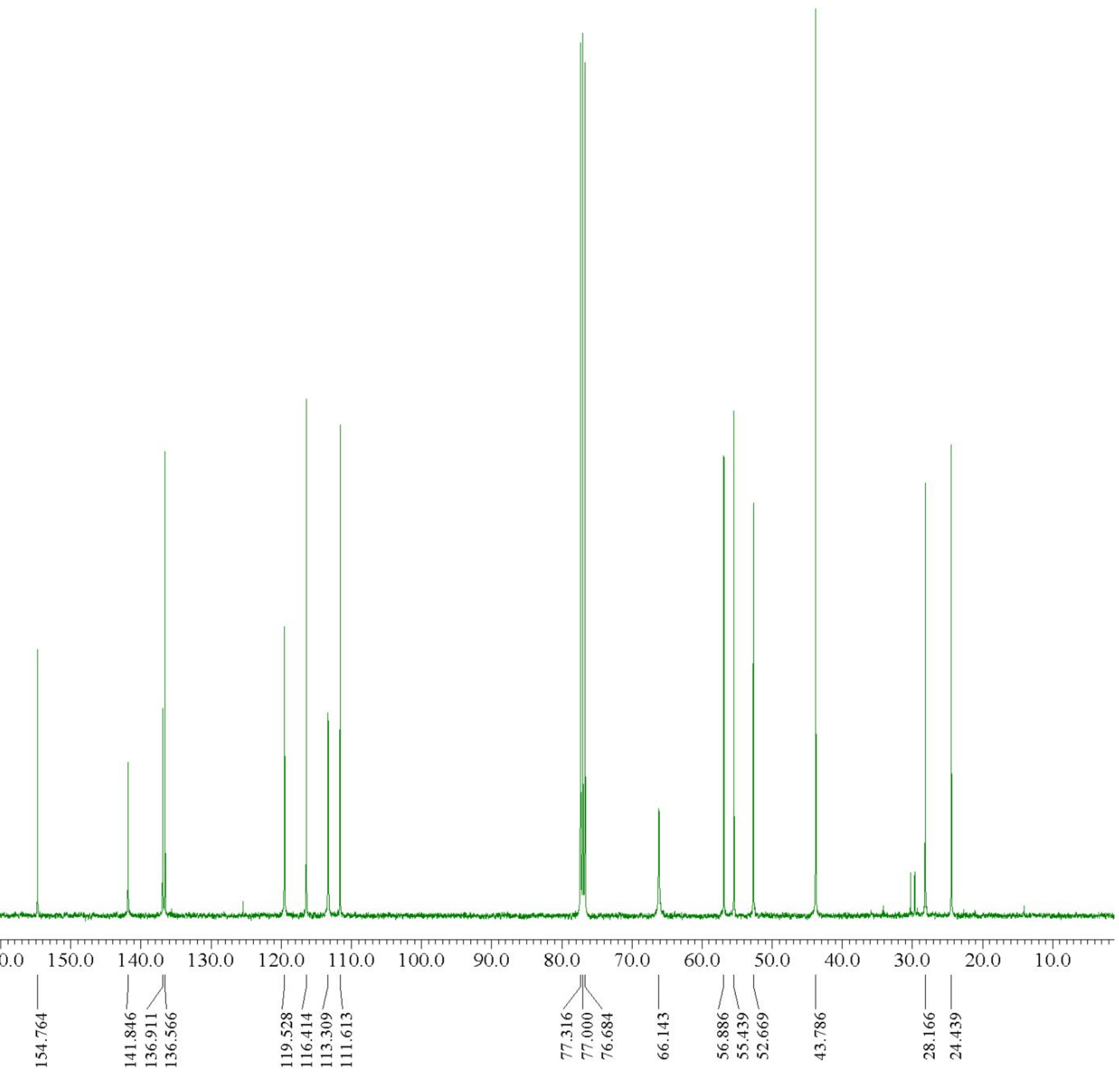

$^{13}C\{1H\}$ NMR, 100 MHz, $CDCl_3$

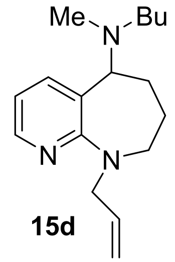

15d

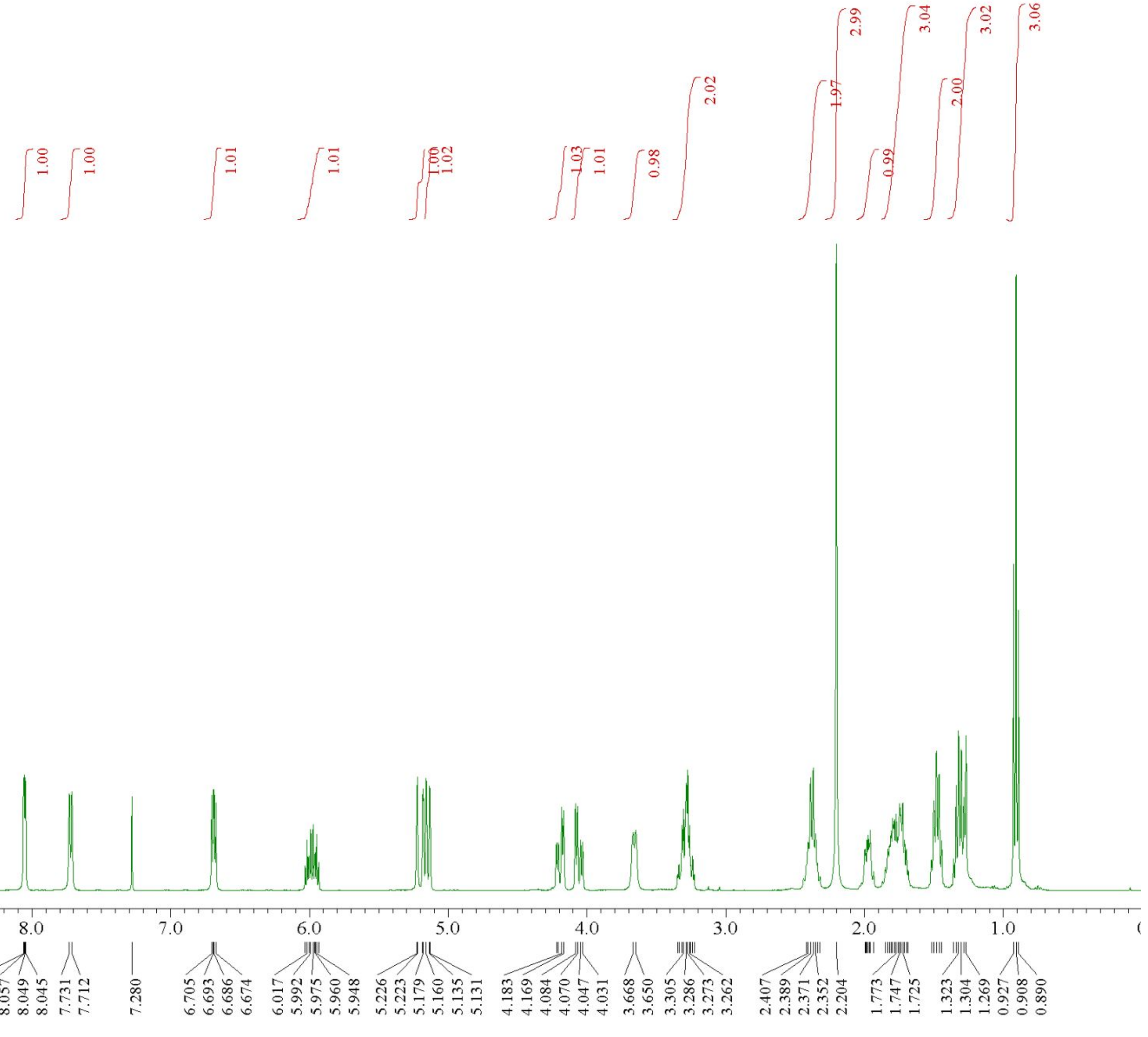

^{1}H NMR, 400 MHz, $CDCl_3$

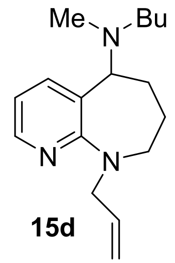

15d

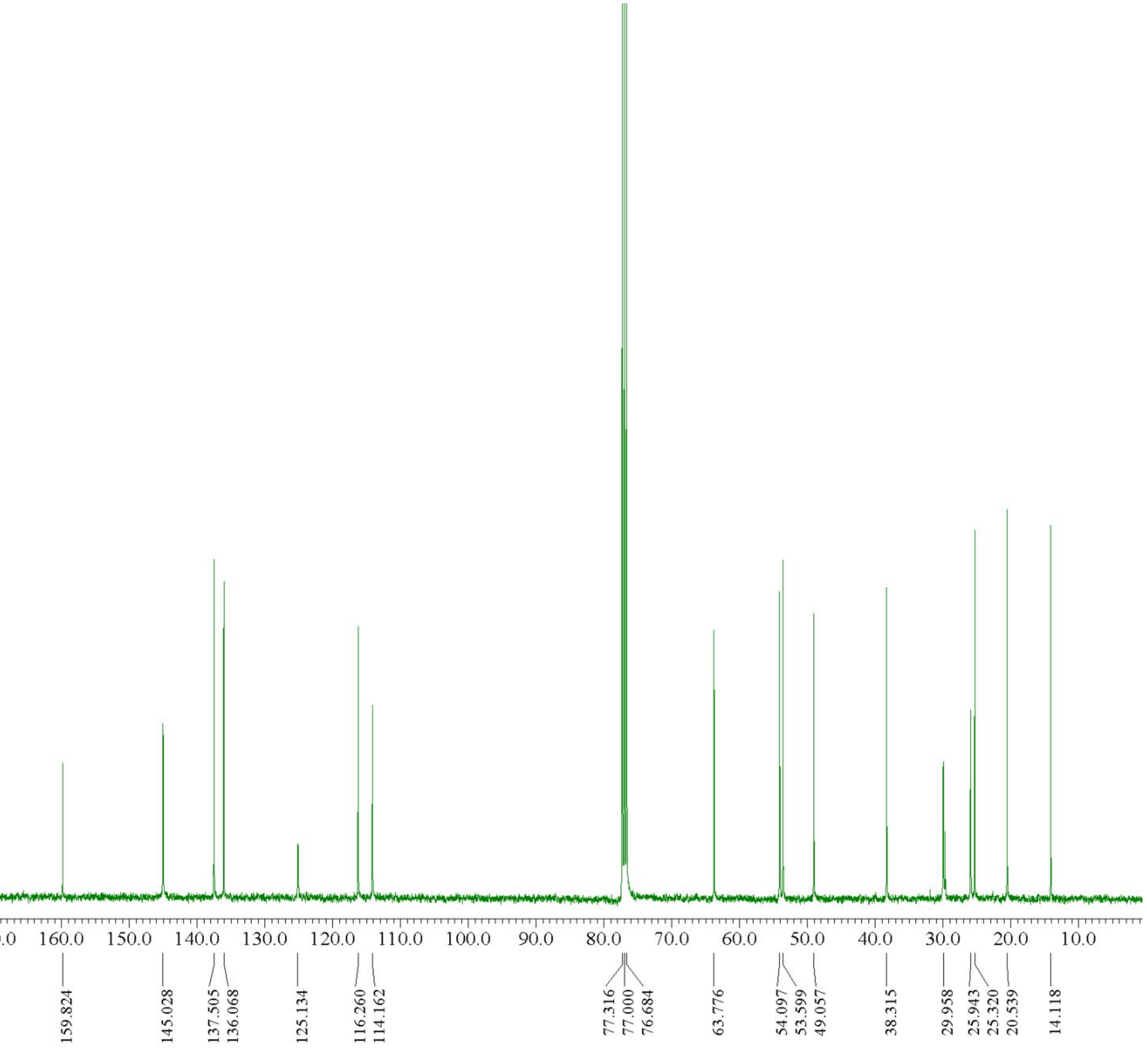

$^{13}C\{^1H\}$ NMR, 100 MHz, $CDCl_3$

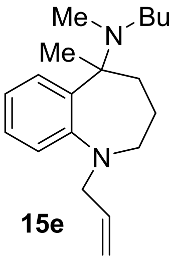

15e

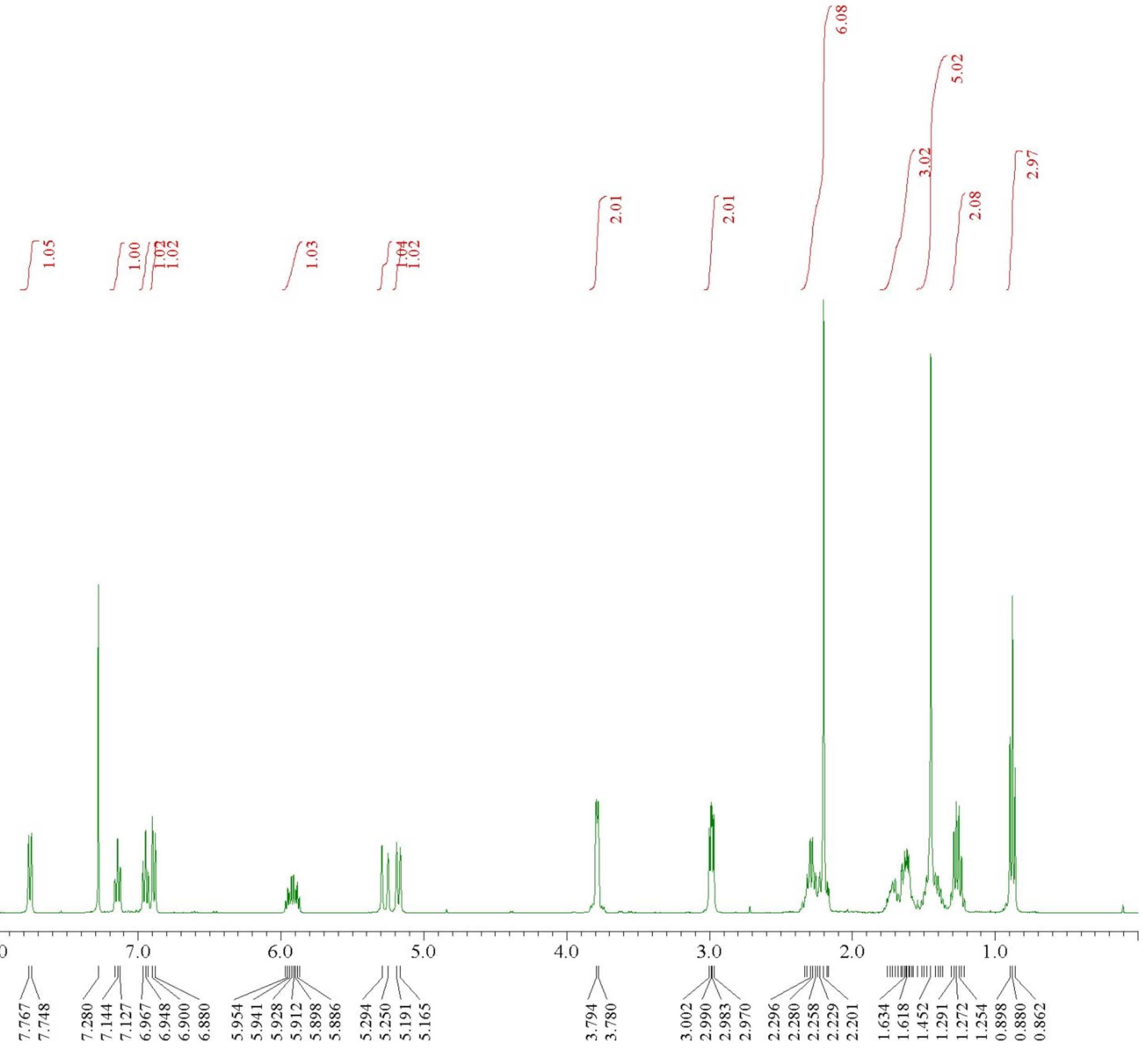

^{1}H NMR, 400 MHz, $CDCl_3$

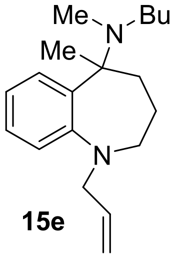

15e

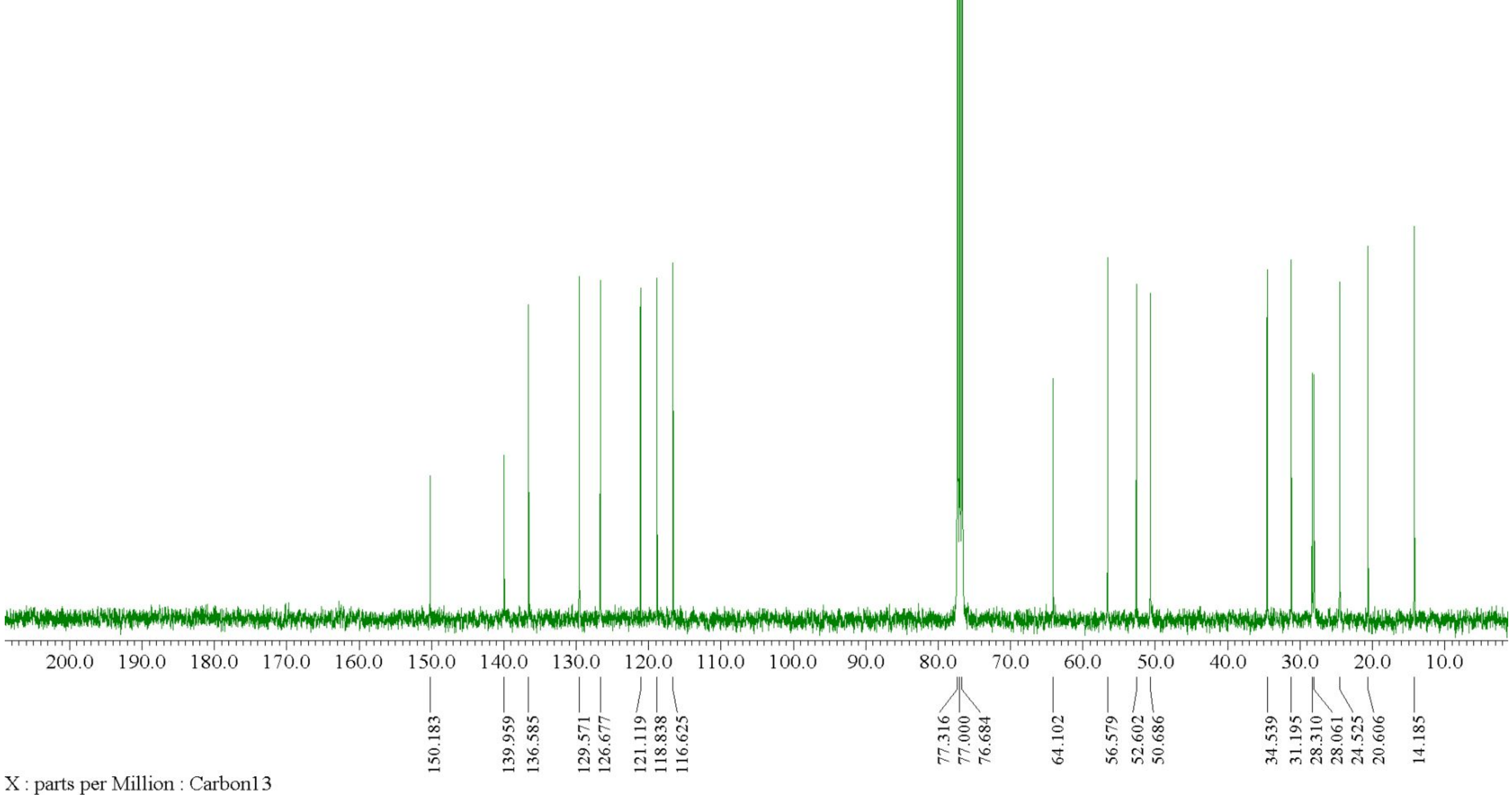

$^{13}C\{^1H\}$ NMR, 100 MHz, $CDCl_3$

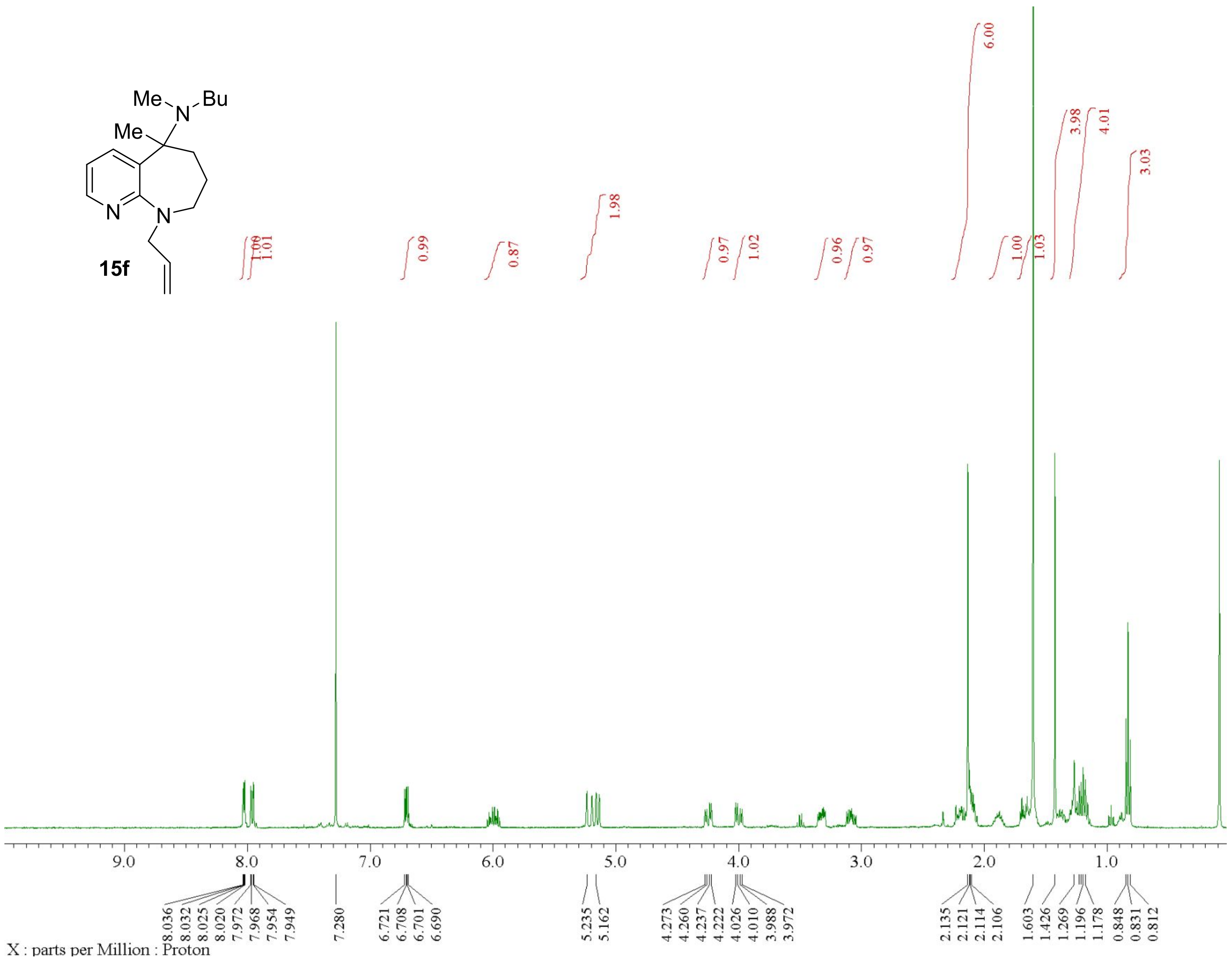

¹H NMR, 400 MHz, $CDCl_3$

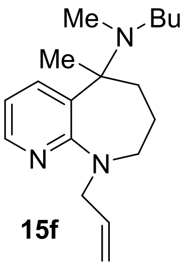

15f

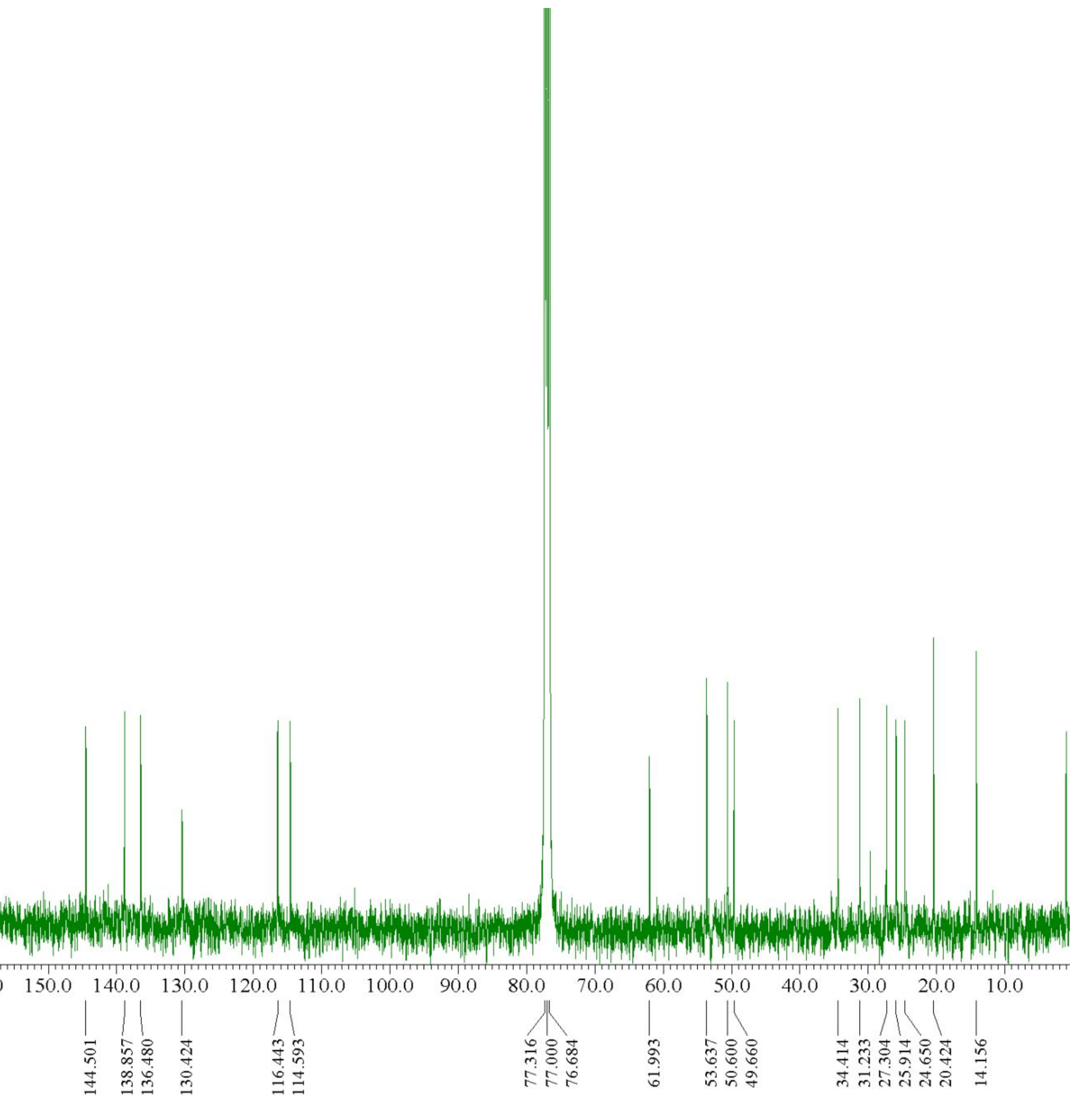

$^{13}C\{1H\}$ NMR, 100 MHz, $CDCl_3$

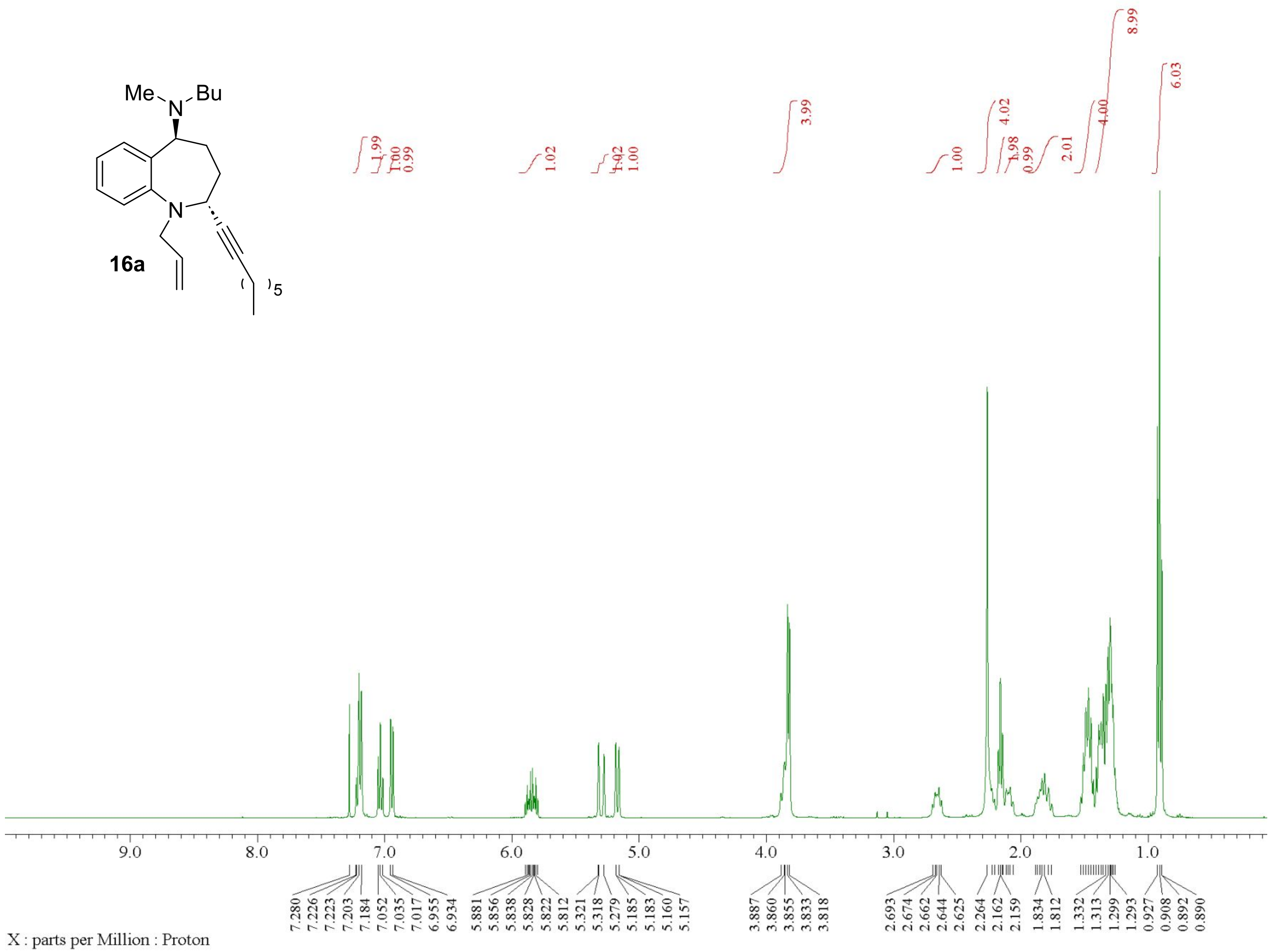

^{1}H NMR, 400 MHz, $CDCl_3$

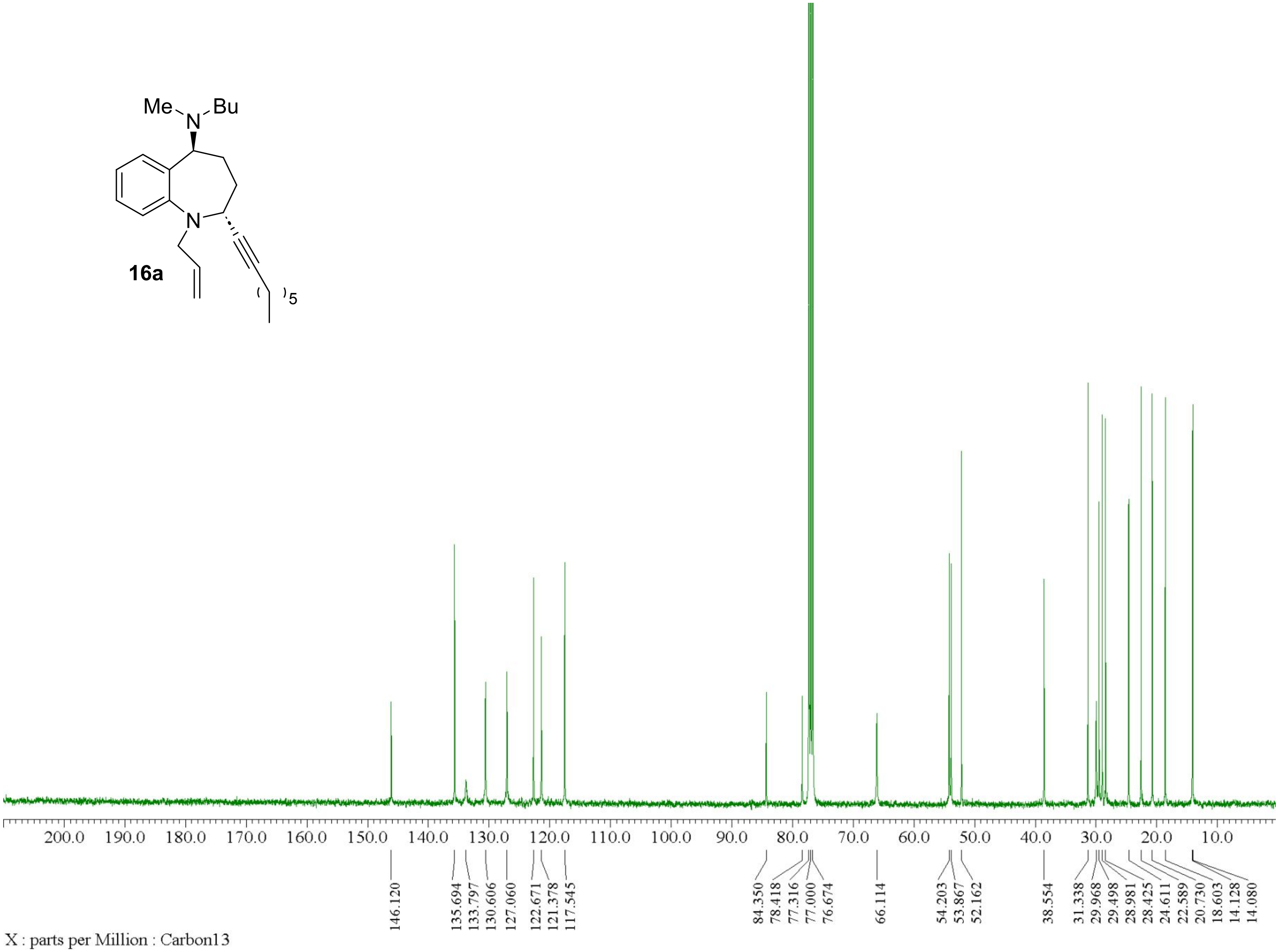

$^{13}C\{^1H\}$ NMR, 100 MHz, $CDCl_3$

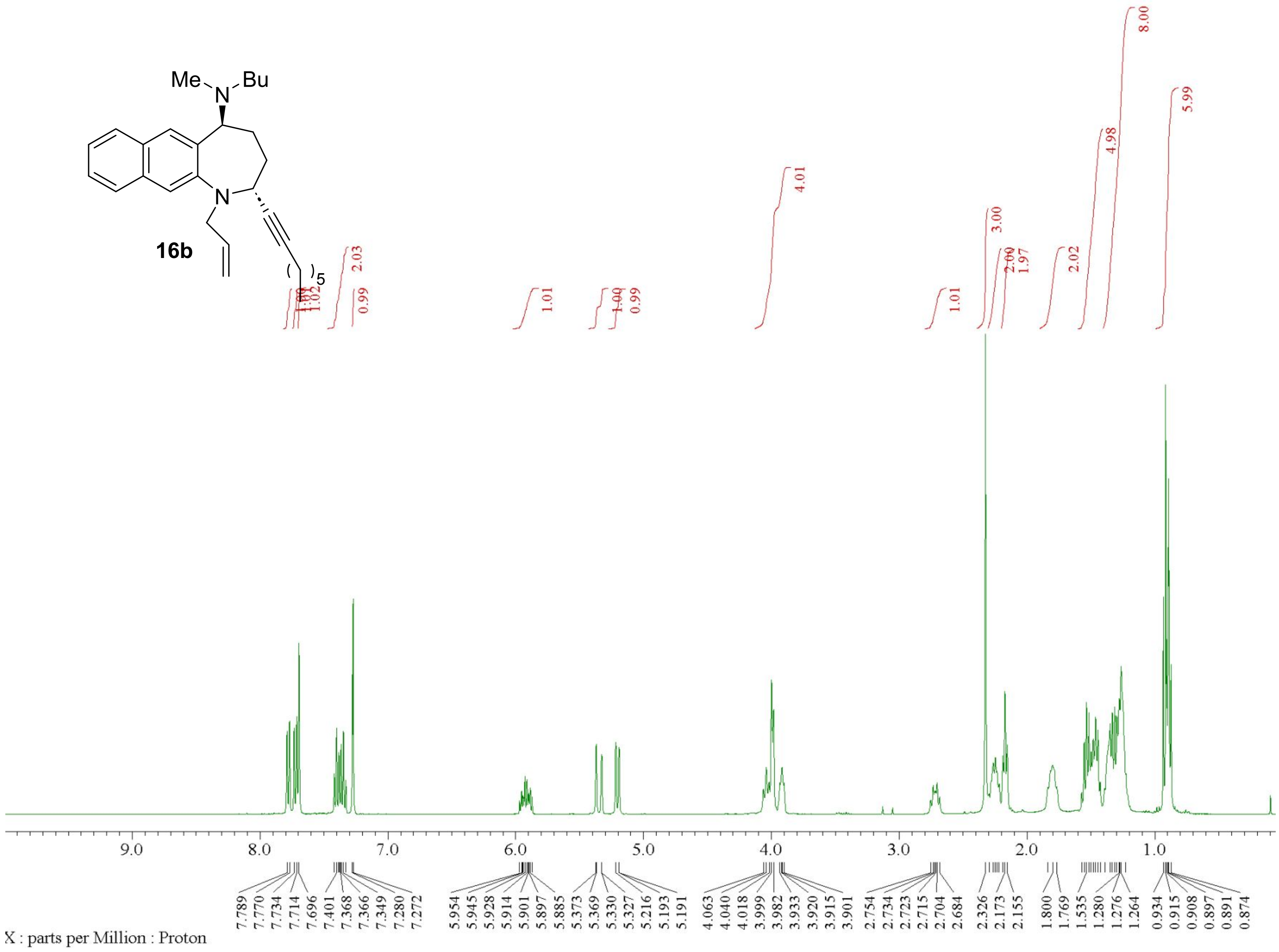

^1H NMR, 400 MHz, $CDCl_3$

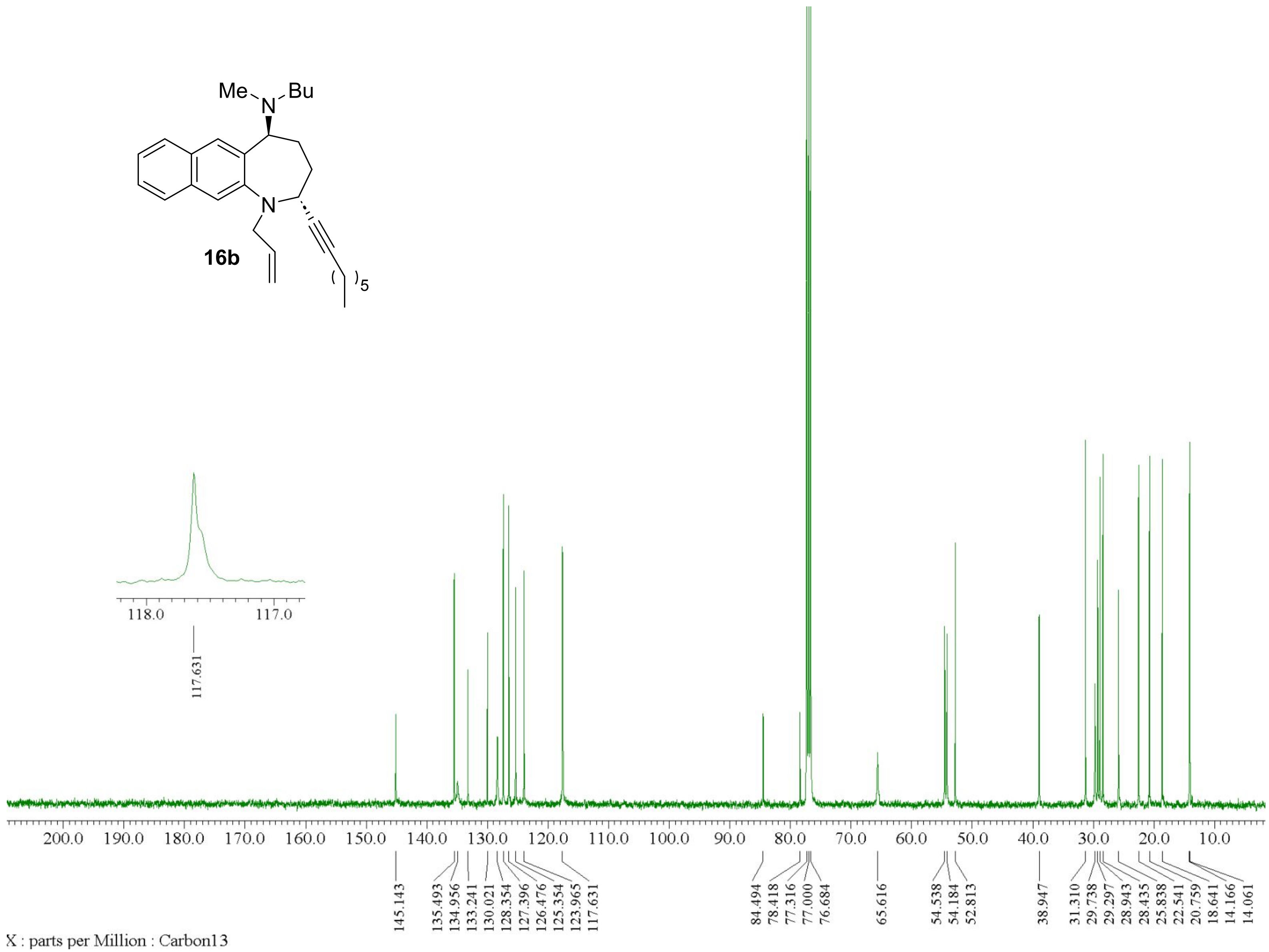

$^{13}C\{^1H\}$ NMR, 100 MHz, $CDCl_3$

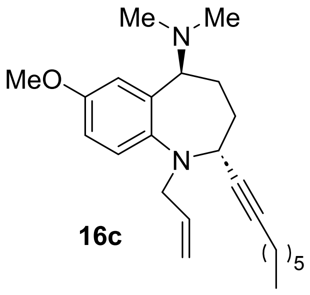

16c

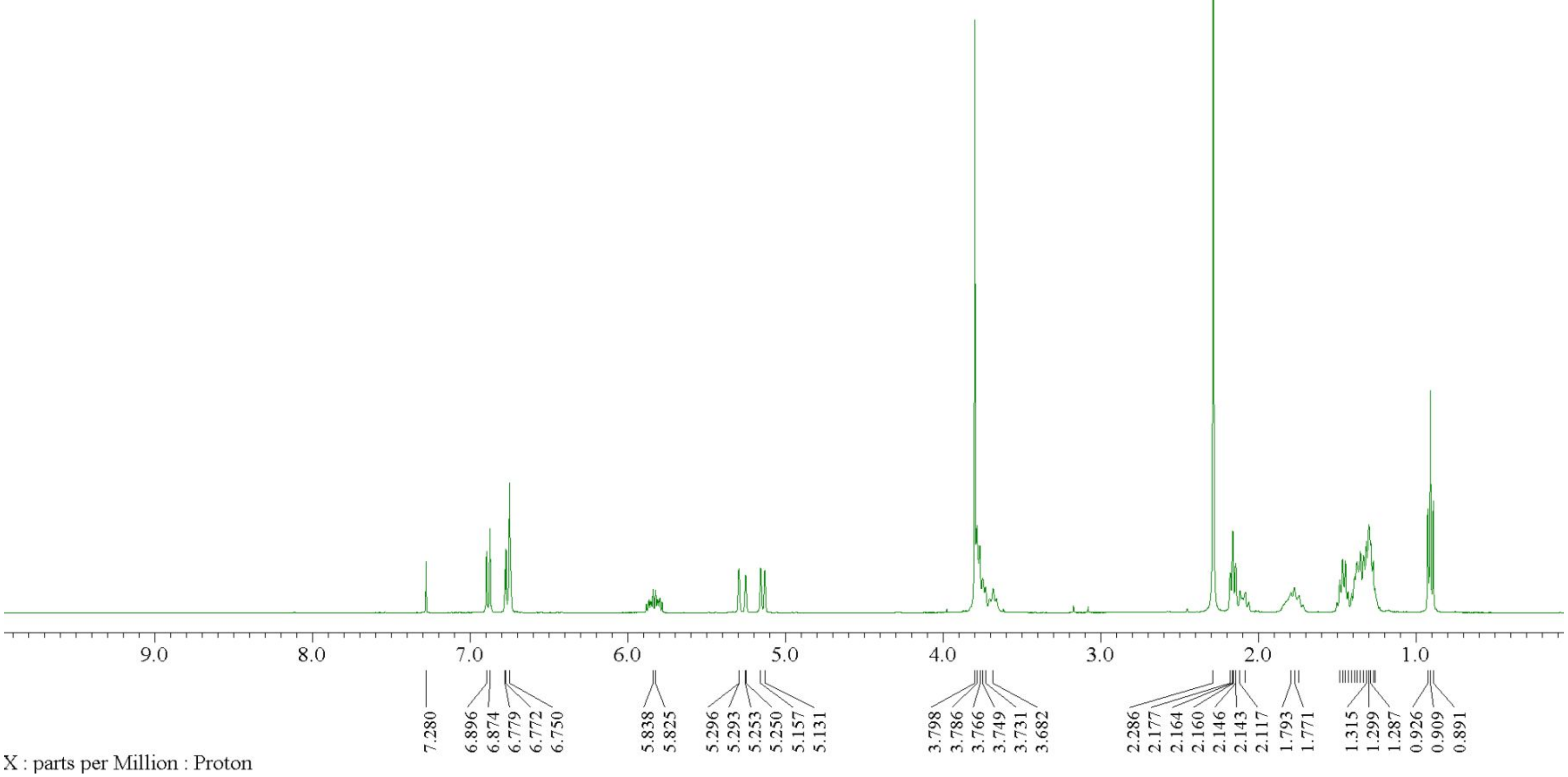

^{1}H NMR, 400 MHz, $CDCl_3$

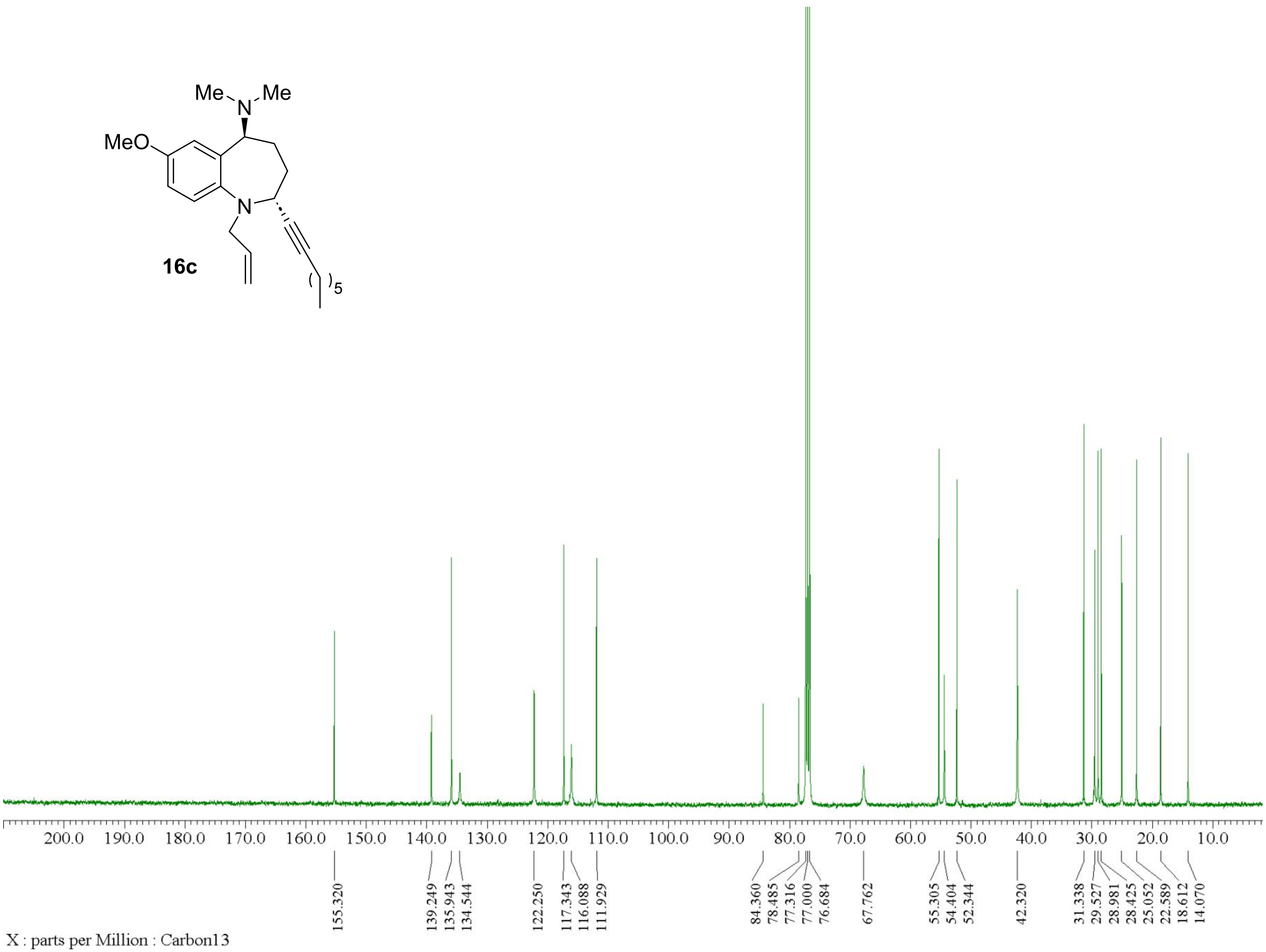

$^{13}C\{1H\}$ NMR, 100 MHz, $CDCl_3$

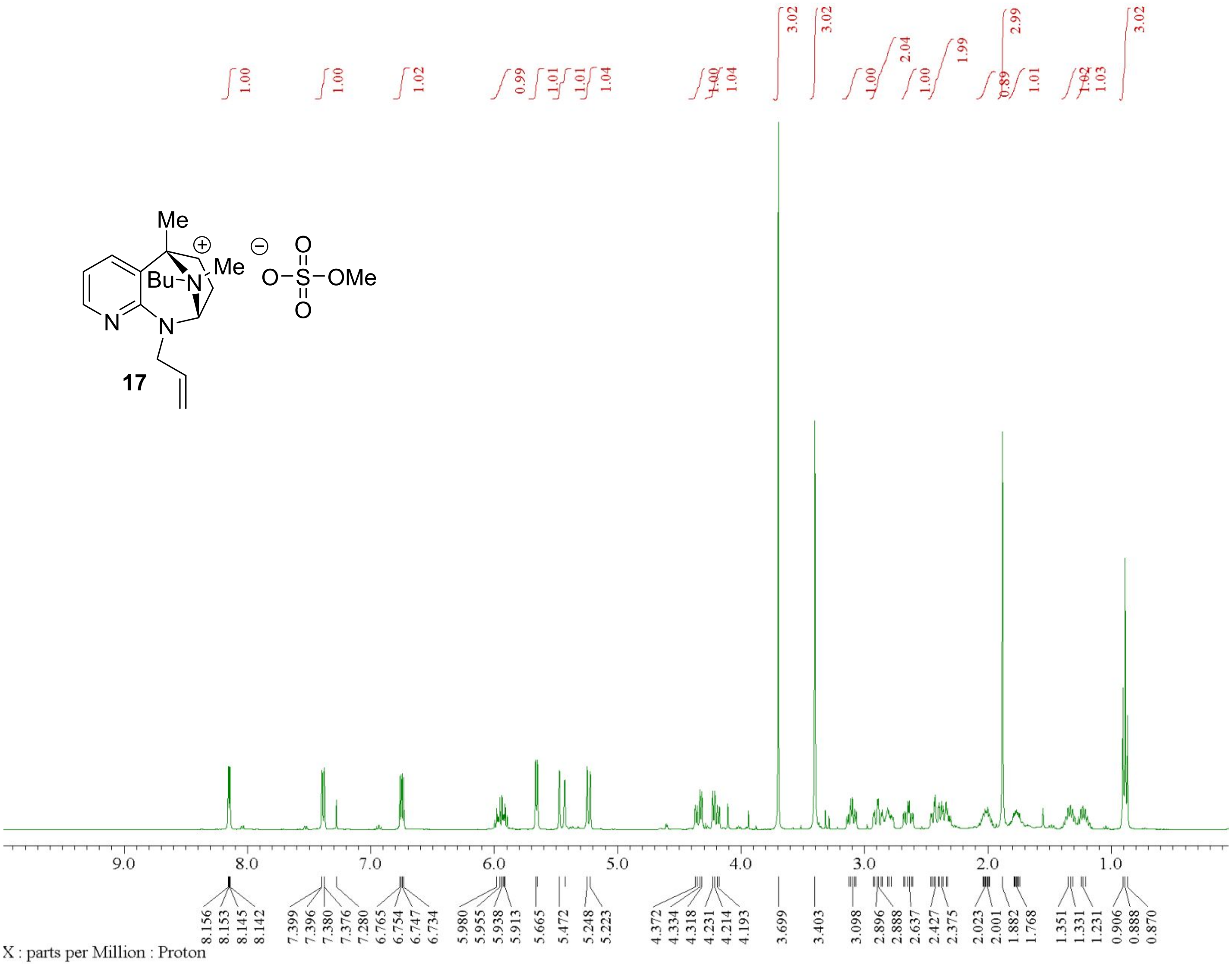

^{1}H NMR, 400 MHz, $CDCl_3$

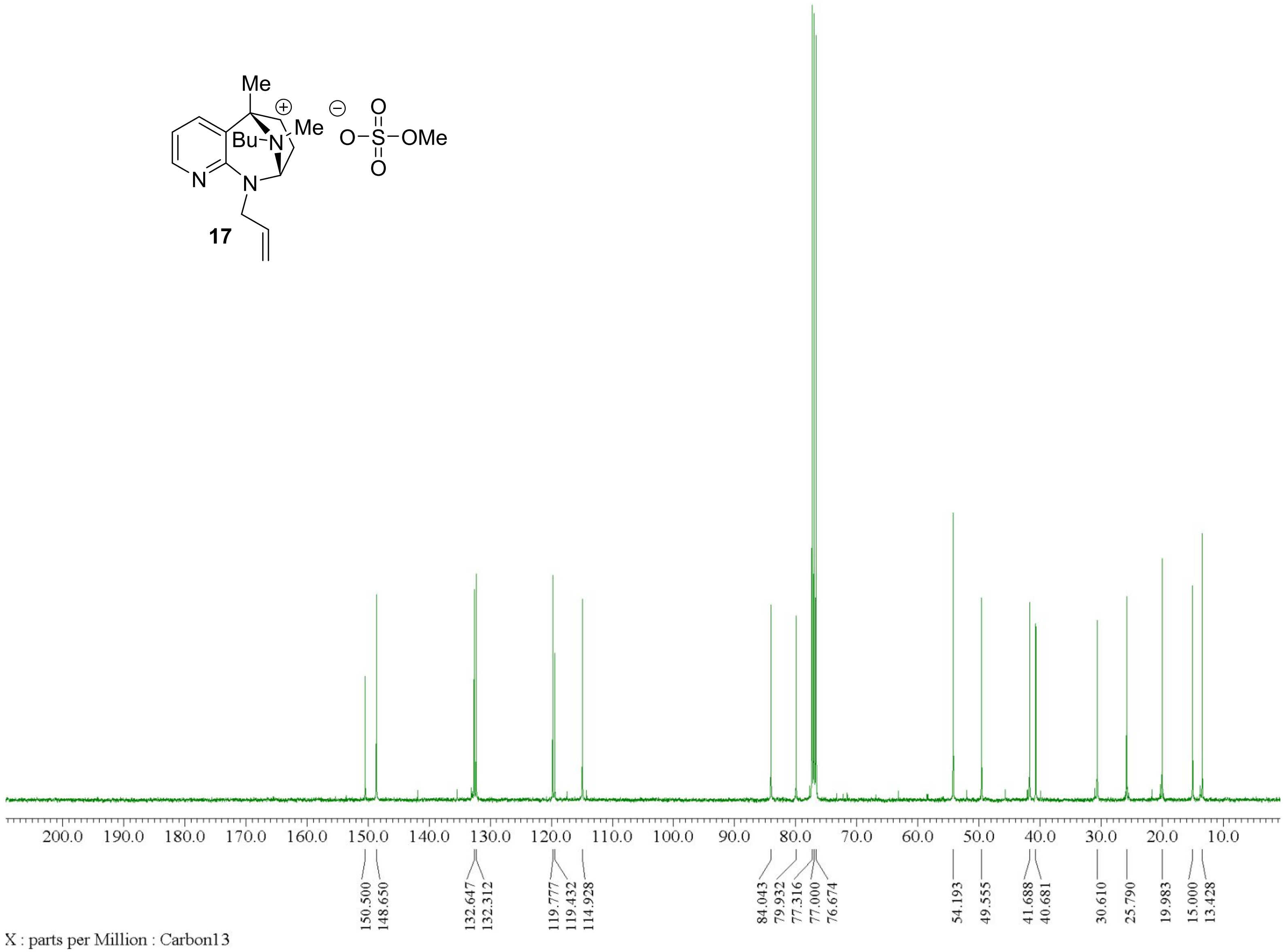

17

$^{13}C\{^1H\}$ NMR, 100 MHz, $CDCl_3$

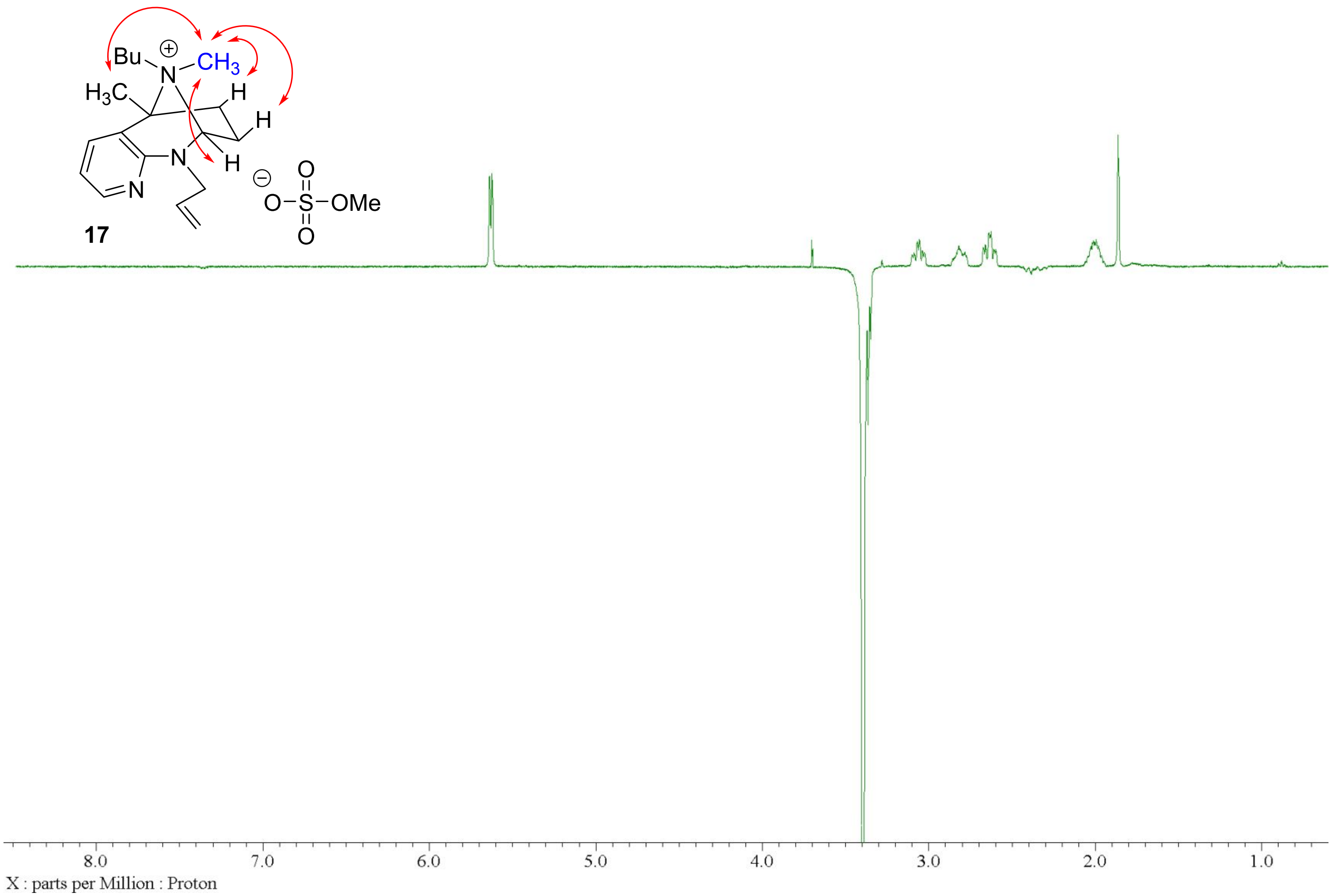

nOe Difference Spectrum (irrad Me at 3.40 ppm, diagnostic enhancements indicated)

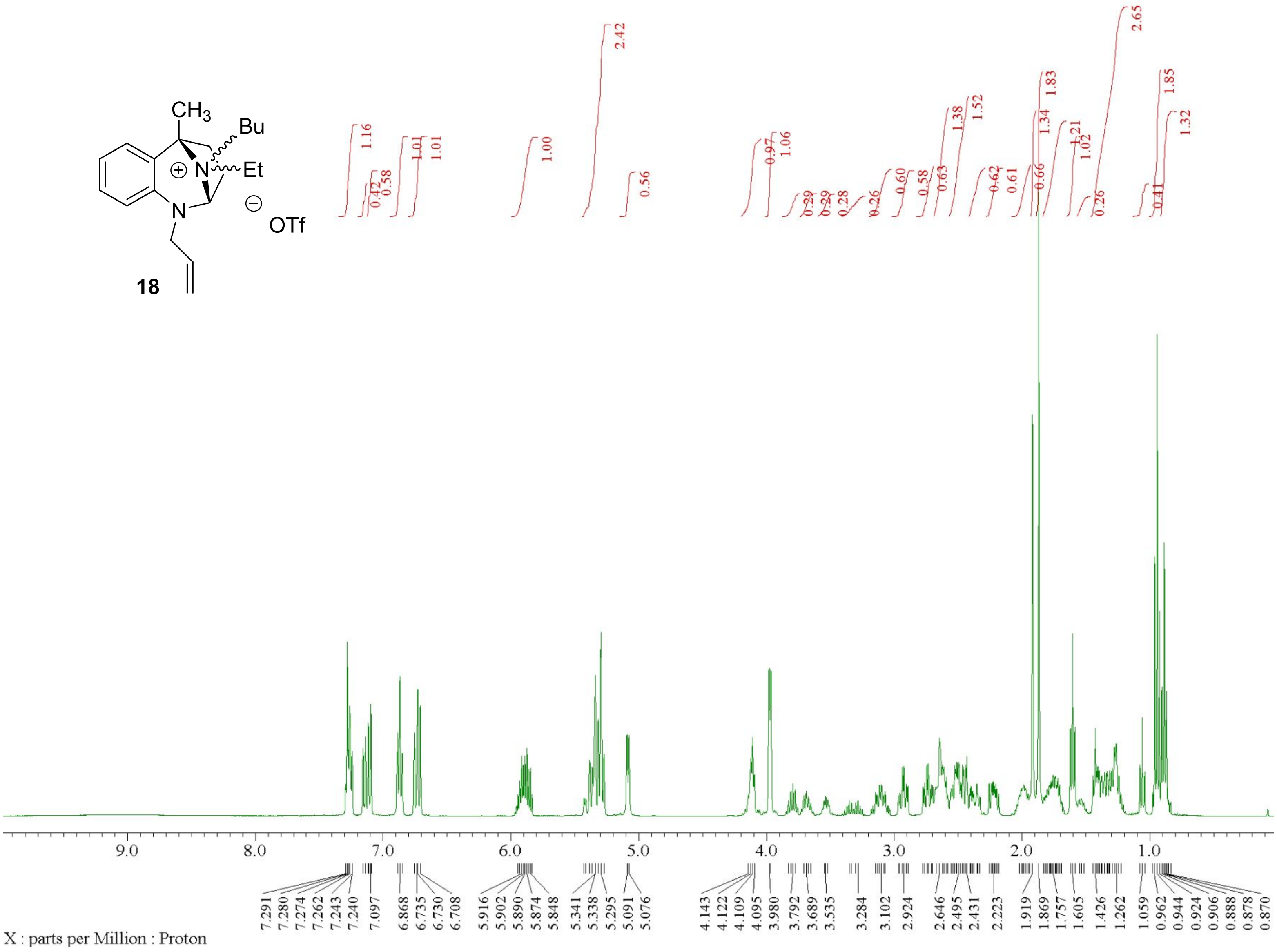

^1H NMR, 400 MHz, $CDCl_3$

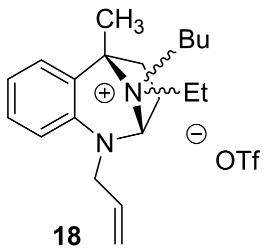

18

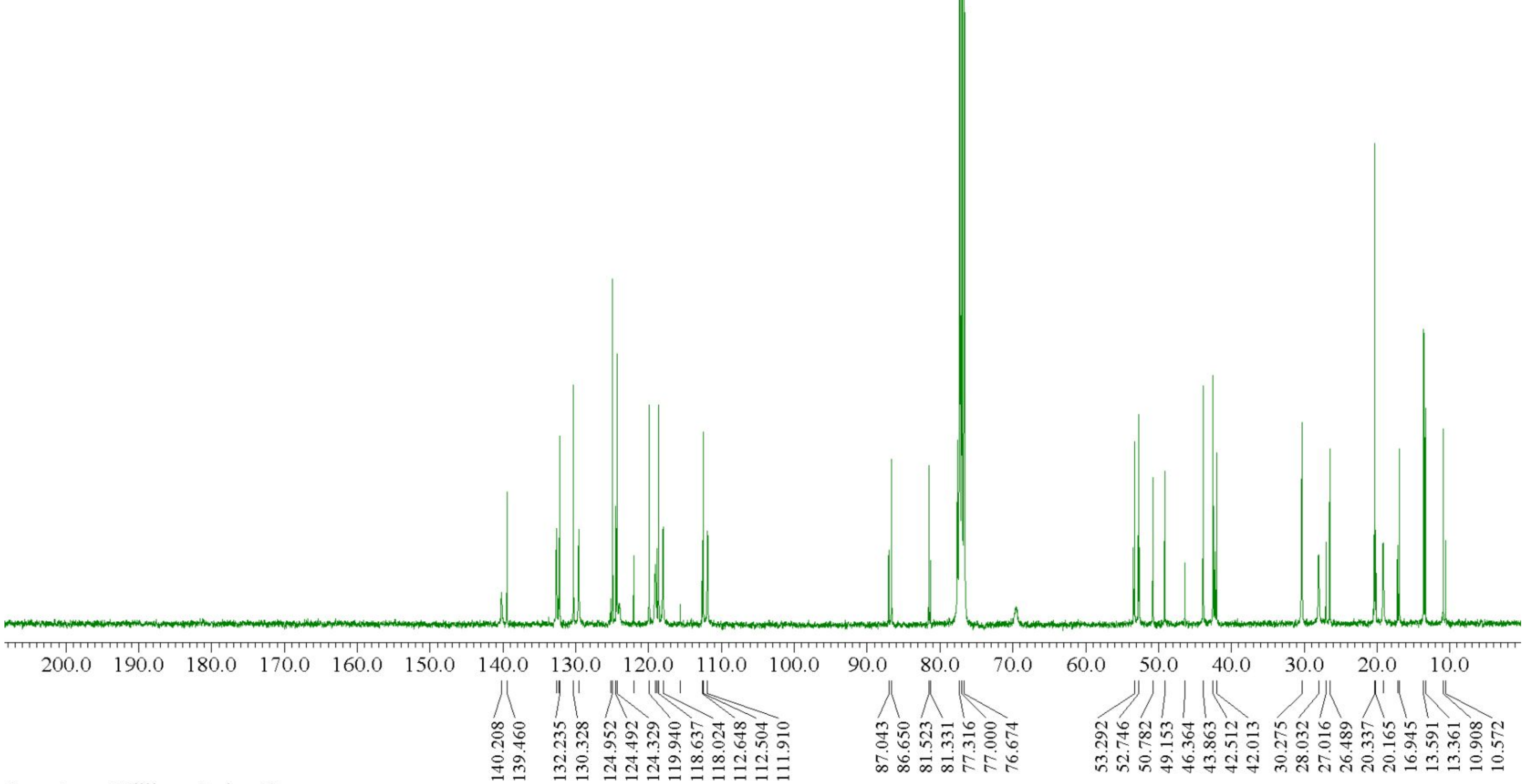

$^{13}C\{1H\}$ NMR, 100 MHz, $CDCl_3$

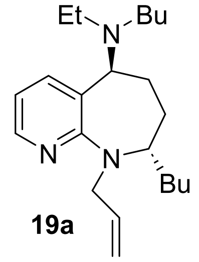

19a

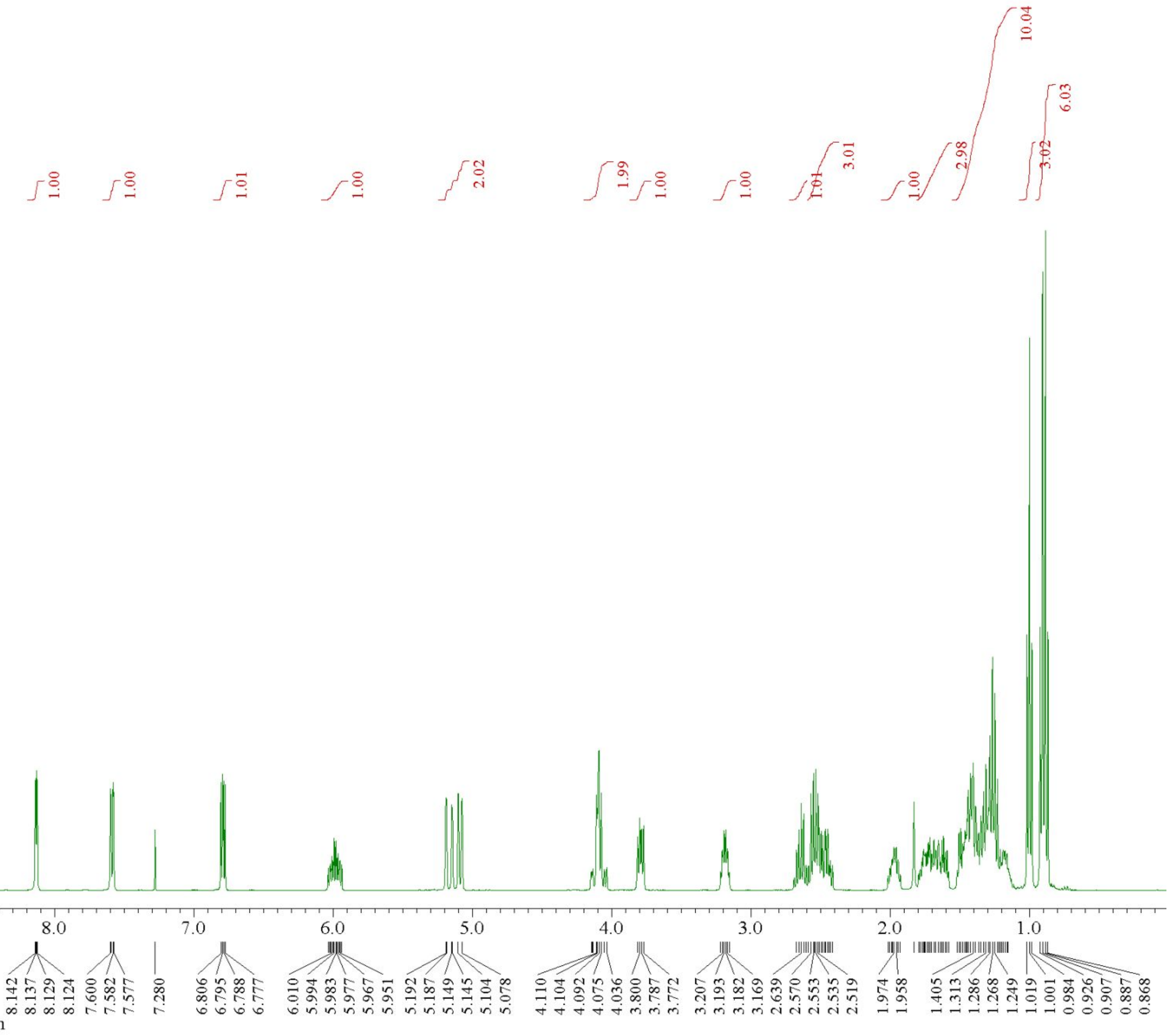

^{1}H NMR, 400 MHz, $CDCl_3$

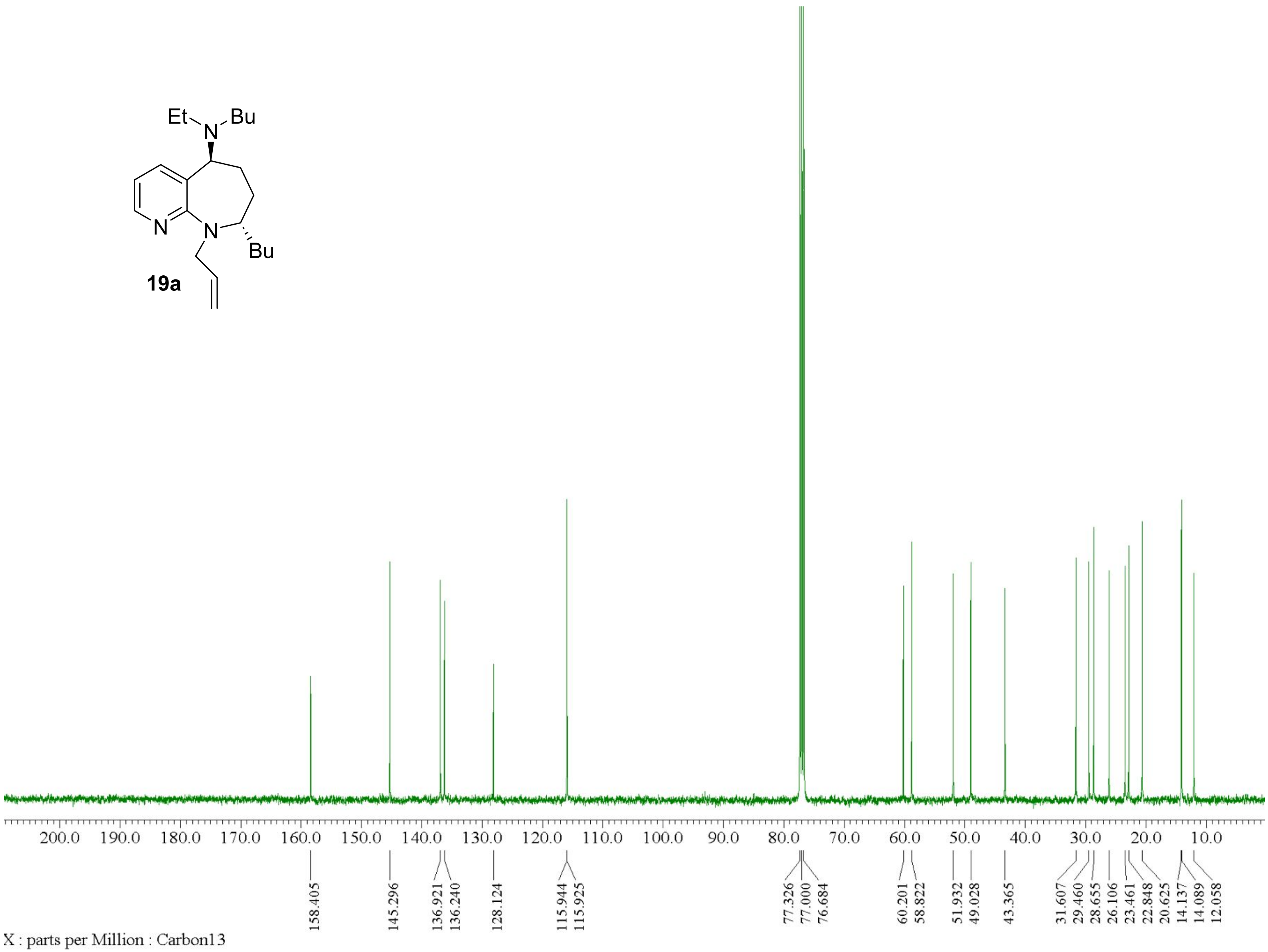

19a

$^{13}C\{^1H\}$ NMR, 100 MHz, $CDCl_3$

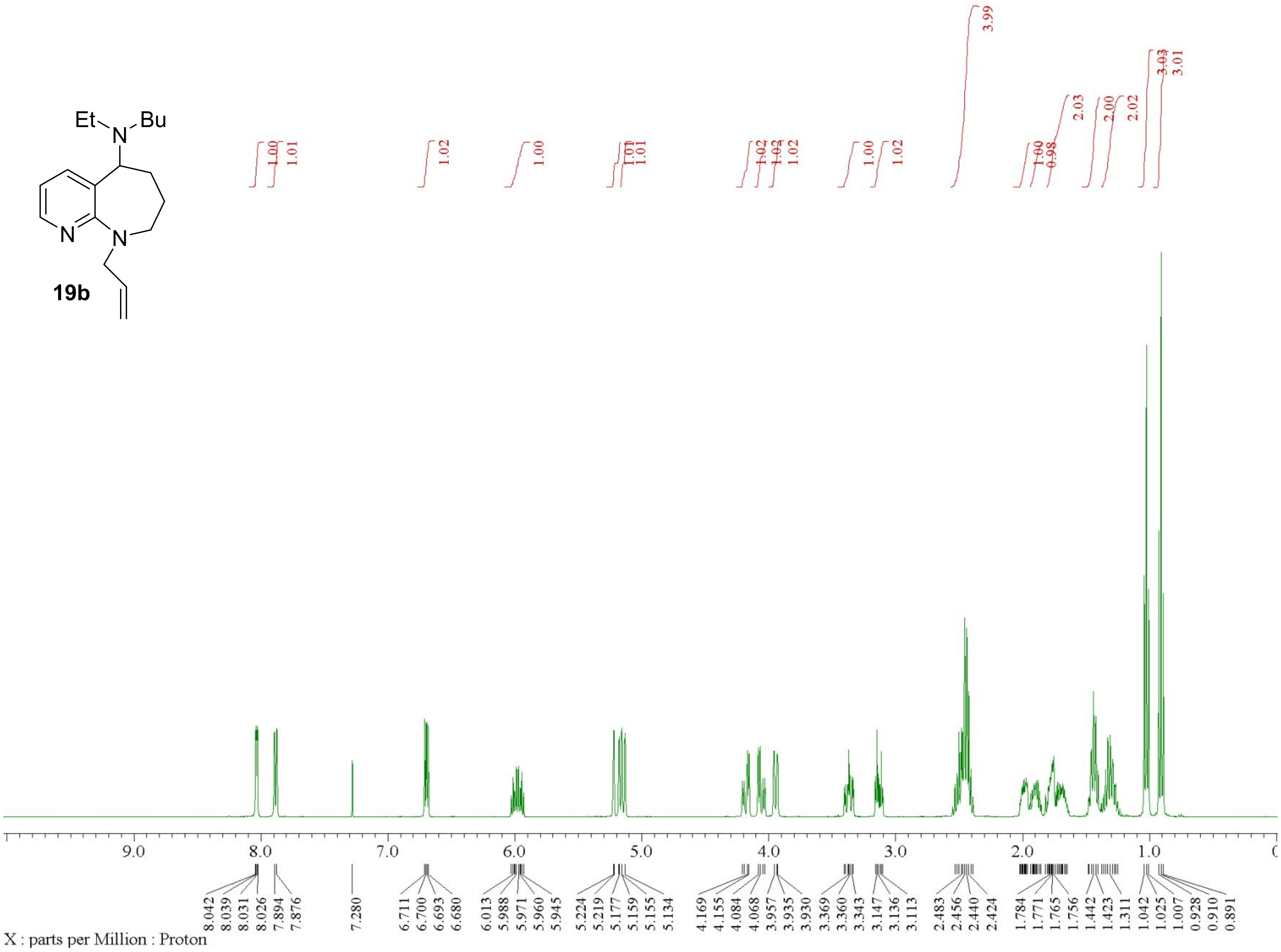

^1H NMR, 400 MHz, $CDCl_3$

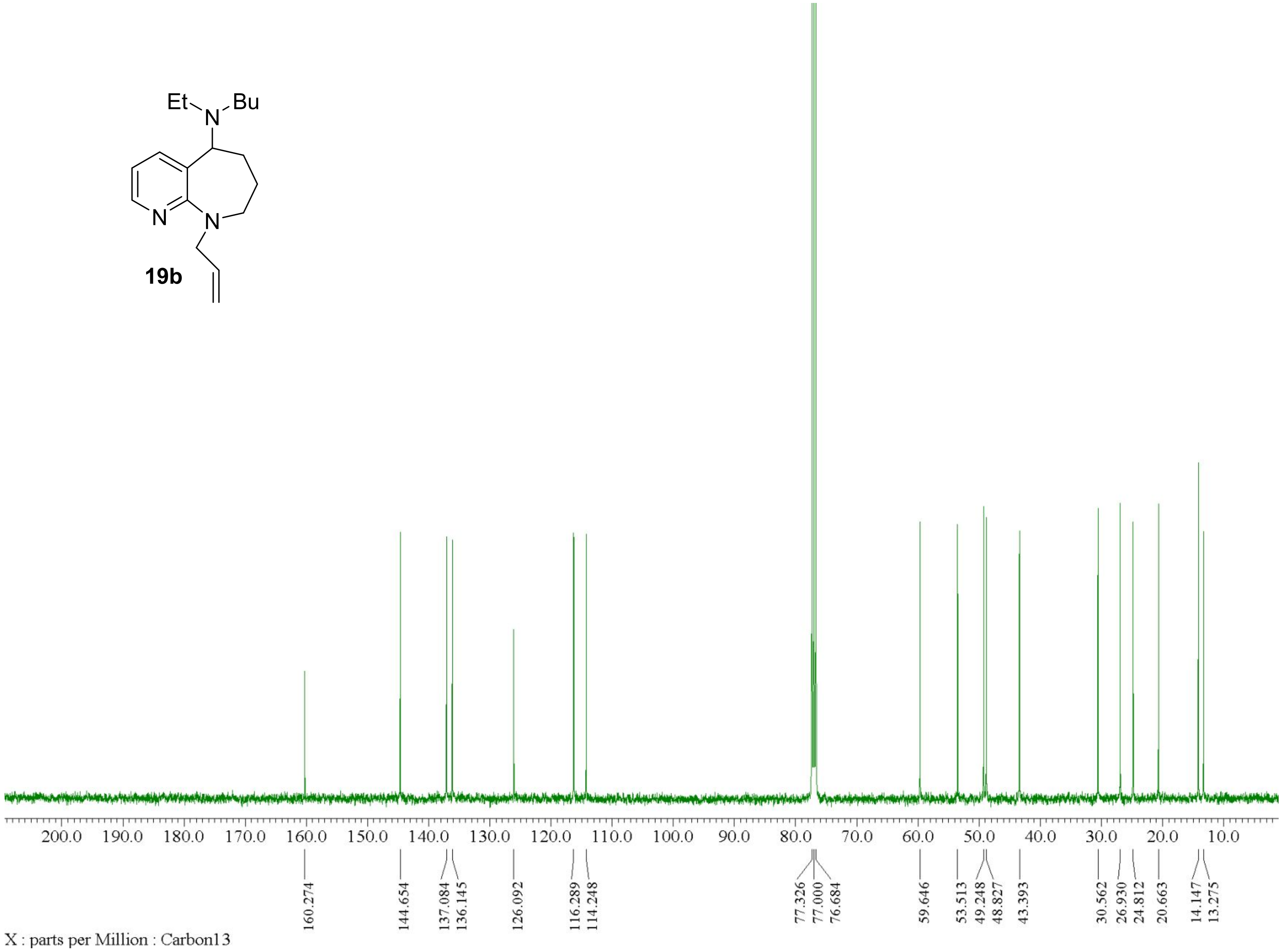

$^{13}C\{^1H\}$ NMR, 100 MHz, $CDCl_3$

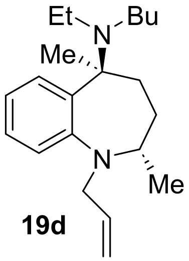

19d

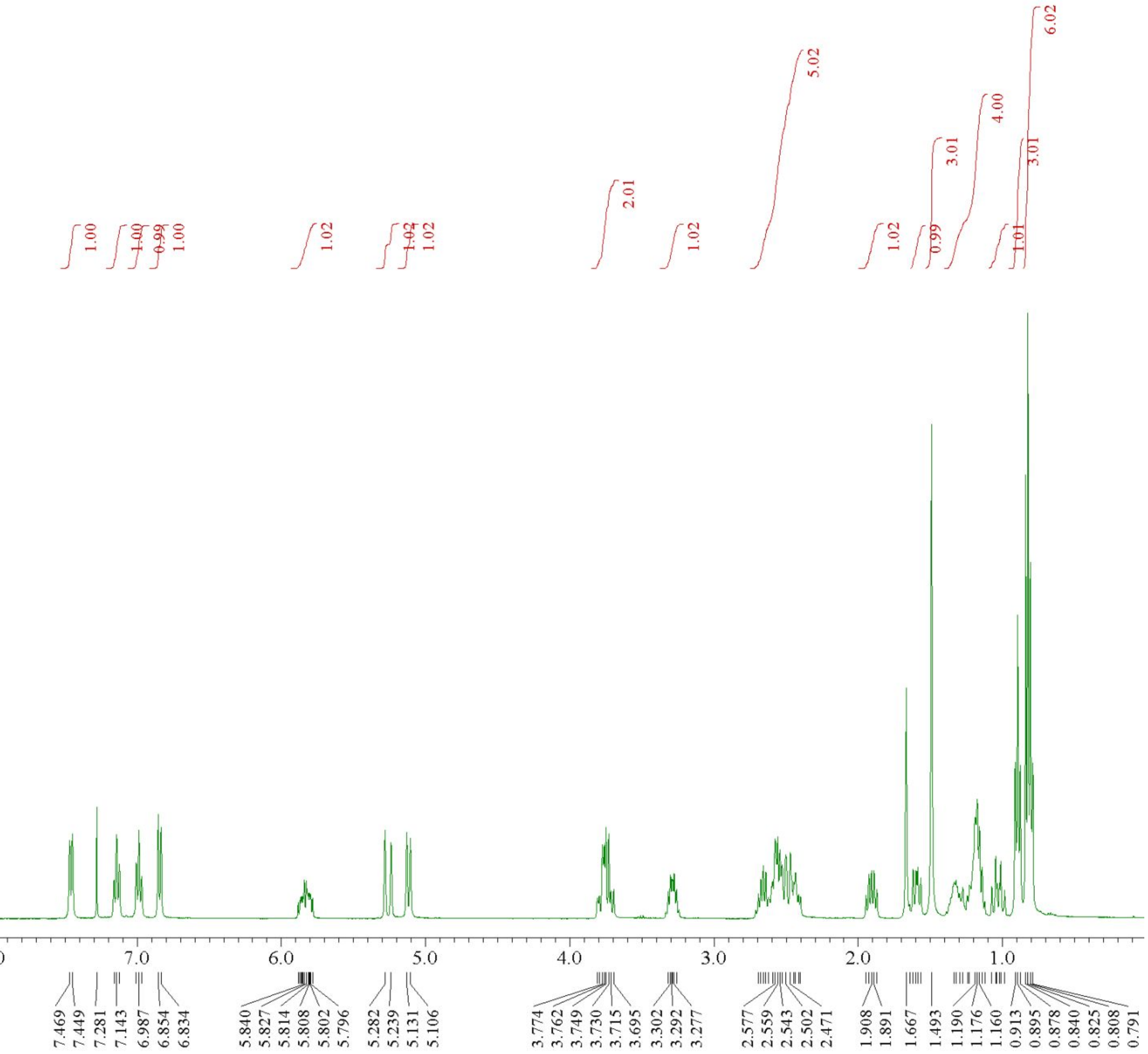

^{1}H NMR, 400 MHz, $CDCl_3$

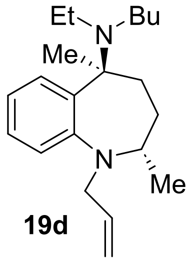

19d

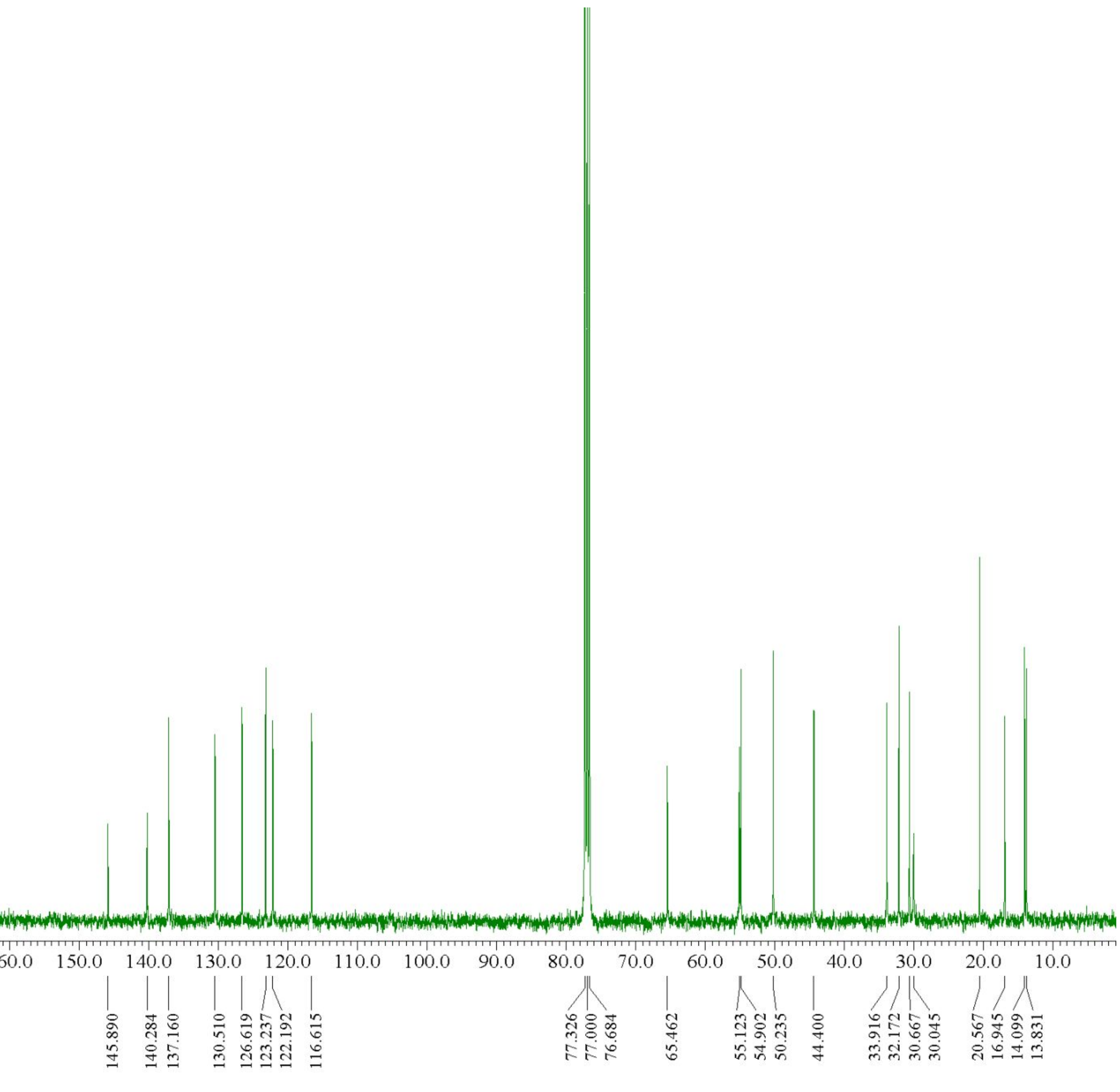

$^{13}C\{1H\}$ NMR, 100 MHz, $CDCl_3$

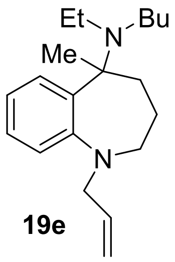

19e

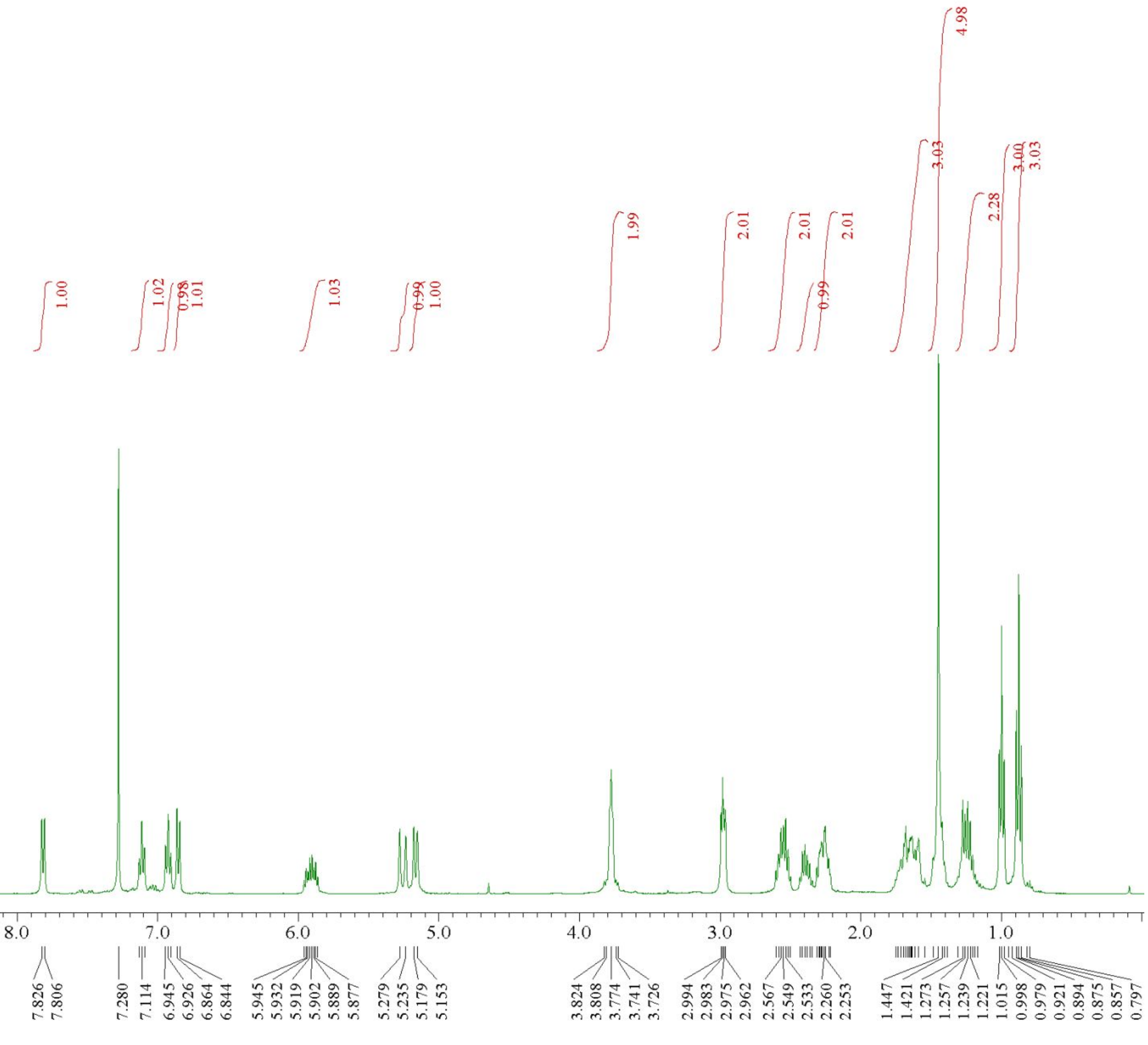

^{1}H NMR, 400 MHz, $CDCl_3$

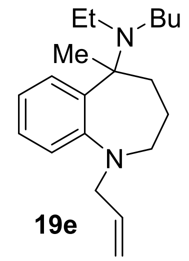

19e

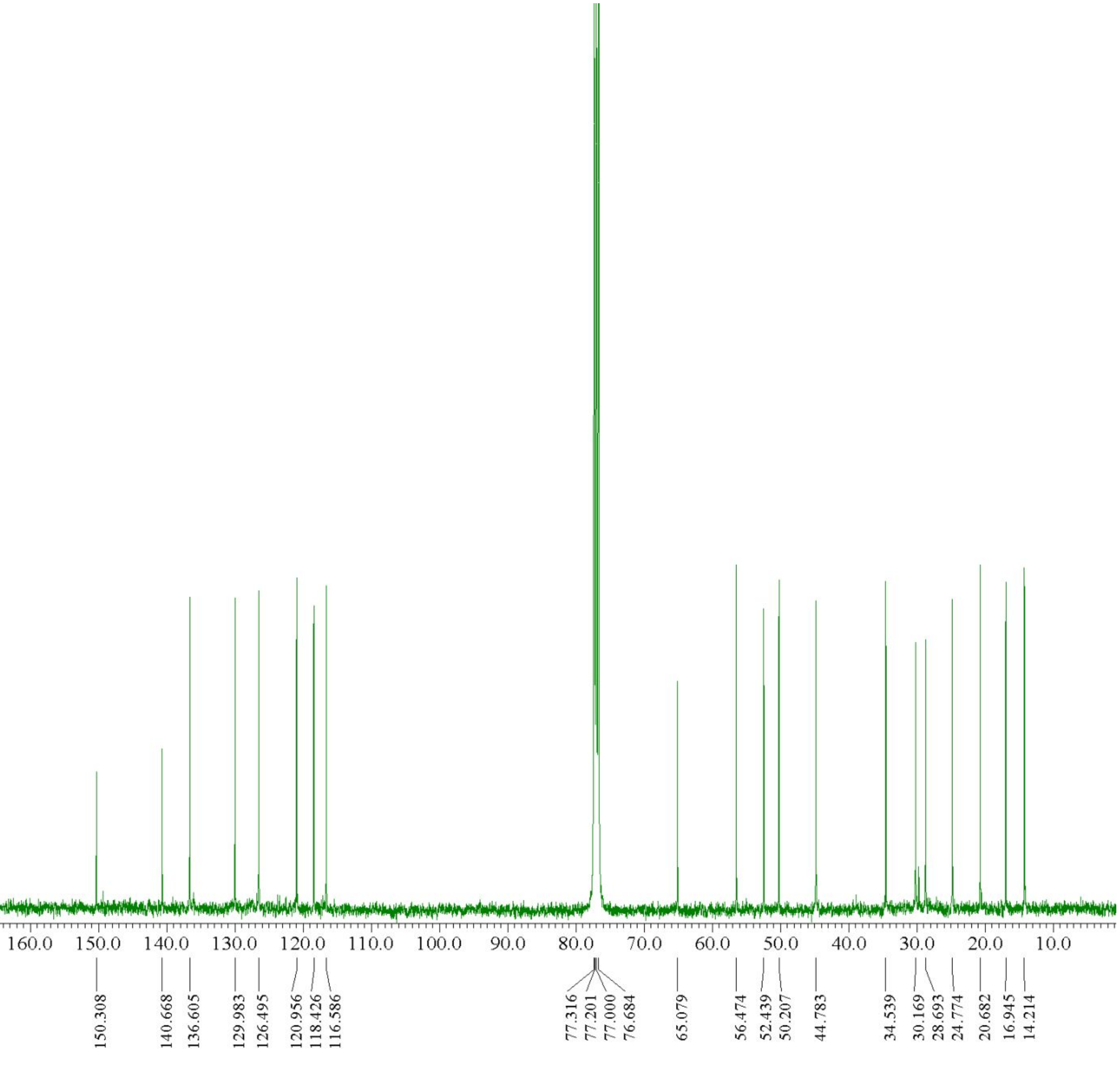

$^{13}C\{^1H\}$ NMR, 100 MHz, $CDCl_3$

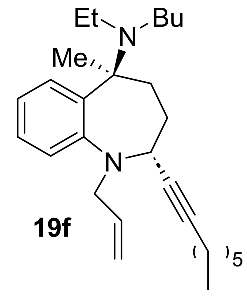

19f

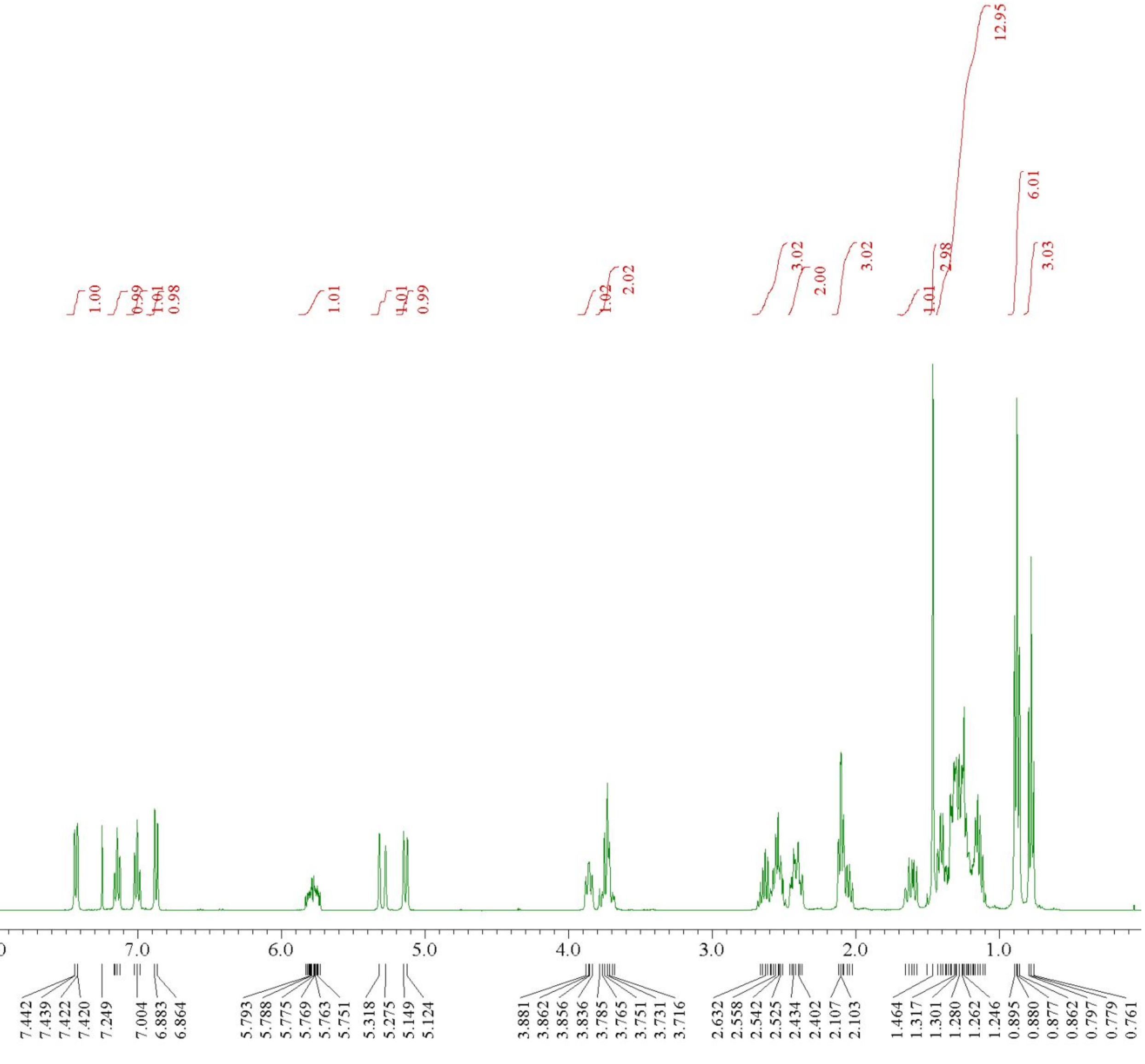

^{1}H NMR, 400 MHz, $CDCl_3$

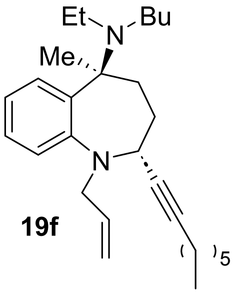

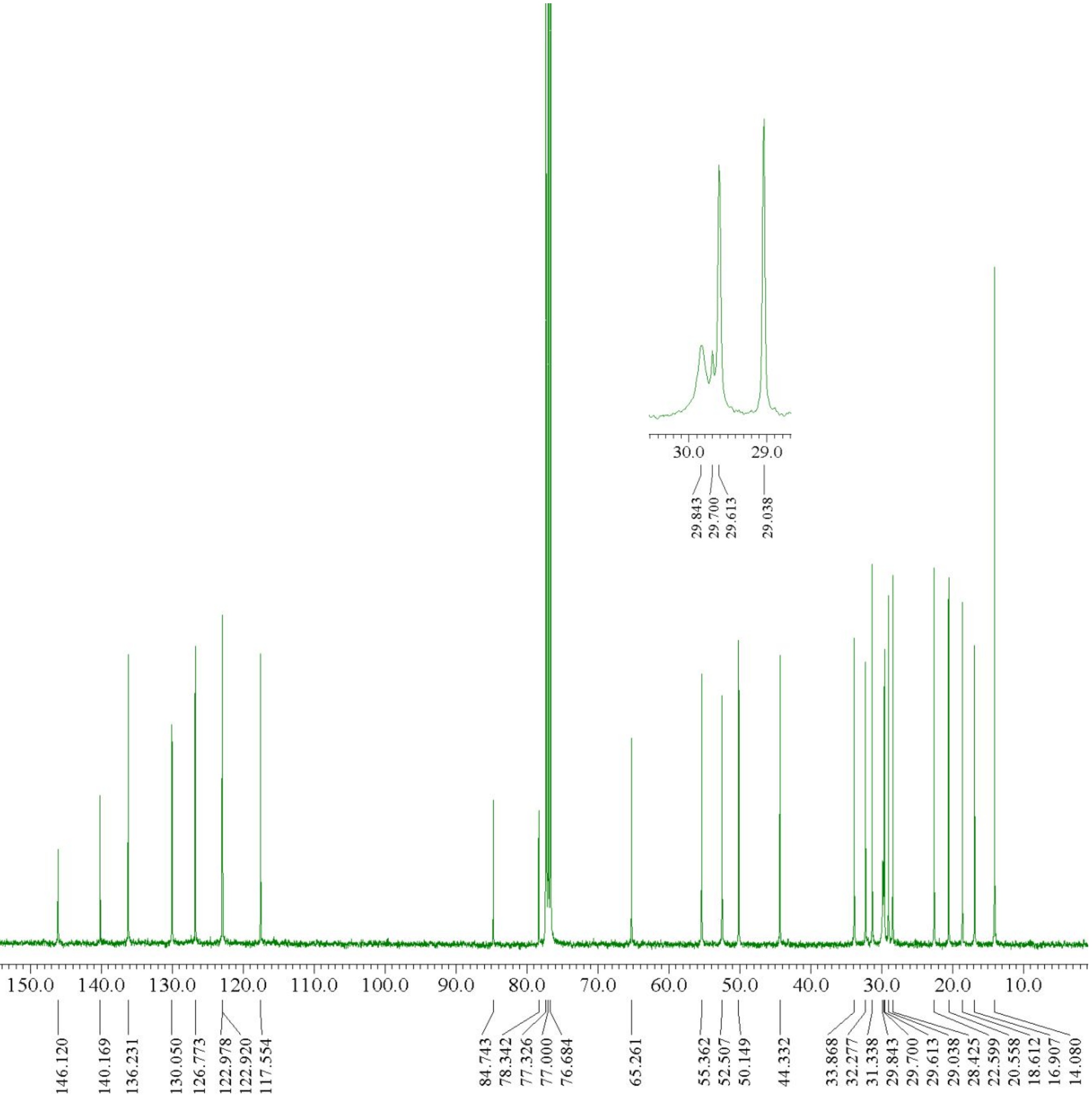

$^{13}C\{1H\}$ NMR, 100 MHz, $CDCl_3$

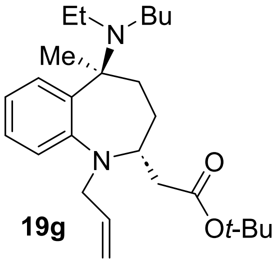

19g

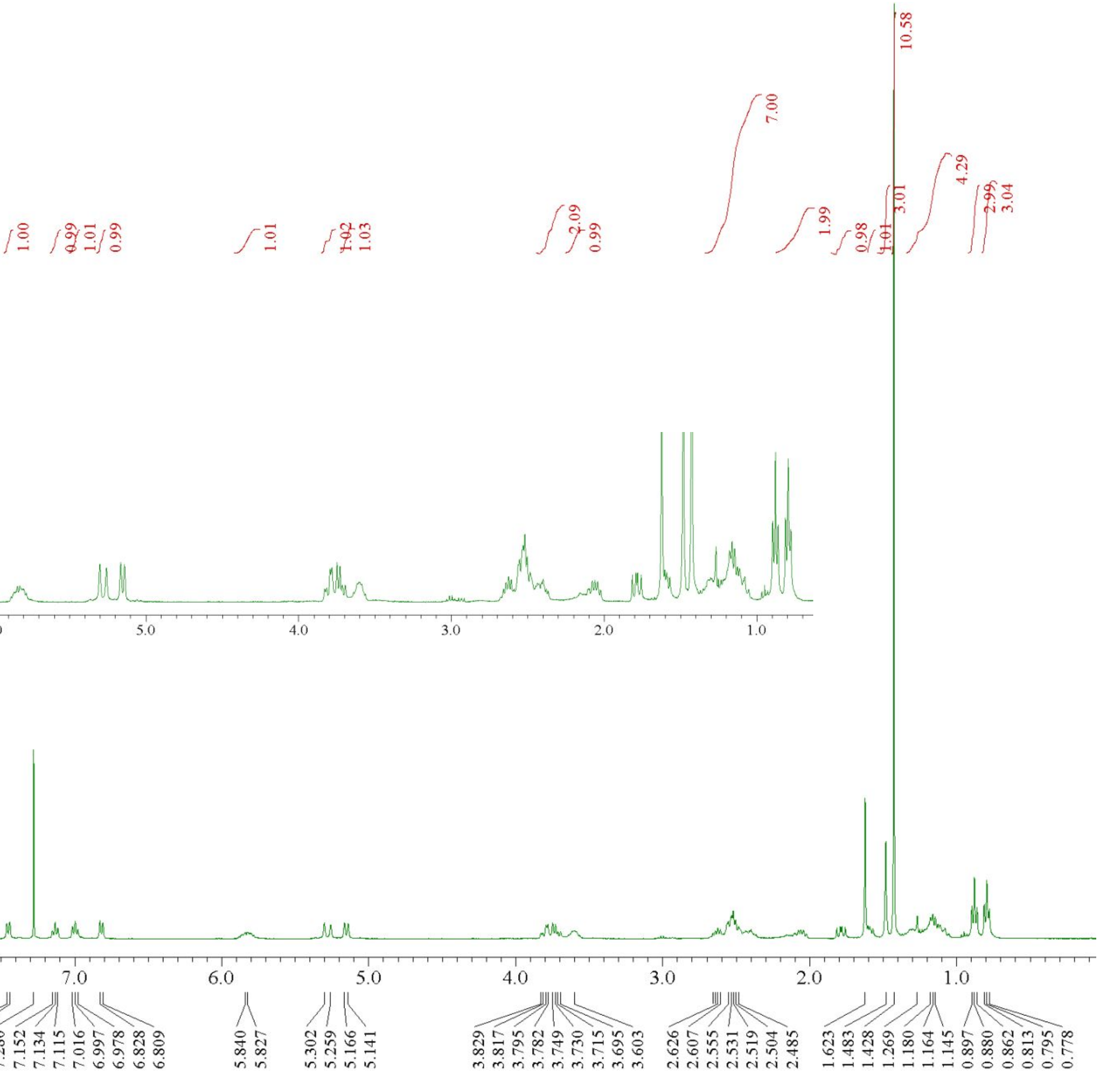

^{1}H NMR, 400 MHz, $CDCl_3$

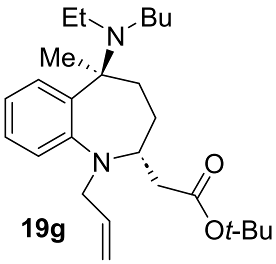

19g

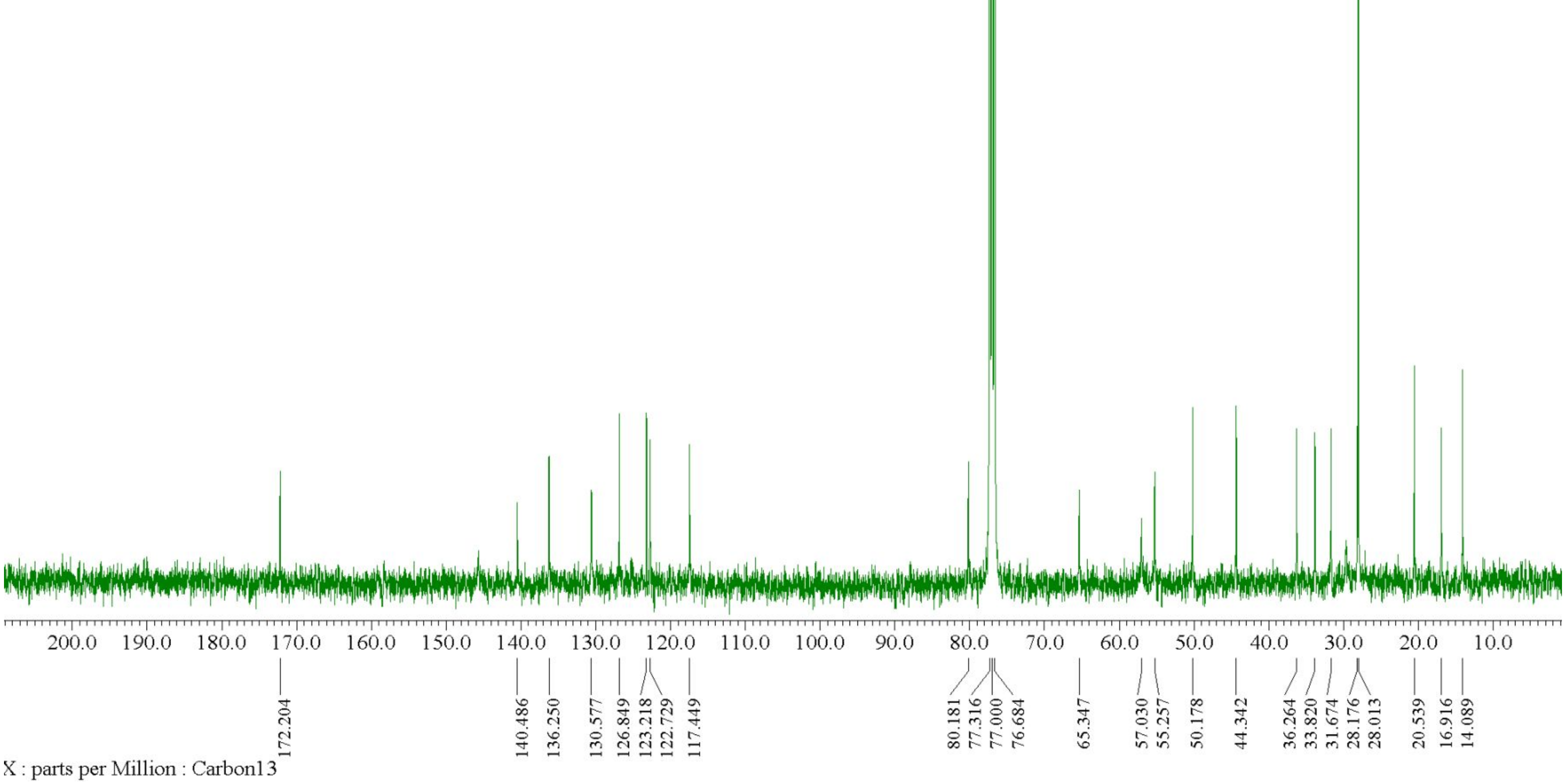

$^{13}C\{1H\}$ NMR, 100 MHz, $CDCl_3$

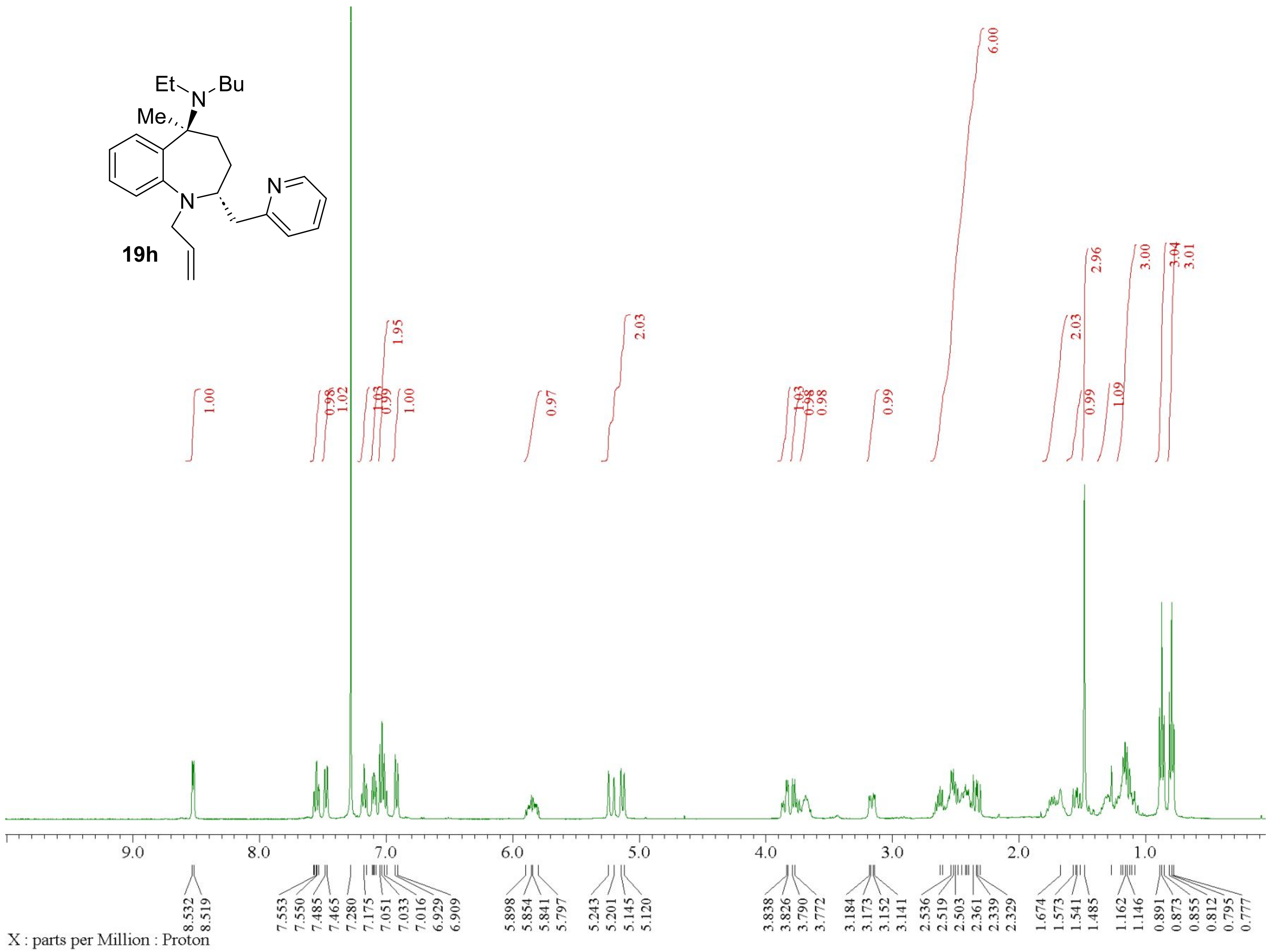

^{1}H NMR, 400 MHz, $CDCl_3$

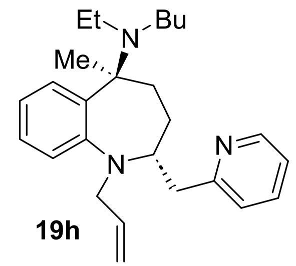

19h

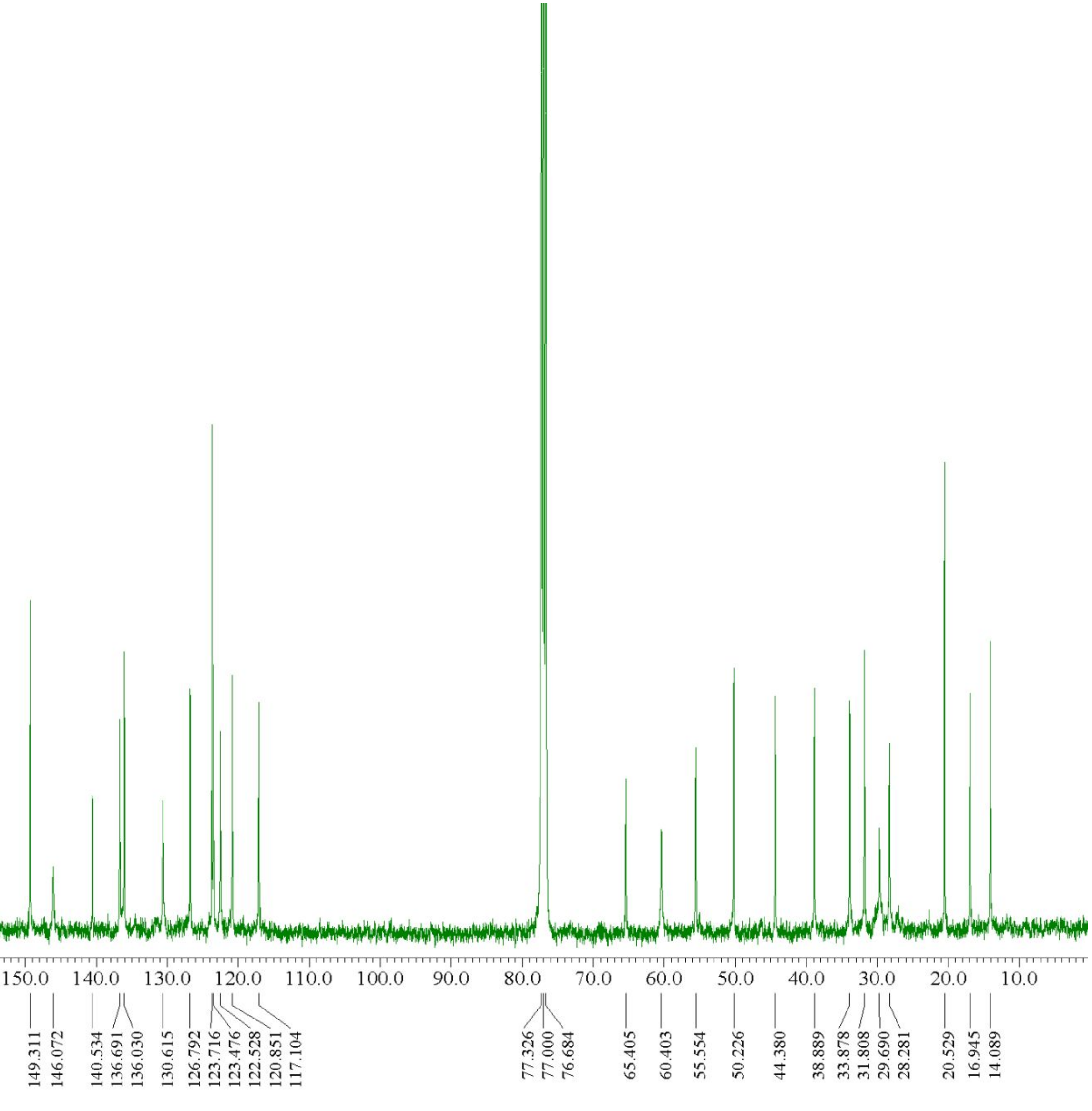

$^{13}C\{1H\}$ NMR, 100 MHz, $CDCl_3$

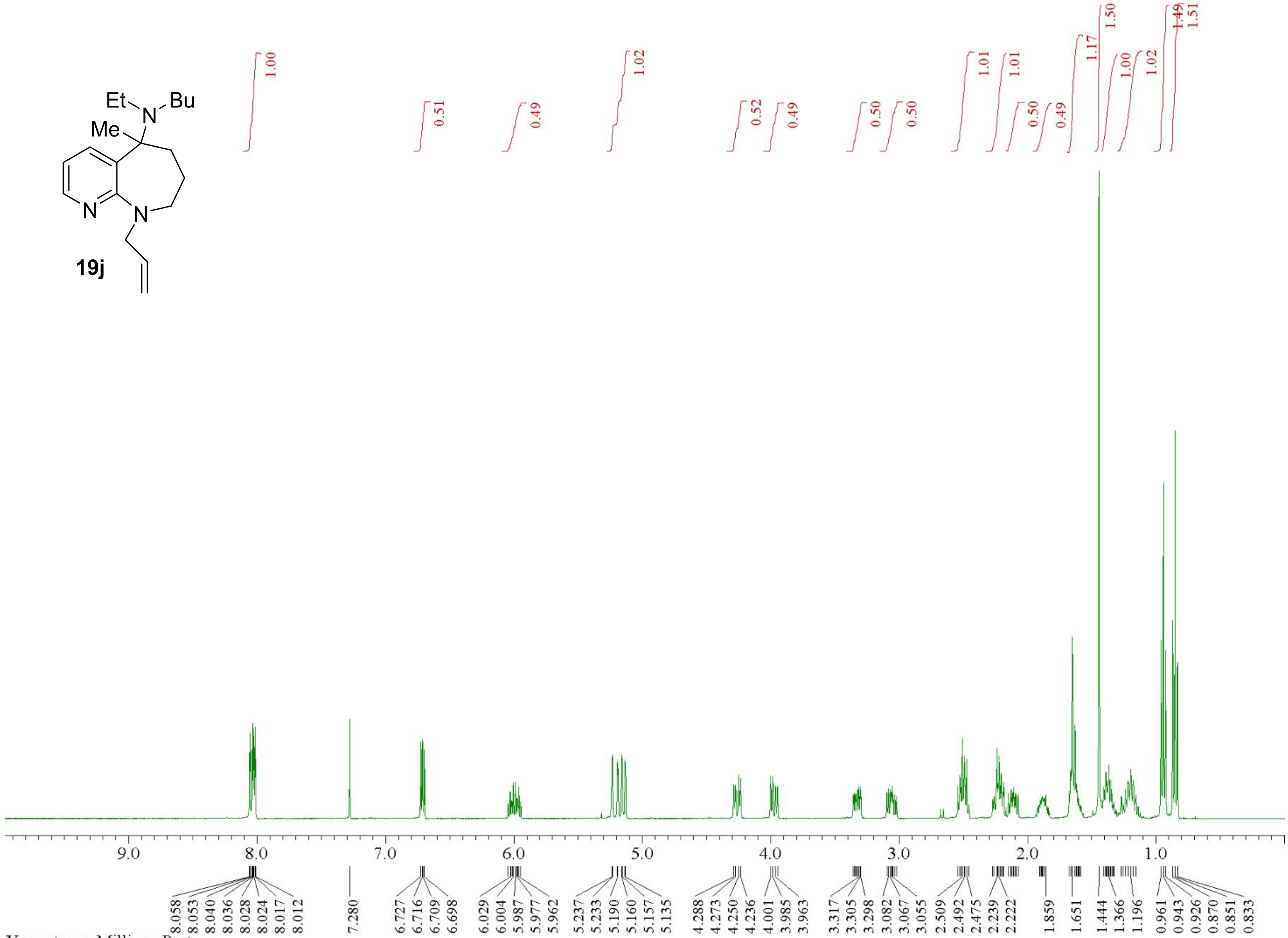

^{1}H NMR, 400 MHz, $CDCl_3$

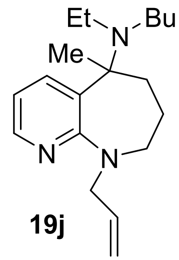

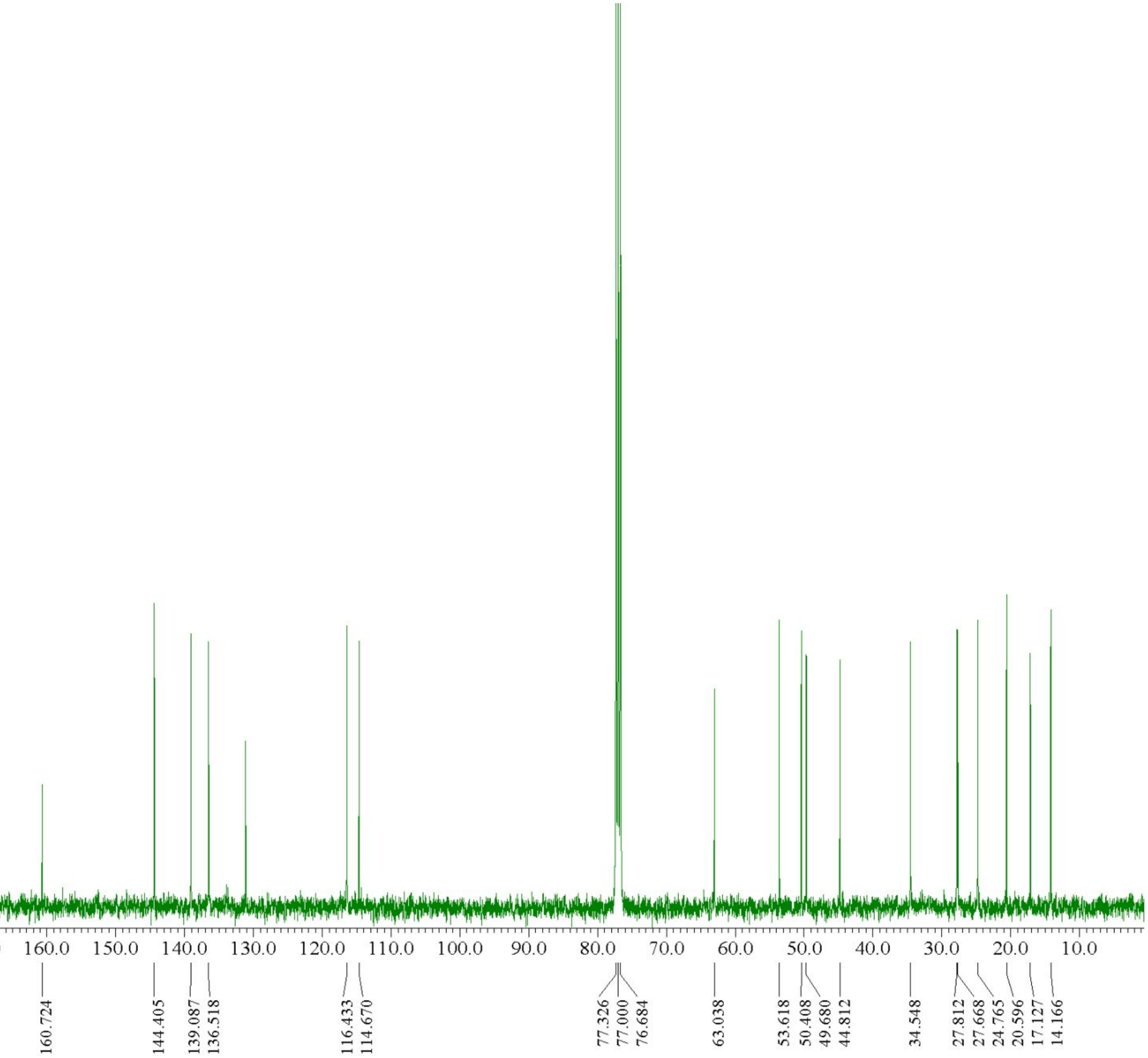

$^{13}C\{1H\}$ NMR, 100 MHz, $CDCl_3$

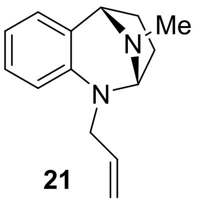

21

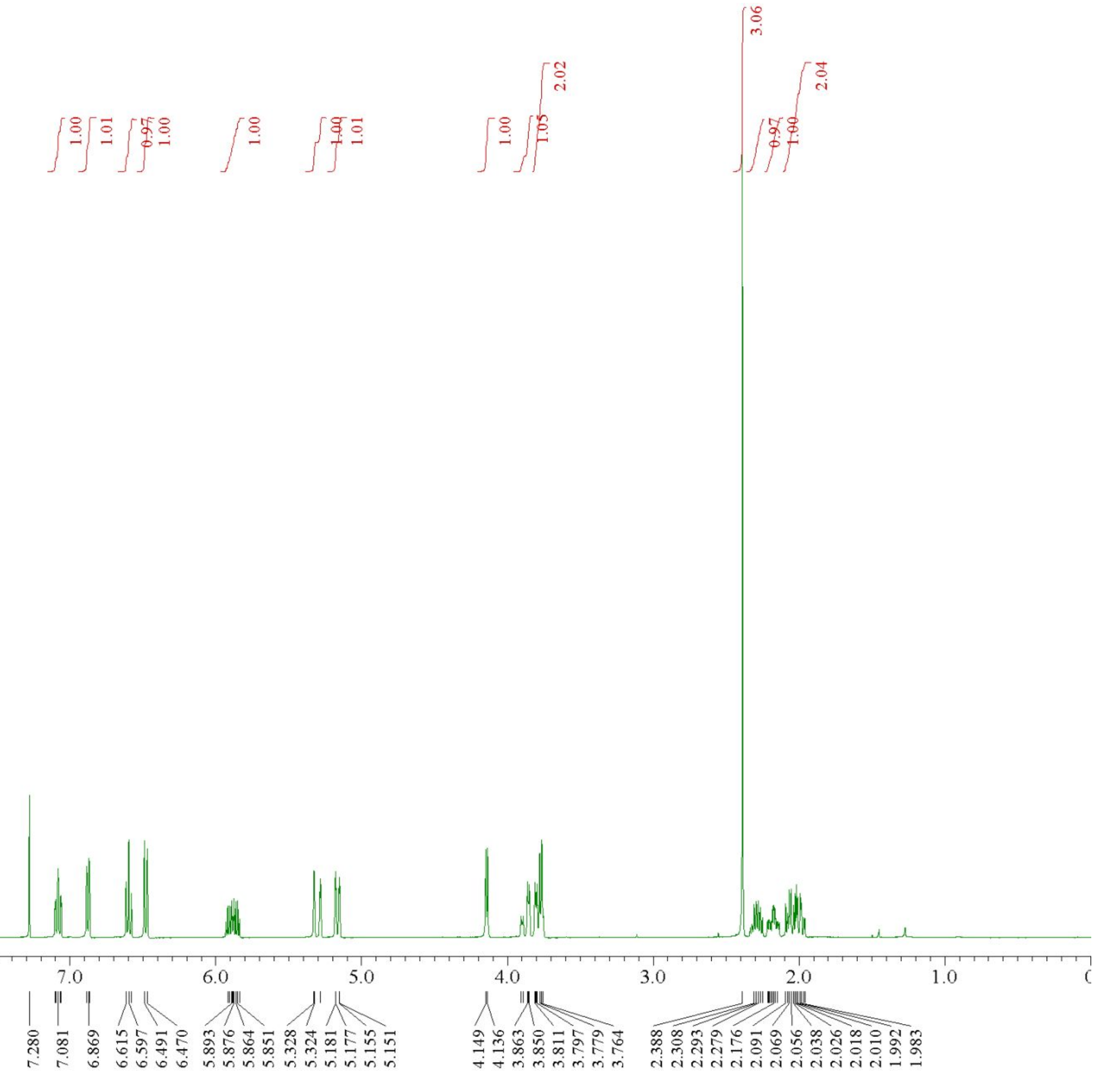

^{1}H NMR, 400 MHz, $CDCl_3$

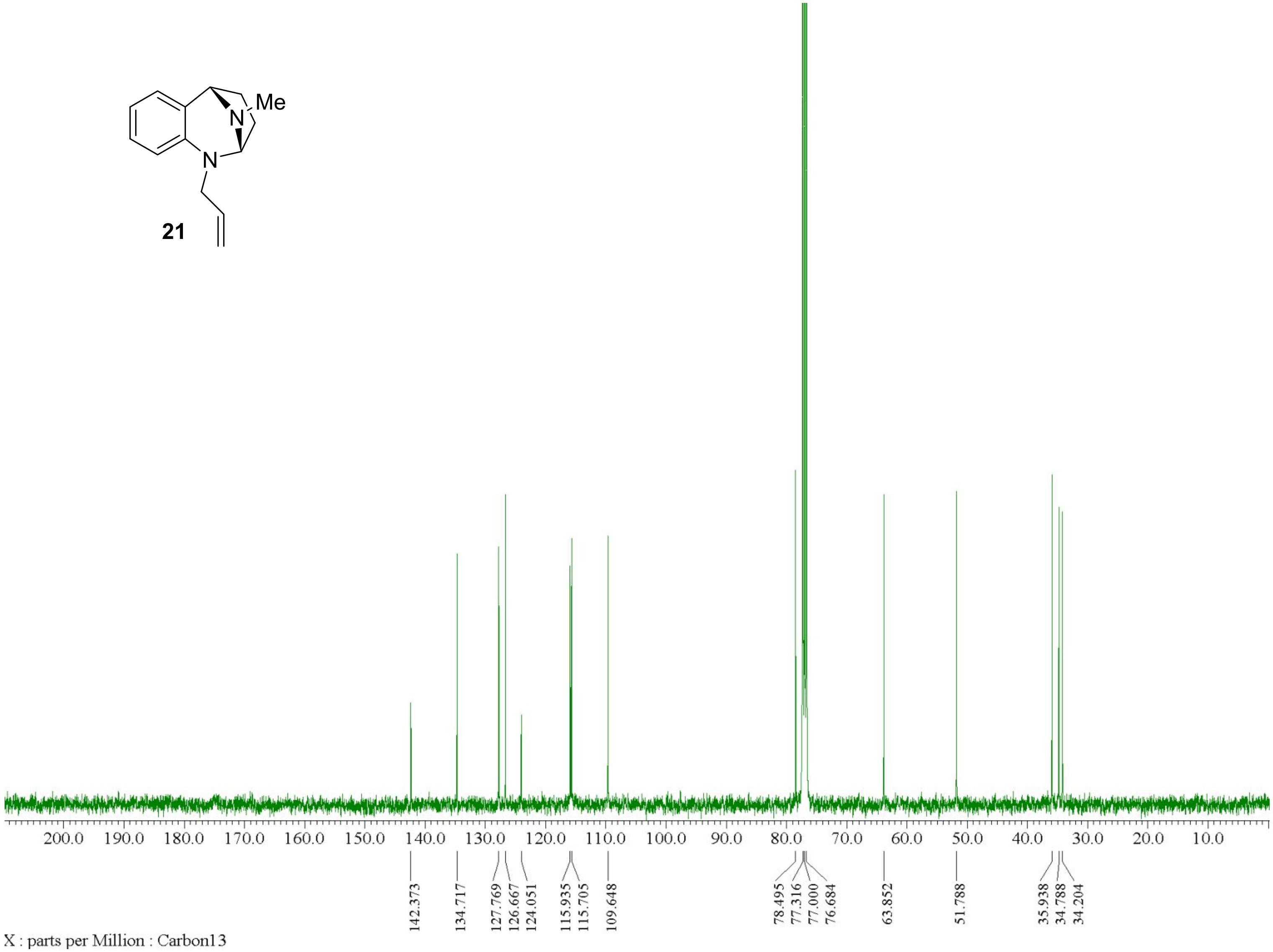

21

$^{13}C\{^1H\}$ NMR, 100 MHz, $CDCl_3$

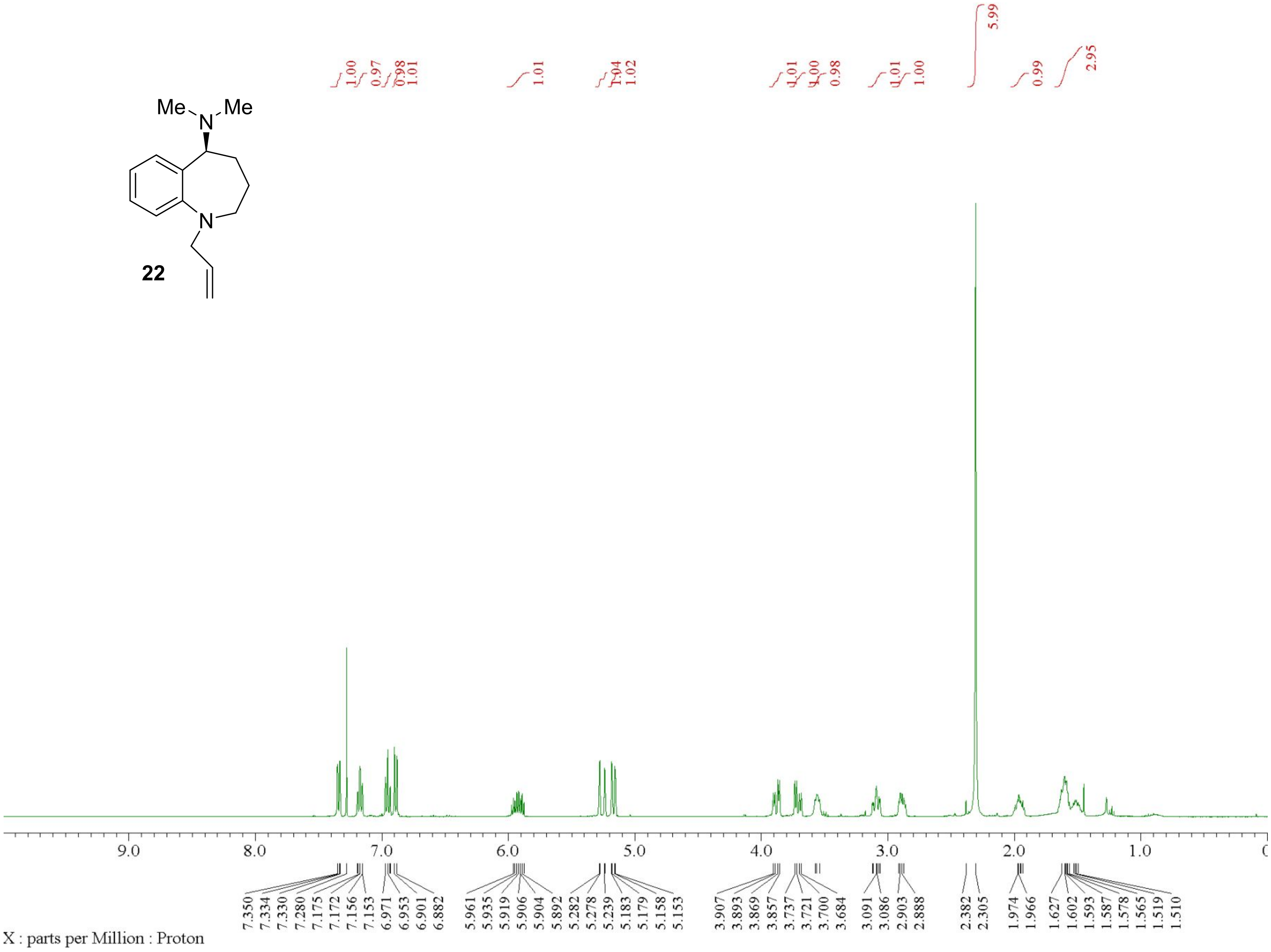

^1H NMR, 400 MHz, $CDCl_3$

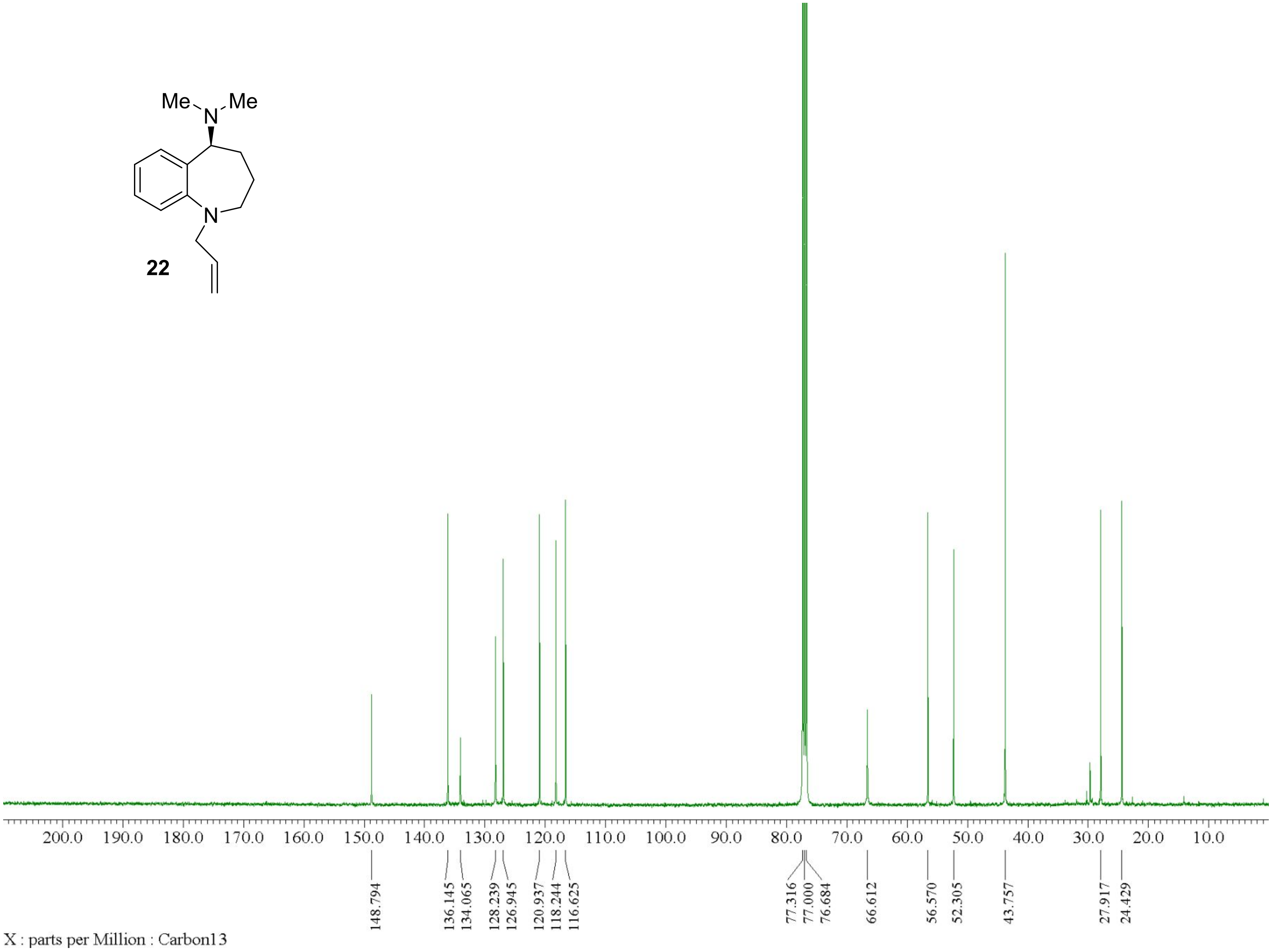

22

$^{13}C\{^1H\}$ NMR, 100 MHz, $CDCl_3$

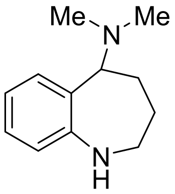

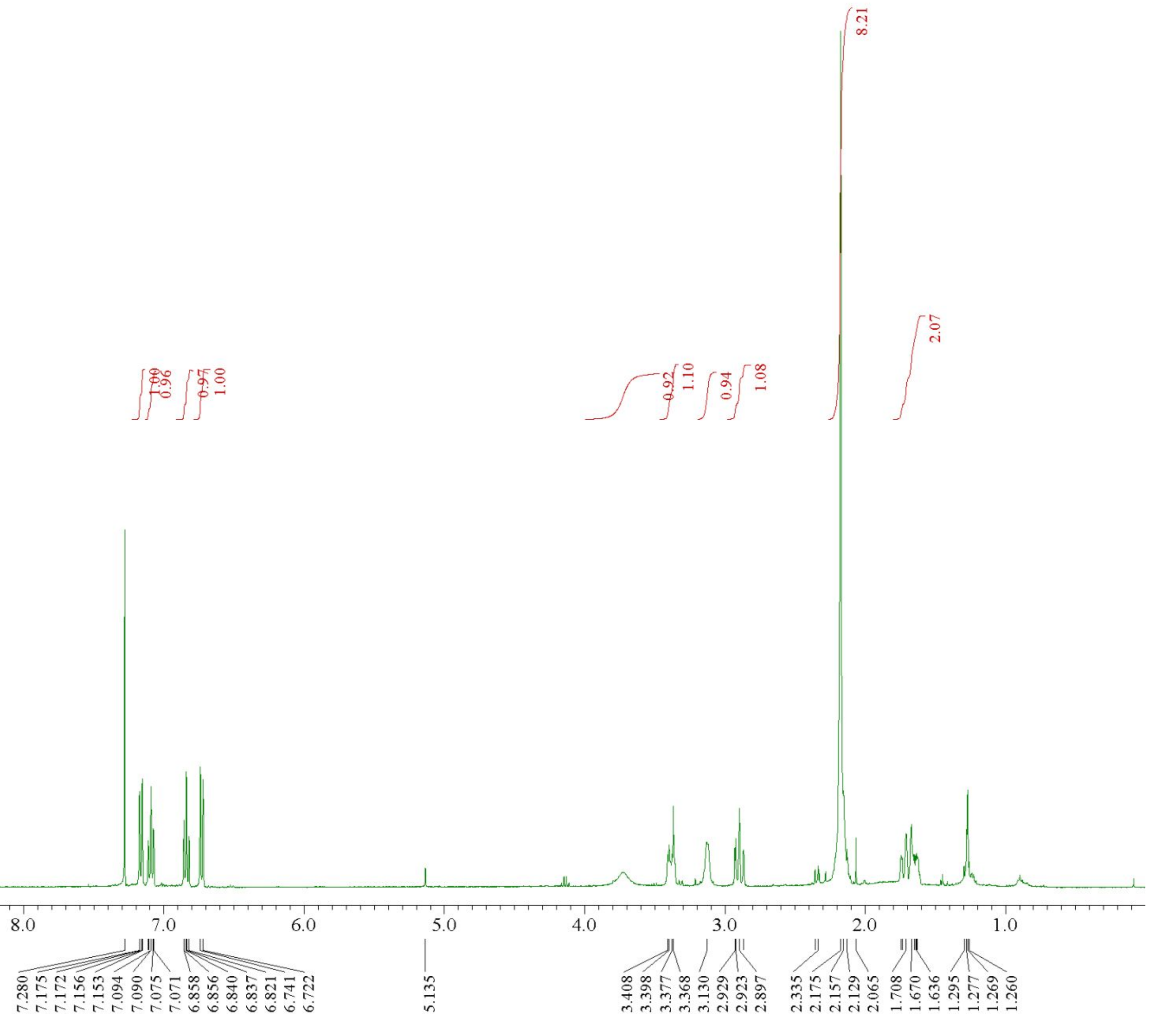

^1H NMR, 400 MHz, $CDCl_3$

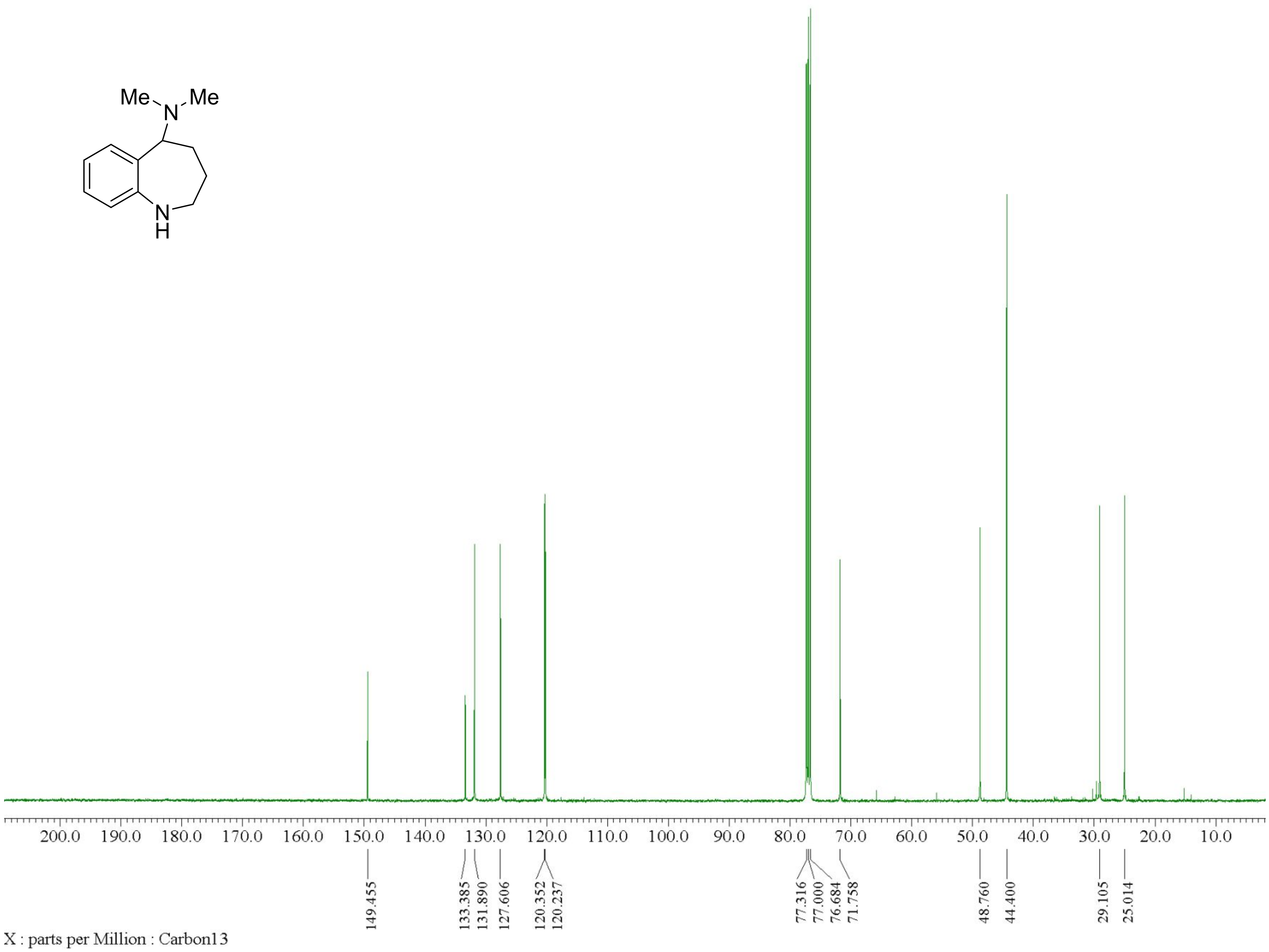

$^{13}C\{1H\}$ NMR, 100 MHz, $CDCl_3$

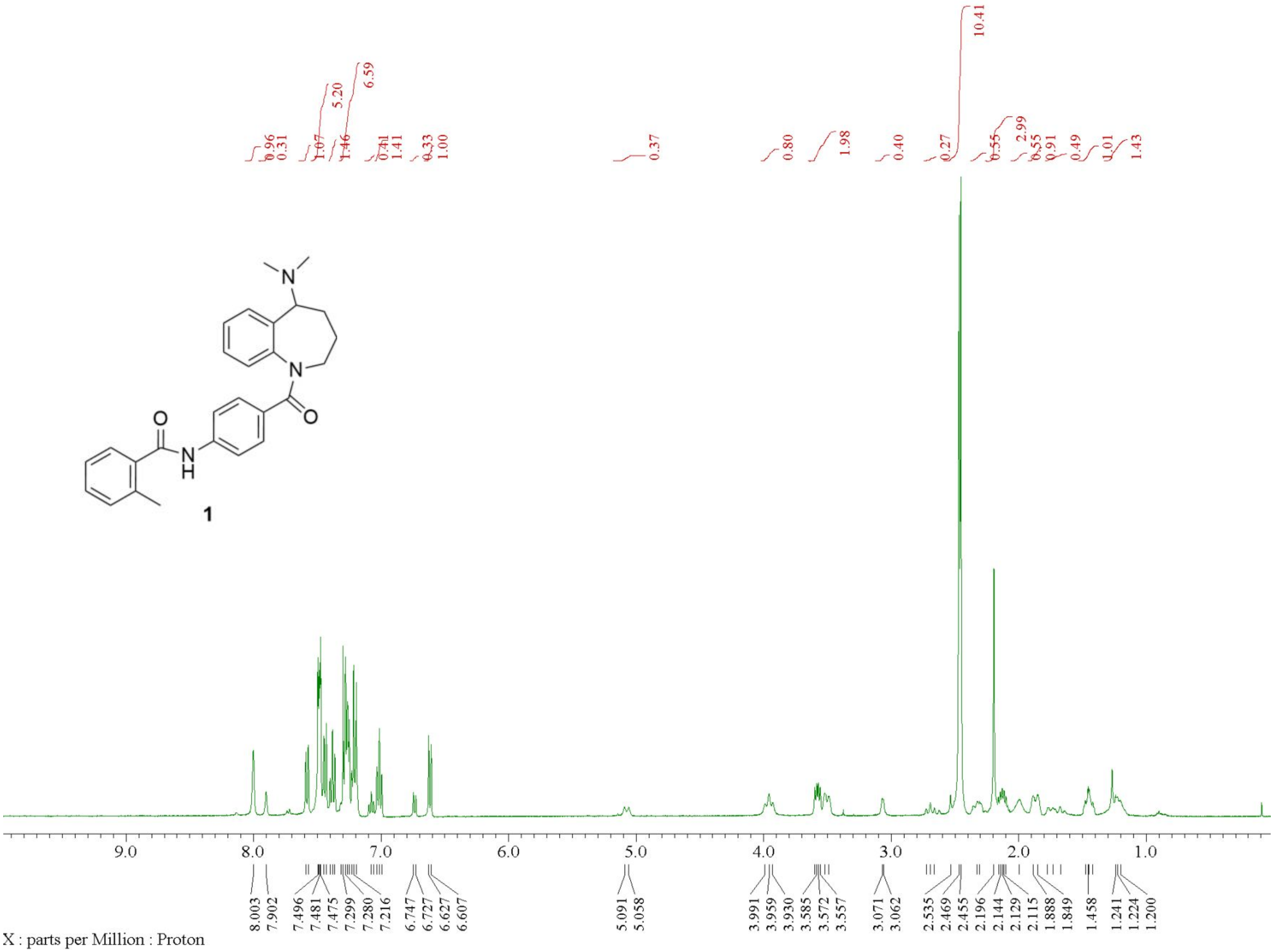

1

^1H NMR, 400 MHz, $CDCl_3$

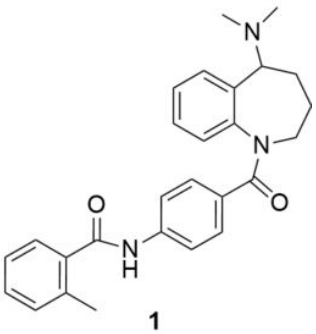

1

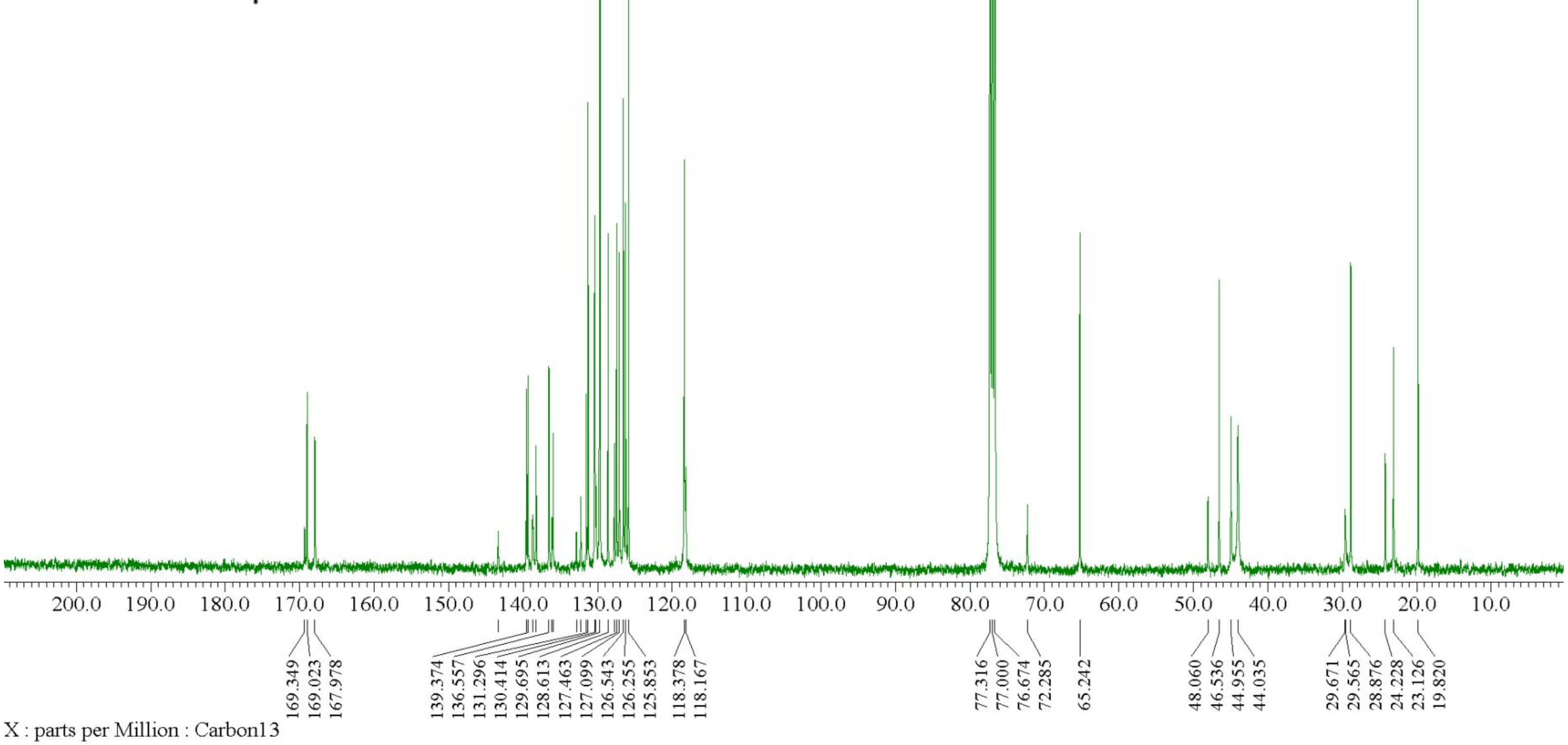

$^{13}C\{1H\}$ NMR, 100 MHz, $CDCl_3$

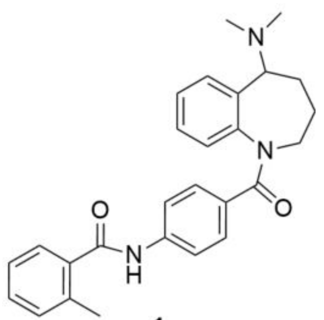

1

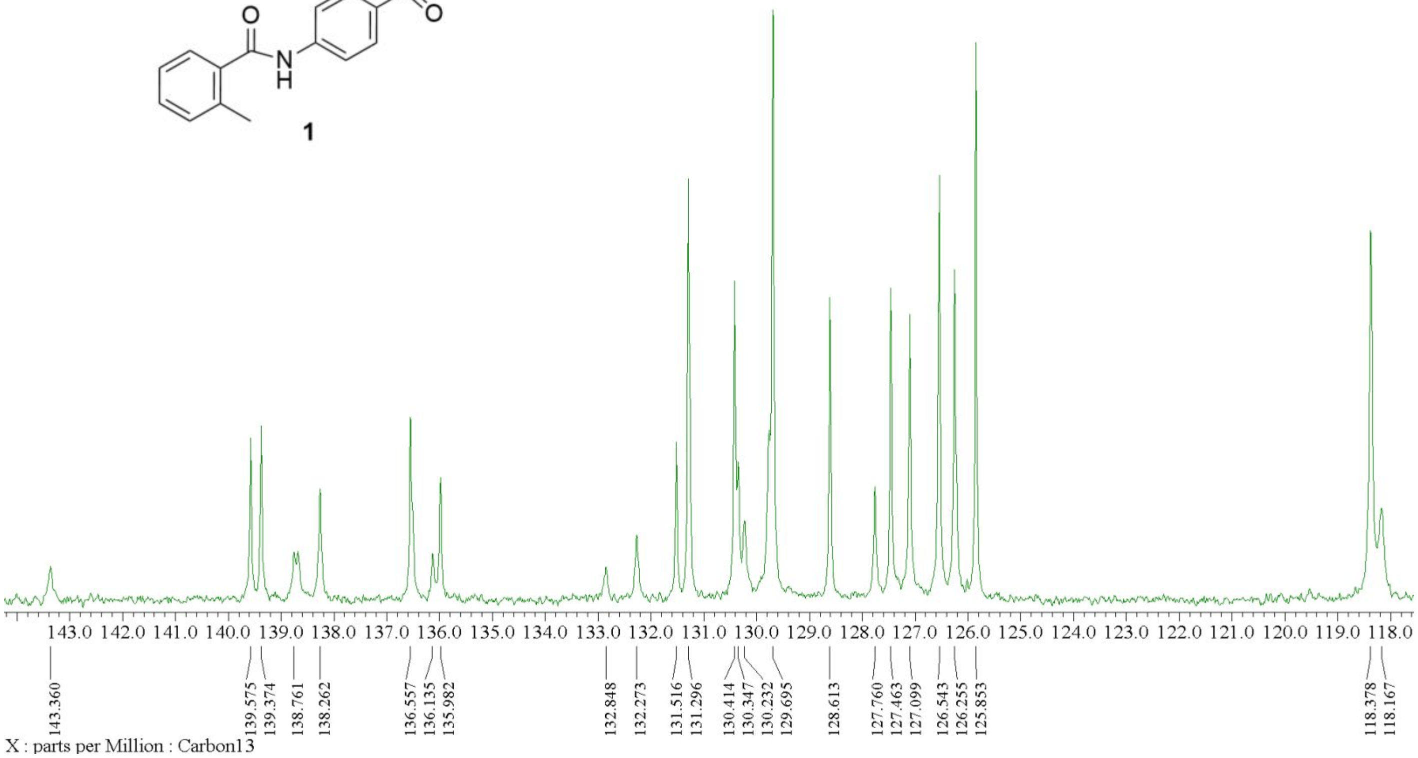

Expansion plot

$^{13}C\{1H\}$ NMR, 100 MHz, $CDCl_3$
